# Asymmetric [4 + 2] annulation of 5*H*-thiazol-4-ones with a chiral dipeptide-based Brønsted base catalyst†
†Electronic supplementary information (ESI) available. CCDC 1419574, 1419583, and 1463891. For ESI and crystallographic data in CIF or other electronic format see DOI: 10.1039/c6sc02039a


**DOI:** 10.1039/c6sc02039a

**Published:** 2016-06-09

**Authors:** Bo Zhu, Shuai Qiu, Jiangtao Li, Michelle L. Coote, Richmond Lee, Zhiyong Jiang

**Affiliations:** a Key Laboratory of Natural Medicine and Immuno-Engineering of Henan Province , Henan University , Kaifeng , Henan , People's Republic of China . Email: chmjzy@henu.edu.cn; b ARC Centre of Excellence for Electromaterials Science , Research School of Chemistry , Australian National University , Canberra ACT 2601 , Australia . Email: richmond.lee@anu.edu.au

## Abstract

Versatile synthetic strategies that access diverse chemical substrates in a highly chemo- and stereo-selective manner are crucial but demanding. Construction of chiral molecules with multiple (hetero)-quaternary carbon stereocenters in a single fashion is a particularly significant challenge, with important applications in the synthesis of a range of bioactive compounds containing the 1,4-sulfur bridged piperidinone structural motif. The asymmetric synthesis of these entities is complicated due to the need to build at least two hetero-quaternary stereocenters concurrently. In order to achieve this, we have developed a new family of dipeptide-based multifunctional Brønsted base organocatalysts that are highly capable of the first asymmetric [4 + 2] annulation reaction of 5*H*-thiazol-4-ones with electron-deficient alkenes. This protocol could be applied to a series of alkenes such as nitroalkenes, 4-oxo-4-arylbutenones, 4-oxo-4-arylbutenoates and methyleneindolinones, providing an efficient approach to valuable chiral 1,4-sulfur bridged piperidinones and their derivatives with multiple hetereo-quaternary stereogenic centers in high yields and enantioselectivities. Density functional theory studies involving 5*H*-thiazol-4-one and nitroolefin catalysis propose stereochemical insights into the origin of enantio- and chemo-selectivity.

## Abstract 2

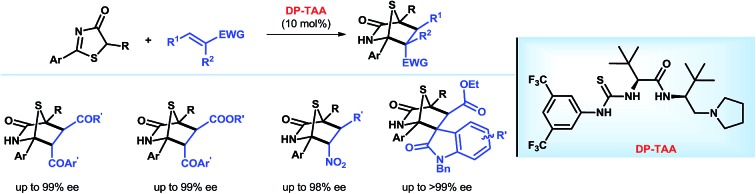
We have developed a new family of dipeptide-based multifunctional Brønsted base organocatalysts capable of the asymmetric [4 + 2] annulation of 5*H*-thiazol-4-ones with electron-deficient alkenes.

## Introduction

**Fig. 1:**
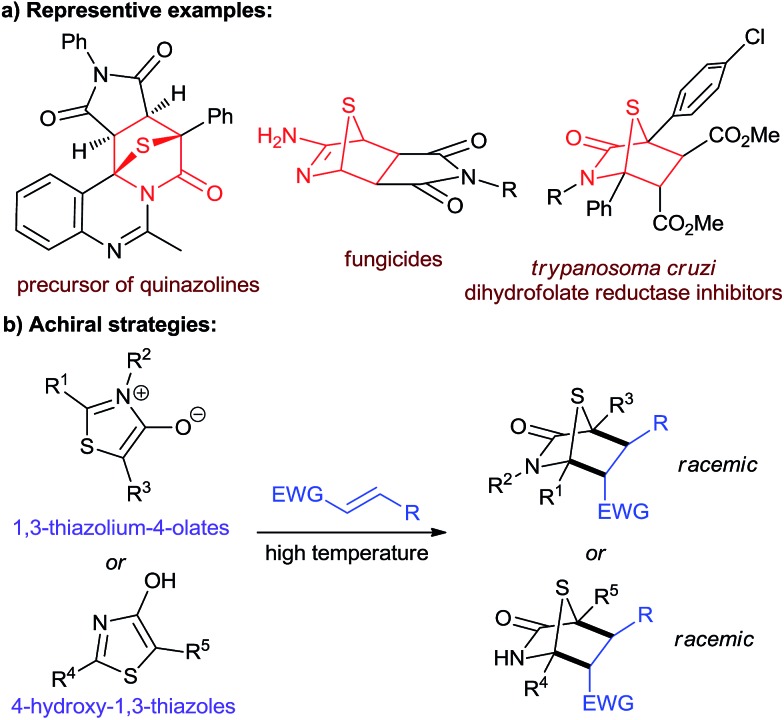
(a) Biologically significant molecules and synthetic targets. (b) Non asymmetric strategies by Foucaud *et al.* to obtain [4 + 2] products.

1,4-Sulfur bridged piperidinones and their related derivatives are important sulfur-containing structural motifs (Fig. 1a).^1^ They are key intermediates in the synthesis of multifarious biologically important heterocyclic compounds, such as quinazolines,1a–c imidazolidines,1*d* thiophenes,1e,f *etc.*1g–j Several of these molecules have been studied as potential fungicides1*k* and *Trypanosoma cruzi* dihydrofolate reductase inhibitors.1*l* Since the use of mesoionic 1,3-thiazolium-4-olates as 1,3-dipolar reagents was reported by Potts and co-workers in 1974,1g,^2^ cycloaddition reactions of 1,3-thiazolium-4-olates with electron-deficient alkenes under harsh reaction conditions have become the most common strategy for the construction of 1,4-sulfur bridged piperidinones (Fig. 1b).1g–j,^3^ As an alternative, in 1975, Foucaud and co-workers described an example using 4-hydroxy-1,3-thiazoles as dienes to undergo [4 + 2] annulation with dimethylmaleate and dimethylfumarate.^4^ Unfortunately, to date, no asymmetric method has yet been developed to access these entities in a non-racemic form.

The tautomers of 4-hydroxy-1,3-thiazoles, 5*H*-thiazol-4-ones,4*a* were featured in the asymmetric catalysis of a recent work by the Palomo group, who, through developing bifunctional ureidopeptide-based Brønsted base catalysts, established highly enantio- and diastereoselective conjugate addition to nitroalkenes.^5^ Their study demonstrated the efficacy of 5*H*-thiazol-4-ones as a new class of sulfur-containing pronucleophiles achieving α,α-disubstituted α-mercapto carboxylic acids. Several elegant asymmetric variants have thus been disclosed subsequently, including iridium-catalyzed allylation,^6^ conjugate addition to enones *via* hydrogen bonding (H-bonding) catalysis,^7^ and chiral phosphine-catalyzed γ-addition with allenoates.^8^ However asymmetric [4 + 2] annulation of 5*H*-thiazol-4-ones, behaving as 2-azadienes,^9^ to effectively and simultaneously build two heteroquaternary stereogenic centers still remains elusive.

**Fig. 2:**
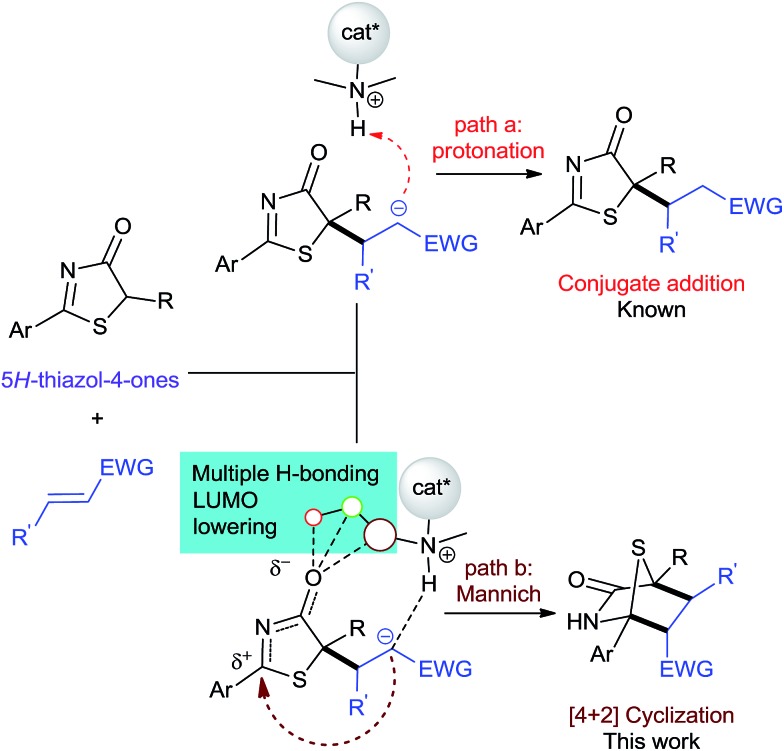
Two plausible paths in the asymmetric reaction between 5*H*-thiazol-4-ones and activated alkenes.

Intuitively, the addition reaction between 5*H*-thiazol-4-ones and electron-deficient alkenes first forms an anionic Michael adduct that could undergo two plausible pathways: protonation or a formal Mannich reaction (Fig. 2). Previous examples employing a bifunctional catalytic system^5^,^7^ showed that conjugate addition-protonation is favored. So in order for [4 + 2] annulation to proceed preferentially, we believe that the activation of the 5*H*-thiazol-4-one imine group is crucial in steering the reaction towards Mannich.

In this regard, we envisioned that a versatile multifunctional catalyst^10^ could help realize this through judicious H-bonding interactions. In recent years, we have employed amino acid-based (thio)urea–tertiary amines and their derivatives as bifunctional Brønsted base catalysts for enantioselective reactions.^11^ In continuation of our pursuits in this burgeoning synthetic field, we herein report a novel family of dipeptide-based10d–g,^12^,^13^ (thio)urea–amide–tertiary amine multifunctional Brønsted base catalysts, enabling the first catalytic asymmetric [4 + 2] cyclization reaction between 5*H*-thiazol-4-ones and electron-deficient alkenes with an extremely broad substrate scope: nitroalkenes, *trans*-4-oxo-4-arylbutenones, *trans*-4-oxo-4-arylbutenoates and methyleneindolinones. This new methodology allows highly chemo- and enantio-selective construction of diverse chiral 1,4-sulfur bridged piperidinone architectures, featuring more than two hetereo-quaternary stereogenic centers.

## Results and discussion

### Optimization of reaction conditions

Our investigations began by examining a model reaction between 5*H*-thiazol-4-one **1a** and nitroalkene **2a** (Table 1). Inferring from Palomo's work, *vide supra*, it was demonstrated that the reaction between 5*H*-thiazol-4-ones and nitroolefins utilizing a ureidopeptide-based catalyst favors conjugate addition-protonation.^5^ In the preliminary study, a reaction using achiral Et_3_N catalyst in CH_2_Cl_2_ at 25 °C afforded the conjugate adduct **4** in 36% yield and no trace of the [4 + 2] annulation product (entry 1). When l-*tert*-leucine-based tertiary amines **I**,**II**, were enlisted as catalysts, we observed a slight improvement in the yield of the [4 + 2] annulation product albeit in the region of low enantioselectivity (entries 2 and 3).

**Table 1:** Optimization of the reaction between **1a** with **2a**^*a*^

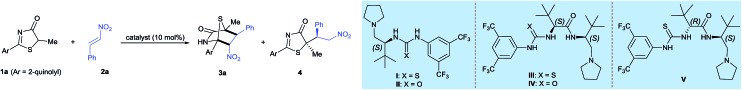											
Entry	Catalyst	Solvent	*T* (°C)	*t* (h)	**3a** : **4**	**3a**			**4**		
						Yield^*b*^ (%)	ee^*c*^ (%)	dr^*d*^	Yield^*b*^ (%)	ee^*c*^ (%)	dr^*d*^
1	Et_3_N	CH_2_Cl_2_	25	18	Trace	Trace	n.a.	—	36	n.a.	n.a.
2	**I**	CH_2_Cl_2_	25	12	1 : 1	42	0	15 : 1	45	64	1 : 1
3	**II**	CH_2_Cl_2_	25	12	3 : 1	62	7	9 : 1	20	3	19 : 1
4	**III**	CH_2_Cl_2_	25	12	4 : 1	73	86	19 : 1	13	88	12 : 1
5	**IV**	CH_2_Cl_2_	25	12	4 : 1	68	87	8 : 1	18	87	9 : 1
6	**III**	THF	25	16	Trace	Trace	n.a.	n.a.	Trace	n.a.	n.a.
7	**III**	Toluene	25	16	5 : 1	84	94	>19 : 11	8	88	12 : 1
8	**III**	Et_2_O	25	16	4 : 1	70	92	>19 : 1	11	96	>19 : 1
9	**III**	CHCl_3_	25	16	4.5 : 1	72	83	>19 : 1	9	85	>19 : 1
10^*e*^	**III**	Toluene	10	36	4 : 1	56	95	>19 : 1	10	31	3 : 1
11	**III**	Toluene	30	18	4.5 : 1	78	92	19 : 1	12	75	19 : 1
12^*e*^	**III**	Toluene	25	18	5 : 1	83	95	>19 : 1	11	88	12 : 1
13^*e*^	**V**	Toluene	25	18	2.5 : 1	68	–75	>19 : 1	17	13	>19 : 1

^*a*^The reaction was performed using **1a** (0.05 mmol) and **2a** (0.075 mmol) in 1.0 mL of solvent.

^*b*^Isolated yield based on **1a** after column chromatography.

^*c*^Enantiomeric ratio of product **3a** was determined *via* chiral phase HPLC analysis.

^*d*^Determined using crude ^1^H NMR analysis.

^*e*^The reaction was performed using **1a** (0.2 mmol) and **2a** (0.4 mmol) in 4.0 mL of solvent.

Given the moderate enantioselectivity with l-*tert*-leucine-based tertiary amine catalysts **I**,**II**, we further prototyped a new chiral dipeptide-based thiourea–amide–tertiary amine (DP–TAA) catalyst **III** constructed with l-*tert*-leucine units, of which the two molecules of l-*tert*-leucine are linked by an amido bond (see ESI†). Catalyst **III** performed extremely well and led to an unprecedented boost in reactivity and stereoselectivity: **3a** in 73% yield with 86% enantiomeric excess (ee) was attained (Table 1, entry 4). By modifying the thiourea unit of **III** to urea, we synthesized a new dipeptide-based urea–amide–tertiary amine (DP–UAA) catalyst **IV**. Catalyst **IV** performed less ideally yield-wise but gave a good ee (entry 5). Thus DP–TAA **III** was selected as the catalyst and tested against various solvents (entries 6–9) but toluene was eventually selected as the optimal solvent. By tuning the reaction temperature (entries 10 and 11), we observed that the yield of **3a** was reduced but that ee remained high at 10 °C. At a slightly higher temperature of 30 °C, no large detrimental changes to yield and ee were seen. Using the optimal conditions, the reactions were performed again with higher substrate loadings in toluene at 25 °C, affording the [4 + 2] adduct **3a** in 83% yield and 95% ee (entry 12). By modifying the catalyst structure and introducing d-*tert*-leucine as the second amino acid unit (DP–TAA **V**), the enantiomer of **3a**, ***ent-*3a**, was obtained in 68% yield and with moderate enantioselectivity (entry 13).

### Expanding the scope of nitroalkenes

**Scheme 1:**
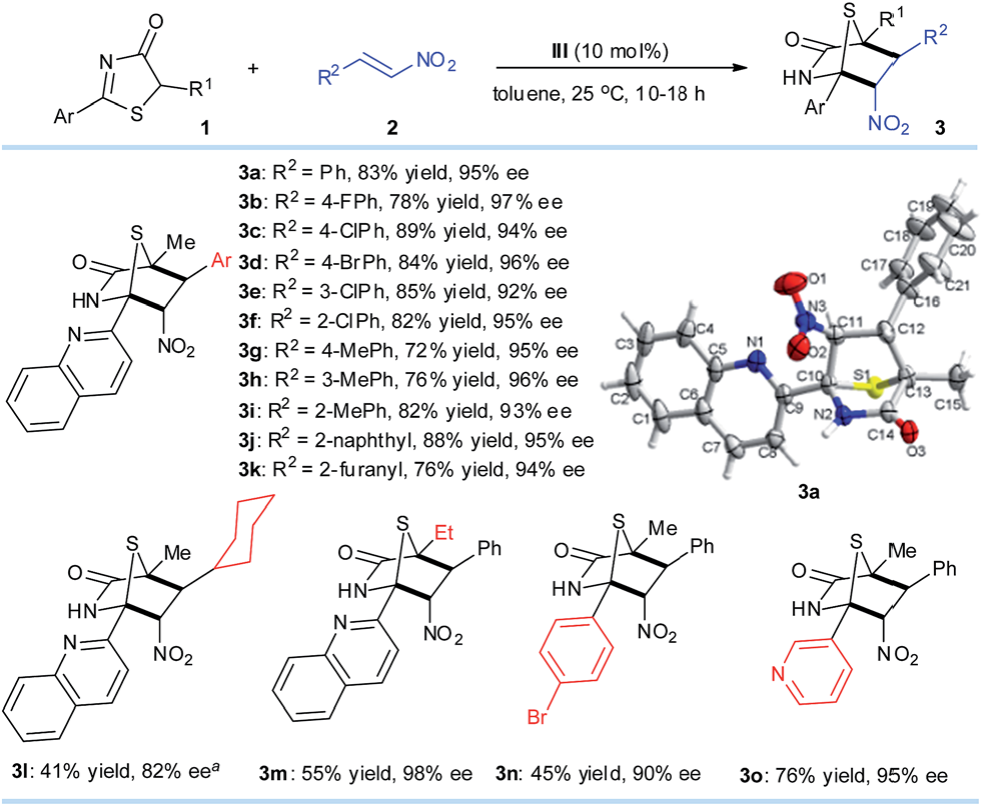
Reaction conditions: **1** (0.2 mmol), **2** (0.4 mmol), **III** (0.02 mmol), toluene (4.0 mL) at 25 °C. All drs are >20 : 1. ees were determined *via* chiral HPLC analysis. ^*a*^ 72 hours.

With the optimized reaction conditions, we examined the substrate scope of the DP–TAA **III**-catalyzed enantioselective [4 + 2] annulation between the 5*H*-thiazol-4-ones 1 and nitroalkenes **2** (Scheme 1). First with **1a** as the model 5*H*-thiazol-4-one substrate, various aryl bearing nitroalkenes **2**, were transformed to the corresponding [4 + 2] cyclo-adducts **3a–j** in moderate to excellent yields and superior ee (92–97%). Replacing the R^2^ substituent on **2** to a 2-furanyl group gave a moderate yield of 76% and high ee of 94%. By reacting **1a** with **2l**, a nitroolefin cyclohexyl derivative, lower yield and poorer reactivity was observed but the corresponding adduct **3l** was achieved with a good 82% ee and moderate chemoselectivity.^14^ Changing the R^1^ group on 5*H*-thiazol-4-one for an ethyl substituent also saw the yield of **3m** drop to 55%, possibly due to steric effects during the conjugate addition process. Altering the aryl group of **1** gave low to moderate yields of **3n** (45%) and **3o** (76%). The absolute configurations of the cycloaddition products were assigned on the basis of the X-ray crystal structural analysis of **3a**.^14^

### Mechanistic proposal

**Fig. 3:**
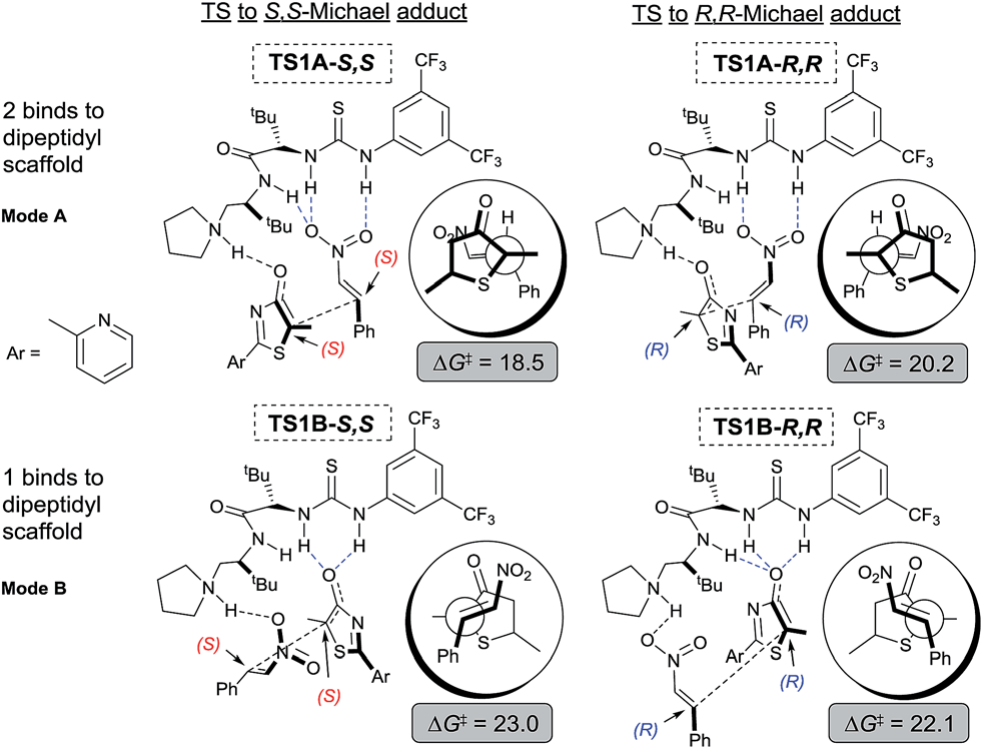
Computational modelling of the possible stereo-outcomes for the first Michael C–C bond formation process. Newman projection diagrams in circles for added perspective. All free energy barriers Δ*G*^‡^ and reaction free energies are kcal mol^–1^ relative to free starting materials **1**, **2** and **III**.

To gain mechanistic and stereochemical insights into this highly enantio- and chemo-selective reaction, we first modelled the reaction between 5*H*-thiazol-4-one **1**, nitroalkene **2**, and catalyst **III** with density functional theory (DFT) at a carefully chosen level of theory (for further theoretical details see the ESI†). Preliminary stereochemical-outcomes in generating the conjugate addition adducts were first considered based on the binding of either **1** (mode B) or **2** (mode A) to the main dipeptidyl scaffold (Fig. 3). Only the eclipsed conformation of the reacting substrates was modelled as our previous work has shown that π–π* orbital interactions directly stabilize the transition state by approximately 2.7 kcal mol^–1^.11*e* A total of four pathways leading to adducts with *S*,*S*- and *R*,*R*-configurations and two different binding modes are possible. Transition state (TS) structures were optimized and free energy barriers calculated for each of to the four pathways (Fig. 3). **TS1A-*S*,*S*** (Δ*G*^‡^ = 18.5 kcal mol^–1^) was revealed as the most stable TS followed by **TS1A-*R*,*R*** (20.2). Both **TS1B-*S*,*S*** (23.0) and **TS1B-*R*,*R*** (22.1) have overall higher activation barriers than **TS1A-*S*,*S*** by about 3–5 kcal mol^–1^. This suggests that the binding of nitroalkene **2** to the urea–thiourea skeleton is preferred due to the stronger oxyanion hole interaction between the positively charged ammonium group and the negative charge of **1**.

**Scheme 2:**
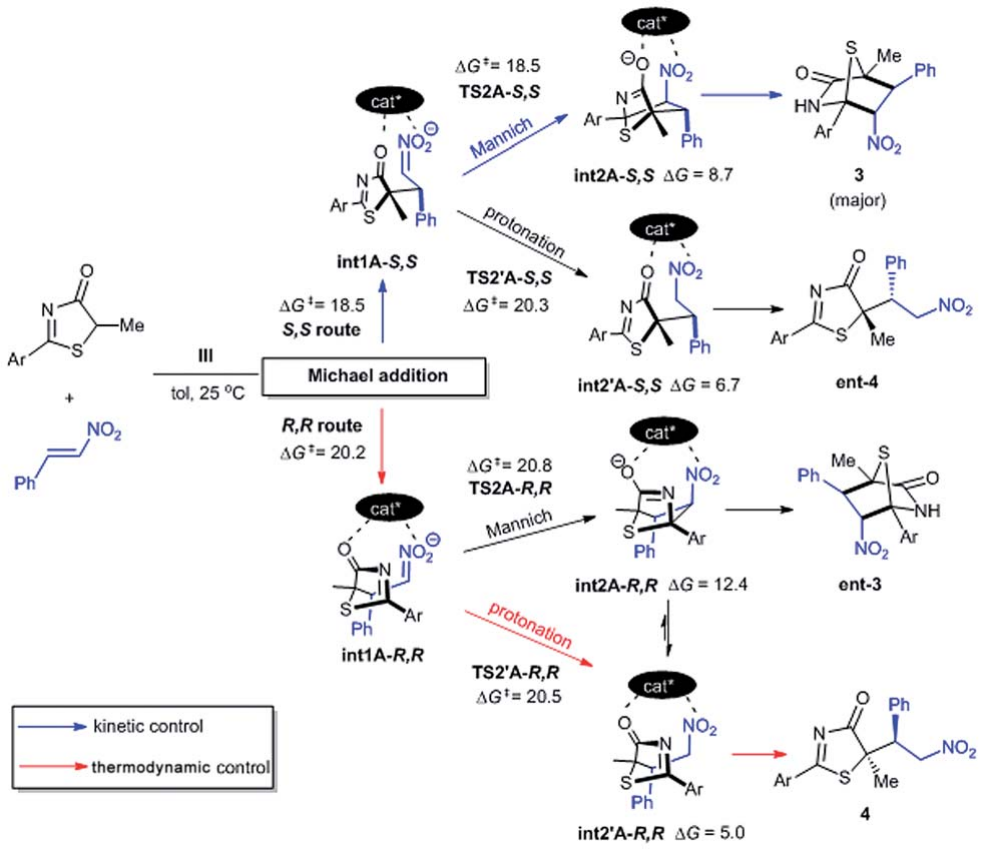
The *S*,*S* route affords the kinetically favorable [4 + 2] product **3** as the major product. Competition between the minor Michael products ***ent*-4** and **4** is thermodynamically controlled. All free energy barriers Δ*G*^‡^ and reaction free energies are kcal mol^–1^ relative to free starting materials **1**, **2** and **III**.

The free energy difference between **TS1A-*S*,*S*** and **TS1A-*R*,*R*** is ΔΔ*G*^‡^ = 1.7 kcal mol^–1^, which would correspond to a ∼6 : 1 *S*,*S* to *R*,*R*-configuration distribution ratio in the product. However, the first Michael addition **TS1** is not the rate determining step. Following the chemical transformation from the two lowest pathways **TS1A-*S*,*S*** and **TS1A-*R*,*R*** through the *S*,*S*- and *R*,*R*-routes respectively, further bifurcation from the Michael adducts **int1A** would lead to either the formal Mannich or protonation reaction pathways, processes with higher or equivalent activation free energies (Scheme 2). The Mannich reaction through the *S*,*S* pathway is the most kinetically dominant route, Δ*G*^‡^ = 18.5 kcal mol^–1^, and hence yields the majority of the [4 + 2] product **3** from **int2A-*S*,*S***, consistent with our experiments. The rest of the reaction channels through protonation or Mannich connect to the intermediates **int2′A*-S*,*S***, **int2′A*-R*,*R*** and **int2A*-R*,*R***, which have very close free energy barriers: Δ*G*^‡^ = 20.3, 20.5 and 20.8 kcal mol^–1^ respectively. The theoretical ee value is 96%, computed based on the activation free energy difference between **TS2A-*S*,*S*** and **TS2A-*R*,*R*** (ΔΔ*G*^‡^ = 2.3 kcal mol^–1^). This value is consistent with the experimental ee value of 95%. For product **4**, we rationalize that under conditions of thermodynamic control **int2′A*-R*,*R*** will most likely be favored as having the lowest Gibbs free energy of reaction (Scheme 2). Indeed as experimentally observed when the reaction temperature was lowered (see Table 1 entry 10) more ***ent-*4** (*i.e.*, **int2′A-*S*,*S***) is formed. This suggests that, when the thermodynamic effect is mitigated, enantioselectivity for the Michael-protonation product will be adversely affected.

**Fig. 4:**
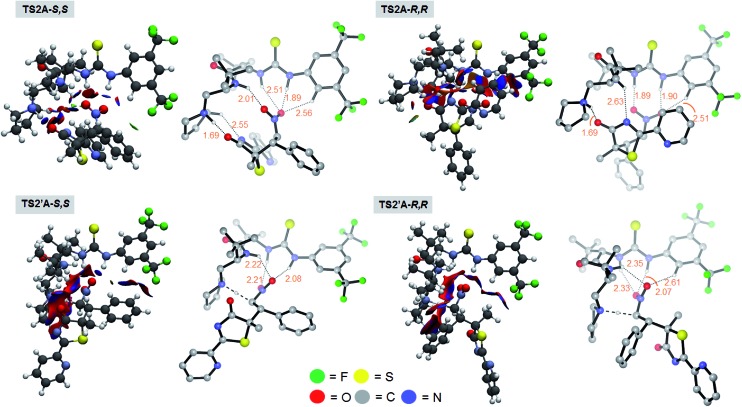
Non-covalent interaction isosurfaces of the optimized TS structures of the Mannich (**TS2A-*S*,*S*** and **TS2A-*R*,*R***) and the protonation processes (**TS2′A-*S*,*S*** and **TS2′A-*R*,*R***). Blue-green-red color scale from –0.4 < sign(*λ*_2_)*ρ*(*r*) > 0.4 au, where blue (positive) is favorable and red (negative) is an unfavorable interaction. Inset: corresponding TS optimized geometries with key H-bonding featured with dotted lines. Non-relevant C–H hydrogens were omitted for visual clarity. Values are bond distances in Å.

Chemically intuitive insights into the stereochemical preference for the rate determining *S*,*S* pathway formal Mannich process (**TS2A*-S*,*S***) were inferred from an examination of the non-covalent interactions (NCI) between the bound catalyst **III** and the substrates **1** and **2** (see Fig. 4). Thus, Mannich and protonation transition state structures, diverging from the *S*,*S* and *R*,*R* pathways were analyzed with the reduced density gradient based NCIplot method (see ESI†) to identify these critical NCI areas. These interactions could either be attractive or repulsive depending on the sign of the second density Hessian eigenvalue (sign(*λ*_2_), Fig. 4). Visual inspection of the NCI isosurfaces of **TS2A-*S*,*S*** shows a smaller degree of repulsive forces compared with **TS2A-*R*,*R***, **TS2′A-*S*,*S*** and **TS2′A-*R*,*R***. More importantly, critical H-bonding features between **TS2A-*S*,*S*** and the rest of the TSs are different. For instance the **TS2A-*S*,*S*** NCI isosurface reveals non-classical H-bonding between the catalyst-pyrrolidinium's activated C_α_-H group and the amide group of **1** (C_α_–H···N = 2.55 Å). The significance of this type of non-classical H-bonding interaction was highlighted by Canizzaro and Houk as playing a crucial role in stereoselective organocatalysis.^15^ Also, the involvement of the three amide N–H groups of the catalyst core in a tripodal H-bonding fashion would bind **2** in an optimal position for the subsequent C–C bond addition, facilitating the Mannich process. Thus, the chemical influence exerted by the dipeptide catalyst toward activating the substrates for [4 + 2] cyclization was achieved the way we had originally envisaged (Fig. 2).

### Reactivity with *trans*-4-oxo-4-arylbutenones and *trans*-4-oxo-4-arylbutenoates

**Scheme 3:**
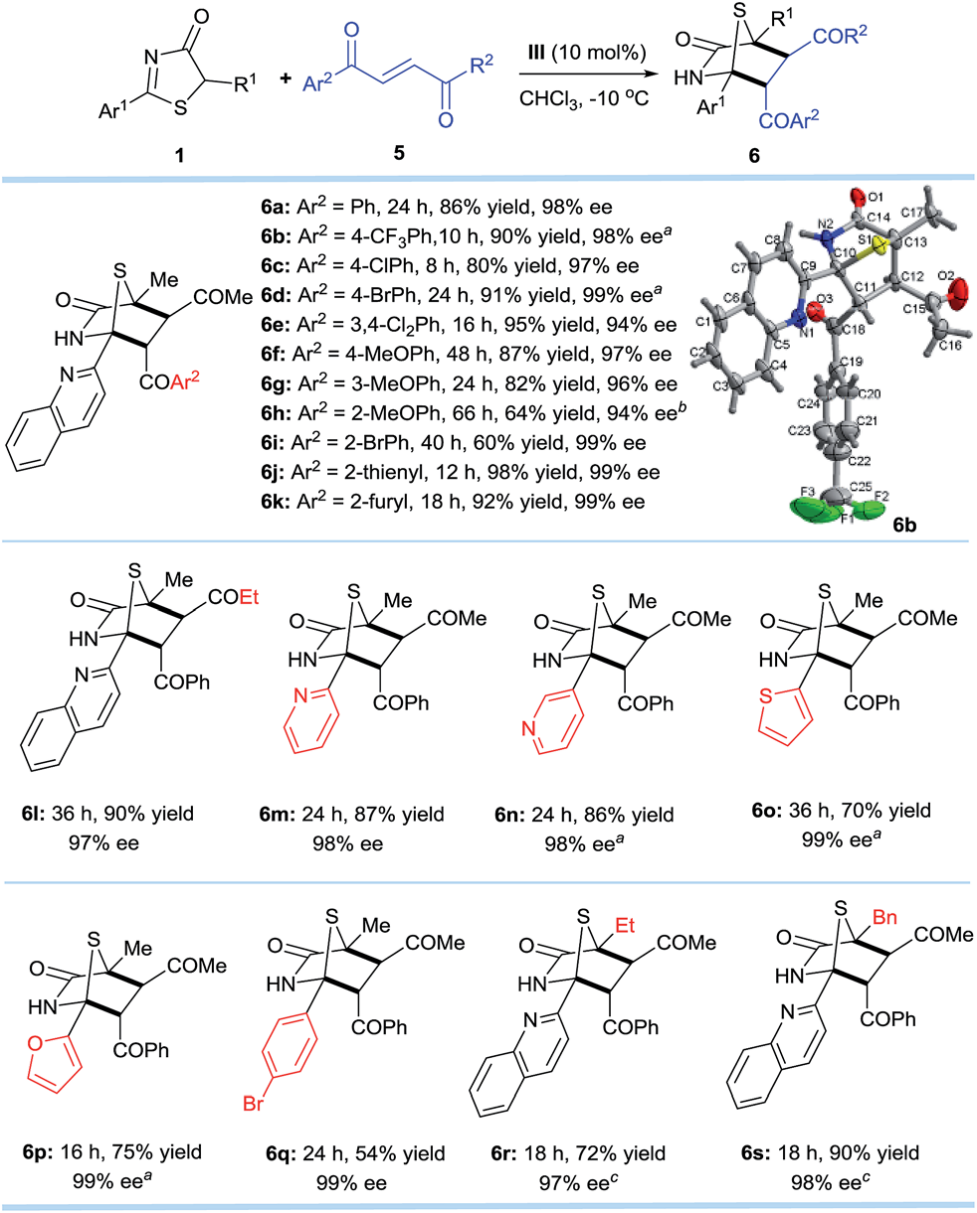
Reaction conditions: **1** (0.2 mmol), **5** (0.3 mmol), **III** (0.02 mmol), CHCl_3_ (4.0 mL) at –10 °C. All drs are >20 : 1. ees were determined *via* chiral phase high-performance liquid chromatography analysis. ^*a*^*T* = –30 °C. ^*b*^*T* = 20 °C. ^*c*^*T* = 25 °C.

Encouraged by the promising potential of the DP–TAA type catalysts for asymmetric [4 + 2] annulation, we shifted our focus to an alkene electrophile *trans*-4-oxo-4-phenylbutenone **5**,^16^ a new substrate for the asymmetric [4 + 2] cyclization (Scheme 3). The reactions were first performed between the 5*H*-thiazol-4-one substrate **1a** and the *trans*-4-oxo-4-arylbutenones **5** bearing various aryls, (hetero)aryls, and ethyl under slightly modified reaction conditions (in CHCl_3_ at –10 °C). The corresponding [4 + 2] cyclo-adducts **6a–l** were obtained in moderate to excellent yields and superior ee (94–99%). Replacing the 2-quinolyl Ar^1^ substituent of **1a** at the 2-position to other (hetero)aryl and aryl groups also gave products **6m–q** with excellent ee values (98–99%). When the methyl R^1^ group of **6a** was replaced by ethyl (**6r**) or benzyl (**6s**), the reaction was sluggish possibly due to steric effects, but satisfactory results were obtained when the reaction was carried out at 25 °C. The absolute configurations of the cycloaddition products were assigned on the basis of the X-ray crystal structural analysis of **6b**.^14^

**Scheme 4:**
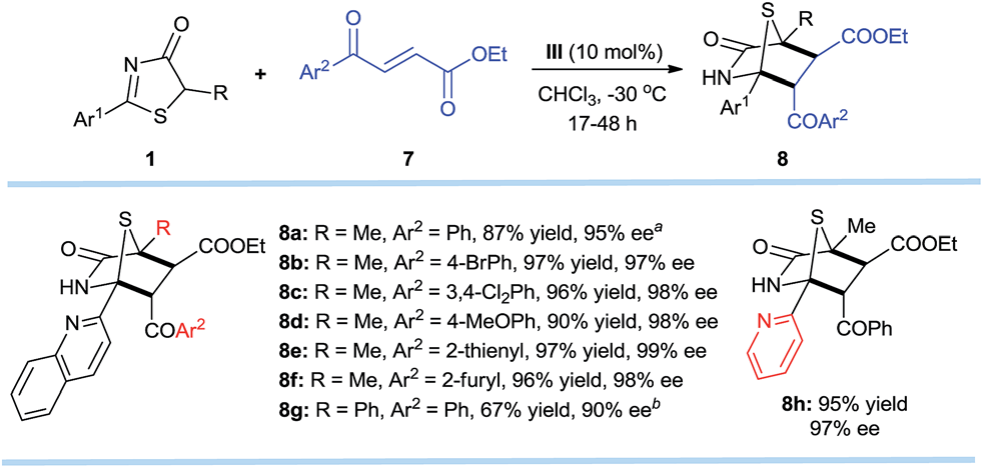
**1** (0.2 mmol), **7** (0.3 mmol), **III** (0.02 mmol), CHCl_3_ (4.0 mL) at –30 °C. All drs are >20 : 1. ee values were determined *via* chiral phase high-performance liquid chromatography analysis. ^*a*^*T* = –20 °C. ^*b*^ 0.04 mmol of **III**, 0.2 mmol of Li_3_PO_4_, *T* = 30 °C.

The established methodology was also successfully applied to *trans*-4-oxo-4-arylbutenoates10h,^16^,^17^ **7** that led to [4 + 2] annulation adducts **8a–h** in 67–97% yields and excellent enantioselectivities, unaffected by steric and electronic effects on the aromatic ring substituents of the *trans*-4-oxo-4-arylbutenoates (Scheme 4). It is noteworthy that both annulation adducts **6** and **8** are analogues of *Trypanosoma cruzi* dihydrofolate reductase inhibitors1*l* (Fig. 1a).

### Reactivity with methyleneindolinones

**Scheme 5:**
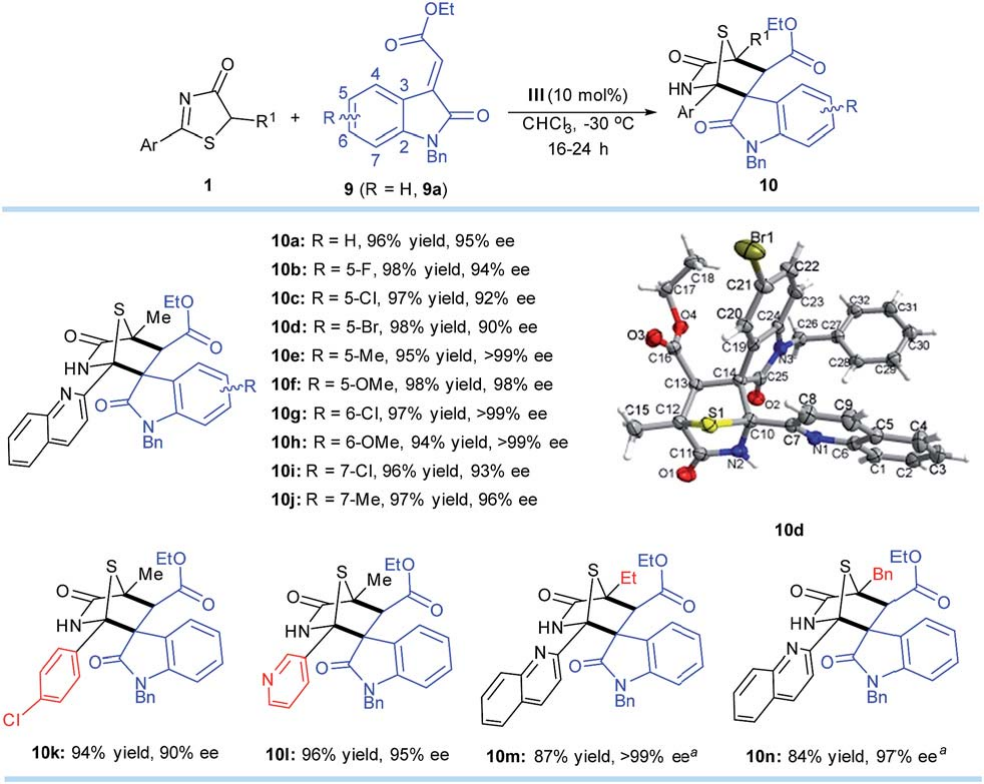
**1** (0.2 mmol), **9** (0.3 mmol), **III** (0.02 mmol), CHCl_3_ (4.0 mL) at –30 °C. All drs are >20 : 1. ees were determined *via* chiral phase high-performance liquid chromatography analysis. ^*a*^*T* = –20 °C.

Methyleneindolinones have been widely used as dipolarophiles in [2 + 3] cyclizations to build spirooxindoles with multiple stereocenters.^18^ However, few catalytic asymmetric strategies^19^ are suitable for the [4 + 2] process. Inspired by the success of [4 + 2] annulation from the DP–TAA catalysts, the reaction between 5*H*-thiazol-4-one substrate **1a** and methyleneindolinone **9a** was first evaluated under the established reaction conditions (10 mol% of catalyst **III** in CHCl_3_ at –30 °C). As expected, the 1,4-sulfur bridged piperidinonespirooxindole **10a**, which has three quaternary and one tertiary stereogenic centers, was obtained in 96% yield with 95% ee and >20 : 1 dr (Scheme 5).

A number of 5*H*-thiazol-4-ones and methyleneindolinones were next examined to determine the substrate generality and limitations of this strategy. Various adducts **10b–n** were attained in excellent yields (84–98%) and enantioselectivities (90–>99% ee). Moreover, only one diastereomer was observed as determined using ^1^H NMR spectroscopy of the crude reaction mixtures for each product. It is worth noting that these synthesized chiral compounds are known as drug candidates for antiproliferative and anticancer agents.^20^ The absolute configurations of the cycloaddition products were assigned on the basis of the X-ray crystal structural analysis of **10d**.^14^

The synthetic value of this novel catalyst in enantioselective synthesis was further explored for the targeted synthesis of a hexa-substituted tetrahydrothiophene **11**, which features four contiguous chiral centers including two heteroquaternary stereogenic centers (eqn (1)). Such a tetrahydrothiophene is an important structural scaffold existing in several molecules displaying interesting biological activities.^21^1
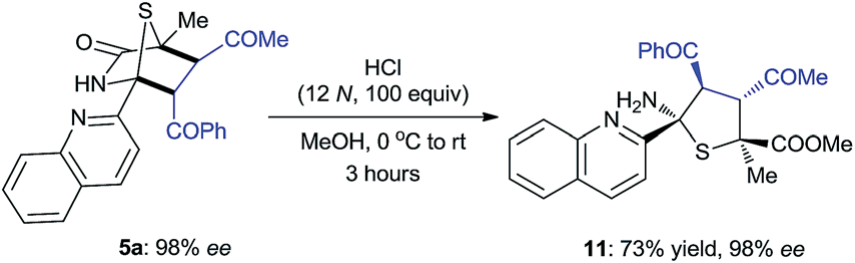

2
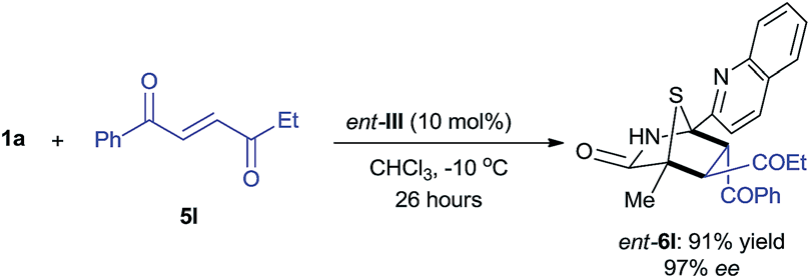



The reaction between 5*H*-thiazol-4-one **1a** and *trans*-4-oxo-4-phenylbutenone **5l** was conducted with 10 mol% of d-*tert*-leucine-based ***ent*-III** catalyst in chloroform at –10 °C (eqn (2)). The enantiomer of **6l** (***ent*-6l**) was successfully obtained in 91% yield with 97% ee after 26 hours, thus demonstrating the potential of these dipeptide-based catalysts for enantiodivergent synthesis.

## Conclusions

In summary, we have developed a new family of dipeptide-based multifunctional Brønsted base catalysts that are DP–TAAs and DP–UAAs. By employing DP–TAA **III** derived from l-*tert*-leucine as the catalyst, the asymmetric reaction of 5*H*-thiazol-4-ones with a broad range of electron-deficient alkenes including nitroolefins, *trans*-4-oxo-4-arylbutenones, *trans*-4-oxo-4-arylbutenoates and methyleneindolinones, unprecedented [4 + 2] annulation with high chemo- and enantio-selectivities (up to 98% yield and >99% ee) was realized. The current method furnishes an expedient approach to chiral 1,4-sulfur bridged piperidinones and their derivatives, such as hexa-substituted tetrahydrothiophenes, which feature two or even three quaternary carbon stereocenters and are potentially important for medicinal chemistry research. Furthermore, mechanistic insights gained from computational modelling studies of **III**, **1** and nitroolefin **2** provide valuable information on the stereo- and chemo-selection origins. Finally, given the highly modular approach in synthesizing the catalyst by varying the amino acid chiral backbones and appending various Brønsted bases, we anticipate that such dipeptide catalysts will find prolific application in more challenging organic transformations.

## Supplementary Material

Supplementary information

Crystal structure data
