# Supplementary material for: Asymmetric [4 + 2] annulation of 5H-thiazol-4-ones with a chiral dipeptide-based Brønsted base catalyst
Source: Chem Sci. 2016 Jun 9;7(9):6060–7. doi: 10.1039/c6sc02039a (PMC6022207; doi:10.1039/c6sc02039a)

Electronic Supplementary Material (ESI) for Chemical Science.
This journal is © The Royal Society of Chemistry 2016

Asymmetric [4+2] Annulation of 5*H*-Thiazol-4-Ones with a Chiral Dipeptide-Based Brønsted Base Catalyst

Bo Zhu, Shuai Qiu, Jiangtao Li, Michelle L. Coote,

Richmond Lee* and Zhiyong Jiang*

Supporting Information

Table of Contents

1. General information
S3-4
2. Synthesis of dipeptide-based **III**
S5-6
3. Optimization of reaction conditions for nitroalkene **2a** as acceptor
S7
4. Optimization of reaction conditions for 4-oxo-4-phenylbutenone **5a** as acceptor
S8
5. Optimization of reaction conditions for methyleneindolinones **9** as acceptor
S9
6. General experimental procedure for [4+2] annulation of 5*H*-thiazol-4-ones **1** with nitroalkenes **2**
S10
7. General experimental procedure for [4+2] annulation of 5*H*-thiazol-4-ones **1** with 4-oxo-4-arylbutenones **5**
S11
8. General experimental procedure for the [4+2] annulation of 5*H*-thiazol-4-ones **1** with 4-oxo-4arylbutenoates **7**
S12
9. General experimental procedure for the [4+2] annulation of 5*H*-thiazol-4-ones **1** with methyleneindolinones **9**
S13
10. Chemoselective results
S14-15
11. Exploration towards reactions between 5*H*-oxazol-4-ones and alkenes
S16
12. Experimental procedure for **5a** to **11**
S17
13. Characterization of adducts
S18-50
14. Determination of the absolute configuration
S51-52
15. Computational method
S53-74
16. Copies of NMR spectra
S75-133

1. General information

General procedures and methods

Experiments involving moisture and/or air sensitive components were performed under a positive pressure of nitrogen in oven-dried glassware equipped with a rubber septum inlet. Dried solvents and liquid reagents were transferred by oven-dried syringes or hypodermic syringe cooled to ambient temperature in a desiccator. Reactions mixtures were stirred in 4 mL sample vial with Teflon-coated magnetic stirring bars unless otherwise stated. Moisture in non-volatile reagents/compounds was removed in high *vacuo* by means of an oil pump and subsequent purging with nitrogen. Solvents were removed in vacuo under ~30 mmHg and heated with a water bath at 30–40 °C using rotary evaporator with aspirator. The condenser was cooled with running water at 0 °C.

All experiments were monitored by analytical thin layer chromatography (TLC). TLC was performed on pre-coated plates, 60 F_{254}. After elution, plate was visualized under UV illumination at 254 nm for UV active material. Further visualization was achieved by staining $KMnO_4$, ceric molybdate, or anisaldehyde solution. For those using the aqueous stains, the TLC plates were heated on a hot plate.

Columns for flash chromatography (FC) contained silica gel 200–300 mesh. Columns were packed as slurry of silica gel in petroleum ether and equilibrated solution using the appropriate solvent system. The elution was assisted by applying pressure of about 2 atm with an air pump.

Instrumentations

Proton nuclear magnetic resonance (^{1}H NMR), carbon NMR (^{13}C NMR), and fluorous (^{19}F NMR) spectra were recorded in $CDCl_3$ otherwise stated. ^{1}H (300 MHz) and ^{13}C (75 MHz) were performed on a 300MHz spectrometer. Chemical shifts are reported in parts per million (ppm), using the residual solvent signal as an internal standard: $CDCl_3$ (^{1}H NMR: δ 7.26, singlet; ^{13}C NMR: δ 77.0, triplet). Multiplicities were given as: *s* (singlet), *d* (doublet), *t* (triplet), *q* (quartet), *quintet*, *m* (multiplets), *dd* (doublet of doublets), *dt* (doublet of triplets), and *br* (broad). Coupling constants (*J*) were recorded in Hertz (Hz). The number of proton atoms (*n*) for a given resonance was indicated by *n*H. The number of carbon atoms (*n*) for a given resonance was indicated by *n*C. HRMS was reported in units of mass of charge ratio (m/z). Mass samples were dissolved in DCM and MeOH (HPLC Grade) unless otherwise stated. Optical rotations were recorded on a polarimeter with a sodium lamp of wavelength 589 nm and reported as follows; $[\alpha]_{\lambda}^{T°C}$ (c = g/100 mL, solvent). Melting points were determined on a melting point apparatus.

Enantiomeric excesses were determined by chiral High Performance Liquid Chromatography (HPLC) analysis. UV detection was monitored at 254 nm, 230 nm and 210 nm at the same time. HPLC samples were dissolved in HPLC grade isopropanol (IPA) unless otherwise stated.

Materials

All commercial reagents were purchased with the highest purity grade. They were used without further purification unless specified. All solvents used, mainly petroleum ether (PE) and ethyl acetate

(EtOAc), were distilled. Anhydrous CH_2Cl_2, $CHCl_3$ and DCE were freshly distilled from CaH_2 and stored under N_2 atmosphere. THF, Et_2O and toluene were freshly distilled from sodium/benzophenone before use. All compounds synthesized were stored in a −20 °C freezer and light-sensitive compounds were protected with aluminium foil.

2. Synthesis of dipeptide-based III

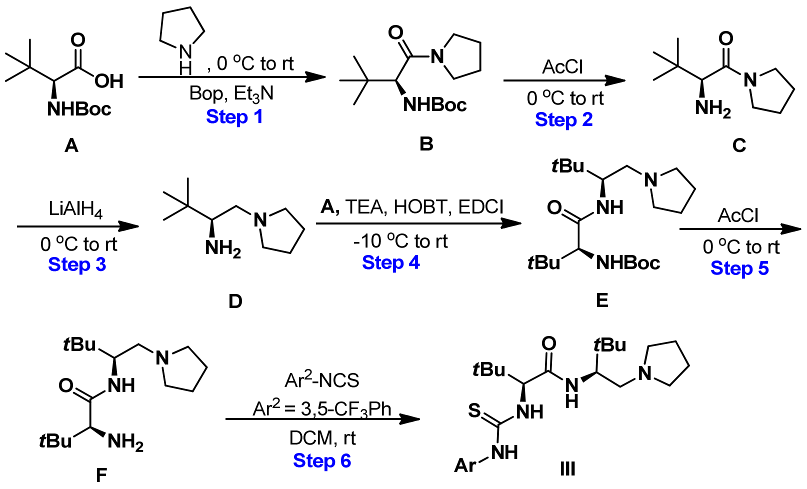

Step 1: To a solution of crude Boc-protected amino acid **A** (2.31 g, 10 mmol) in THF (15 mL), Et_3N (2.78 mL, 20 mmol) and BOP (5.41 g, 12 mmol) was added at 0 °C. Pyridine (1.16 mL, 13 mmol) was injected after the mixture was stirred for 0.5 hour at 0 °C. Then the reaction mixture was stirred at room temperature for another 8 hours (monitored by TLC). Solvent was removed under reduced pressure and **B** was obtained by flash chromatography (*silica gel*, Hexane/ethyl acetate 10:1–3:1), as a white solid, 2.32 g, 81.6% yield.

Step 2: AcCl (4.6 mL, 65.6 mmol) was added to a solution of **B** (2.32 g, 8.2 mmol) in MeOH (20 mL) 0 °C. The reaction mixture was stirred at room temperature for 8 hours (monitored by TLC). Solvent was removed under reduced pressure and solid **C** (1.51 g, 100%) was obtained for the next step without any purification.

Step 3: $LiAlH_4$ (1.25 g, 32.8 mmol) was added into a solution of **C** (1.51 g, 8.2 mmol) in THF (20 mL) slowly. The mixture was stirred at 0 ºC for about 5.0 minutes then warmed to room temperature and stirred overnight. Subsequently, the reaction was carefully quenched by NaOH aqueous solution (4.0 *N,* 2 mL). The solids were filtered off and washed by THF, and the filtrate was dried over Na_2SO_4. Solvent was removed under reduced pressure and **D** was obtained as yellow oil in 86.5% yield (1.2 g).

Step 4: To a solution of diamine **D** (0.75 g 4.4 mmol) in THF (25 mL) were added TEA (1.84 ml, 13.2 mmol), Boc-protected amino acid **A** (1.2 g, 5.3 mmol) and HOBt (0.89 g, 6.6 mmol) at −10 °C for 15 min. Then EDCI (1.3 g, 6.6 mmol) was added the mixture, and the mixture was stirred overnight at room temperature. The reaction mixture was poured into water and extracted with AcOEt. The AcOEt layer was washed with water and brine and dried over Na_2SO_4. Filtration and concentration in vacuo and purification by *silica gel* flash column chromatography (AcOEt/n-hexane = 1/5) gave 1.28 g (76%) of **E** as a white solid.

Step 5 AcCl (1.89 mL, 26.7mmol) was added to a solution of **E** (1.28 g, 3.34 mmol) in MeOH (20 mL) 0 °C. The reaction mixture was stirred at room temperature for 8 hours (monitored by TLC). Solvent was removed under reduced pressure and solid **F** (0.95 g, 100%) was obtained for the next step without any purification.

Step 6 (Final step)**:** To a solution of **F** (308.6 mg, 1.09 mmol) in DCM (5 mL) was added 3,5-di(trifluoromethyl)phenyl isothiocyanate (295.2 mg, 1.09 mmol). The resulting solution was stirred for 5 min at 0 ^{o}C. The reaction was concentrated *in vacuo* and the residue was purified by *silica gel* chromatography (*silica gel*, DCM/MeOH 100:1–30:1) to afford product **III** as a white solid in 85% yield (513.1 mg).

3. Optimization of reaction conditions for nitroalkene 2a as acceptor

Table 1 Asymmetric [4+2] cyclization catalyzed by amino acid-derived tertiary amine−(thio)ureas[a]

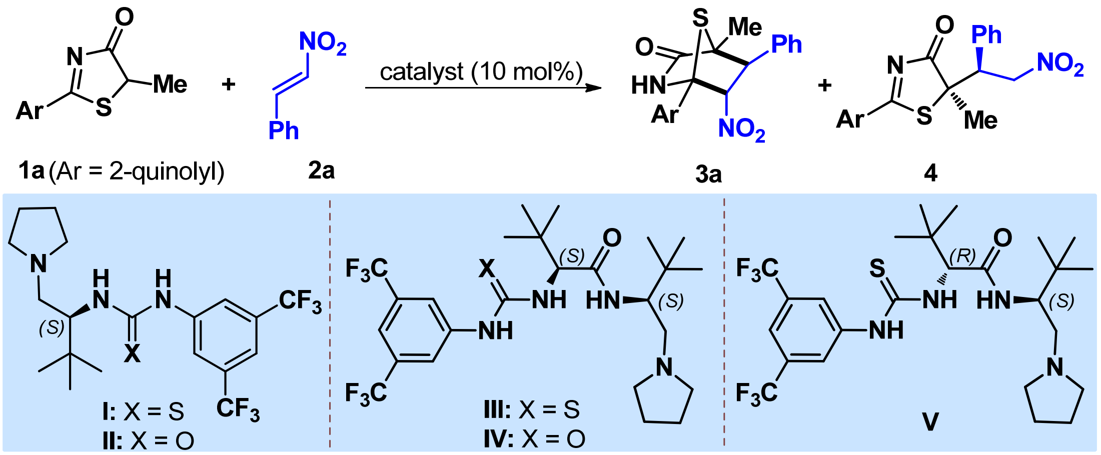

Entry	Catalyst	Solvent	*T* (°C)	*t* (h)	**3a**:**4**	**3a** Yield (%)[b]	**3a** e.e. (%)[c]	**3a** d.r.[d]	**4** Yield (%)[b]	**4** e.e. (%)[c]	**4** d.r.[d]
1	Et_3N	CH_2Cl_2	25	18	trace	trace	n.a.	--	36	n.a.	n.a.
2	**I**	CH_2Cl_2	25	12	1:1	42	0	15:1	45	64	1:1
3	**II**	CH_2Cl_2	25	12	3:1	62	7	9:1	20	3	19:1
4	**III**	CH_2Cl_2	25	12	4:1	73	86	19:1	13	88	12:1
5	**IV**	CH_2Cl_2	25	12	4:1	68	87	8:1	18	87	9:1
6	**III**	THF	25	16	trace	trace	n.a.	n.a.	trace	n.a.	n.a.
7	**III**	toluene	25	16	5:1	84	94	>19:11	8	88	12:1
8	**III**	Et_2O	25	16	4:1	70	92	>19:1	11	96	>19:1
9	**III**	$CHCl_3$	25	16	4.5:1	72	83	>19:1	9	85	>19:1
10	**III**	toluene	10	36	4:1	56	95	>19:1	10	31	3:1
11	**III**	toluene	30	18	4.5:1	78	92	19:1	12	75	19:1
12[e]	**III**	toluene	25	18	5:1	83	95	>19:1	11	88	12:1
13[e]	**V**	toluene	25	18	2.5:1	68	−75	>19:1	17	13	>19:1

[a]The reaction was performed using **1a** (0.05 mmol) and **2a** (0.075 mmol) in 1.0 mL of solvent.

[b]Isolated yield based on **1a** after column chromatography.

[c]Enantiomeric ratio of product **3a** was determined via chiral phase HPLC analysis.

[d]Determined by crude ^1H NMR analysis.

[e]The reaction was performed using **1a** (0.2 mmol) and **2a** (0.4 mmol) in 4.0 mL of solvent.

4. Optimization of reaction conditions for 4-oxo-4-phenylbutenone 5a as acceptor

Table 2 Condition optimization[a]

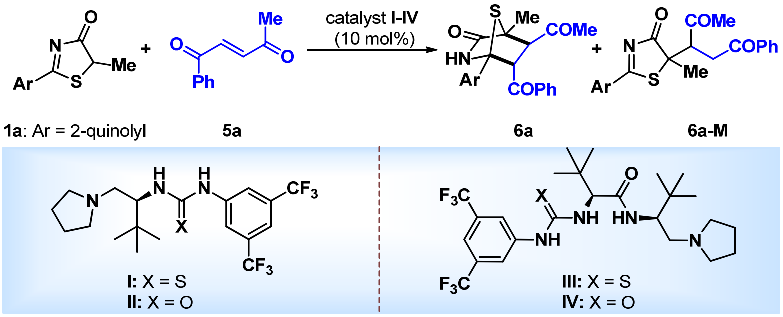

Entry	Catalyst	Solvent	T (°C)	t (h)	6a Yield (%)[b]	6a e.e. (%)[c]	6a-M Yield (%)[b]	6a-M e.e. (%)[c]	6a-M d.r.[d]
1	Et_3N	CH_2Cl_2	25	96	11	n.a.	72	n.a.	10:1
2	**I**	CH_2Cl_2	25	3	27	62	57	62	>19:1
3	**II**	CH_2Cl_2	25	3	44	54	46	37	>19:1
4	**III**	CH_2Cl_2	25	2.5	85	87	12	32	>19:1
5	**IV**	CH_2Cl_2	25	3	78	68	18	58	19:1
6	**III**	toluene	25	7	54	78	20	73	>19:1
7	**III**	Et_2O	25	7	13	71	5	72	>19:1
8	**III**	$CHCl_3$	25	3.5	82	91	10	50	13:1
9	**III**	DCE	25	5	80	88	13	26/69	11:1
10	**III**	$CHCl_3$	0	18	80	96	17	60/62	13:1
11	**III**	$CHCl_3$	−10	24	86	98	9	69/53	19:1

[a]Reaction conditions: **1a** (0.05 mmol), **5a** (0.075 mmol), solvent (1.0 mL). For product **6a**, all *dr*s are >20:1 determined by crude ^{1}H NMR.

[b]Yield of isolated product.

[c]*Ee* was determined via chiral phase HPLC analysis.

[d]Determined by ^{1}H NMR analysis.

5. Optimization of reaction conditions for methyleneindolinones 9 as acceptor

Table 3 Condition optimization[a]

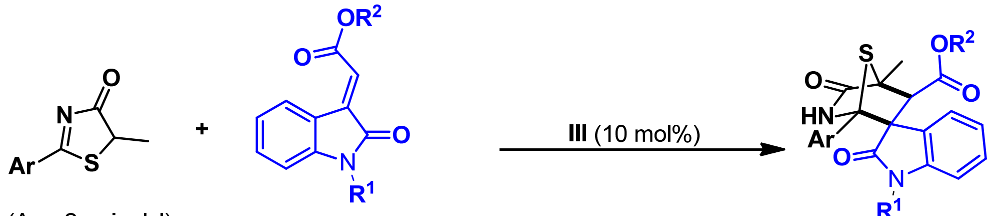

1a (Ar = 2-quinolyl) **9** **10**

Entry	Solvent	R[1]	R[2]	*T* (°C)	*t* (h)	**10**	
						Yield (%)[b]	*ee* (%)[c]
1	DCE	Me	Et	25	5	85	73
2	$CHCl_3$	Me	Et	25	5	91	75
3	$CHCl_3$	Me	Et	−10	10	86	85
4	$CHCl_3$	Me	*t*Bu	−10	12	84	80
5	$CHCl_3$	Boc	Et	−10	12	trace	n.a.
6	$CHCl_3$	Bn	Et	−10	12	90	94
7	$CHCl_3$	Bn	Et	−30	24	96	95

[a]Reaction conditions: **1a** (0.05 mmol), **9** (0.075 mmol), solvent (1.0 mL). For product **10**, all *dr*s are >20:1 determined by crude ^{1}H NMR.

[b]Yield of isolated product.

[c]*Ee* was determined via chiral phase HPLC analysis.

6. General experimental procedure for the [4+2] annulation of 5*H*-thiazol-4-ones 1 with nitroalkenes 2

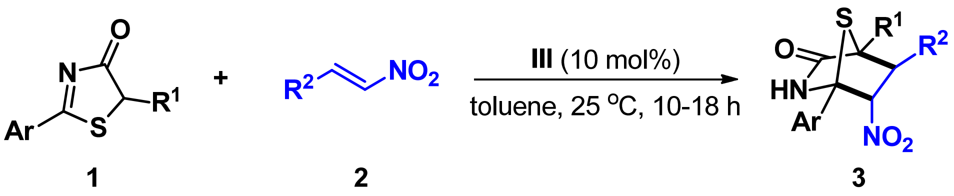

Nitroalkene **2** (0.4 mmol, 2.0 equiv) and catalyst **III** (11.5 mg, 0.02 mmol, 0.1 equiv) were dissolved in toluene (4.0 mL) and stirred at 25 °C for 15 min. Then 5*H*-thiazol-4-one **1** (0.2 mmol, 1.0 equiv) was added. The reaction mixture was stirred at 25 °C and monitored by TLC. Upon complete consumption of 5*H*-thiazol-4-one **1**, the reaction mixture was directly loaded onto a short *silica gel* column, followed by gradient elution with CH_2Cl_2/MeOH (800/1–300/1). Removing the solvent *in vacuo*, afforded products **3a-n**.

7. General experimental procedure for [4+2] annulation of 5*H*-thiazol-4-ones 1 with 4-oxo-4-arylbutenones 5

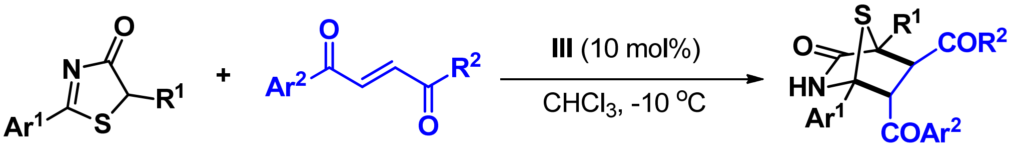

1 **5** **6**

4-Oxo-4-arylbutenone **5** (0.3 mmol, 1.5 equiv) and catalyst **III** (11.5 mg, 0.02 mmol, 0.1 equiv) were dissolved in $CHCl_3$ (4.0 mL) and stirred at −10 °C for 15 min. Then 5*H*-thiazol-4-one **1** (0.2 mmol, 1.0 equiv) was added. The reaction mixture was stirred at −10 °C and monitored by TLC. Upon complete consumption of 5*H*-thiazol-4-one **1**, the reaction mixture was directly loaded onto a short *silica gel* column, followed by gradient elution with CH_2Cl_2/MeOH (800/1–200/1). Removing the solvent *in vacuo*, afforded products **6a-s**.

8. General experimental procedure for [4+2] annulation of 5*H*-thiazol-4-ones 1 with 4-oxo-4-arylbutenoates 7

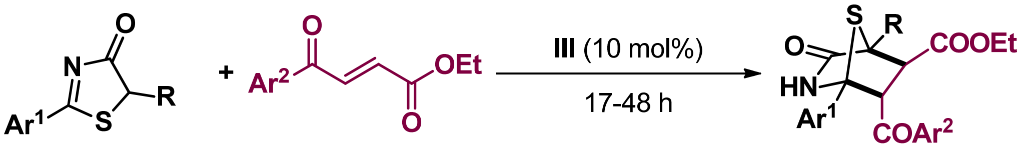

1 7 8

(R = Me): 4-Oxo-4-arylbutenoate **7** (0.3 mmol, 1.5 equiv) and catalyst **III** (11.5 mg, 0.02 mmol, 0.1 equiv) were dissolved in $CHCl_3$ (4.0 mL) and stirred at −30 °C for 15 min. Then 5*H*-thiazol-4-one **1** (0.2 mmol, 1.0 equiv) was added. The reaction mixture was stirred at −30 °C and monitored by TLC. Upon complete consumption of 5*H*-thiazol-4-one **1**, the reaction mixture was directly loaded onto a short *silica gel* column, followed by gradient elution with CH_2Cl_2/MeOH (800/1–200/1). Removing the solvent *in vacuo*, afforded products **8a-f** and **8h**.

(R = Ph): 4-Oxo-4-arylbutenoate **7** (0.3 mmol, 1.5 equiv), Li_3PO_4 (0.2 mmol, 1.0 equiv) and catalyst **III** (22.2 mg, 0.04 mmol, 0.2 equiv) were dissolved in $CHCl_3$ (4.0 mL) and stirred at 30 °C for 15 min. Then 5*H*-thiazol-4-one **1** (0.2 mmol, 1.0 equiv) was added. The reaction mixture was stirred at 30 °C and monitored by TLC. Upon complete consumption of 5*H*-thiazol-4-one **1**, the reaction mixture was directly loaded onto a short *silica gel* column, followed by gradient elution with CH_2Cl_2/MeOH (800/1–200/1). Removing the solvent *in vacuo*, afforded products **8g**.

9. General experimental procedure for [4+2] annulation of 5*H*-thiazol-4-ones 1 with methyleneindolinones 9

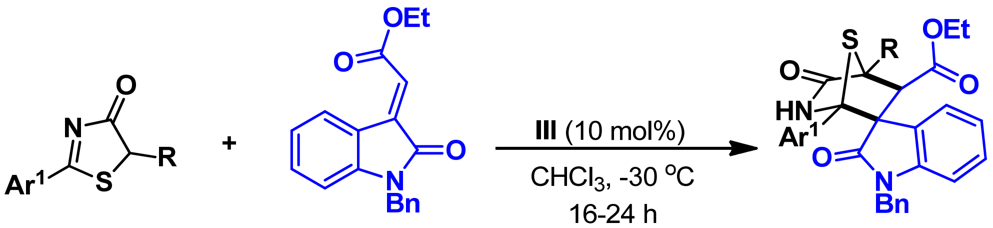

1 **9** **10**

Methyleneindolinones **9** (0.3 mmol, 1.5 equiv) and catalyst **III** (11.5 mg, 0.02 mmol, 0.1 equiv) were dissolved in $CHCl_3$ (4.0 mL) and stirred at −30 °C for 15 min. Then 5*H*-thiazol-4-one **1** (0.2 mmol, 1.0 equiv) was added. The reaction mixture was stirred at −30 °C and monitored by TLC. Upon complete consumption of 5*H*-thiazol-4-one **1**, the reaction mixture was directly loaded onto a short *silica gel* column, followed by gradient elution with CH_2Cl_2/MeOH (800/1–200/1). Removing the solvent *in vacuo*, afforded products **10a-n**.

10. Chemoselective results

Table 4 Reactions between **1** and **2** to afford **3** and **4**[a]

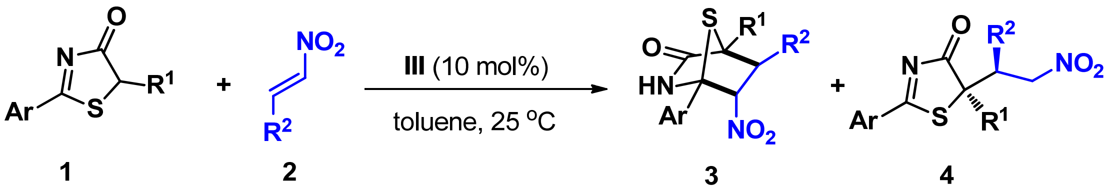

Entry	Ar	R^1	R^2	*t* (h)	3a Yield (%)[b]	4 Yield (%)[b]
1	2-quinolyl	Me	Ph	15	83	11
2	2-quinolyl	Me	4-FPh	12	78	16
3	2-quinolyl	Me	4-ClPh	14	89	6
4	2-quinolyl	Me	4-BrPh	13	84	8
5	2-quinolyl	Me	3-ClPh	15	85	9
6	2-quinolyl	Me	2-ClPh	18	82	12
7	2-quinolyl	Me	4-MePh	10	72	20
8	2-quinolyl	Me	3-MePh	13	76	18
9	2-quinolyl	Me	2-MePh	13	82	16
10	2-quinolyl	Me	2-naphthyl	16	88	8
11	2-quinolyl	Me	2-furyl	16	76	18
12	2-quinolyl	Me	cyclohexyl	72	41	51
13	2-quinolyl	Et	Ph	14	55	35
14	4-BrPh	Me	Ph	14	45	52
15	3-pyridyl	Me	Ph	10	76	19

[a]0.2 mmol scale. [b] Isolated yields.

Table 5 Reactions between **1** and **5** to afford **6** and **6-M**[a]

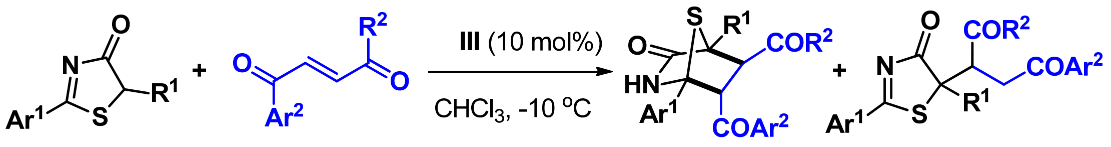

1 **5** **6** **6-M**

entry	Ar^1	R^1	Ar^2	R^2	*t* (h)	**6** yield (%)[b]	**6-M** yield (%)[b]
1	2-quinolyl	Me	Ph	Me	24	86	8
2	2-quinolyl	Me	4-CF_3Ph	Me	10	90	trace
3	2-quinolyl	Me	4-ClPh	Me	8	80	11
4	2-quinolyl	Me	4-BrPh	Me	24	91	trace
5	2-quinolyl	Me	3,4-Cl_2Ph	Me	16	95	trace
6	2-quinolyl	Me	4-MeOPh	Me	48	87	6
7	2-quinolyl	Me	3-MeOPh	Me	24	82	9
8	2-quinolyl	Me	2-MeOPh	Me	66	64	25
9	2-quinolyl	Me	2-BrPh	Me	40	60	28
10	2-quinolyl	Me	2-thienyl	Me	12	98	0
11	2-quinolyl	Me	2-furyl	Me	18	92	trace
12	2-quinolyl	Me	Ph	Et	36	90	5
13	2-pyridyl	Et	Ph	Me	24	87	7
14	3-pyridyl	Me	Ph	Me	24	86	10
15	2-thienyl	Me	Ph	Me	36	70	15
16	2-furyl	Me	Ph	Me	16	75	13
17	4-BrPh	Me	Ph	Me	24	54	42
18	2-quinolyl	Et	Ph	Me	18	72	10
19	2-quinolyl	Bn	Ph	Me	18	90	trace

[a]0.2 mmol scale. [b] Isolated yields.

11. Exploration towards reactions between 5*H*-oxazol-4-ones and alkenes

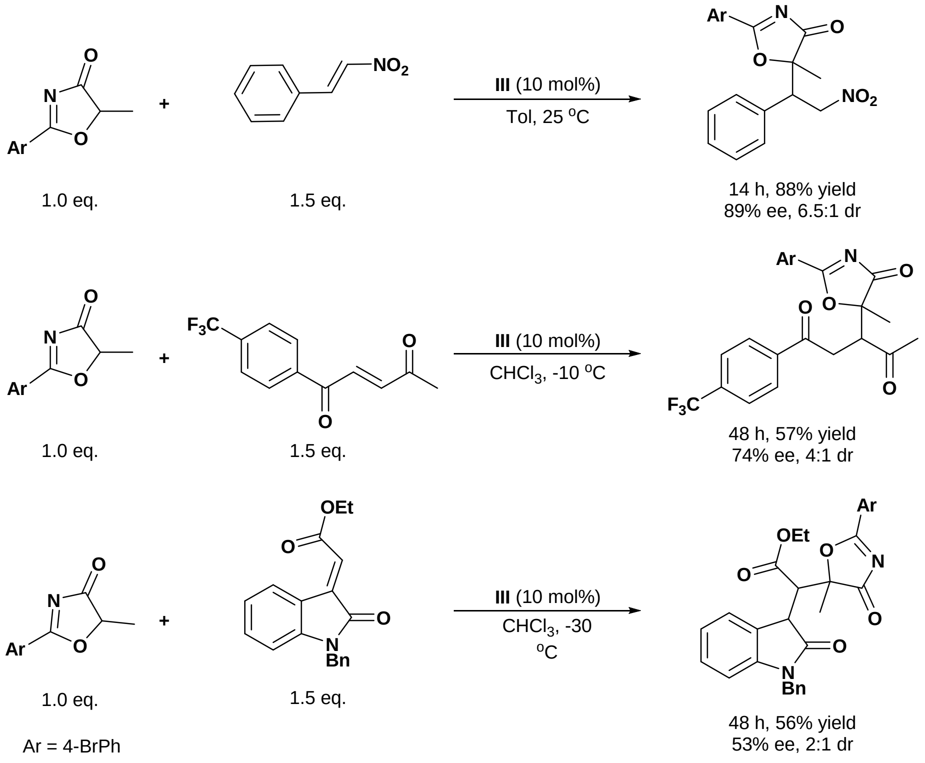

The results indicated that the reactions of 5*H*-oxazol-4-one with a series of activated alkenes under the established reaction conditions only presented conjugate addition adducts, and the corresponding annulation adducts were not observed yet. The moderate yields for 4-oxo-4-arylbutenone and methyleneindolinone as the substrates are produced by the instability of 5*H*-oxazol-4-one in the reactions.

12. Experimental procedure for 5a to 11

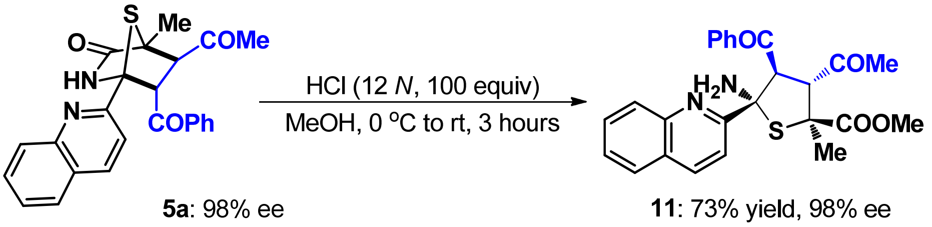

5a: 98% ee

11: 73% yield, 98% ee

The [4+2] annulation adduct **5a** (41.6 mg, 0.1 mmol, 1.0 equiv) was dissolved in MeOH (1.0 mL) and stirred at 0 °C. Then HCl (12 *N*, 0.834 mL, 10.0 mmol, 100.0 equiv) was added. The reaction mixture was stirred at room temperature until the reaction completed (3.0 hours). Evaporation of methanol followed by drying under high vacuo and neutralized by $NaHCO_3$ (aq.) until pH > 7. The mixture was then extracted with EtOAc (3.0 mL x 3), washed with brine, and dried over Na_2SO_4. Concentration was loaded onto a short *silica gel* column, followed by gradient elution with PE/EA (20/1–2/1). Removing the solvent *in vacuo*, afforded products gave the **11** (32.7 mg, 73%).

13. Characterization of adducts

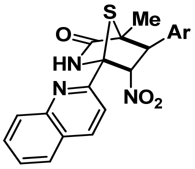

3a: Ar = Ph

White solid, Mp 165.3–166.5 °C; 83% yield; 95% *ee*; $[\alpha]_D^{26}$ –143.0 (*c* 1.00, $CHCl_3$); ^1H NMR (300 MHz, $CDCl_3$) δ 8.34 (d, *J* = 8.5 Hz, 1H), 8.07 (d, *J* = 8.5 Hz, 1H), 7.90–7.87 (m, 2H), 7.81–7.73 (m, 2H), 7.68–7.60 (m, 3H), 7.50–7.44 (m, 3H), 6.27 (d, *J* = 4.8 Hz, 1H), 4.04 (d, *J* = 4.8 Hz, 1H), 1.29 (s, 3H); ^{13}C NMR (75 MHz, $CDCl_3$) δ 176.8, 150.1, 147.0, 138.3, 136.0, 130.6, 129.6, 129.1, 129.0, 128.6, 128.0, 127.9, 127.6, 117.4, 98.6, 76.7, 67.1, 54.8, 13.8; HRMS (ESI) m/z 392.1074 (M+H$^+$), calc. for $C_{21}H_{18}N_3O_3S$ 392.1069.

The *ee* was determined by HPLC analysis. CHIRALPAK IC (4.6 mm i.d. x 250 mm); Hexane/2-propanol = 70/30; flow rate 1.0 mL/min; 25 °C; 254 nm; retention time: 6.7min (minor) and 11.3 min (major).

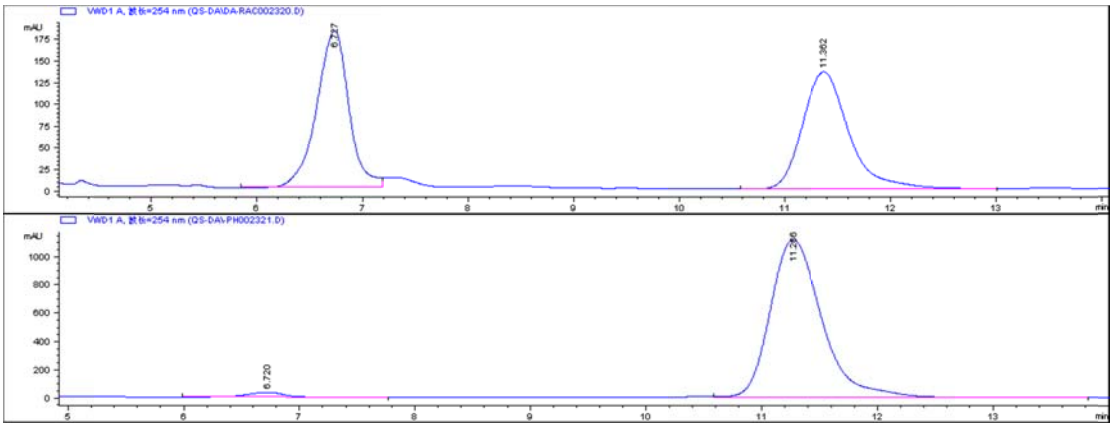

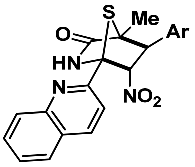

3b: Ar = 4-FPh

White solid, Mp 163.0–165.0 °C; 78% yield; 97% *ee*; $[\alpha]_D^{26}$ –296.6 (*c* 1.00, $CHCl_3$); ^1H NMR (300 MHz, $CDCl_3$) δ 8.34 (d, *J* = 8.4 Hz, 1H), 8.07 (d, *J* = 8.4 Hz, 1H), 7.90–7.87 (m, 2H), 7.81–7.72 (m, 2H), 7.68–7.61 (m, 3H), 7.16 (t, *J* = 8.6 Hz, 2H), 6.23 (d, *J* = 4.8 Hz, 1H), 4.01 (d, *J* = 4.8 Hz, 1H), 1.28 (s, 3H); ^{13}C NMR (75 MHz, $CDCl_3$) δ 176.7, 150.0, 147.1, 138.3, 131.9, 130.6, 130.3, 130.2, 129.6, 128.0, 127.7, 117.4, 116.2, 115.9, 98.6, 76.7, 66.9, 54.3, 13.8; HRMS (ESI) m/z 410.0972 (M+H$^+$), calc. for $C_{21}H_{17}FN_3O_3S$ 410.0975.

The *ee* was determined by HPLC analysis. CHIRALPAK IC (4.6 mm i.d. x 250 mm); Hexane/2-propanol = 70/30; flow rate 1.0 mL/min; 25 °C; 254 nm; retention time: 6.9 min (minor) and 11.9 min (major).

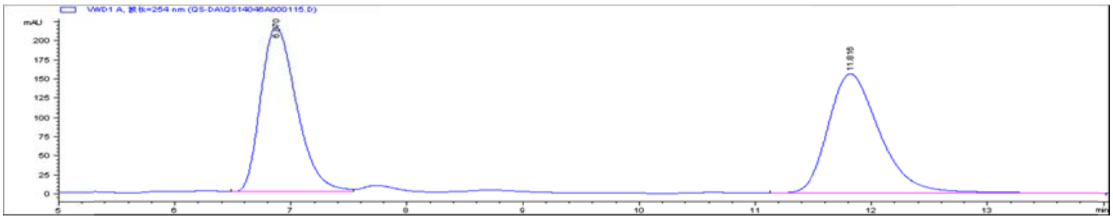

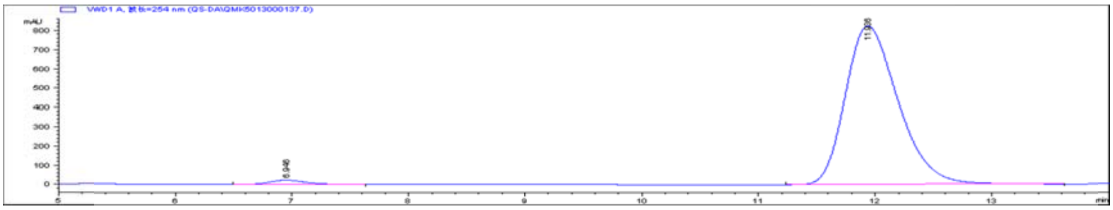

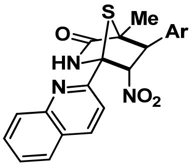

3c: Ar = 4-ClPh

White solid, Mp 97.5–98.6 °C; 89% yield; 94% *ee*; $[\alpha]_D^{18}$ –298.7 (*c* 1.00, $CHCl_3$); ^{1}H NMR (300 MHz, $CDCl_3$) δ 8.34 (d, *J* = 8.5 Hz, 1H), 8.07 (d, *J* = 8.5 Hz, 1H), 7.90–7.85 (m, 2H), 7.81–7.72 (m, 2H), 7.66–7.61 (m, 3H), 7.44 (d, *J* = 8.5 Hz, 2H), 7.26 (s, 2H), 6.22 (d, *J* = 4.8 Hz, 1H), 4.00 (d, *J* = 4.8 Hz, 1H), 1.29 (s, 4H); ^{13}C NMR (75 MHz, $CDCl_3$) δ 176.7, 150.0, 147.1, 138.3, 135.0, 134.6, 130.6, 129.8, 129.6, 129.3, 128.0, 127.9, 127.7, 117.4, 98.3, 76.8, 66.8, 54.4, 13.8; HRMS (ESI) m/z 426.0683 (M+H$^+$), calc. for $C_{21}H_{17}ClN_3O_3S$ 426.0679.

The *ee* was determined by HPLC analysis. CHIRALPAK IC (4.6 mm i.d. x 250 mm); Hexane/2-propanol = 70/30; flow rate 1.0 mL/min; 25 °C; 254 nm; retention time: 7.0 min (minor) and 12.4 min (major).

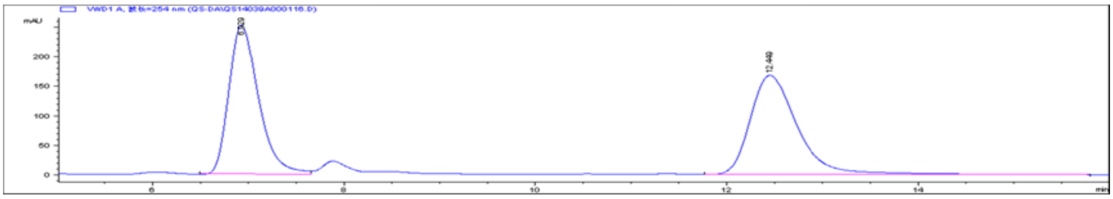

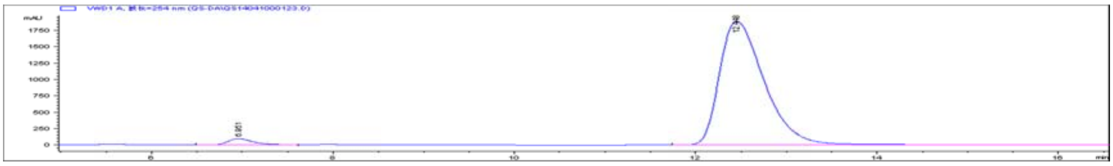

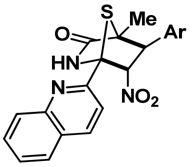

3d: Ar = 4-BrPh

White solid, Mp 95.4–97.1 °C; 84% yield; 96% *ee*; $[\alpha]_D^{21}$ –254.6 (*c* 1.00, $CHCl_3$); ^{1}H NMR (300 MHz, $CDCl_3$) δ 8.34 (d, *J* = 8.4 Hz, 1H), 8.07 (d, *J* = 8.4 Hz, 1H), 7.88 (d, *J* = 7.1 Hz, 2H), 7.81–7.72 (m, 2H), 7.66–7.54 (m, 5H), 6.22 (d, *J* = 4.8 Hz, 1H), 3.98 (d, *J* = 4.8 Hz, 1H), 1.29 (s, 3H); ^{13}C NMR (75 MHz, $CDCl_3$) δ 176.8, 150.0, 147.1, 138.3, 135.1, 132.2, 130.6, 130.2, 129.6, 128.0, 127.9, 127.6, 123.1, 117.4, 98.1, 76.8, 66.7, 54.5, 13.8; HRMS (ESI) m/z 470.0175 (M+H$^+$), calc. for $C_{21}H_{17}BrN_3O_3S$ 470.0174.

The *ee* was determined by HPLC analysis. CHIRALPAK IC (4.6 mm i.d. x 250 mm); Hexane/2-propanol = 70/30; flow rate 1.0 mL/min; 25 °C; 254 nm; retention time: 7.2 min (minor) and 12.9 min (major).

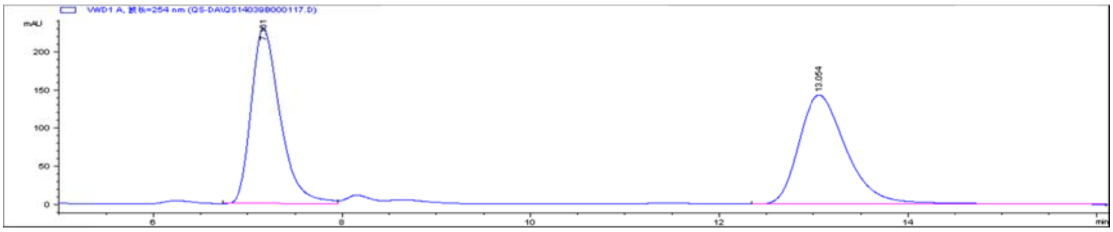

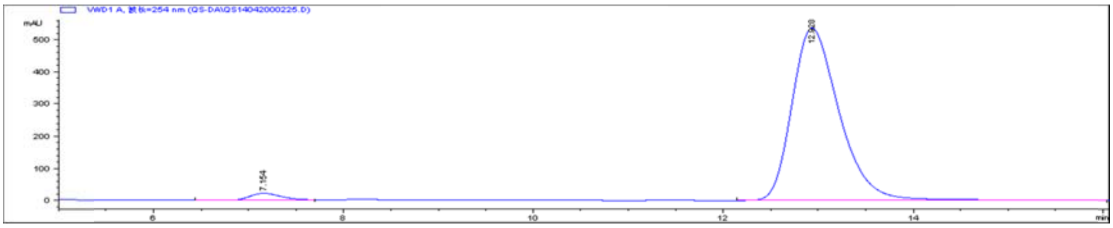

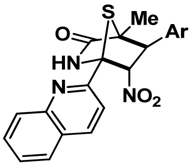

3e: Ar = 3-ClPh

White solid, Mp 163.2–165.8 °C; 85% yield; 92% *ee*; $[\alpha]_D^{26}$ –464.2 (*c* 1.00, $CHCl_3$); ^1H NMR (300 MHz, $CDCl_3$) δ 8.34 (d, *J* = 8.4 Hz, 1H), 8.08 (d, *J* = 8.4 Hz, 1H), 7.89 (d, *J* = 7.0 Hz, 2H), 7.81–7.73 (m, 3H), 7.66–7.61 (m, 1H), 7.53 –7.51 (m, 1H), 7.45–7.37 (m, 2H), 6.23 (d, *J* = 4.8 Hz, 1H), 4.00 (d, *J* = 4.8 Hz, 1H), 1.31 (s, 3H); ^{13}C NMR (75 MHz, $CDCl_3$) δ 176.5, 150.0, 147.1, 138.3, 138.2, 135.0, 130.6, 130.3, 129.6, 129.2, 128.2, 128.0, 127.9, 127.7, 127.2, 117.4, 98.3, 76.8, 66.8, 54.6, 13.8; HRMS (ESI) m/z 426.0684 ($M+H^+$), calc. for $C_{21}H_{17}ClN_3O_3S$ 426.0679.

The *ee* was determined by HPLC analysis. CHIRALPAK IC (4.6 mm i.d. x 250 mm); Hexane/2-propanol = 70/30; flow rate 1.0 mL/min; 25 °C; 254 nm; retention time: 7.0 min (minor) and 11.6 min (major).

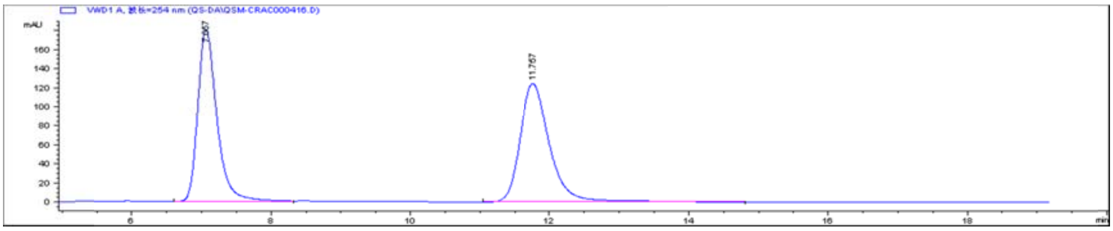

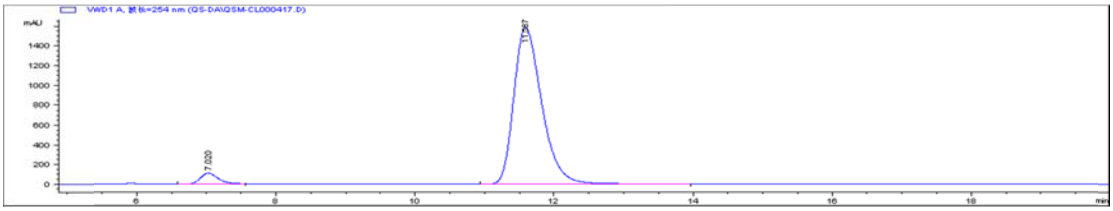

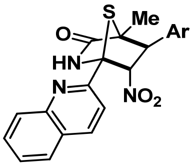

3f: Ar = 2-ClPh

White solid, Mp 184.0–185.6 °C; 82% yield; 95% *ee*; $[\alpha]_D^{26}$ –306.2 (*c* 1.00, $CHCl_3$); ^1H NMR (300 MHz, $CDCl_3$) δ 8.34 (d, *J* = 8.2 Hz, 1H), 8.10 (dd, *J* = 16.6, 8.2 Hz, 2H), 7.88 (d, *J* = 8.2 Hz, 1H), 7.77 (dd, *J* = 16.6, 7.6 Hz, 3H), 7.68 – 7.59 (m, 1H), 7.52 (d, *J* = 7.6 Hz, 1H), 7.41 (dt, *J* = 16.6, 7.6 Hz, 2H), 6.28 (d, *J* = 4.8 Hz, 1H), 4.89 (d, *J* = 4.8 Hz, 1H), 1.35 (s, 3H); ^{13}C NMR (75 MHz, $CDCl_3$) δ 176.3, 150.1, 147.2, 138.3, 135.9, 133.9, 130.7, 130.2, 130.0, 129.7, 128.1, 128.0, 127.8, 127.7, 127.6, 117.4, 77.0, 98.1, 67.5, 49.7, 12.9; HRMS (ESI) m/z 426.0675 ($M+H^+$), calc. for $C_{21}H_{17}ClN_3O_3S$ 426.0679.

The *ee* was determined by HPLC analysis. CHIRALPAK IC (4.6 mm i.d. x 250 mm); Hexane/2-propanol = 70/30; flow rate 1.0 mL/min; 25 °C; 254 nm; retention time: 5.8 min (minor) and 15.7 min (major).

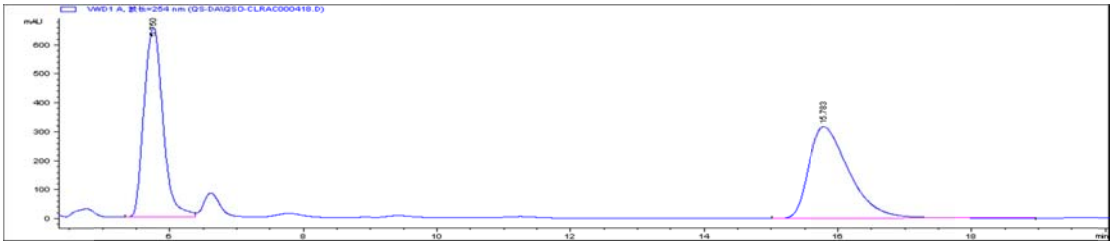

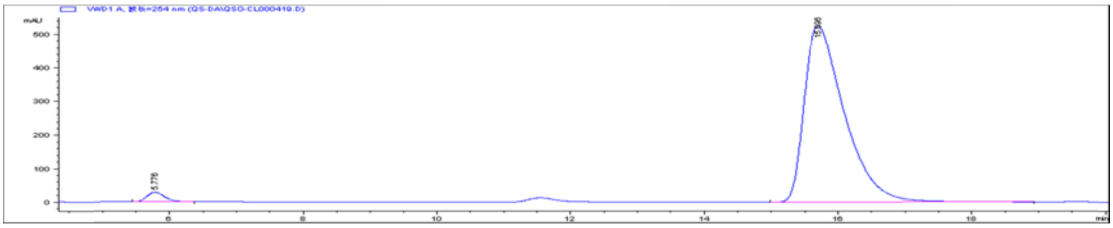

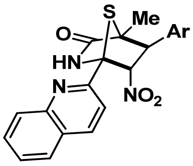

3g: Ar = 4-MePh

White solid, Mp 160.7–161.9 °C; 72% yield; 95% *ee*; $[\alpha]_D^{26}$ –120.4 (*c* 1.00, $CHCl_3$); ^{1}H NMR (300 MHz, $CDCl_3$) δ 8.33 (d, *J* = 8.4 Hz, 1H), 8.06 (d, *J* = 8.4 Hz, 1H), 7.88 (d, *J* = 8.4 Hz, 1H), 7.89–7.77 (m, 2H), 7.74 (d, *J* = 8.4 Hz, 1H), 7.65–7.60 (m, 1H), 7.54 (d, *J* = 8.1 Hz, 2H), 7.27 (s, 1H), 7.25 (s, 1H), 6.24 (d, *J* = 4.8 Hz, 1H), 4.00 (d, *J* = 4.8 Hz, 1H), 2.40 (s, 3H), 1.29 (s, 3H); ^{13}C NMR (75 MHz, $CDCl_3$) δ 176.8, 150.2, 147.1, 138.8, 138.2, 133.0, 130.6, 129.7, 129.6, 128.4, 128.0, 127.9, 127.7, 117.4, 98.8, 76.7, 67.1, 54.6, 21.2, 13.8; HRMS (ESI) m/z 406.1222 (M+H$^+$), calc. for $C_{22}H_{20}N_3O_3S$ 406.1225.

The *ee* was determined by HPLC analysis. CHIRALPAK IC (4.6 mm i.d. x 250 mm); Hexane/2-propanol = 70/30; flow rate 1.0 mL/min; 25 °C; 254 nm; retention time: 7.8 min (minor) and 14.2 min (major).

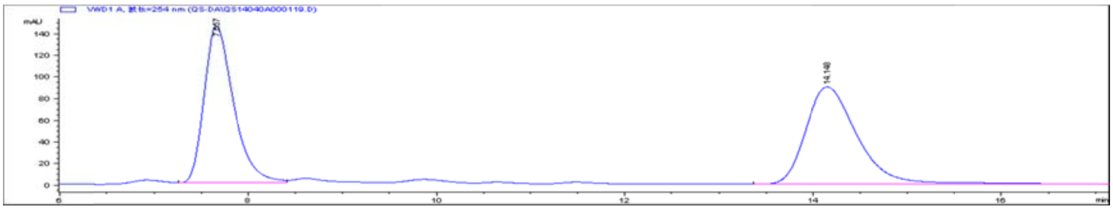

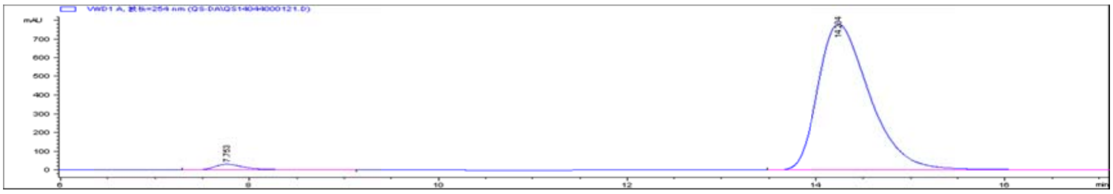

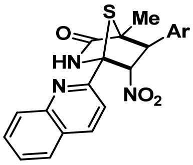

3h: Ar = 3-MePh

White solid, Mp 169.6–170.8 °C; 76% yield; 96% *ee*; $[\alpha]_D^{26}$ –218.1 (*c* 1.00, $CHCl_3$); ^{1}H NMR (300 MHz, $CDCl_3$) δ 8.34 (d, *J* = 8.5 Hz, 1H), 8.07 (d, *J* = 8.5 Hz, 1H), 7.88 (d, *J* = 8.2 Hz, 1H), 7.77 (dd, *J* = 16.3, 7.8 Hz, 3H), 7.63 (t, *J* = 7.5 Hz, 1H), 7.48 (d, *J* = 7.8 Hz, 1H), 7.43 (s, 1H), 7.34 (t, *J* = 7.5 Hz, 1H), 7.23 (s, 1H), 6.24 (d, *J* = 4.8 Hz, 1H), 3.99 (d, *J* = 4.8 Hz, 1H), 2.43 (s, 3H), 1.30 (s, 3H); ^{13}C NMR (75 MHz, $CDCl_3$) δ 176.8, 150.2, 147.1, 138.8, 138.2, 135.9, 130.6, 129.7, 129.6, 129.4, 129.0, 128.0, 127.9, 127.6, 125.5, 117.5, 98.8, 76.7, 67.1, 54.9, 21.6, 13.8; HRMS (ESI) m/z 406.1223 (M+H$^+$), calc. for $C_{22}H_{20}N_3O_3S$ 406.1225.

The *ee* was determined by HPLC analysis. CHIRALPAK IC (4.6 mm i.d. x 250 mm); Hexane/2-propanol = 70/30; flow rate 1.0 mL/min; 25 °C; 254 nm; retention time: 6.9 min (minor) and 12.9 min (major).

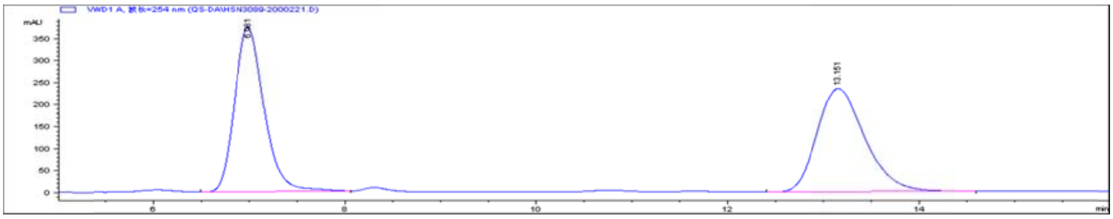

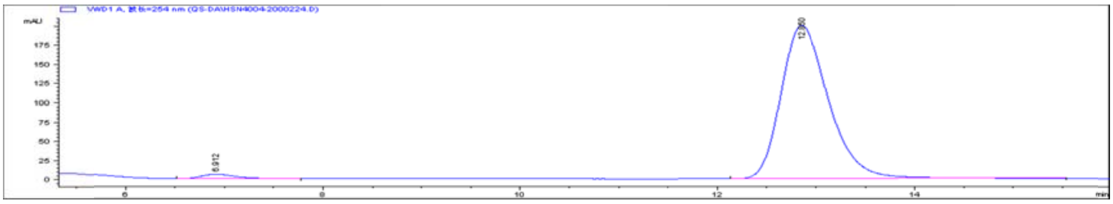

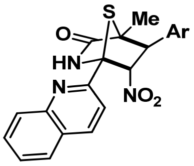

3i: Ar = 2-MePh

White solid, Mp 182.2–183.5 °C; 82% yield; 93% *ee*; $[\alpha]_D^{26}$ –34.6 (*c* 1.00, $CHCl_3$); ^{1}H NMR (300 MHz, $CDCl_3$) δ 8.33 (d, *J* = 8.3 Hz, 1H), 8.11–8.06 (m, 2H), 7.96 (s, 1H), 7.88 (d, *J* = 8.3 Hz, 1H), 7.80–7.73 (m, 2H), 7.65–7.60 (m, 1H), 7.38–7.29 (m, 3H), 6.24 (d, *J* = 4.9 Hz, 1H), 4.49 (d, *J* = 4.9 Hz, 1H), 2.40 (s, 3H), 1.29 (s, 3H); ^{13}C NMR (75 MHz, $CDCl_3$) δ 176.9, 150.2, 147.1, 138.2, 137.8, 134.6, 131.0, 130.6, 129.6, 128.5, 128.0, 127.8, 127.6, 126.9, 125.9, 117.4, 99.6, 76.8, 67.4, 48.9, 20.4, 13.0; HRMS (ESI) m/z 406.1229 (M+H$^+$), calc. for $C_{22}H_{20}N_3O_3S$ 406.1225.

The *ee* was determined by HPLC analysis. CHIRALPAK IC (4.6 mm i.d. x 250 mm); Hexane/2-propanol = 70/30; flow rate 1.0 mL/min; 25 °C; 254 nm; retention time: 5.3 min (minor) and 9.5 min (major).

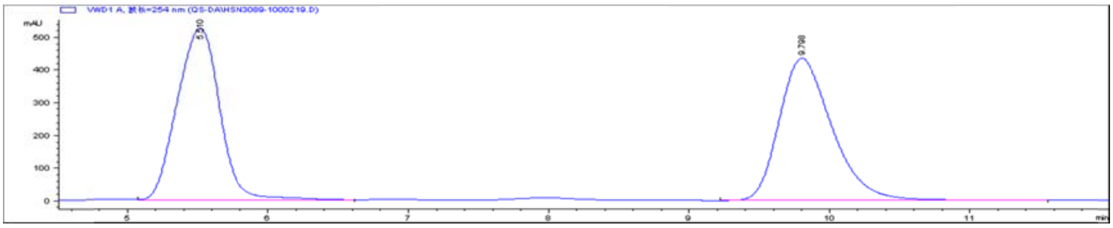

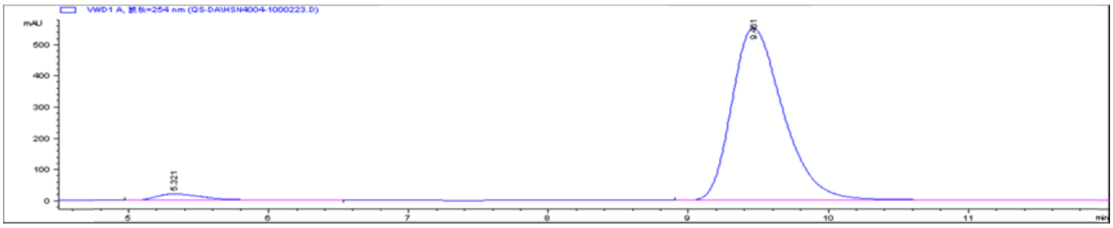

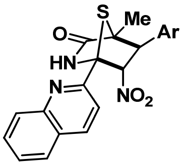

3j: Ar = 2-naphthyl

White solid, Mp 181.4–183.0 °C; 88% yield; 95% *ee*; $[\alpha]_D^{26}$ –384.7 (*c* 1.00, $CHCl_3$); ^{1}H NMR (300 MHz, $CDCl_3$) δ 8.36 (d, *J* = 9.1 Hz, 1H), 8.09 (d, *J* = 9.7 Hz, 2H), 7.97–7.85 (m, 6H), 7.79 (t, *J* = 7.6 Hz, 2H), 7.63 (t, *J* = 7.6 Hz, 1H), 7.61–7.54 (m, 2H), 6.40 (d, *J* = 4.7 Hz, 1H), 4.20 (d, *J* = 4.7 Hz, 1H), 1.32 (s, 3H); ^{13}C NMR (75 MHz, $CDCl_3$) δ 176.8, 150.2, 147.1, 138.3, 133.4, 133.3, 130.6, 129.6, 129.0, 128.4,

128.1, 127.9, 127.7, 126.7, 125.5, 117.5, 98.5, 76.8, 67.1, 55.2, 13.9; HRMS (ESI) m/z 442.1231 (M+H$^+$), calc. for $C_{25}H_{20}N_3O_3S$ 442.1225.

The *ee* was determined by HPLC analysis. CHIRALPAK IC (4.6 mm i.d. x 250 mm); Hexane/2-propanol = 70/30; flow rate 1.0 mL/min; 25 ^oC; 254 nm; retention time: 8.0 min (minor) and 16.9 min (major).

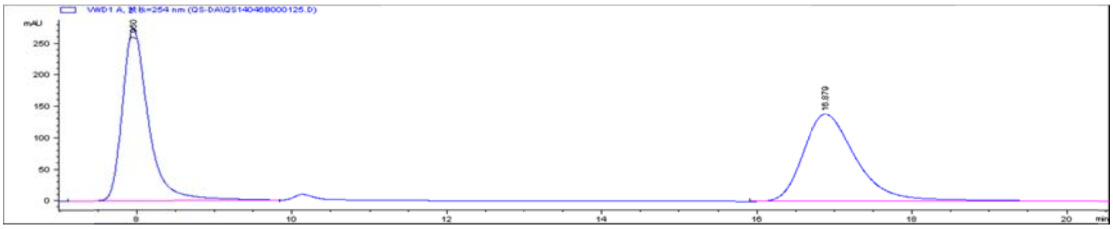

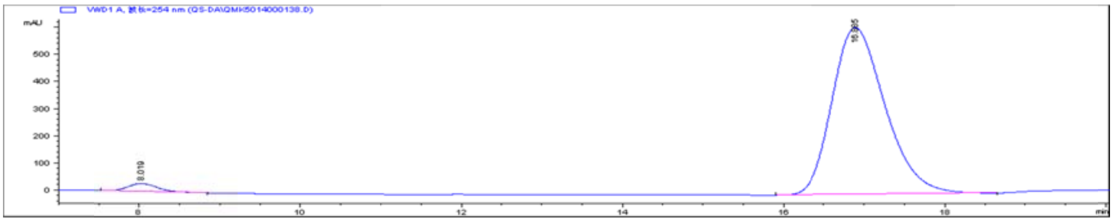

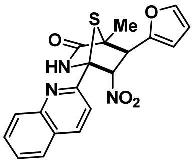

3k

White solid, Mp 156.2–157.9 ^oC; 76% yield; 94% *ee*; $[\alpha]_D^{26}$ –251.7 (*c* 1.00, $CHCl_3$); ^1H NMR (300 MHz, $CDCl_3$) δ 8.33 (d, *J* = 8.4 Hz, 1H), 8.07 (d, *J* = 8.4 Hz, 1H), 7.88 (d, *J* = 8.4 Hz, 1H), 7.83 (s, 1H), 7.78 (t, *J* = 7.6 Hz, 1H), 7.72 (d, *J* = 8.4 Hz, 1H), 7.63 (t, *J* = 7.6 Hz, 1H), 7.53 (d, *J* = 2.1 Hz, 1H), 6.56 (d, *J* = 2.1 Hz, 1H), 6.51 – 6.44 (m, 1H), 6.27 (d, *J* = 4.6 Hz, 1H), 4.27 (d, *J* = 4.6 Hz, 1H), 1.41 (s, 3H); ^{13}C NMR (75 MHz, $CDCl_3$) δ 176.1, 149.9, 149.2, 147.0, 143.4, 138.3, 130.6, 129.6, 128.0, 127.9, 127.6, 117.3, 110.8, 109.9, 96.8, 76.8, 66.4, 49.3, 13.4; HRMS (ESI) m/z 382.0864 (M+H$^+$), calc. for $C_{19}H_{16}N_3O_4S$ 382.0862.

The *ee* was determined by HPLC analysis. CHIRALPAK IC (4.6 mm i.d. x 250 mm); Hexane/2-propanol = 70/30; flow rate 1.0 mL/min; 25 ^oC; 254 nm; retention time: 8.9 min (minor) and 14.7 min (major).

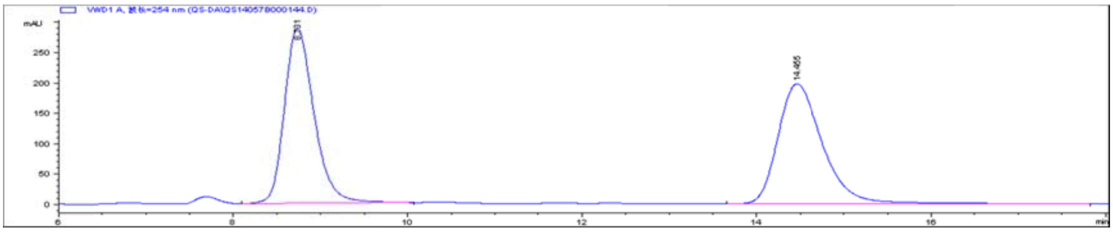

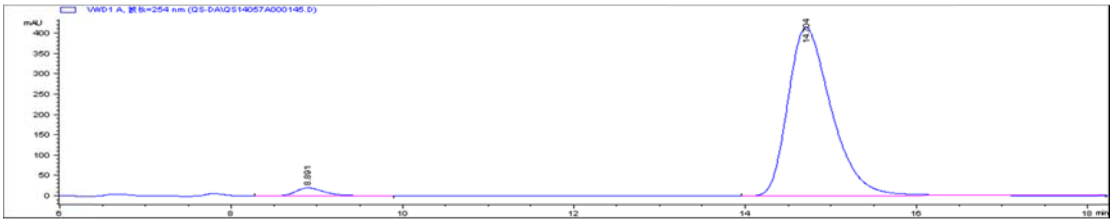

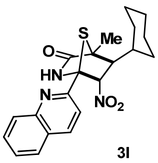

3l

White solid, Mp 94.0–95.6 °C; 41% yield; 82% *ee*; $[\alpha]_D^{26}$ –136.7 (*c* 1.00, $CHCl_3$); ^1H NMR (300 MHz, $CDCl_3$) δ 8.30 (d, *J* = 8.6 Hz, 1H), 8.05 (d, *J* = 8.6 Hz, 1H), 7.86 (d, *J* = 7.6 Hz, 1H), 7.77 (d, *J* = 7.6 Hz, 2H), 7.61 (d, *J* = 7.6 Hz, 2H), 5.85 (d, *J* = 4.3 Hz, 1H), 2.97 (s, 1H), 2.09–1.78 (m, 5H), 1.70 (s, 3H), 1.37–1.19 (m, 6H); ^{13}C NMR (75 MHz, $CDCl_3$) δ 177.1, 149.9, 147.0, 138.2, 130.6, 129.6, 128.0, 127.8, 127.6, 117.3, 95.0, 64.5, 52.9, 37.8, 32.0, 28.5, 26.5, 26.2, 26.0, 13.3; HRMS (ESI) m/z 398.1536 (M+H$^+$), calc. for $C_{21}H_{24}N_3O_3S$ 398.1538.

The *ee* was determined by HPLC analysis. CHIRALPAK IA (4.6 mm i.d. x 250 mm); Hexane/2-propanol = 90/10; flow rate 1.0 mL/min; 25 °C; 254 nm; retention time: 14.8 min (minor) and 16.5 min (major).

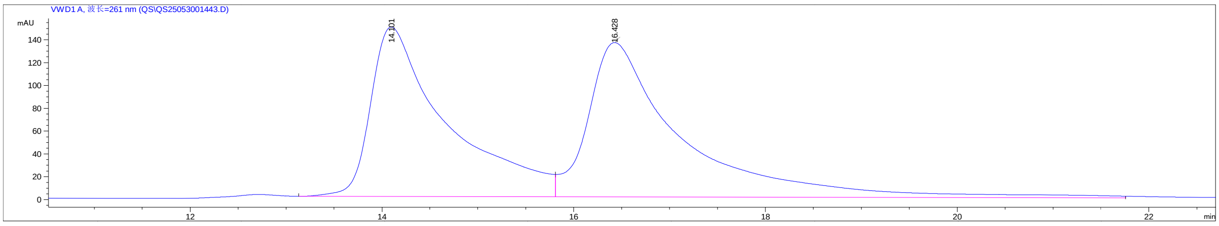

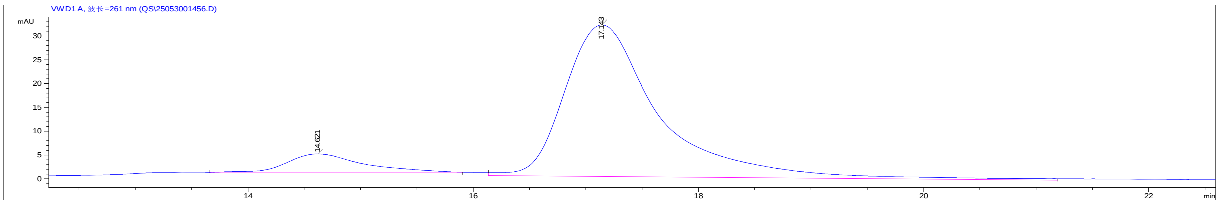

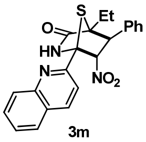

3m

Green solid, Mp 131.8–132.9 °C; 55% yield; 98% *ee*; $[\alpha]_D^{26}$ –201.9 (*c* 1.98, $CHCl_3$); ^1H NMR (300 MHz, $CDCl_3$) δ 8.33 (d, *J* = 8.4 Hz, 1H), 8.06 (d, *J* = 8.4 Hz, 1H), 7.90–7.86 (m, 2H), 7.78–7.74 (m, 2H), 7.67–7.59 (m, 3H), 7.45-7.43 (m, 3H), 6.30 (d, *J* = 4.8 Hz, 1H), 4.03 (d, *J* = 4.8 Hz, 1H), 2.08–1.96 (m, 1H), 1.33–1.21 (m, 1H), 0.95 (t, *J* = 7.3 Hz, 3H); ^{13}C NMR (75 MHz, $CDCl_3$) δ 176.2, 150.5, 147.1, 138.2, 136.3, 130.5, 129.6, 129.0, 128.8, 128.0, 127.8, 127.6, 117.6, 98.6, 76.0, 73.6, 55.2, 21.4, 10.9; HRMS (ESI) m/z 406.1228 (M+H$^+$), calc. for $C_{22}H_{20}N_3O_3S$ 406.1225.

The *ee* was determined by HPLC analysis. CHIRALPAK IC (4.6 mm i.d. x 250 mm); Hexane/2-propanol = 80/20; flow rate 1.0 mL/min; 25 °C; 254 nm; retention time: 7.7min (minor) and 11.6 min (major).

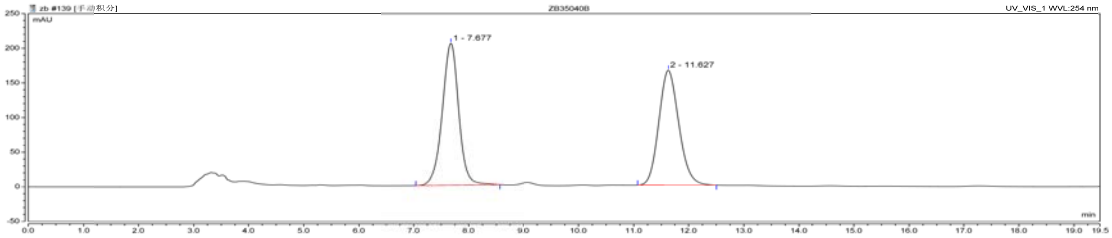

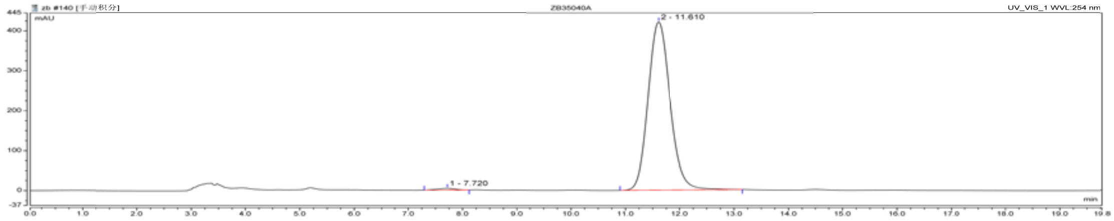

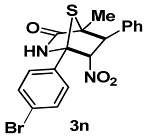

3n

White solid, Mp 165.3–171.9 °C; 45% yield; 90% *ee*; $[\alpha]_D^{26}$ –181.8 (*c* 1.48, $CHCl_3$); ^1H NMR (300 MHz, $CDCl_3$) δ 8.22 (s, 1H), 7.64–7.57 (m, 4H), 7.50–7.49 (m, 2H), 7.43–7.41 (m, 3H), 5.98 (d, *J* = 4.7 Hz, 1H), 3.90 (d, *J* = 4.7 Hz, 1H), 1.23 (s, 3H); ^{13}C NMR (75 MHz, $CDCl_3$) δ 178.2, 135.6, 132.6, 130.3, 129.1, 128.5, 127.4, 124.3, 98.5, 76.3, 66.1, 56.1, 13.4; HRMS (ESI) m/z 419.0064 ($M+H^+$), calc. for $C_{18}H_{16}BrN_2O_3S$ 419.0065.

The *ee* was determined by HPLC analysis. CHIRALPAK IC (4.6 mm i.d. x 250 mm); Hexane/2-propanol = 90/10; flow rate 1.0 mL/min; 25 °C; 254 nm; retention time: 6.9 min (minor) and 14.2 min (major).

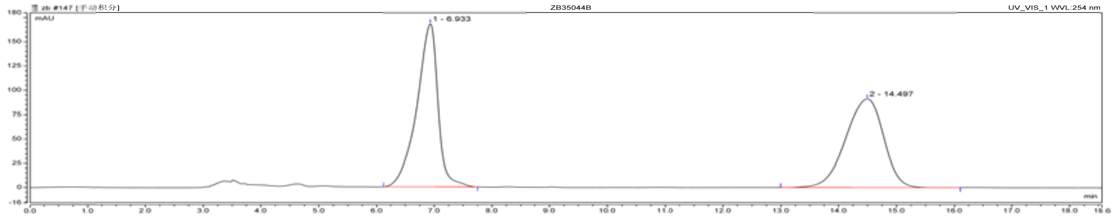

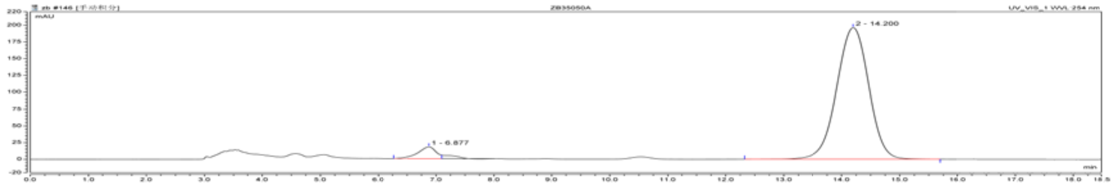

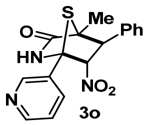

3o

White solid, Mp 136.9–138.1 °C; 76% yield; 95% *ee*; $[\alpha]_D^{27}$ –569.3 (*c* 1.00, $CHCl_3$); ^1H NMR (300 MHz, $CDCl_3$) δ 8.62–8.61 (m, 1H), 7.89–7.83 (m, 2H), 7.70 (d, *J* = 7.9 Hz, 1H), 7.64–7.61 (m, 2H), 7.45–7.35 (m, 4H), 6.21 (d, *J* = 4.8 Hz, 1H), 3.91 (d, *J* = 4.8 Hz, 1H), 1.24 (s, 3H); ^{13}C NMR (75 MHz, $CDCl_3$) δ 177.4), 150.8, 149.6, 137.7, 136.0, 129.0, 128.9, 128.6, 124.5, 120.8, 98.2, 76.6, 66.6, 55.3, 13.7; HRMS (ESI) m/z 342.0911 ($M+H^+$), calc. for $C_{17}H_{16}N_3O_3S$ 342.0912.

The *ee* was determined by HPLC analysis. CHIRALPAK IC (4.6 mm i.d. x 250 mm); Hexane/2-propanol = 70/30; flow rate 1.0 mL/min; 25 °C; 254 nm; retention time: 7.3 min (minor) and 13.0 min (major).

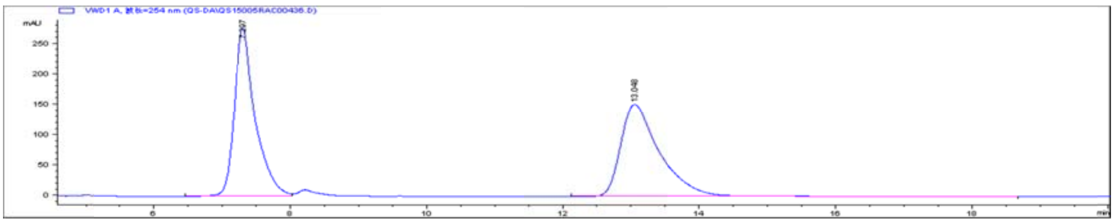

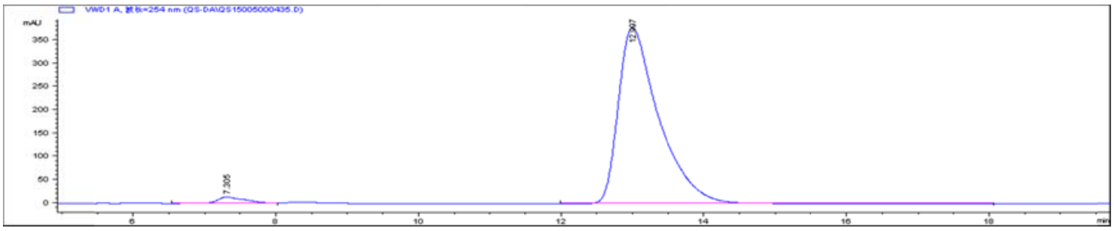

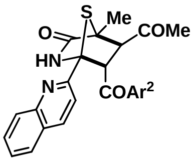

6a: Ar^2 = Ph

White solid, Mp 164.9–165.8 °C; 86% yield; 98% *ee*; $[\alpha]_D^{24}$ –169.6 (*c* 2.37, $CHCl_3$); ^1H NMR (300 MHz, $CDCl_3$) δ 8.14 (d, *J* = 8.4 Hz, 1H), 7.72 (d, *J* = 8.4 Hz, 2H), 7.68–7.63 (m, 3H), 7.61–7.56 (m, 2H), 7.48 (dd, *J* = 7.8, 7.2 Hz, 1H), 7.34 (t, *J* = 7.2 Hz, 1H), 7.22 (t, *J* = 7.8 Hz, 2H), 5.70 (d, *J* = 5.4 Hz, 1H), 3.56 (d, *J* = 5.4 Hz, 1H), 2.28 (s, 3H), 1.77 (s, 3H); ^{13}C NMR (75 MHz, $CDCl_3$) δ 204.9, 198.2, 176.5, 152.2, 146.7, 137.6, 136.9, 133.1, 129.9, 129.2, 128.4, 128.0, 127.5, 127.3, 127.2, 118.2, 79.6, 66.6, 62.0, 58.4, 32.8, 14.4; HRMS (ESI) m/z 417.1277 (M+H$^+$), calc. for $C_{24}H_{21}N_2O_3S$ 417.1273.

The *ee* was determined by HPLC analysis. CHIRALPAK IA (4.6 mm i.d. x 250 mm); Hexane/2-propanol = 80/20; flow rate 1.0 mL/min; 25 °C; 254 nm; retention time: 24.2 min (minor) and 41.6 min (major).

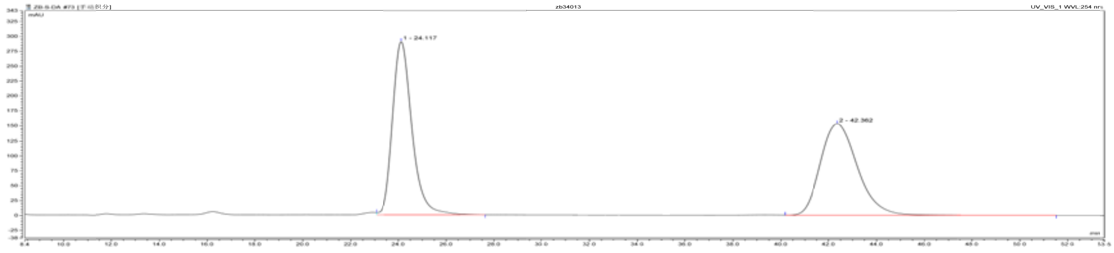

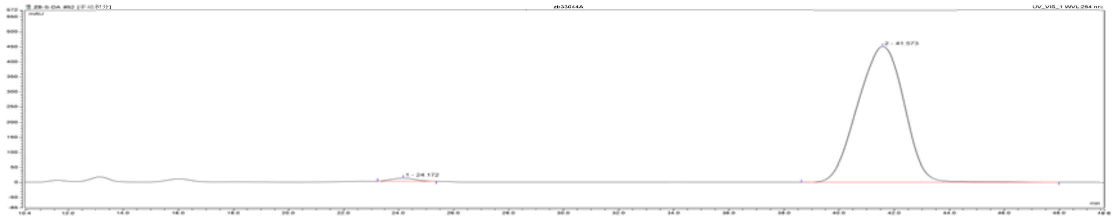

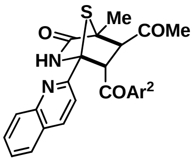

6b: Ar^2 = 4-CF_3Ph

White solid, Mp 181.7–183.6 °C; 90% yield; 98% *ee*; $[\alpha]_D^{25}$ –144.8 (*c* 4.22, ^1H NMR (300 MHz, $CDCl_3$) δ 8.13 (d, *J* = 8.4 Hz, 1H), 7.71–7.67 (m, 4H), 7.60–7.53 (m, 3H), 7.51–7.46 (m, 1H), 7.38 (d, *J* = 8.4 Hz, 2H), 5.65 (d, *J* = 5.2 Hz, 1H), 3.67 (d, *J* = 5.2 Hz, 1H), 2.32 (s, 3H), 1.79 (s, 3H); ^{13}C NMR (75 MHz, $CDCl_3$) δ 204.6, 197.2, 176.2, 151.7, 146.5, 139.8, 137.8, 134.2, 133.8, 130.2, 128.8, 128.2, 127.5, 127.4, 125.2, 125.1, 125.0, 121.3, 118.2, 79.7, 66.7, 62.4, 58.2, 32.6, 14.5; HRMS (ESI) m/z 485.1149 (M+H$^+$), calc. for $C_{25}H_{20}F_3N_2O_3S$ 485.1147.

The *ee* was determined by HPLC analysis. CHIRALPAK IA (4.6 mm i.d. x 250 mm); Hexane/2-propanol = 80/20; flow rate 1.0 mL/min; 25 °C; 230 nm; retention time: 15.7 min (minor) 24.9 min (major).

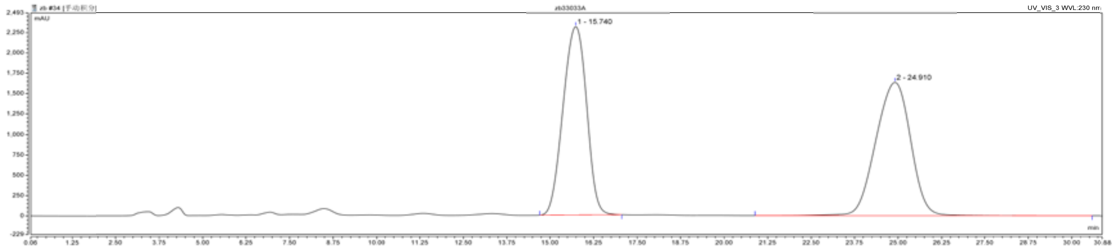

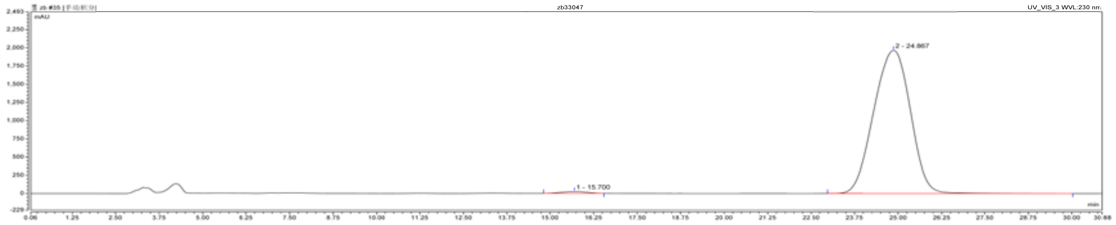

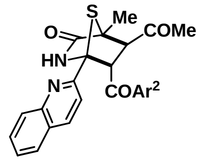

6c: Ar^2 = 4-ClPh

Pale yellow solid, Mp 143.2–145.0 °C; 80% yield; 97% *ee*; $[\alpha]_D^{26}$ –172.8 (*c* 2.92, $CHCl_3$); ^{1}H NMR (300 MHz, $CDCl_3$) δ 8.16 (d, *J* = 8.7 Hz, 1H), 7.73 (t, *J* = 8.7 Hz, 2H), 7.66–7.58 (m, 4H), 7.52–7.48 (m, 2H), 7.18 (d, *J* = 8.7 Hz, 2H), 5.64 (d, *J* = 5.3 Hz, 1H), 3.56 (d, *J* = 5.3 Hz, 1H), 2.30 (s, 3H), 1.78 (s, 3H); ^{13}C NMR (75 MHz, $CDCl_3$) δ 204.7, 197.0, 176.4, 152.0, 146.7, 139.7, 137.7, 135.3, 130.1, 129.4, 129.1, 128.6, 127.6, 127.4, 118.3, 79.8, 66.7, 61.6, 58.5, 32.7, 14.5; HRMS (ESI) m/z 451.0881 (M+H$^+$), calc. for $C_{24}H_{20}ClN_2O_3S$ 451.0883.

The *ee* was determined by HPLC analysis. CHIRALPAK IA (4.6 mm i.d. x 250 mm); Hexane/2-propanol = 80/20; flow rate 1.0 mL/min; 25 °C; 254 nm; retention time: 21.5 min (minor) and 32.7 min (major).

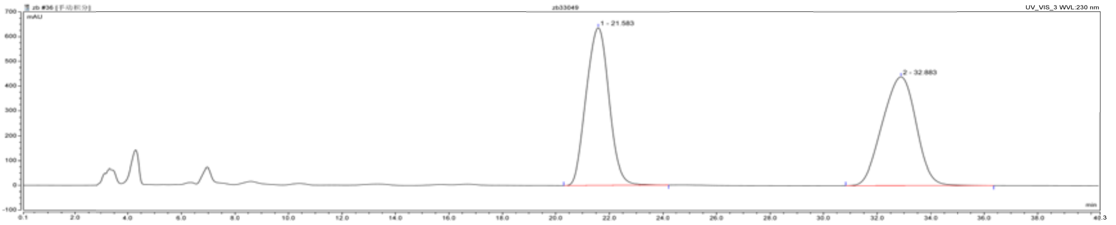

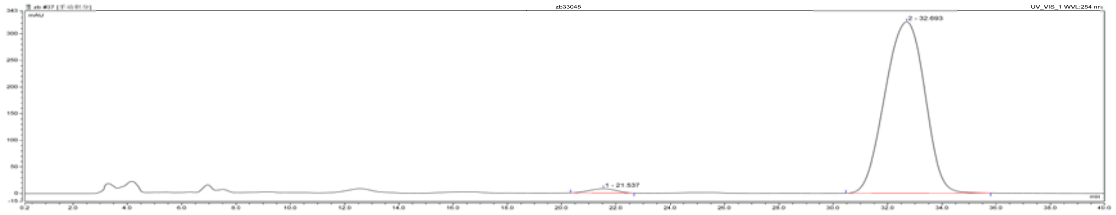

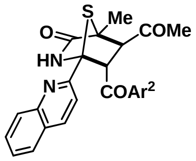

6d: Ar^2 = 4-BrPh

White solid, Mp 175.2–176.5 °C; 91% yield; 99% *ee*; $[\alpha]_D^{26}$ –167.5 (*c* 1.26, $CHCl_3$); ^{1}H NMR (300 MHz, $CDCl_3$) δ 8.18 (d, *J* = 8.4 Hz, 1H), 7.76 (d, *J* = 8.4 Hz, 1H), 7.71 (d, *J* = 8.4 Hz, 1H), 7.67–7.60 (m, 2H), 7.54–7.49 (m, 3H), 7.42 (s, 1H), 7.35 (d, *J* = 8.4 Hz, 2H), 5.62 (d, *J* = 5.3 Hz, 1H), 3.57 (d, *J* = 5.3 Hz, 1H), 2.31 (s, 3H), 1.79 (s, 3H); ^{13}C NMR (75 MHz, $CDCl_3$) δ 204.7, 197.2, 176.4, 151.9, 146.7, 137.8, 135.7, 131.7, 130.2, 129.5, 129.1, 128.4, 127.6, 127.5, 127.4, 118.2, 79.7, 66.7, 61.7, 58.4, 32.8, 14.5; HRMS (ESI) m/z 495.0381 (M+H$^+$), calc. for $C_{24}H_{20}BrN_2O_3S$ 495.0378.

The *ee* was determined by HPLC analysis. CHIRALPAK IA (4.6 mm i.d. x 250 mm); Hexane/2-propanol = 80/20; flow rate 1.0 mL/min; 25 °C; 254 nm; retention time: 21.8min (minor) and 32.0 min (major).

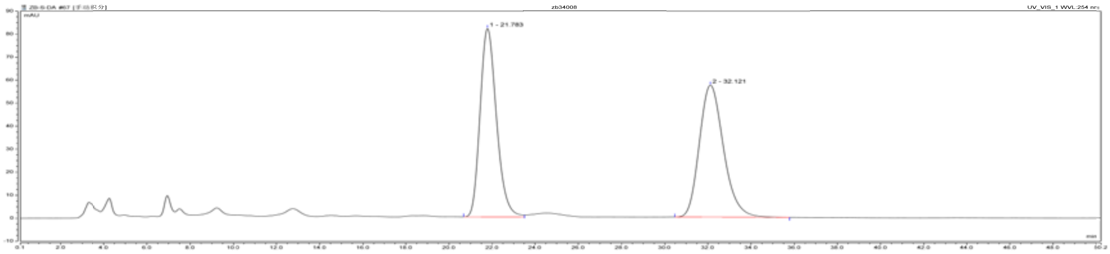

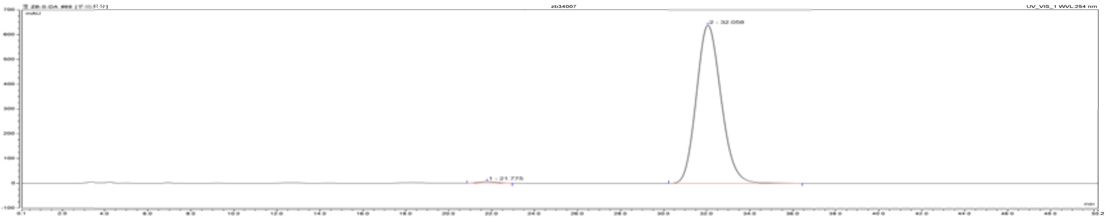

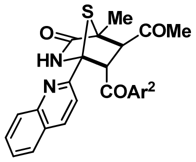

6e: Ar^2 = 3,4-Cl_2Ph

White solid, Mp 176.8–178.7 °C; 95% yield; 94% *ee*; $[\alpha]^{25}_{D}$ –191.5 (*c* 4.46, $CHCl_3$); ^{1}H NMR (300 MHz, $CDCl_3$) δ 8.20 (d, *J* = 8.4 Hz, 1H), 7.76–7.68 (m, 3H), 7.62–7.58 (m, 3H), 7.53–7.48 (m, 1H), 7.44 (dd, *J* = 8.4, 1.9 Hz, 1H), 7.20 (d, *J* = 8.4 Hz, 1H), 5.52 (d, *J* = 5.2 Hz, 1H), 3.69 (d, *J* = 5.2 Hz, 1H), 2.34 (s, 3H), 1.79 (s, 3H); ^{13}C NMR (75 MHz, $CDCl_3$) δ 204.6, 195.6, 176.2, 151.7, 146.5, 137.8, 137.5, 136.4, 132.8, 130.3, 130.2, 129.8, 128.9, 127.6, 127.5, 127.4, 127.0, 118.2, 79.7, 66.7, 62.2, 58.1, 32.6, 14.5; HRMS (ESI) m/z 485.0494 (M+H$^+$), calc. for $C_{24}H_{19}Cl_2N_2O_3S$ 485.0493.

The *ee* was determined by HPLC analysis. CHIRALPAK IC (4.6 mm i.d. x 250 mm); Hexane/2-propanol = 80/20; flow rate 1.0 mL/min; 25 °C; 254 nm; retention time: 15.9min (minor) and 30.3 min (major).

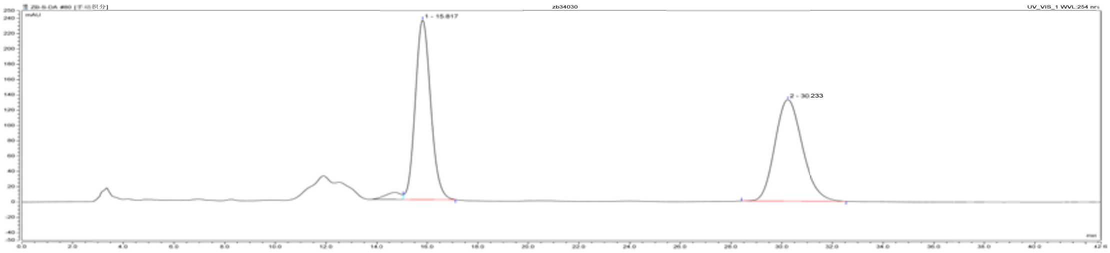

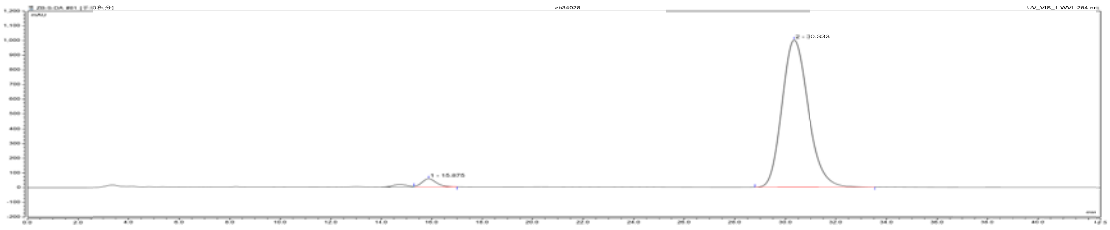

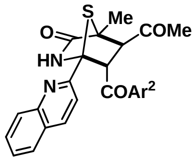

6f: Ar^2 = 4-MeOPh

White solid, Mp 148.5–150.0 °C; 87% yield; 97% *ee*; $[\alpha]_{25D}$ –190.5 (*c* 3.78, $CHCl_3$); ^{1}H NMR (300 MHz, $CDCl_3$) δ 8.14 (d, *J* = 8.7 Hz, 1H), 7.73 (dd, *J* = 8.7, 5.3 Hz, 3H), 7.65 (t, *J* = 5.3 Hz, 2H), 7.67–7.55 (m, 2H), 7.51–7.46 (m, 1H), 6.69 (d, *J* = 8.7 Hz, 2H), 5.64 (d, *J* = 5.4 Hz, 1H), 3.72 (s, 3H), 3.54 (d, *J* = 5.4 Hz, 1H), 2.29 (s,

3H), 1.76 (s, 3H); ^{13}C NMR (75 MHz, $CDCl_3$) δ 205.1, 196.3, 176.6, 163.6, 152.4, 146.8, 137.5, 130.4, 129.9, 129.8, 129.4, 127.6, 127.3, 127.2, , 118.2, 113.6, 79.7, 66.6, 61.5, 58.7, 55.4, 32.8, 14.4; HRMS (ESI) m/z 447.1377 ($M+H^+$), calc. for $C_{25}H_{23}N_2O_4S$ 447.1379.

The *ee* was determined by HPLC analysis. CHIRALPAK IA (4.6 mm i.d. x 250 mm); Hexane/2-propanol = 80/20; flow rate 1.0 mL/min; 25 °C; 254 nm; retention time: 34.1 min (minor) and 48.8 min (major).

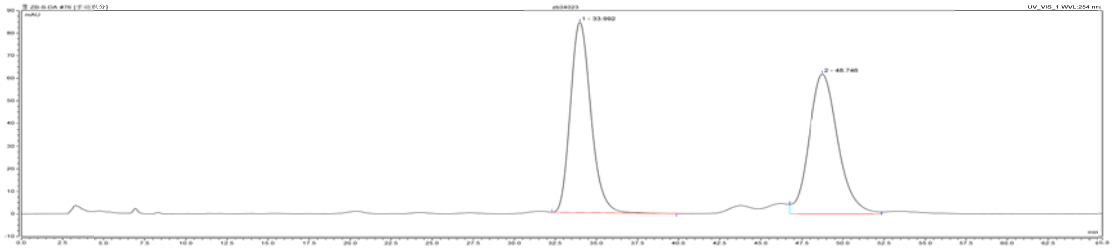

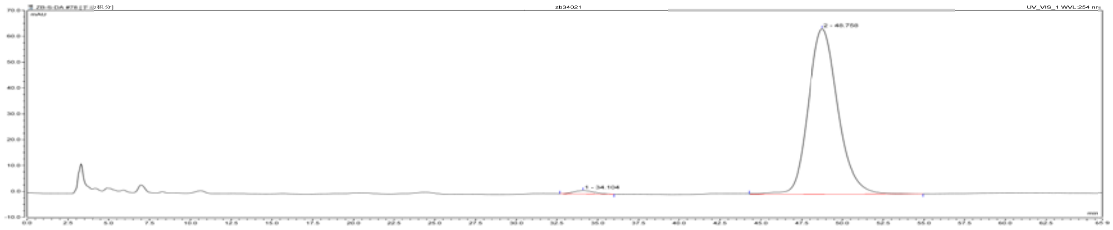

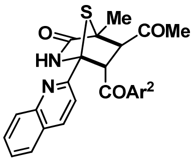

6g: Ar^2 = 3-MeOPh

White solid, Mp 149.1–150.8 °C; 82% yield; 96% *ee*; $[\alpha]_D^{24}$ –192.4 (*c* 1.47, $CHCl_3$); ^{1}H NMR (300 MHz, $CDCl_3$) δ 8.17 (d, *J* = 8.3 Hz, 1H), 7.73 (t, *J* = 8.3 Hz, 3H), 7.62 (dd, *J* = 11.2, 4.6 Hz, 1H), 7.53–7.47 (m, 2H), 7.22 (d, *J* = 7.7 Hz, 1H), 7.14 (dt, *J* = 11.2, 4.6 Hz, 2H), 6.89–6.86 (m, 1H), 5.65 (d, *J* = 5.4 Hz, 1H), 3.72 (s, 3H), 3.55 (d, *J* = 5.4 Hz, 1H), 2.30 (s, 3H), 1.78 (s, 3H); ^{13}C NMR (75 MHz, $CDCl_3$) δ 204.9, 197.9, 176.4, 159.5, 152.1, 146.8, 138.2, 137.6, 130.0, 129.4, 129.3, 127.6, 127.4, 127.3, 120.6, 119.9, 118.2, 112.0, 79.5, 66.6, 62.5, 58.4, 55.34, 32.9, 14.4; HRMS (ESI) m/z 447.1381 ($M+H^+$), calc. for $C_{25}H_{23}N_2O_4S$ 447.1379.

The *ee* was determined by HPLC analysis. CHIRALPAK IA (4.6 mm i.d. x 250 mm); Hexane/2-propanol = 80/20; flow rate 1.0 mL/min; 25 °C; 254 nm; retention time: 23.1 min (minor) and 42.7 min (major).

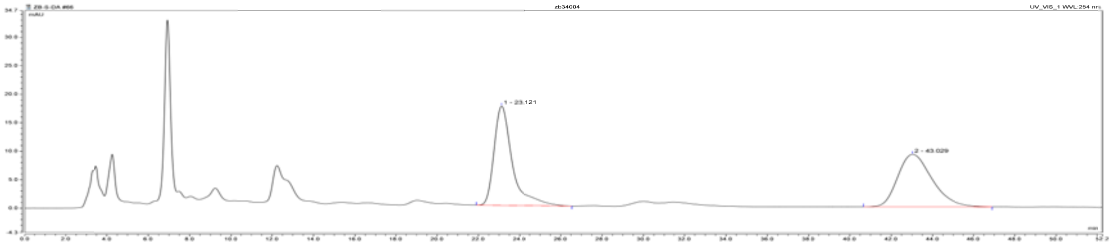

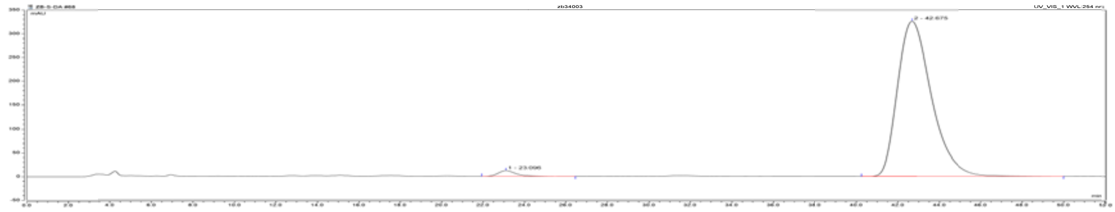

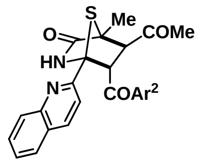

6h: Ar^2 = 2-MeOPh

Weak yellow solid, Mp 132.1–133.8 °C; 64% yield; 94% *ee*; $[\alpha]_D^{26}$ −198.8 (*c* 2.28, $CHCl_3$); ^1H NMR (300 MHz, $CDCl_3$) δ 8.00 (d, *J* = 8.5 Hz, 1H), 7.79 (d, *J* = 8.5 Hz, 1H), 7.70–7.62 (m, 3H), 7.56 (d, *J* = 8.5 Hz, 1H), 7.50 (t, *J* = 7.5 Hz, 1H), 7.03–6.97 (m, 2H), 6.54–6.46 (m, 2H), 5.52 (d, *J* = 5.2 Hz, 1H), 3.80 (d, *J* = 5.2 Hz, 1H), 3.72 (s, 3H), 2.32 (s, 3H), 1.74 (s, 3H); ^{13}C NMR (75 MHz, $CDCl_3$) δ 205.0, 199.3, 176.2, 156.8, 151.9, 146.6, 137.0, 132.8, 129.9, 129.7, 129.3, 128.5, 127.4, 127.3, 127.2, 120.3, 117.6, 110.4, 78.8, 68.5, 66.4, 57.1, 55.0, 32.3, 14.0; HRMS (ESI) m/z 447.1383 ($M+H^+$), calc. for $C_{25}H_{23}N_2O_4S$ 447.1379.

The *ee* was determined by HPLC analysis. CHIRALPAK IC (4.6 mm i.d. x 250 mm); Hexane/2-propanol = 70/30; flow rate 1.0 mL/min; 25 °C; 254 nm; retention time: 25.0 min (minor) and 31.4 min (major).

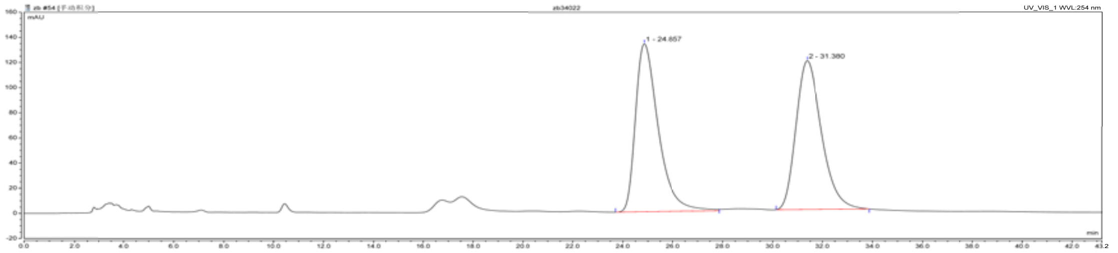

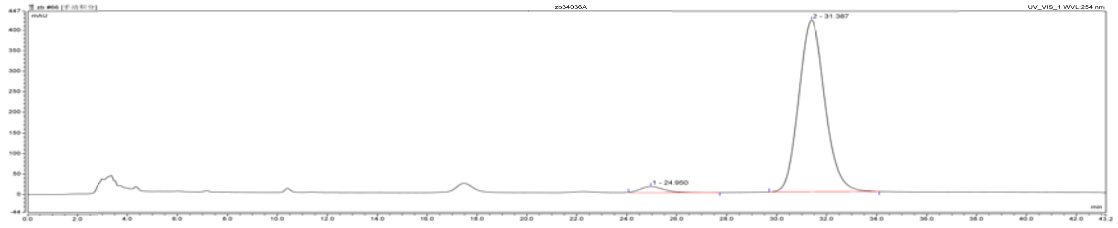

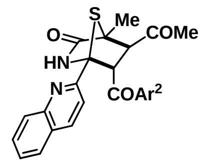

6i: Ar^2 = 2-BrPh

White solid, Mp 151.5–153.0 °C; 60% yield; 99% *ee*; $[\alpha]_D^{25}$ −170.0 (*c* 0.725, $CHCl_3$); ^1H NMR (300 MHz, $CDCl_3$) δ 7.94 (d, *J* = 8.6 Hz, 1H), 7.85 (d, *J* = 8.6 Hz, 1H), 7.72 (s, 1H), 7.67–7.62 (m, 2H), 7.54–7.45 (m, 2H), 7.11 (dd, *J* = 7.6, 1.6 Hz, 1H), 6.83 (dd, *J* = 7.6, 1.6 Hz, 1H), 6.76–6.65 (m, 2H), 5.44 (d, *J* = 5.1 Hz, 1H), 3.90 (d, *J* = 5.1 Hz, 1H), 2.29 (s, 3H), 1.71 (s, 3H); ^{13}C NMR (75 MHz, $CDCl_3$) δ 204.6, 198.8, 175.9, 151.4, 146.5, 140.2, 137.5, 132.9, 131.2, 130.2, 129.3, 128.7, 127.5, 127.4, 126.6, 118.6, 117.6, 78.2, 68.2, 66.5, 56.6, 32.6, 14.3; HRMS (ESI) m/z 495.0375 ($M+H^+$), calc. for $C_{24}H_{20}BrN_2O_3S$ 495.0378.

The *ee* was determined by HPLC analysis. CHIRALPAK IA (4.6 mm i.d. x 250 mm); Hexane/2-propanol = 80/20; flow rate 1.0 mL/min; 25 °C; 254 nm; retention time: 20.7 min (major) and 23.0 min (minor).

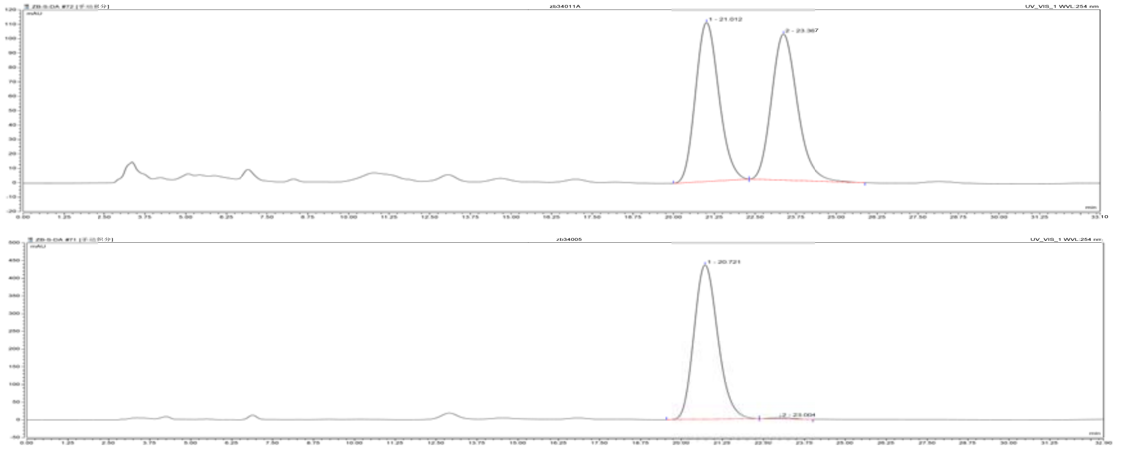

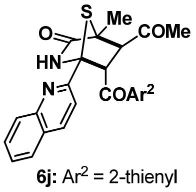

6j: Ar^2 = 2-thienyl

White solid, Mp 179.1–181.0 °C; 98% yield; 99% *ee*; $[\alpha]_D^{26}$ –147.2 (*c* 4.06, $CHCl_3$); ^{1}H NMR (300 MHz, $CDCl_3$) δ 8.19 (d, *J* = 8.2 Hz, 1H), 7.76–7.73 (m, 3H), 7.65 (s, 1H), 7.63–7.58 (m, 1H), 7.51 (d, *J* = 8.2 Hz, 1H), 7.46–7.44 (m, 2H), 6.89 –6.86 (m, 1H), 5.52 (d, *J* = 5.4 Hz, 1H), 3.65 (d, *J* = 5.4 Hz, 1H), 2.35 (s, 3H), 1.78 (s, 3H); ^{13}C NMR (75 MHz, $CDCl_3$) δ 204.8, 190.2, 176.6, 152.1, 146.8, 144.2, 137.6, 134.7, 132.5, 130.0, 129.3, 128.0, 127.6, 127.4, 127.3, 118.3, 79.8, 66.7, 62.5, 59.0, 32.8, 14.4; HRMS (ESI) m/z 423.0833 (M+H$^+$), calc. for $C_{22}H_{19}N_2O_3S_2$ 423.0837.

The *ee* was determined by HPLC analysis. CHIRALPAK IA (4.6 mm i.d. x 250 mm); Hexane/2-propanol = 80/20; flow rate 1.0 mL/min; 25 °C; 254 nm; retention time: 34.4 min (minor) and 68.6 min (major).

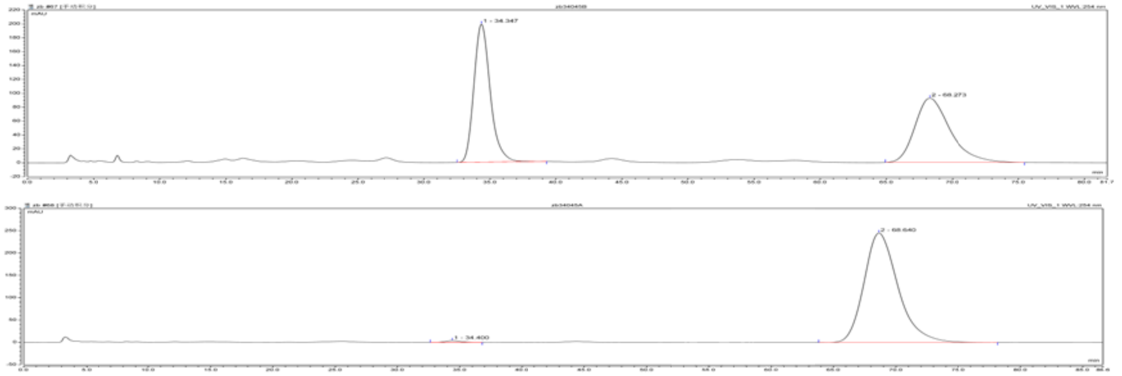

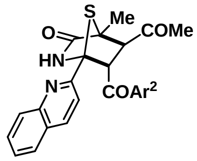

6k: Ar^2 = 2-furyl

White solid, Mp 165.0–166.2 °C; 92% yield; 99% *ee*; $[\alpha]_D^{26}$ –192.1 (*c* 1.36, $CHCl_3$); ^{1}H NMR (300 MHz, $CDCl_3$) δ 8.20 (d, *J* = 8.0 Hz, 1H), 7.83–7.73 (m, 3H), 7.64 (t, *J* = 8.0 Hz, 1H), 7.55–7.50 (m, 2H), 7.27 (s, 1H), 7.03 (d, *J* = 3.6 Hz, 1H), 6.36–6.31 (m, 1H), 5.39 (d, *J* = 5.3 Hz, 1H), 3.64 (d, *J* = 5.3 Hz, 1H), 2.39 (s, 3H), 1.77 (s, 3H); ^{13}C NMR (75 MHz, $CDCl_3$) δ 204.5, 185.3, 176.3, 152.3, 152.2, 146.9, 146.6, 137.5, 130.0, 129.4, 127.6, 127.4, 127.3, 118.2, 118.1, 112.5, 79.2, 66.3, 62.9, 58.3, 32.2, 14.3; HRMS (ESI) m/z 407.1069 (M+H$^+$), calc. for $C_{22}H_{19}N_2O_4S$ 407.1066.

The *ee* was determined by HPLC analysis. CHIRALPAK IA (4.6 mm i.d. x 250 mm); Hexane/2-propanol = 70/30; flow rate 1.0 mL/min; 25 °C; 230 nm; retention time: 24.4 min (minor) and 38.8 min (major).

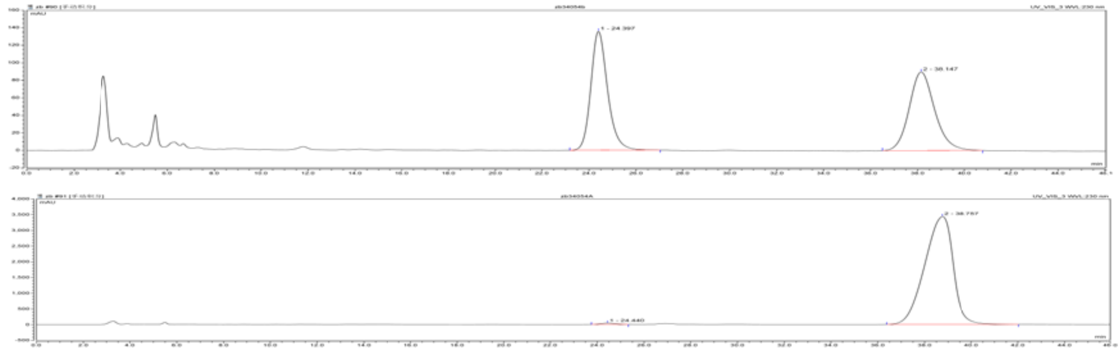

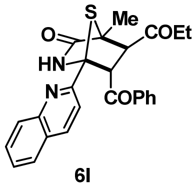

6l

White solid, Mp 158.2–160.0 °C; 90% yield; 97% *ee*; $[\alpha]_D^{20}$ –199.8 (*c* 2.65, $CHCl_3$); ^1H NMR (300 MHz, $CDCl_3$) δ 8.15 (d, *J* = 8.5 Hz, 1H), 7.74–7.70 (m, 3H), 7.64–7.56 (m, 3H), 7.54–7.46 (m, 2H), 7.35 (t, *J* = 7.4 Hz, 1H), 7.22 (d, *J* = 7.4 Hz, 1H), 5.69 (d, *J* = 5.5 Hz, 1H), 3.50 (d, *J* = 5.5 Hz, 1H), 2.71–2.60 (m, 1H), 2.35–2.22 (m, 1H), 1.71 (s, 3H), 1.04 (t, *J* = 7.2 Hz, 3H); ^{13}C NMR (75 MHz, $CDCl_3$) δ 207.9, 198.3, 176.6, 152.2, 146.8, 137.6, 136.9, 133.2, 129.9, 129.3, 128.4, 128.0, 127.6, 127.3, 127.2, 118.2, 79.5, 66.8, 62.3, 57.4, 39.2, 14.2, 7.6; HRMS (ESI) m/z 431.1430 ($M+H^+$), calc. for $C_{25}H_{23}N_2O_3S$ 431.1429.

The *ee* was determined by HPLC analysis. CHIRALPAK IC (4.6 mm i.d. x 250 mm); Hexane/2-propanol = 80/20; flow rate 1.0 mL/min; 25 °C; 254 nm; retention time: 20.0 min (minor) and 22.4 min (major).

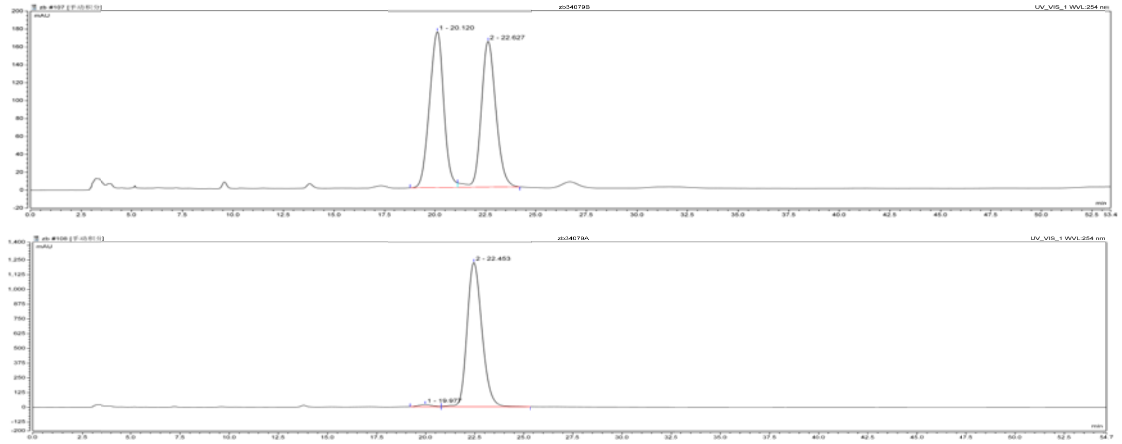

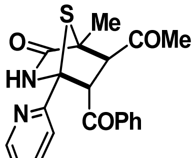

6m

White solid, Mp 138.6–139.1 °C; 87% yield; 98% *ee*; $[\alpha]_D^{23}$ –104.5 (*c* 1.71, $CHCl_3$); ^1H NMR (300 MHz, $CDCl_3$) δ 8.81 (d, *J* = 1.8 Hz, 1H), 8.54 (dd, *J* = 4.8, 1.8 Hz, 1H), 7.87 – 7.83 (m, 1H), 7.64 – 7.62 (m, 3H), 7.55 (t, *J* = 7.6 Hz, 1H), 7.38 (t, *J* = 7.6 Hz, 2H), 7.29 – 7.27 (m, 1H), 5.44 (d, *J* = 5.4 Hz, 1H), 3.35 (d, *J* = 5.4 Hz, 1H), 2.24 (s, 3H), 1.71 (s, 3H); ^{13}C NMR (75 MHz, $CDCl_3$) δ 204.5, 198.3, 177.1, 150.2, 147.3, 136.0, 134.2, 133.9, 130.2, 129.0, 128.0, 123.5, 76.4, 65.6, 61.6, 59.1, 33.0, 14.2; HRMS (ESI) m/z 367.1119 ($M+H^+$), calc. for $C_{20}H_{19}N_2O_3S$ 367.1116.

The *ee* was determined by HPLC analysis. CHIRALPAK IC (4.6 mm i.d. x 250 mm); Hexane/2-propanol = 70/30; flow rate 1.0 mL/min; 25 °C; 254 nm; retention time: 16.9 min (major) and 23.6 min (minor).

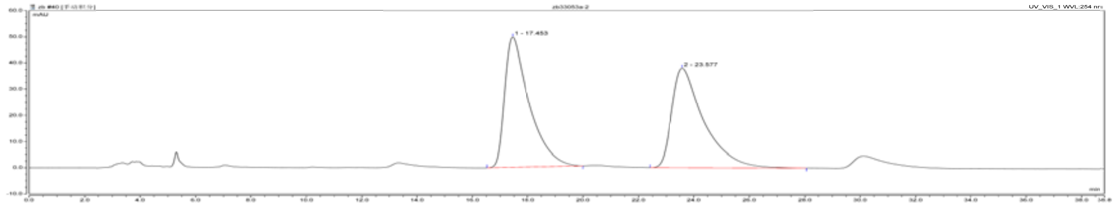

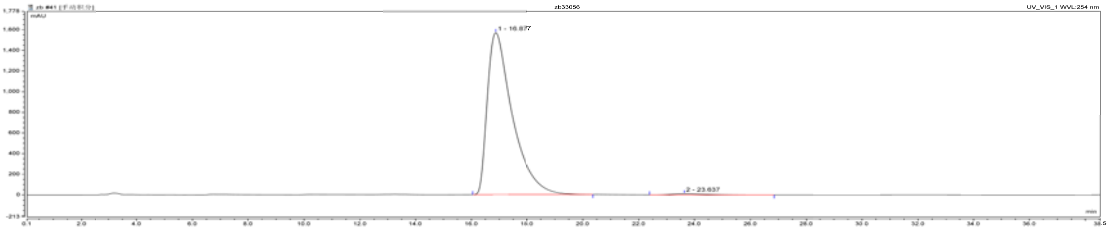

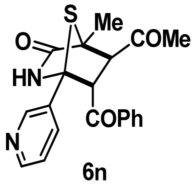

6n

White solid, Mp 120.2–122.1 °C; 86% yield; 98% *ee*; $[\alpha]_D^{24}$ –112.9 (*c* 1.81, $CHCl_3$); ^1H NMR (300 MHz, $CDCl_3$) δ 8.28–8.27 (m, 1H), 7.67–7.61 (m, 1H), 7.59–7.55 (m, 3H), 7.44–7.38 (m, 1H), 7.28 – 7.23 (m, 3H), 7.10–7.06 (m, 1H), 5.50 (d, *J* = 5.4 Hz, 1H), 3.40 (d, *J* = 5.4 Hz, 1H), 2.19 (s, 3H), 1.67 (s, 3H); ^{13}C NMR (75 MHz, $CDCl_3$) δ 204.8, 198.0, 176.6, 152.6, 149.1, 137.1, 136.6, 133.5, 128.6, 128.1, 123.7 121.0, 78.9, 66.2, 62.4, 58.6, 32.8, 14.4; HRMS (ESI) m/z 367.1112 (M+H$^+$), calc. for $C_{20}H_{19}N_2O_3S$ 367.1116.

The *ee* was determined by HPLC analysis. CHIRALPAK IC (4.6 mm i.d. x 250 mm); Hexane/2-propanol = 70/30; flow rate 1.0 mL/min; 25 °C; 254 nm; retention time: 18.9 min (major) and 33.6 min (minor).

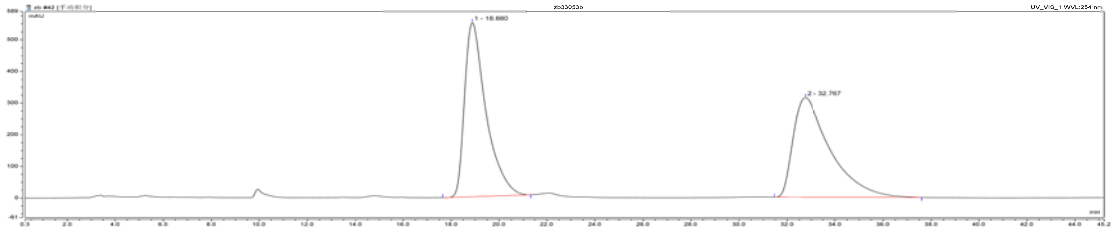

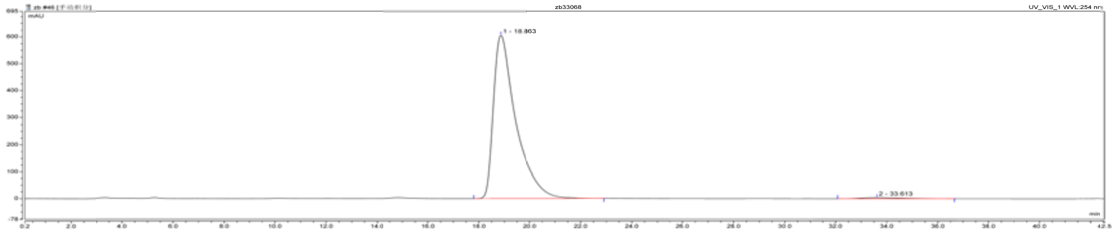

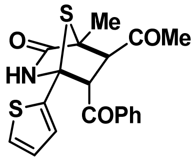

6o

White solid, Mp 134.4–135.6 °C; 70% yield; 99% *ee*; $[\alpha]_D^{25}$ –99.7 (*c* 0.48, $CHCl_3$); ^1H NMR (300 MHz, $CDCl_3$) δ 7.74 (d, *J* = 7.5 Hz, 2H), 7.59 (t, *J* = 7.5 Hz, 1H), 7.44 (t, *J* = 7.5 Hz, 2H), 7.28 (dd, *J* = 5.1, 2.4 Hz, 1H), 7.11–7.10 (m, 1H), 6.98 (s, 1H), 6.90 (dd, *J* = 5.1, 2.4 Hz, 1H), 5.43 (d, *J* = 5.5 Hz, 1H), 3.26 (d, *J* = 5.5 Hz, 1H), 2.21 (s, 3H), 1.70 (s, 3H); ^{13}C NMR (75 MHz, $CDCl_3$) δ 204.2, 198.6, 176.7, 136.9, 136.3, 134.2, 129.1, 128.2, 127.2, 126.6, 126.3, 75.3, 66.2, 61.8, 59.8, 32.9, 14.3; HRMS (ESI) m/z 372.0733 (M+H$^+$), calc. for $C_{19}H_{18}NO_3S_2$ 372.0728.

The *ee* was determined by HPLC analysis. CHIRALPAK IA (4.6 mm i.d. x 250 mm); Hexane/2-propanol = 80/20; flow rate 1.0 mL/min; 25 °C; 254 nm; retention time: 24.4 min (minor) and 25.9 min (major).

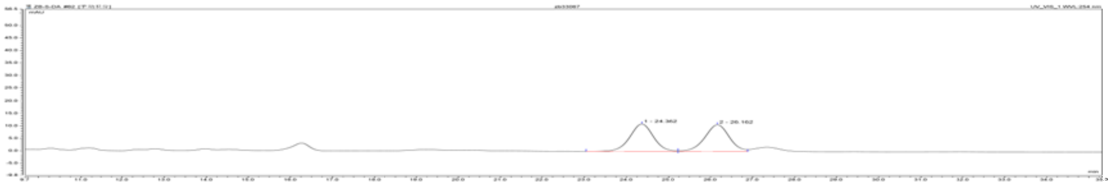

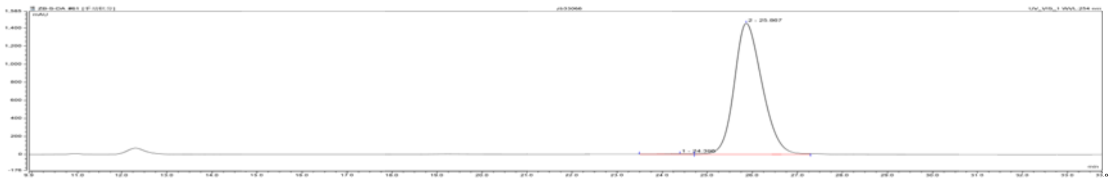

White solid, Mp 155.2–156.7 °C; 75% yield; 99% *ee*; $[\alpha]_D^{26}$ –94.3 (*c* 0.74, $CHCl_3$); ^{1}H NMR (300 MHz, $CDCl_3$) δ 7.72 (d, *J* = 7.6 Hz, 2H), 7.56 (t, *J* = 7.6 Hz, 1H), 7.42 (t, *J* = 7.6 Hz, 2H), 7.29 (s, 1H), 6.91 (s, 1H), 6.45 (d, *J* = 3.3 Hz, 1H), 6.32–6.30 (m, 1H), 5.42 (d, *J* = 5.5 Hz, 1H), 3.32 (d, *J* = 5.5 Hz, 1H), 2.22 (s, 3H), 1.70 (s, 3H); ^{13}C NMR (75 MHz, $CDCl_3$) δ 204.4, 198.1, 176.4, 146.5, 143.0, 136.2, 134.0, 128.9, 128.2, 111.1, 109.0, 72.9, 65.6, 60.8, 58.7, 32.8, 14.3; HRMS (ESI) m/z 356.0958 (M+H$^+$), calc. for $C_{19}H_{18}NO_4S$ 356.0957.

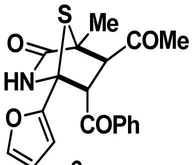

6p

The *ee* was determined by HPLC analysis. CHIRALPAK IA (4.6 mm i.d. x 250 mm); Hexane/2-propanol = 90/10; flow rate 1.0 mL/min; 25 °C; 254 nm; retention time: 50.3 min (major) and 54.5 min (minor).

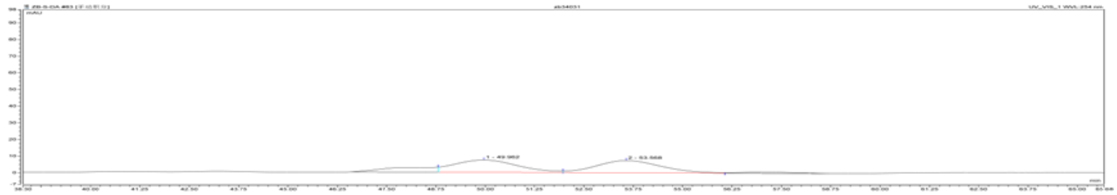

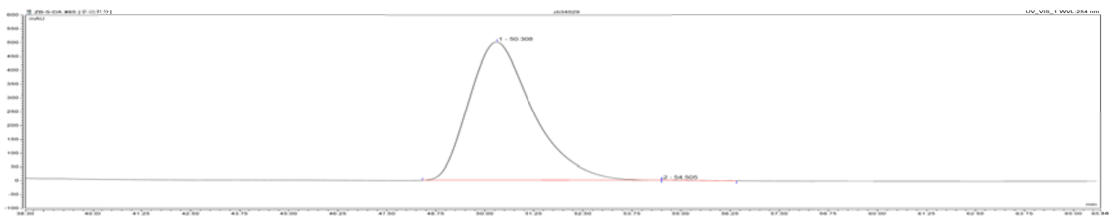

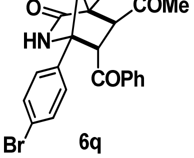

6q

White solid, Mp 140.0–141.8 °C; 54% yield; 99% *ee*; $[\alpha]_D^{20}$ –111.6 (*c* 1.61, $CHCl_3$); ^{1}H NMR (300 MHz, $CDCl_3$) δ 7.68–7.63 (m, 2H), 7.59–7.54 (m, 1H), 7.48–7.45 (m, 2H), 7.41–7.38 (m, 4H), 7.29 (s, 1H), 5.40 (d, *J* = 5.4 Hz, 1H), 3.31 (d, *J* = 5.4 Hz, 1H), 2.23 (s, 3H), 1.69 (s, 3H); ^{13}C NMR (75 MHz, $CDCl_3$) δ 204.6, 198.4, 177.2, 136.2, 134.2, 133.0, 132.1, 129.0, 128., 127.6, 123.2, 77.7, 65.7, 61.6, 59.4, 33.04, 14.3; HRMS (ESI) m/z 444.0273 (M+H$^+$), calc. for $C_{21}H_{19}BrNO_3S$ 444.0269.

The *ee* was determined by HPLC analysis. CHIRALPAK IC (4.6 mm i.d. x 250 mm); Hexane/2-propanol = 80/20; flow rate 1.0 mL/min; 25 °C; 254 nm; retention time: 8.7 min (minor) and 9.6 min (major).

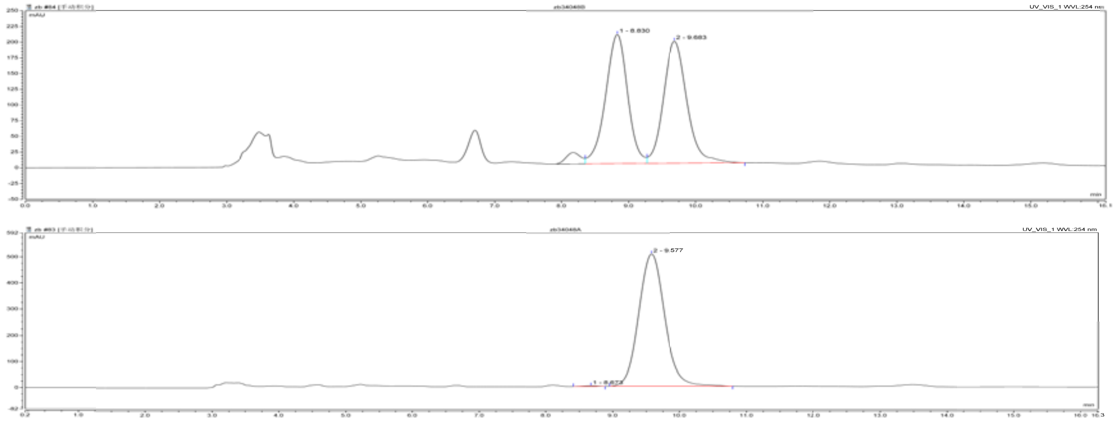

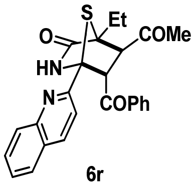

6r

Weak yellow solid, Mp 128.1–129.6 °C; 72% yield; 97% *ee*; $[\alpha]_D^{26}$ –102.5 (*c* 1.06, $CHCl_3$); ^1H NMR (300 MHz, $CDCl_3$) δ 8.16 (d, *J* = 8.5 Hz, 1H), 7.77–7.66 (m, 5H), 7.62–7.56 (m, 1H), 7.52–7.46 (m, 1H), 7.43 (s, 1H), 7.40–7.35 (m, 1H), 7.28–7.23 (m, 1H), 5.70 (d, *J* = 5.5 Hz, 1H), 3.51 (d, *J* = 5.5 Hz, 1H), 2.55–2.42 (m, 1H), 2.27 (s, 3H), 1.92–1.80 (m, 1H), 1.15 (t, *J* = 7.2 Hz, 3H); ^{13}C NMR (75 MHz, $CDCl_3$) δ 205.3, 198.5, 175.8, 152.4, 146.8, 137.6, 137.0, 133.2, 130.0, 129.3, 128.4, 128.1, 127.6, 127.4, 127.3, 118.3, 79.0, 74.0, 61.9, 58.6, 33.2, 22.1, 12.1; HRMS (ESI) m/z 431.1431 $(M+H^+)$, calc. for $C_{25}H_{23}N_2O_3S$ 431.1429.

The *ee* was determined by HPLC analysis. CHIRALPAK IA (4.6 mm i.d. x 250 mm); Hexane/2-propanol = 80/20; flow rate 1.0 mL/min; 25 °C; 254 nm; retention time: 25.8 min (minor) and 46.4 min (major).

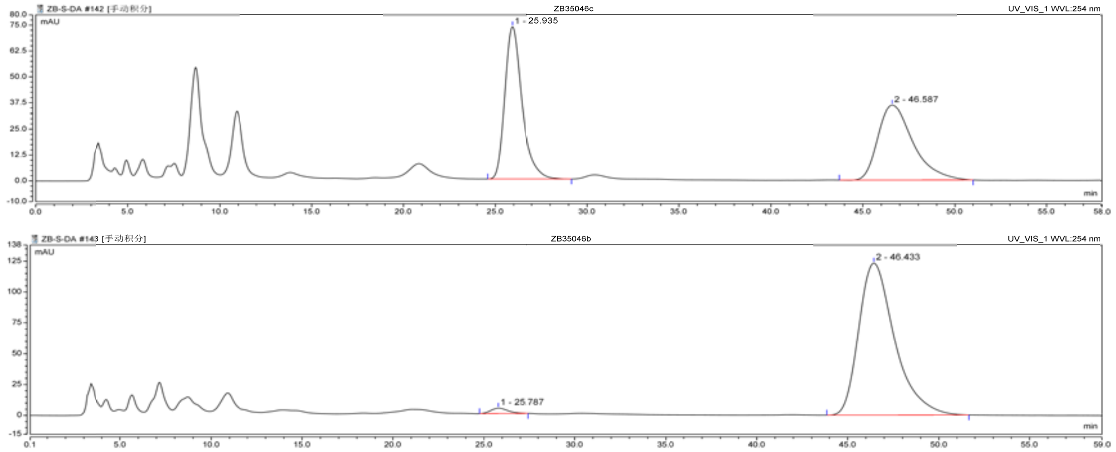

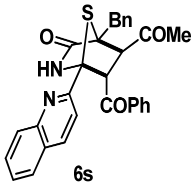

6s

Weak yellow solid, Mp 137.3–138.8 °C; 90% yield; 98% *ee*; $[\alpha]_D^{26}$ –83.3 (*c* 4.68, $CHCl_3$); ^1H NMR (300 MHz, $CDCl_3$) δ 8.06 (d, *J* = 8.5 Hz, 1H), 7.69–7.62 (m, 6H), 7.58–7.53 (m, 1H), 7.48–7.42 (m, 1H), 7.39–7.34 (m, 3H), 7.30–7.22 (m, 5H), 5.66 (d, *J* = 5.6 Hz, 1H), 3.87 (d, *J* = 13.5 Hz, 1H), 3.67 (d, *J* = 5.6 Hz, 1H), 3.02 (d, *J* = 13.5 Hz, 1H), 2.29 (s, 3H); ^{13}C NMR (75 MHz, $CDCl_3$) δ 205.6, 198.1, 175.6,

152.1, 146.6, 137.4, 137.2, 136.9, 133.2, 129.8, 129.1, 128.4, 128.3, 128.0, 127.5, 127.3, 127.2, 118.3, 79.1, 74.1, 62.5, 58.2, 33.8, 33.3; HRMS (ESI) m/z 493.1583 (M+H$^+$), calc. for $C_{30}H_{25}N_2O_3S$ 493.1586.

The *ee* was determined by HPLC analysis. CHIRALPAK IC (4.6 mm i.d. x 250 mm); Hexane/2-propanol = 80/20; flow rate 1.0 mL/min; 25 ^{o}C; 254 nm; retention time: 21.4 min (minor) and 31.8 min (major).

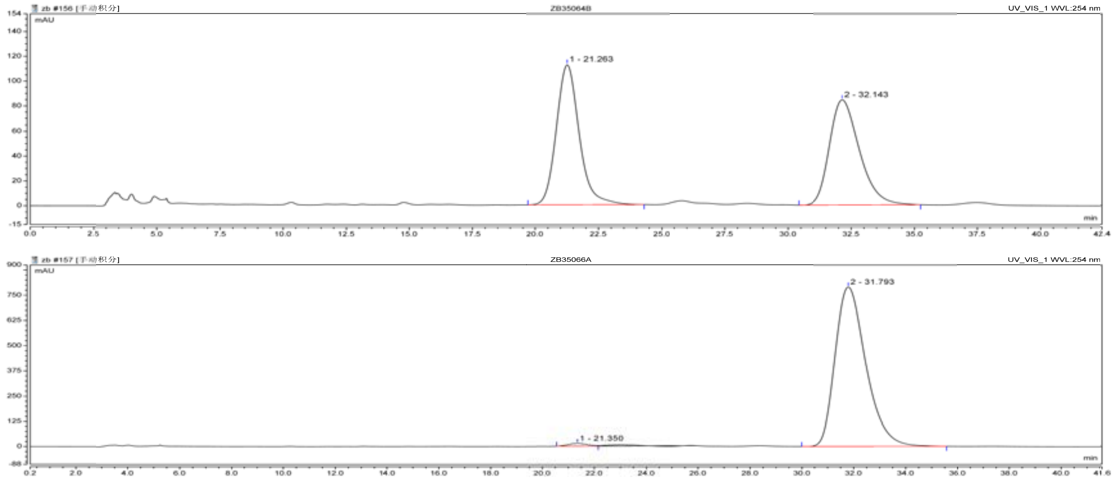

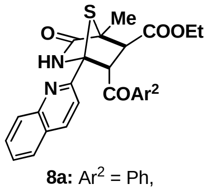

8a: Ar^2 = Ph,

White solid, Mp 141.8–143.2 ^{o}C; 87% yield; 95% *ee*; $[\alpha]_D^{26}$ −138.4 (*c* 1.95, $CHCl_3$); ^{1}H NMR (300 MHz, $CDCl_3$) δ 8.15 (d, *J* = 8.5 Hz, 1H), 7.74–7.66 (m, 5H), 7.62 – 7.56 (m, 1H), 7.52–7.44 (m, 2H), 7.36 (t, *J* = 7.4 Hz, 1H), 7.22 (d, *J* = 7.4 Hz, 1H), 5.76 (d, *J* = 5.4 Hz, 1H), 4.36–4.22 (m, 2H), 3.34 (d, *J* = 5.4 Hz, 1H), 1.76 (s, 3H), 1.29 (t, *J* = 7.1 Hz, 3H); ^{13}C NMR (75 MHz, $CDCl_3$) δ 198.0, 176.6, 170.6, 152.2, 146.8, 137.6, 137.1, 133.1, 129.9, 129.3, 128.3, 128.1, 127.6, 127.4, 127.3, 118.3, 79.9, 66.5, 62.0, 61.8, 53.3, 14.3, 14.2; HRMS (ESI) m/z 447.1383 (M+H$^+$), calc. for $C_{25}H_{23}N_2O_4S$ 447.1379.

The *ee* was determined by HPLC analysis. CHIRALPAK IA (4.6 mm i.d. x 250 mm); Hexane/2-propanol = 80/20; flow rate 1.0 mL/min; 25 ^{o}C; 230 nm; retention time: 14.9 min (minor) and 18.1 min (major).

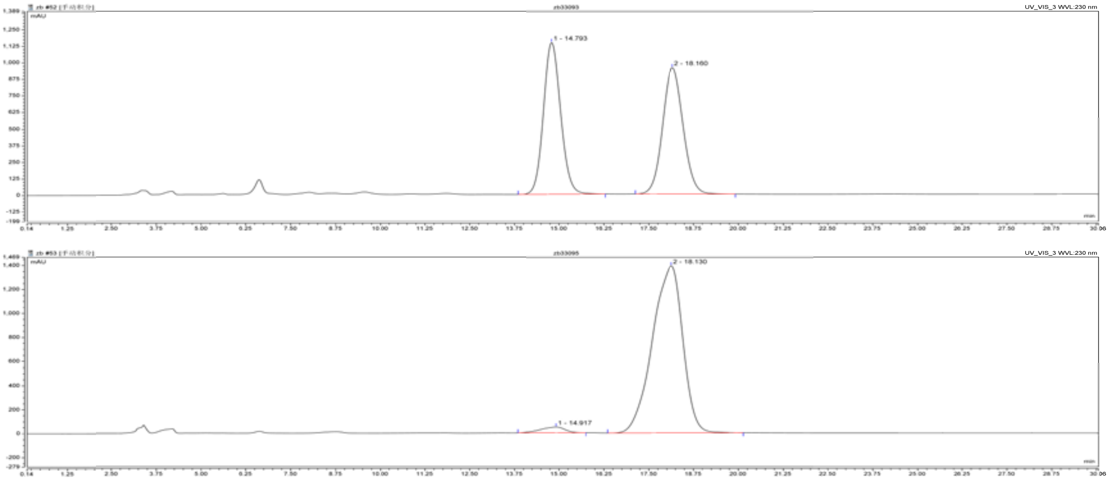

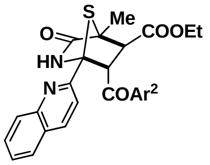

8b: Ar^2 = 4-BrPh

White solid, Mp 157.3–159.0 °C; 97% yield; 97% *ee*; $[\alpha]_D^{20}$ –170.7 (*c* 4.98, $CHCl_3$); ^{1}H NMR (300 MHz, $CDCl_3$) δ 8.16 (d, *J* = 8.4 Hz, 1H), 7.73 (t, *J* = 8.4 Hz, 2H), 7.65–7.57 (m, 5H), 7.53–7.47 (m, 1H), 7.35 (d, *J* = 8.4 Hz, 2H), 5.69 (d, *J* = 5.4 Hz, 1H), 4.36 –4.19 (m, 2H), 3.35 (d, *J* = 5.4 Hz, 1H), 1.75 (s, 3H), 1.29 (t, *J* = 7.1 Hz, 3H); ^{13}C NMR (75 MHz, $CDCl_3$) δ 197.1, 176.61, 170.51, 152.1, 146.7, 137.7, 135.8, 131.5, 130.1, 129.6, 129.1, 128.4, 127.6, 127.4, 118.4, 80.1, 66.6, 61.8, 61.7, 53.2, 14.3, 14.2; HRMS (ESI) m/z 525.0486 (M+H$^+$), calc. for $C_{25}H_{22}BrN_2O_4S$ 525.0484.

The *ee* was determined by HPLC analysis. CHIRALPAK IC (4.6 mm i.d. x 250 mm); Hexane/2-propanol = 90/10; flow rate 1.2 mL/min; 25 °C; 254 nm; retention time: 39.0min (major) and 42.1 min (minor).

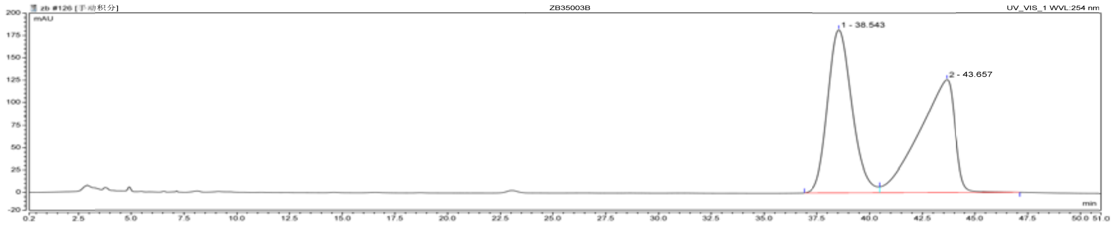

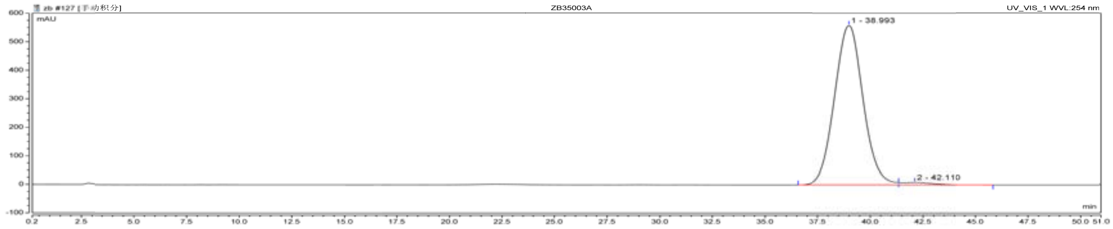

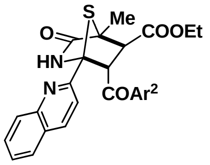

8c: Ar^2 = 3,4-Cl_2Ph

White solid, Mp 155.4–156.5 °C; 96% yield; 98% *ee*; $[\alpha]_D^{20}$ –124.7 (*c* 2.76, $CHCl_3$); ^{1}H NMR (300 MHz, $CDCl_3$) δ 8.20 (s, 1H), 7.78–7.74 (m, 1H), 7.72–7.70 (m, 1H), 7.63 (d, *J* = 3.6 Hz, 2H), 7.58–7.49 (m, 3H), 7.27–7.24 (m, 1H), 5.59 (d, *J* = 5.2 Hz, 1H), 4.30 (qq, *J* = 10.8, 7.1 Hz, 2H), 3.43 (d, *J* = 5.2 Hz, 1H), 1.77 (s, 3H), 1.32 (t, *J* = 7.1 Hz, 3H); ^{13}C NMR (75 MHz, $CDCl_3$) δ 195.6, 176.5, 170.4, 151.8, 146.6, 137.8, 137.6, 136.6, 132.9, 130.3, 130.2, 130.0, 129.0, 127.6, 127.5, 127.1, 118.3, 80.0, 66.6, 62.4, 62.0, 52.9, 14.3, 14.2; HRMS (ESI) m/z 515.0602 (M+H$^+$), calc. for $C_{25}H_{21}Cl_2N_2O_4S$ 515.0599.

The *ee* was determined by HPLC analysis. CHIRALPAK IC (4.6 mm i.d. x 250 mm); Hexane/2-propanol = 70/30; flow rate 1.0 mL/min; 25 °C; 254 nm; retention time: 12.1 min (major) and 14.4 min (minor).

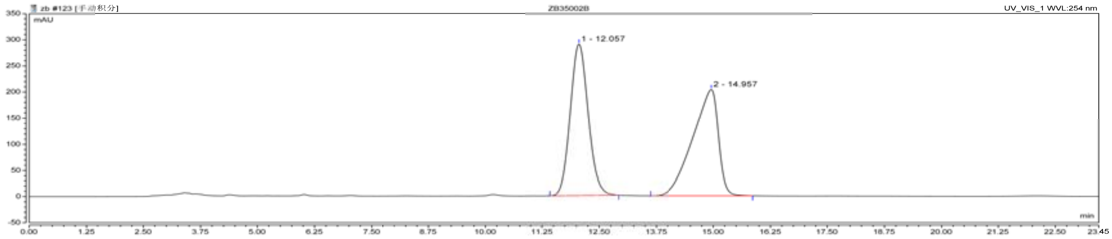

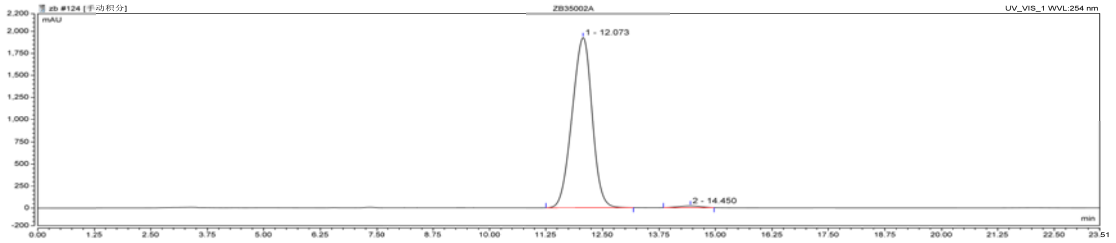

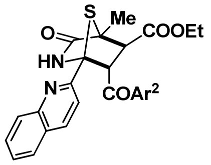

8d: Ar^2 = 4-MeOPh

Weak yellow solid, Mp 122.2–124.0 °C; 90% yield; 98% *ee*; $[\alpha]_D^{26}$ –173.9 (*c* 4.61, $CHCl_3$); ^{1}H NMR (300 MHz, $CDCl_3$) δ 8.16 (d, *J* = 8.7 Hz, 1H), 7.75–7.71 (m, 5H), 7.62–7.57 (m, 1H), 7.2–7.46 (m, 2H), 6.70 (d, *J* = 8.7 Hz, 2H), 5.71 (d, *J* = 5.4 Hz, 1H), 4.37–4.20 (m, 2H), 3.74 (s, 3H), 3.32 (d, *J* = 5.4 Hz, 1H), 1.75 (s, 3H), 1.30 (t, *J* = 7.1 Hz, 3H); ^{13}C NMR (75 MHz, $CDCl_3$) δ 196.2, 176.7, 170.7, 163.5, 152.4, 146.8, 137.5, 130.6, 130.0, 129.9, 129.4, 127.6, 127.3, 127.2, 118.3, 113.5, 80.0, 66.5, 61.7, 61.5, 55.4, 53.5, 14.3, 14.2; HRMS (ESI) m/z 477.1481 (M+H$^+$), calc. for $C_{26}H_{25}N_2O_5S$ 477.1484.

The *ee* was determined by HPLC analysis. CHIRALPAK IA (4.6 mm i.d. x 250 mm); Hexane/2-propanol = 80/20; flow rate 1.0 mL/min; 25 °C; 254 nm; retention time: 20.3 min (minor) and 23.7 min (major).

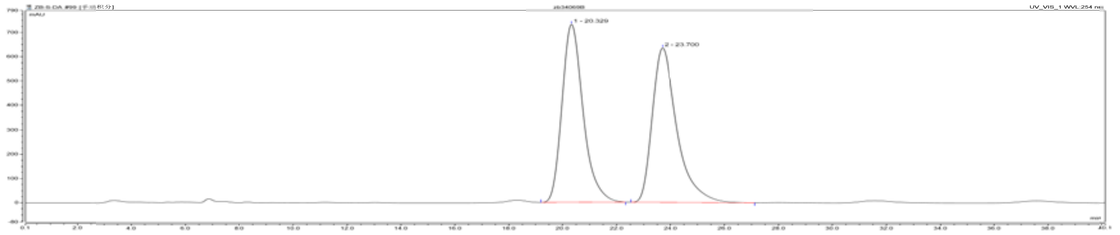

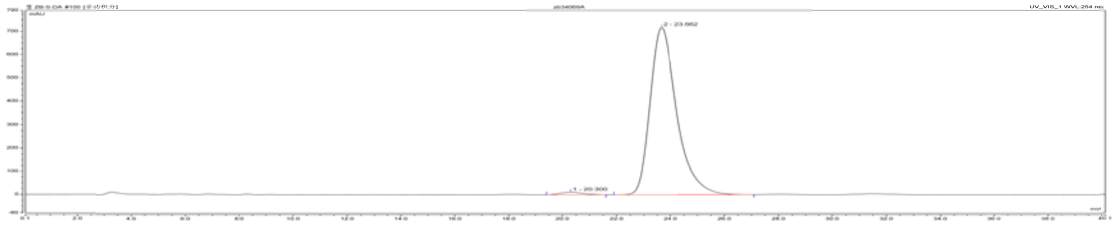

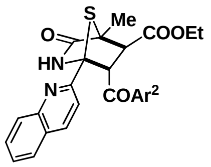

8e: Ar^2 = 2-thienyl

White solid, Mp 137.5–138.7 °C; 97% yield; 99% *ee*; $[\alpha]_D^{26}$ –132.0 (*c* 4.67, $CHCl_3$); ^{1}H NMR (300 MHz, $CDCl_3$) δ 8.18 (d, *J* = 8.5 Hz, 1H), 7.74–7.71 (m, 3H), 7.62–7.55 (m, 3H), 7.51–7.46 (m, 2H), 6.90–6.87 (m, 1H), 5.57 (d, *J* = 5.3 Hz, 1H), 4.37–4.20 (m, 2H), 3.41 (d, *J* = 5.3 Hz, 1H), 1.76 (s, 3H), 1.30 (t, *J* = 7.1 Hz, 3H); ^{113}C NMR (75 MHz, $CDCl_3$) δ 190.0, 176.6, 170.5, 152.1, 146.8, 144.3, 137.6, 134.7, 132.6, 129.9, 129.3, 127.9, 127.6, 127.4, 127.3, 118.2, 80.0, 66.5, 62.7, 61.8, 53.6, 14.2,(two peaks); HRMS (ESI) m/z 453.0940 (M+H$^+$), calc. for $C_{23}H_{21}N_2O_4S_2$ 453.0943.

The *ee* was determined by HPLC analysis. CHIRALPAK IC (4.6 mm i.d. x 250 mm); Hexane/2-propanol = 70/30; flow rate 1.0 mL/min; 25 °C; 254 nm; retention time: 23.2 min (major) and 26.4 min (minor).

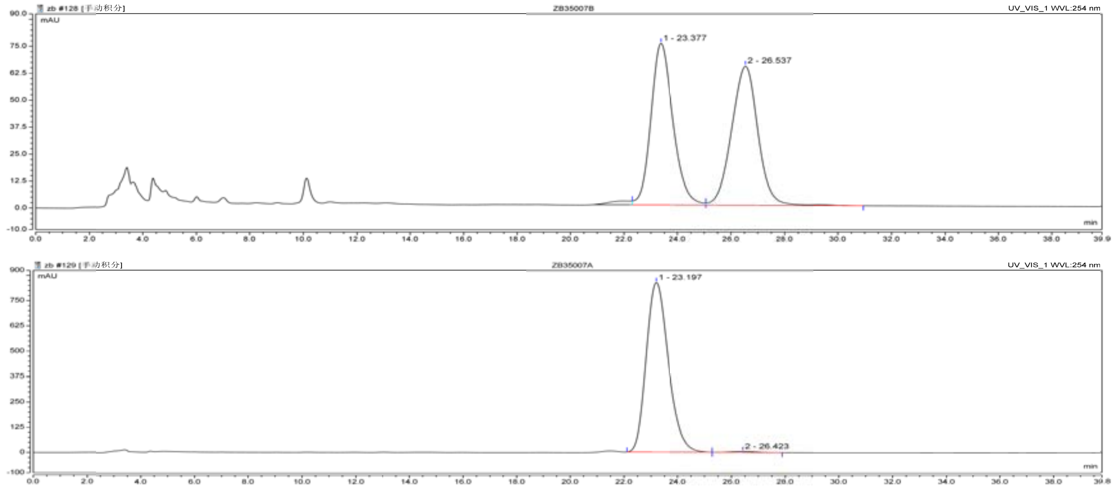

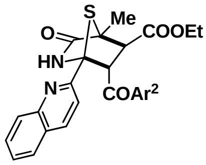

8f: Ar² = 2-furyl

White solid, Mp 141.8–143.1 °C; 96% yield; 98% *ee*; $[\alpha]_D^{26}$ –186.1 (*c* 4.29, $CHCl_3$); ^1H NMR (300 MHz, $CDCl_3$) δ 8.19 (d, *J* = 8.5 Hz, 1H), 7.79–7.73 (m, 3H), 7.65–7.60 (m, 1H), 7.54–7.49 (m, 2H), 7.25 (s, 1H), 7.06 (d, *J* = 3.6 Hz, 1H), 6.29 (dd, *J* = 3.6, 1.7 Hz, 1H), 5.45 (d, *J* = 5.2 Hz, 1H), 4.38–4.22 (m, 2H), 3.46 (d, *J* = 5.2 Hz, 1H), 1.76 (s, 3H), 1.32 (t, *J* = 7.1 Hz, 3H); ^{13}C NMR (75 MHz, $CDCl_3$) δ 185.0, 176.3, 170.5, 152.3, 152.2, 146.8, 146.6, 137.4, 130.0, 129.4, 127.6, 128.4, 127.3, 118.2, 112.3, 79.5, 66.3, 63.1, 61.7, 53.0, 14.2; HRMS (ESI) m/z 437.1175 (M+H$^+$), calc. for $C_{23}H_{21}N_2O_5S$ 437.1171.

The *ee* was determined by HPLC analysis. CHIRALPAK IA (4.6 mm i.d. x 250 mm); Hexane/2-propanol = 80/20; flow rate 1.0 mL/min; 25 °C; 254 nm; retention time: 30.7 min (minor) and 35.7 min (major).

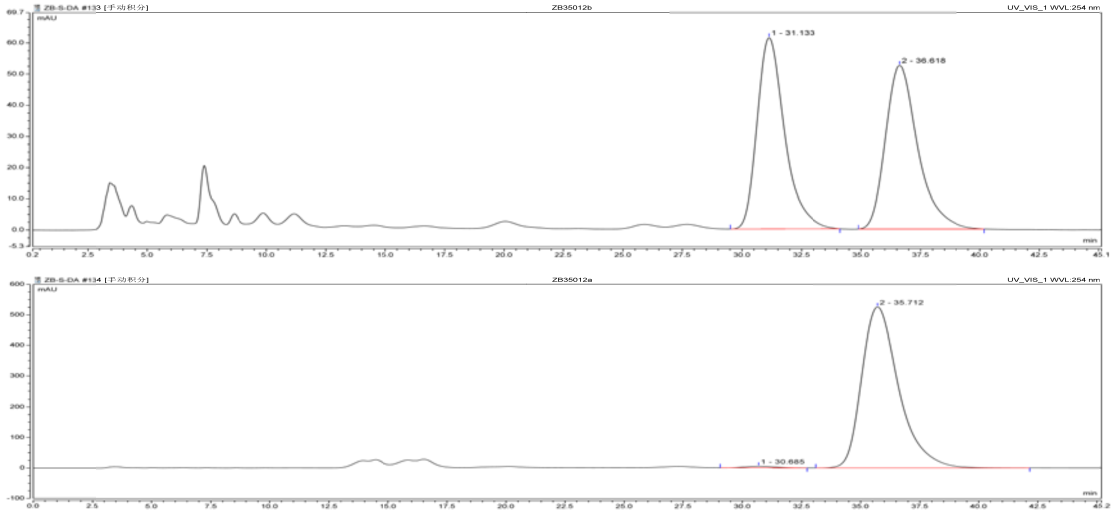

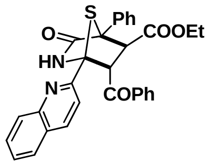

8g

Pale yellow solid, Mp 158.4–160.1 °C; 67% yield; 90% *ee*; $[\alpha]_D^{28}$ –205.7 (*c* 1.50, $CHCl_3$); ^1H NMR (300 MHz, $CDCl_3$) δ 8.21 (d, *J* = 8.5 Hz, 1H), 7.83 (d, *J* = 8.5 Hz, 1H), 7.78–7.70 (m, 4H), 7.64–7.49 (m, 5H), 7.43–7.35 (m, 4H), 7.28 (s, 1H), 7.23 (s, 1H), 5.86 (d, *J* = 5.4 Hz, 1H), 3.80 (d, *J* = 5.4 Hz, 1H), 3.82–3.62 (m, 2H), 0.74 (t, *J* = 7.1 Hz, 3H); ^{13}C NMR (75 MHz, $CDCl_3$) δ 197.7, 174.5, 170.1, 152.1,

146.8, 137.7, 137.0, 133.2, 131.4, 130.0, 129.3, 128.7, 128.6, 128.4, 128.2, 128.0, 127.6, 127.4, 118.3, 78.4, 74.3, 62.5, 61.3, 54.7, 13.4; HRMS (ESI) m/z 509.1538 (M+H$^+$), calc. for $C_{30}H_{25}N_2O_4S$ 509.1535.

The *ee* was determined by HPLC analysis. CHIRALPAK IC (4.6 mm i.d. x 250 mm); Hexane/2-propanol = 60/40; flow rate 1.0 mL/min; 25 °C; 254 nm; retention time: 19.3 min (minor) and 50.0 min (major).

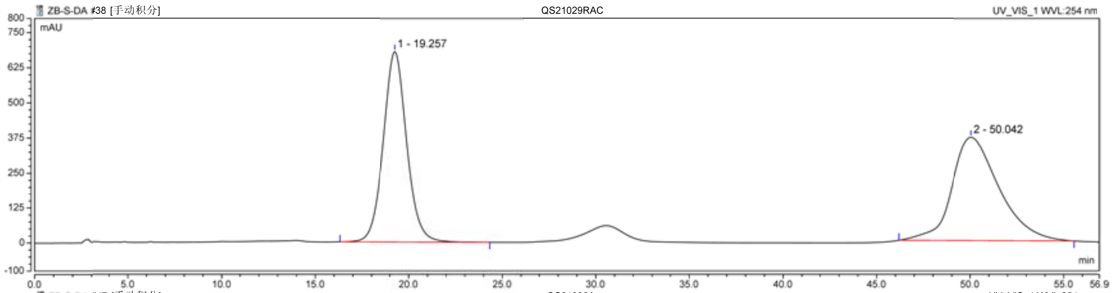

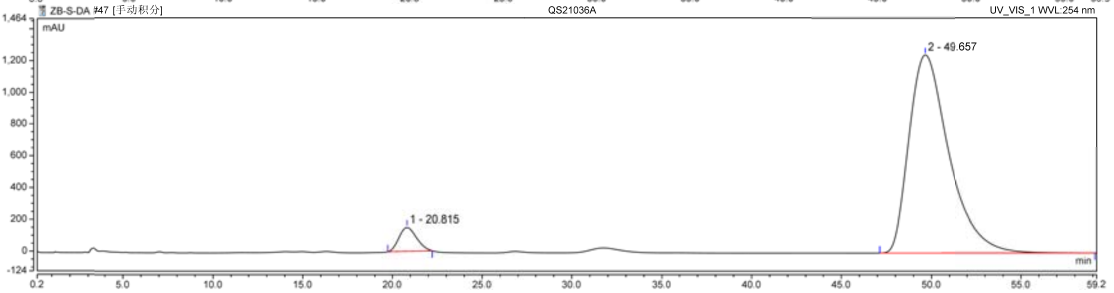

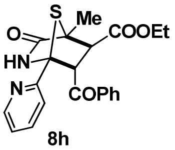

8h

White solid, Mp 125.5–126.8 °C; 95% yield; 97% *ee*; $[\alpha]_D^{26}$ –134.7 (*c* 3.72, $CHCl_3$); ^{1}H NMR (300 MHz, $CDCl_3$) δ 8.80 (d, *J* = 2.0 Hz, 1H), 8.54–8.52 (m, 1H), 7.95 (s, 1H), 7.88–7.84 (m, 1H), 7.68 (d, *J* = 7.4 Hz, 2H), 7.50 (t, *J* = 7.4 Hz, 1H), 7.35–7.24 (m, 3H), 7.24 (s, 0H), 5.43 (d, *J* = 5.4 Hz, 1H), 4.30 (q, *J* = 7.1 Hz, 2H), 3.22 (d, *J* = 5.4 Hz, 1H), 1.68 (s, 3H), 1.28 (t, *J* = 7.1 Hz, 3H); ^{13}C NMR (75 MHz, $CDCl_3$) δ 197.7, 177.3, 170.5, 150.1, 147.2, 136.1, 134.0, 133.9, 130.2, 128.8, 128.2, 123.5, 76.7, 65.4, 62.0, 54.0, 14.2; HRMS (ESI) m/z 397.1221 (M+H$^+$), calc. for $C_{21}H_{21}N_2O_4S$ 397.1222.

The *ee* was determined by HPLC analysis. CHIRALPAK IC (4.6 mm i.d. x 250 mm); Hexane/2-propanol = 70/30; flow rate 1.0 mL/min; 25 °C; 254 nm; retention time: 14.8 min (major) and 24.5 min (minor).

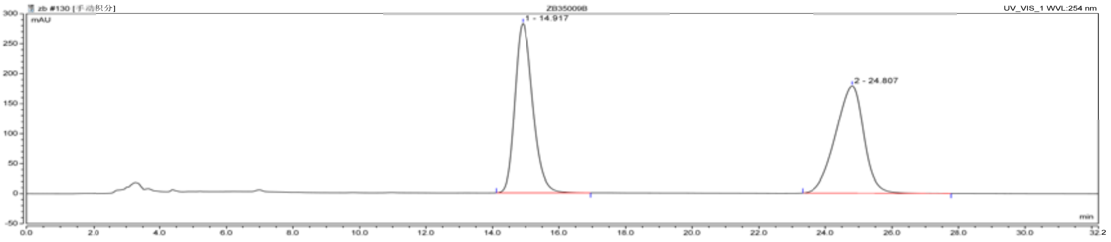

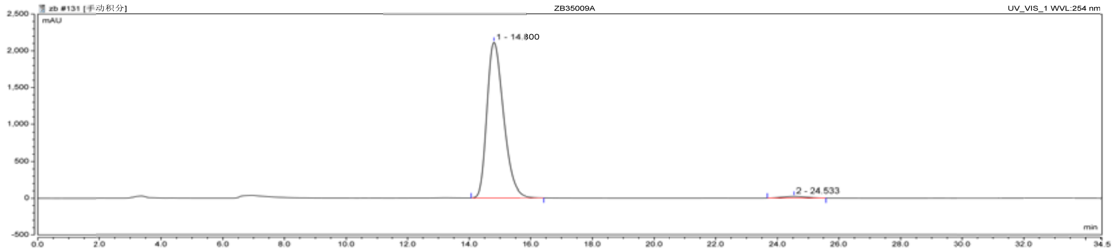

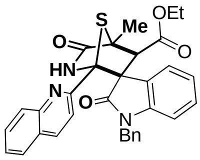

10a

Pale yellow solid, Mp 102.2–103.8 °C; 96% yield; 95% *ee*; $[\alpha]_D^{24}$ –348.8 (*c* 0.05, $CHCl_3$); ^1H NMR (300 MHz, $CDCl_3$) δ 7.95–7.91 (m, 3H), 7.79 (d, *J* = 8.5 Hz, 1H), 7.74–7.68 (m, 2H), 7.58–7.52 (m, 1H), 7.05–6.88 (m, 4H), 6.84–6.75 (m, 4H), 6.25–6.23 (m, 1H), 4.74 (s, 2H), 3.76 (s, 1H), 3.74–3.51 (m, 2H), 1.98 (s, 3H), 0.53 (t, *J* = 7.2 Hz, 3H); ^{13}C NMR (75 MHz, $CDCl_3$) δ 176.2, 174.2, 167.9, 150.4, 146.6, 143.1, 136.2, 135.0, 130.2, 129.6, 129.4, 128.4, 127.5, 127.4, 127.3, 126.5, 126.4, 126.0, 122.0, 118.9, 108.7, 82.7, 67.8, 63.0, 60.6, 60.2, 44.0, 13.3, 13.1; HRMS (ESI) m/z 550.1803 ($M+H^+$), calc. for $C_{32}H_{28}N_3O_4S$ 550.1801.

The *ee* was determined by HPLC analysis. CHIRALPAK IC (4.6 mm i.d. x 250 mm); Hexane/2-propanol = 70/30; flow rate 1.0 mL/min; 25 °C; 254 nm; retention time: 36.8 min (minor) and 42.1min (major).

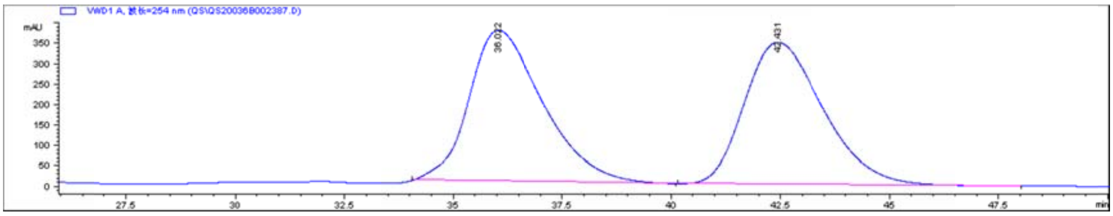

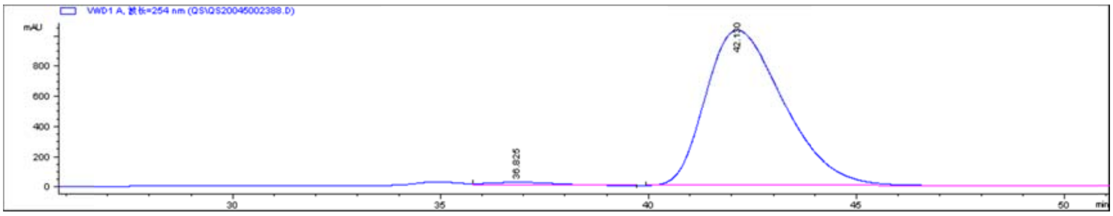

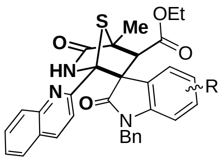

10b: R = 5-F

Pale yellow solid, Mp 130.4–132.2 °C; 98% yield; 94% *ee*; $[\alpha]_D^{24}$ –138.0 (*c* 0.05, $CHCl_3$); ^1H NMR (300 MHz, $CDCl_3$) δ 7.89 (t, *J* = 9.0 Hz, 3H), 7.77–7.69 (m, 3H), 7.59–7.54 (m, 1H), 7.06 (t, *J* = 7.2 Hz, 1H), 6.98 (d, *J* = 8.6 Hz, 1H), 6.88–6.78 (m, 4H), 6.69 (td, *J* = 8.6, 3.5 Hz, 1H), 6.16 (dd, *J* = 8.6, 3.5 Hz, 1H), 4.82 (d, *J* = 15.8 Hz, 1H), 4.69 (d, *J* = 15.8 Hz, 1H), 3.78–3.60 (m, 3H), 1.97 (s, 3H), 0.59 (t, *J* = 7.2 Hz, 3H); ^{13}C NMR (75 MHz, $CDCl_3$) δ 176.1, 174.1, 167.6, 160.0, 156.8, 150.2, 146.6, 139.1, 139.0, 136.6, 134.7, 130.3, 129.5, 128.4, 127.8, 127.7, 127.6, 127.5, 127.4, 126.5, 118.7, 115.7, 115.4, 114.7, 114.3, 109.2, 109.0, 82.8, 67.8, 62.9, 60.8, 60.2, 44.2, 13.2, 13.1; HRMS (ESI) m/z 590.1529 ($M+Na^+$), calc. for $C_{32}H_{26}FN_3O_4SNa$ 590.1526.

The *ee* was determined by HPLC analysis. CHIRALPAK ID-3 (4.6 mm i.d. x 250 mm); Hexane/2-propanol = 80/20; flow rate 1.0 mL/min; 25 °C; 254 nm; retention time: 51.7 min (major) and 60.1 min (minor).

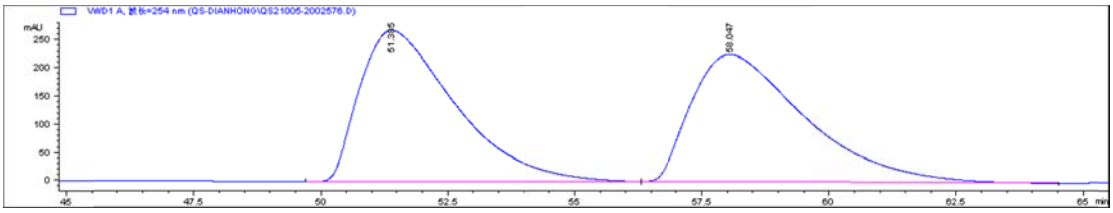

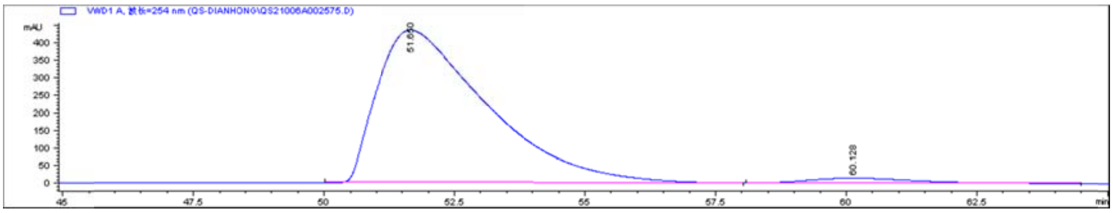

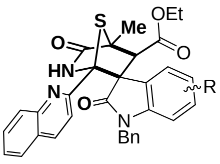

10c: R = 5-Cl

Pale yellow solid, Mp 155.0–156.3 °C; 97% yield; 92% *ee*; $[\alpha]_D^{24}$ –140.5 (*c* 0.05, $CHCl_3$); ^1H NMR (300 MHz, $CDCl_3$) δ 7.91–7.85 (m, 4H), 7.72 (t, *J* = 7.4 Hz, 2H), 7.59–7.54 (m, 1H), 7.06 (t, *J* = 7.4 Hz, 1H), 7.00–6.94 (m, 2H), 6.88–6.78 (m, 4H), 6.17 (d, *J* = 8.3 Hz, 1H), 4.82 (d, *J* = 15.8 Hz, 1H), 4.69 (d, *J* = 15.8 Hz, 1H), 3.82 – 3.59 (m, 3H), 1.97 (s, 3H), 0.61 (t, *J* = 7.1 Hz, 3H); ^{13}C NMR (75 MHz, $CDCl_3$) δ 176.1, 173.9, 167.6, 150.2, 146.6, 141.6, 136.7, 134.6, 130.4, 129.5, 129.2, 128.5, 127.9, 127.6, 127.5, 126.5, 118.8, 109.6, 82.8, 67.6, 63.0, 60.9, 60.3, 44.1, 13.3, 13.1; HRMS (ESI) m/z 584.1415 $(M+H^+)$, calc. for $C_{32}H_{27}ClN_3O_4S$ 584.1411.

The *ee* was determined by HPLC analysis. CHIRALPAK ID-3 (4.6 mm i.d. x 250 mm); Hexane/2-propanol = 70/30; flow rate 1.0 mL/min; 25 °C; 254 nm; retention time: 54.0 min (major) and 66.0 min (minor).

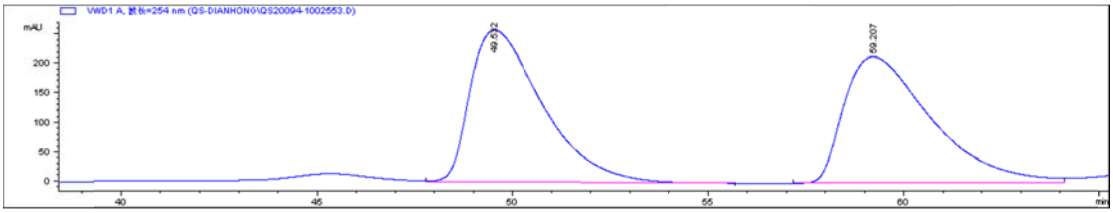

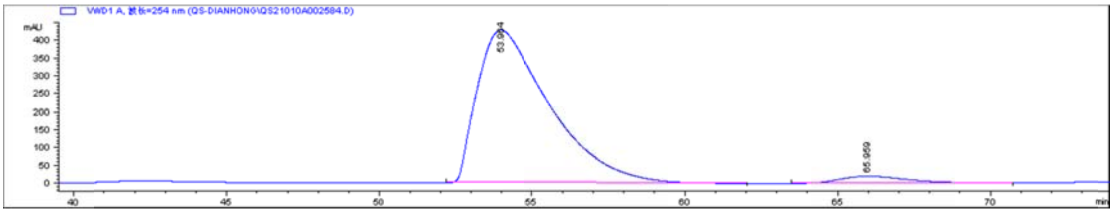

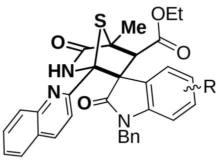

10d: R = 5-Br

Pale yellow solid, Mp 145.3–146.3 °C; 98% yield; 90% *ee*; $[\alpha]_D^{24}$ –86.1 (*c* 0.05, $CHCl_3$); ^1H NMR (300 MHz, $CDCl_3$) δ 8.03 (d, *J* = 2.6 Hz, 1H), 7.91 (d, *J* = 2.6 Hz, 1H), 7.88 (s, 2H), 7.71 (t, *J* = 7.4 Hz, 2H), 7.56 (t, *J* = 7.4 Hz, 1H), 7.12–7.03 (m, 2H), 6.98 (d, *J* = 8.4 Hz, 1H), 6.85 (t, *J* = 7.6 Hz, 2H), 6.78 (d, *J* = 7.6 Hz, 2H), 6.13 (d, *J* = 8.4 Hz, 1H), 4.81 (d, *J* = 15.9 Hz, 1H), 4.68 (d, *J* = 15.9 Hz, 1H), 3.76–3.66 (m, 3H), 1.97 (s, 3H), 0.60 (t, *J* = 7.1 Hz, 3H); ^{13}C NMR (75 MHz, $CDCl_3$) δ 176.1, 173.8, 167.5, 150.1, 146.6, 142.1, 136.7, 134.5, 132.1, 130.3, 129.4, 129.2, 128.4, 128.2, 127.5, 127.4, 126.5, 118.8, 114.7, 110.0,

67.5, 62.9, 60.9, 60.2, 44.1, 13.3, 13.1; HRMS (ESI) m/z 650.0720 (M+Na$^+$), calc. for $C_{32}H_{26}BrN_3O_4SNa$ 650.0725.

The *ee* was determined by HPLC analysis. CHIRALPAK ID-3 (4.6 mm i.d. x 250 mm); Hexane/2-propanol = 70/30; flow rate 1.0 mL/min; 25 °C; 254 nm; retention time: 50.7 min (minor) and 61.3 min (major).

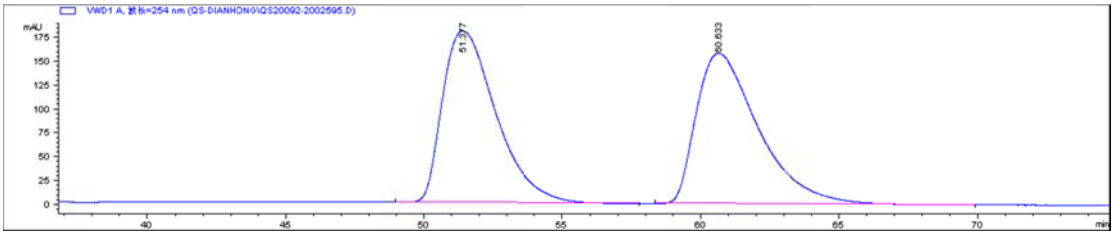

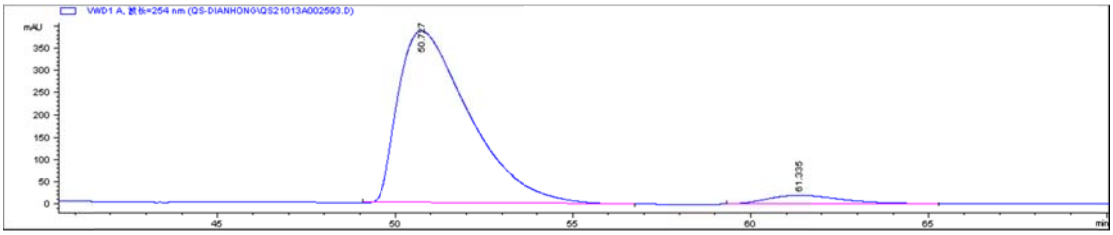

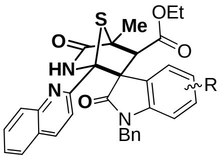

10e: R = 5-Me

Pale yellow solid, Mp 137.5–138.5 °C; 95% yield; >99% *ee*; $[\alpha]_D^{24}$ –431.3 (*c* 0.05, $CHCl_3$); ^{1}H NMR (300 MHz, $CDCl_3$) δ 7.99 (s, 1H), 7.93 (d, *J* = 8.4 Hz, 1H), 7.81 (d, *J* = 8.4 Hz, 1H), 7.73–7.68 (m, 3H), 7.57–7.52 (m, 1H), 7.01 (t, *J* = 7.8 Hz, 1H), 6.89 (d, *J* = 7.8 Hz, 1H), 6.80–6.71 (m, 5H), 6.12 (d, *J* = 7.8 Hz, 1H), 4.75 (d, *J* = 16.0 Hz, 1H), 4.69 (d, *J* = 16.0 Hz, 1H), 3.75 (s, 1H), 3.73–3.54 (m, 2H), 2.29 (s, 3H), 1.98 (s, 3H), 0.53 (t, *J* = 7.1 Hz, 3H); ^{13}C NMR (75 MHz, $CDCl_3$) δ 176.3, 174.2, 168.0, 150.5, 146.6, 140.7, 136.2, 135.1, 131.5, 130.2, 129.6, 128.3, 127.5, 127.4, 127.2, 127.1, 126.5, 126.0, 118.9, 108.5, 82.7, 67.9, 62.9, 60.7, 60.1, 44.0, 21.2, 13.2; HRMS (ESI) m/z 586.1780 (M+Na$^+$), calc. for $C_{33}H_{29}N_3O_4SNa$ 586.1776.

The *ee* was determined by HPLC analysis. CHIRALPAK ID-3 (4.6 mm i.d. x 250 mm); Hexane/2-propanol = 70/30; flow rate 1.0 mL/min; 25 °C; 254 nm; retention time: 75.3 min (major) and 98.9 min (minor).

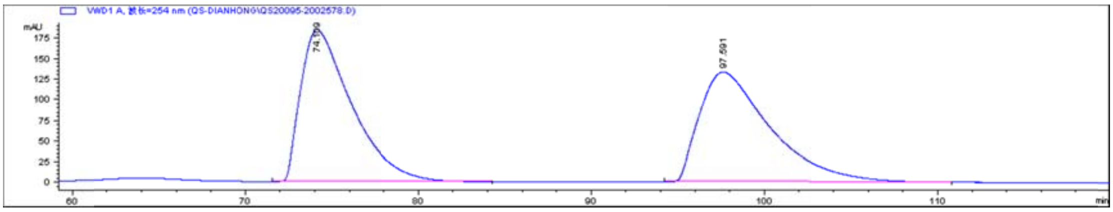

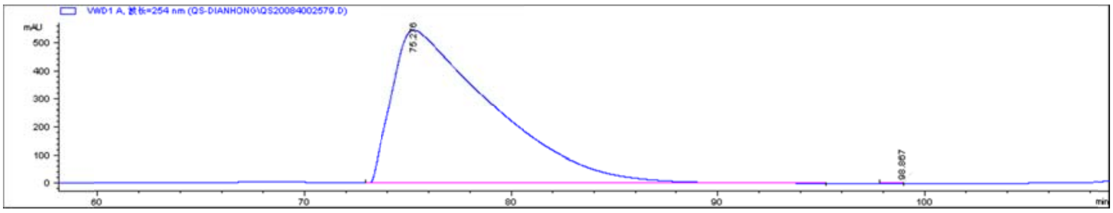

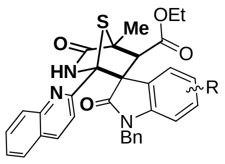

10f: R = 5-OMe

Pale yellow solid, Mp 141.1–142.3 °C; 98% yield; 98% *ee*; $[\alpha]_D^{24}$ –303.4 (*c* 0.05, $CHCl_3$); ^{1}H NMR (300 MHz, $CDCl_3$) δ 7.90 (s, 1H), 7.86 (d, *J* = 8.7 Hz, 1H), 7.76 (d, *J* = 8.7 Hz, 1H), 7.66–7.62 (m, 2H), 7.52–7.46 (m, 2H), 6.95 (t, *J* = 7.2 Hz, 1H), 6.87 (d, *J* = 8.5 Hz, 1H), 6.75–6.66 (m, 4H), 6.45 (dd, *J* = 8.5, 2.6 Hz, 1H), 6.06 (d, *J* = 8.5 Hz, 1H), 4.65 (s, 2H), 3.72–3.50 (m, 6H), 1.90 (s, 3H), 0.50 (t, *J* = 7.1 Hz, 3H); ^{13}C NMR (75 MHz, $CDCl_3$) δ 176.2, 173.9, 167.8, 155.2, 150.3, 146.6, 136.5, 136.3, 135.0, 130.2, 129.5, 128.3, 127.5, 127.4, 127.2, 127.1, 126.5, 118.7, 114.2, 113.5, 109.0, 82.7, 68.0, 62.9, 60.6, 60.0, 55.9, 44.0, 13.3, 13.1; HRMS (ESI) m/z 602.1724 (M+Na$^+$), calc. for $C_{33}H_{29}N_3O_5SNa$ 602.1726.

The *ee* was determined by HPLC analysis. CHIRALPAK ID-3 (4.6 mm i.d. x 250 mm); Hexane/2-propanol = 70/30; flow rate 1.0 mL/min; 25 °C; 254 nm; retention time: 93.3 min (major) and 136.3 min (minor).

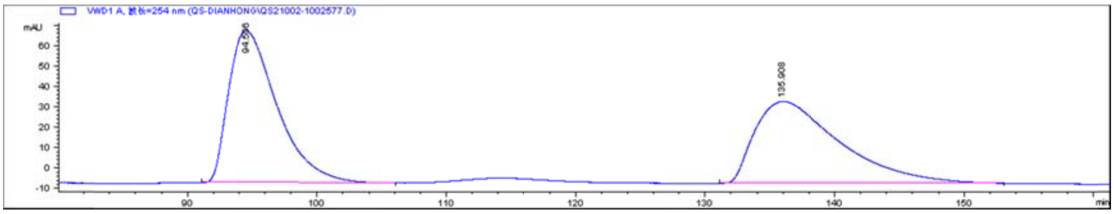

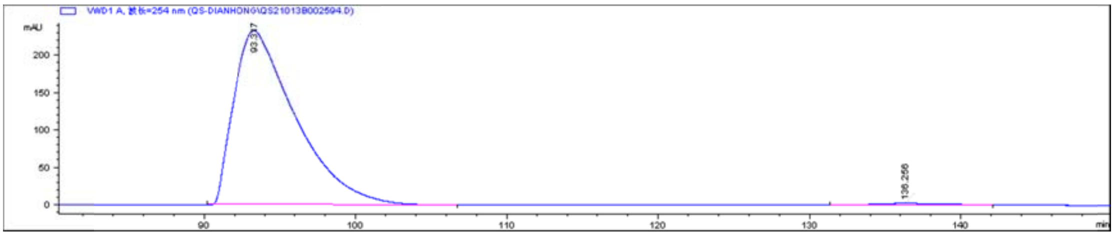

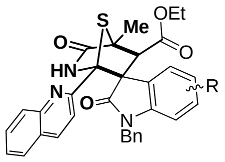

10g: R = 6-Cl

Pale yellow solid, Mp 96.4–96.9 °C; 97% yield; >99% *ee*; $[\alpha]_D^{24}$ –133.7 (*c* 0.05, $CHCl_3$); ^{1}H NMR (300 MHz, $CDCl_3$) δ 7.85–7.75 (m, 4H), 7.64 (t, *J* = 7.4 Hz, 2H), 7.52–7.46 (m, 1H), 7.00 (t, *J* = 7.4 Hz, 1H), 6.88–6.71 (m, 6H), 6.19 (s, 1H), 4.73 (d, *J* = 15.9 Hz, 1H), 4.61 (d, *J* = 15.9 Hz, 1H), 3.70–3.41 (m, 3H), 1.88 (s, 3H), 0.53 (t, *J* = 7.1 Hz, 3H); ^{13}C NMR (75 MHz, $CDCl_3$) δ 176.1, 174.2, 167.6, 150.2, 146.6, 144.2, 136.6, 135.2, 134.4, 130.3, 129.4, 128.5, 127.6, 127.5, 127.4, 127.3, 126.4, 124.4, 121.8, 118.7, 109.1, 82.7, 67.2, 62.8, 60.8, 60.3, 44.1, 13.3, 13.0; HRMS (ESI) m/z 584.1414 (M+H$^+$), calc. for $C_{32}H_{27}ClN_3O_4S$ 584.1411.

The *ee* was determined by HPLC analysis. CHIRALPAK ID-3 (4.6 mm i.d. x 250 mm); Hexane/2-propanol = 70/30; flow rate 1.0 mL/min; 25 °C; 254 nm; retention time: 44.4 min (minor) and 48.8 min (major).

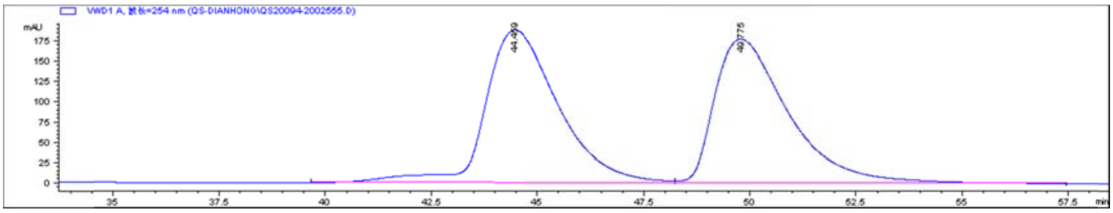

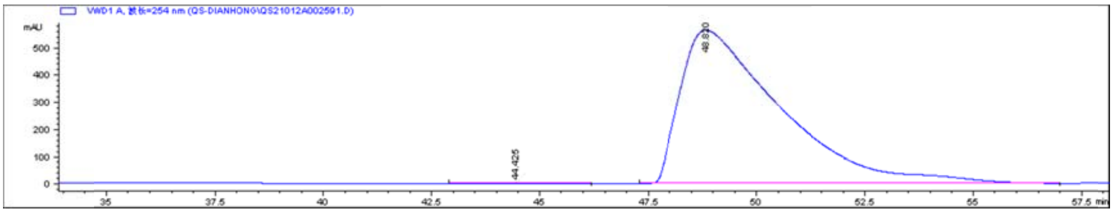

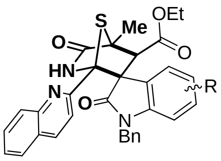

10h: R = 6-OMe

Pale yellow solid, Mp 123.0–124.8 °C; 94% yield; >99% *ee*; $[\alpha]_D^{24}$ –269.5 (*c* 0.05, $CHCl_3$); ^1H NMR (300 MHz, $CDCl_3$) δ 7.92–7.86 (m, 2H), 7.75 (dd, *J* = 8.5, 1.6 Hz, 2H), 7.67–7.62 (m, 2H), 7.52–7.46 (m, 1H), 6.94 (t, *J* = 7.1 Hz, 1H), 6.84 (d, *J* = 8.5 Hz, 1H), 6.73–6.63 (m, 4H), 6.38 (dd, *J* = 8.5, 2.3 Hz, 1H), 5.75 (d, *J* = 2.3 Hz, 1H), 4.68 (d, *J* = 15.9 Hz, 1H), 4.59 (d, *J* = 15.9 Hz, 1H), 3.70–3.48 (m, 6H), 1.89 (s, 3H), 0.54 (t, *J* = 7.2 Hz, 3H); ^{13}C NMR (75 MHz, $CDCl_3$) δ 176.2, 174.6, 168.0, 160.7, 150.5, 146.6, 144.3, 136.3, 134.9, 130.2, 129.6, 128.3, 127.5, 127.4, 127.2, 126.5, 118.9, 117.7, 105.4, 96.7, 82.6, 67.5, 62.8, 60.6, 60.1, 55.2, 44.0, 13.4, 13.1; HRMS (ESI) m/z 602.1730 ($M+Na^+$), calc. for $C_{33}H_{29}N_3O_5SNa$ 602.1726.

The *ee* was determined by HPLC analysis. CHIRALPAK ID-3 (4.6 mm i.d. x 250 mm); Hexane/2-propanol = 70/30; flow rate 1.0 mL/min; 25 °C; 254 nm; retention time: 101.9 min (minor) and 110.0 min (major).

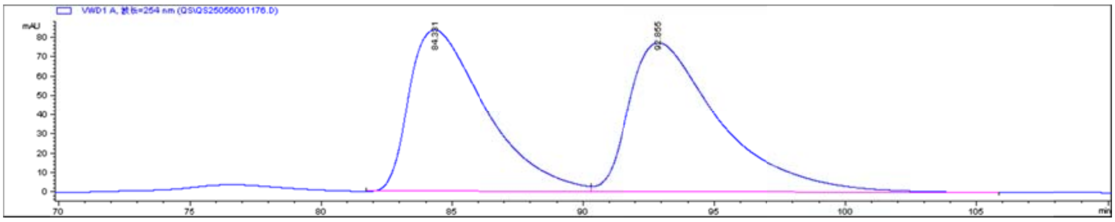

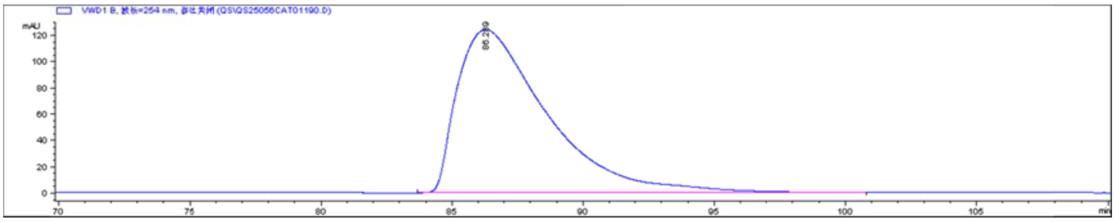

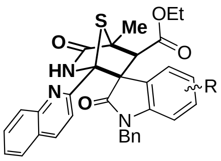

10i: R = 7-Cl

Pale yellow solid, Mp 70.4–72.1 °C; 96% yield; 93% *ee*; $[\alpha]_D^{24}$ –240.4 (*c* 0.05, $CHCl_3$); ^1H NMR (300 MHz, $CDCl_3$) δ 7.88–7.83 (m, 3H), 7.75–7.68 (m, 3H), 7.57–7.52 (m, 1H), 7.11–7.05 (m, 1H), 7.00–6.75 (m, 7H), 5.06 (d, *J* = 15.5 Hz, 1H), 4.78 (d, *J* = 15.5 Hz, 1H), 3.75 (s, 1H), 3.72–3.51 (m, 2H), 1.96 (s, 3H), 0.55 (t, *J* = 7.2 Hz, 3H); ^{13}C NMR (75 MHz, $CDCl_3$) δ 176.2, 174.2, 167.6, 150.1, 146.6, 136.5, 136.4, 130.3, 129.5, 128.2, 127.5, 127.4, 127.2, 126.7, 122.5, 122.4, 122.3, 122.2, 118.7, 117.6, 117.3, 82.9, 67.6, 62.8, 60.7, 60.6, 45.5, 13.2, 13.1; HRMS (ESI) m/z 606.1233 ($M+Na^+$), calc. for $C_{32}H_{26}ClN_3O_4SNa$ 606.1230.

The *ee* was determined by HPLC analysis. CHIRALPAK ID-3 (4.6 mm i.d. x 250 mm); Hexane/2-propanol = 70/30; flow rate 1.0 mL/min; 25 °C; 254 nm; retention time: 39.2 min (major) and 65.5 min (minor).

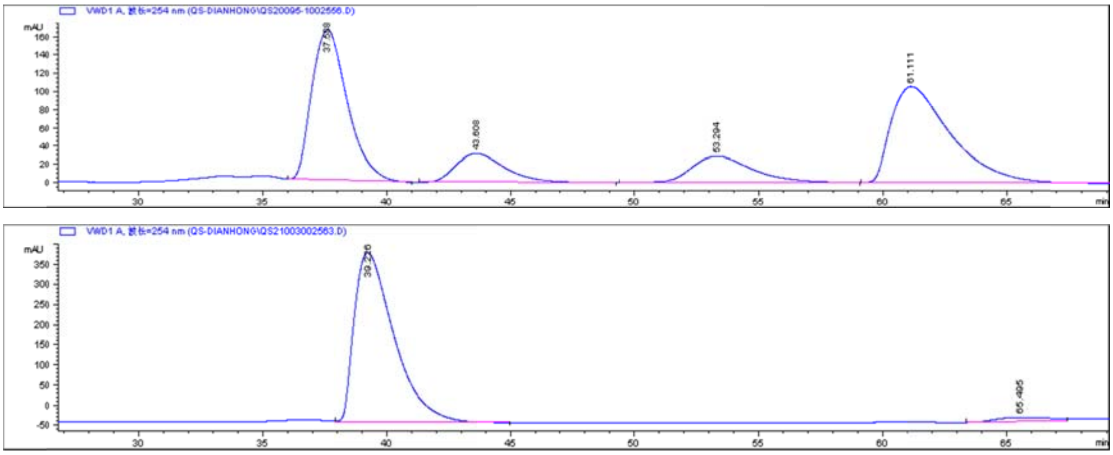

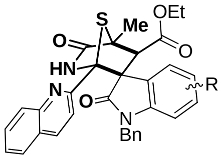

10j: R = 7-Me

Pale yellow solid, Mp 87.0–88.6 °C; 97% yield; 96% *ee*; $[\alpha]_D^{24}$ –281.9 (*c* 0.05, $CHCl_3$); ^1H NMR (300 MHz, $CDCl_3$) δ 7.90–7.87 (m, 2H), 7.82–7.75 (m, 2H), 7.67–7.63 (m, 2H), 7.52–7.46 (m, 1H), 6.98 (t, *J* = 7.4 Hz, 1H), 6.86–6.77 (m, 4H), 6.71 (d, *J* = 7.4 Hz, 1H), 6.62 (d, *J* = 7.4 Hz, 2H), 5.04 (d, *J* = 17.0 Hz, 1H), 4.84 (d, *J* = 17.0 Hz, 1H), 3.74–3.52 (m, 3H), 1.91 (s, 3H), 1.79 (s, 3H), 0.59 (t, *J* = 7.2 Hz, 3H); ^{13}C NMR (75 MHz, $CDCl_3$) δ 176.3, 175.3, 168.0, 150.5, 146.6, 141.2, 137.1, 136.2, 133.4, 130.2, 129.6, 128.5, 127.5, 126.9, 126.6, 125.2, 124.4, 122.0, 119.1, 119.0, 83.0, 67.2, 63.0, 60.7, 60.6, 45.3, 18.6, 13.5, 13.2; HRMS (ESI) m/z 586.1778 (M+Na$^+$), calc. for $C_{33}H_{29}N_3O_4SNa$ 586.1776.

The *ee* was determined by HPLC analysis. CHIRALPAK ID-3 (4.6 mm i.d. x 250 mm); Hexane/2-propanol = 70/30; flow rate 1.0 mL/min; 25 °C; 254 nm; retention time: 79.9 min (major) and 139.8 min (minor).

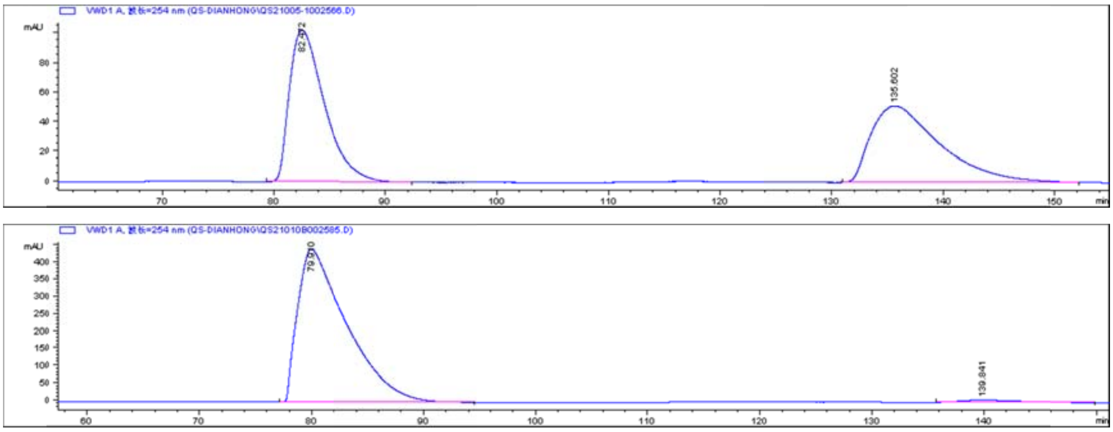

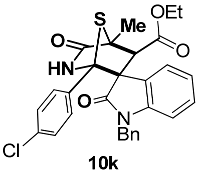

10k

Pale yellow solid, Mp 87.8–89..8 °C; 94% yield; 90% *ee*; $[\alpha]_D^{24}$ –72.5 (*c* 0.05, $CHCl_3$); ^1H NMR (300 MHz, $CDCl_3$) δ 7.82 (d, *J* = 7.3 Hz, 1H), 7.61 (s, 1H), 7.28–7.25 (m, 3H), 7.08–7.04 (m, 3H), 6.99–6.88 (m, 5H), 6.46 (d, *J* = 7.3 Hz, 1H), 5.03 (d, *J* = 15.7 Hz, 1H), 4.67 (d, *J* = 15.7 Hz, 1H), 3.68–3.45 (m, 3H), 1.92 (s, 3H), 0.47 (t, *J* = 7.2 Hz, 3H); ^{13}C NMR (75 MHz, $CDCl_3$) δ 177.7, 174.8, 167.6, 142.5, 135.0, 134.9, 129.7, 129.4, 128.7, 128.3, 127.8, 127.3, 127.1, 126.6, 126.0, 122.4, 108.8, 82.6, 68.0, 61.3, 60.7, 60.4, 44.0, 13.2, 12.9; HRMS (ESI) m/z 555.1123 (M+Na$^+$), calc. for $C_{29}H_{25}ClN_2O_4SNa$ 555.1121.

The *ee* was determined by HPLC analysis. CHIRALPAK IC (4.6 mm i.d. x 250 mm); Hexane/2-propanol = 70/30; flow rate 1.0 mL/min; 25 °C; 254 nm; retention time: 19.4 min (major) and 22.5 min (minor).

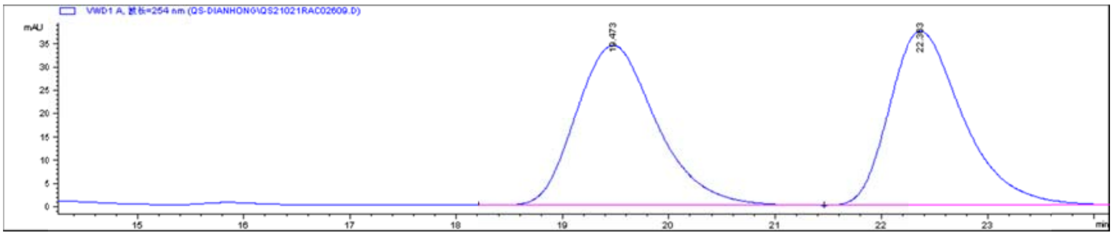

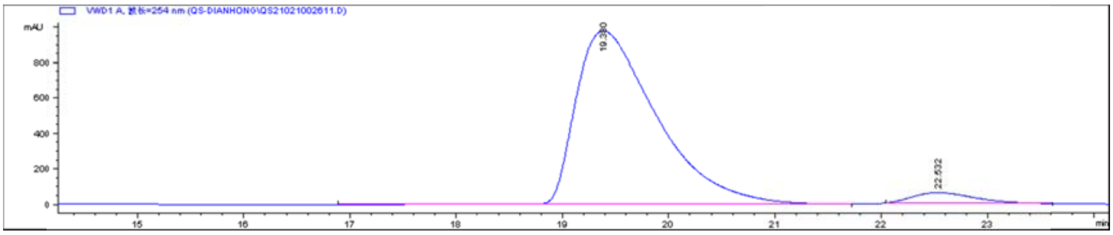

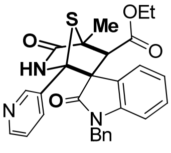

10l

Pale yellow solid, Mp 101.3–102.6 °C; 96% yield; 95% *ee*; $[\alpha]_D^{24}$ –62.0 (*c* 0.05, $CHCl_3$); ^1H NMR (300 MHz, $CDCl_3$) δ 8.25 (d, *J* = 4.2 Hz, 1H), 7.81–7.78 (m, 1H), 7.59 (s, 1H), 7.33 (td, *J* = 7.7, 1.7 Hz, 1H), 7.26–7.22 (m, 3H), 7.12–6.98 (m, 4H), 6.92–6.82 (m, 2H), 6.39 (d, *J* = 7.7 Hz, 1H), 4.89 (d, *J* = 15.7 Hz, 1H), 4.72 (d, *J* = 15.7 Hz, 1H), 3.71–3.49 (m, 3H), 1.94 (s, 3H), 0.50 (t, *J* = 7.2 Hz, 3H); ^{13}C NMR (75 MHz, $CDCl_3$) δ 176.6, 174.4, 167.8, 150.4, 148.5, 142.9, 136.0, 135.3, 129.2, 128.5, 127.5, 127.2, 126.1, 126.0, 123.6, 122.0, 121.6, 108.4, 82.8, 67.3, 62.4, 60.6, 60.3, 44.0, 13.2, 13.1; HRMS (ESI) m/z 522.1466 ($M+Na^+$), calc. for $C_{28}H_{25}N_3O_4SNa$ 522.1463.

The *ee* was determined by HPLC analysis. CHIRALPAK IC (4.6 mm i.d. x 250 mm); Hexane/2-propanol = 70/30; flow rate 1.0 mL/min; 25 °C; 254 nm; retention time: 21.1 min (major) and 31.4 min (minor).

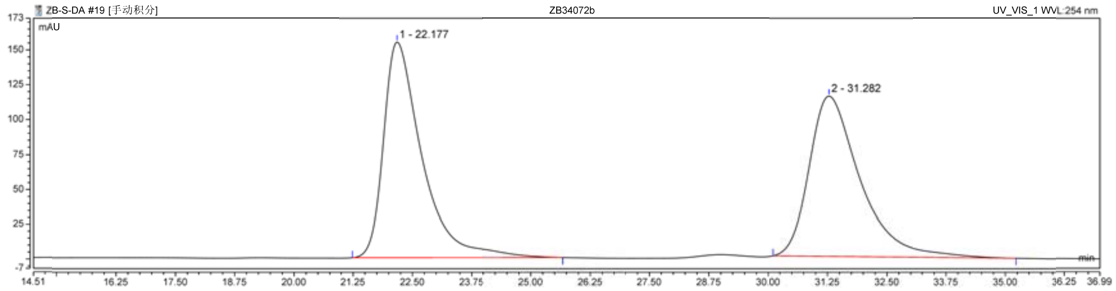

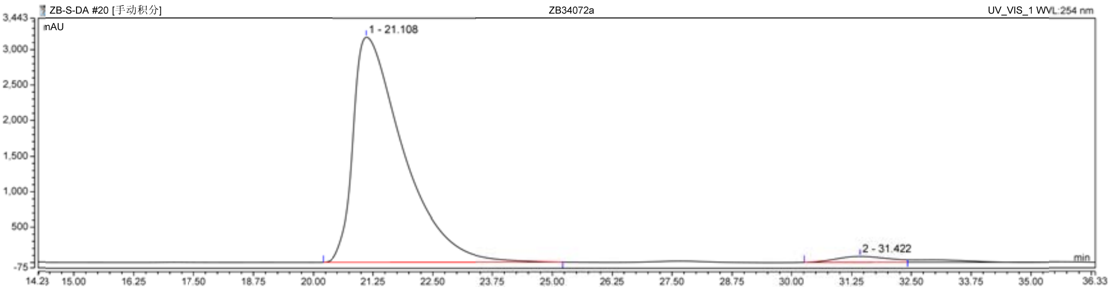

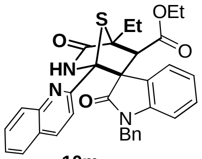

10m

Pale yellow solid, Mp 92.3–94.1 °C; 87% yield; >99% *ee*; $[\alpha]_D^{24}$ –67.8 (*c* 0.05, $CHCl_3$); ^1H NMR (300 MHz, $CDCl_3$) δ 7.93–7.91 (m, 3H), 7.80 (d, *J* = 7.8 Hz, 1H), 7.79–7.68 (m, 2H), 7.54 (t, *J* = 7.8 Hz, 1H), 7.06–7.04 (m, 1H), 6.98–6.91 (m, 3H), 6.86–6.78 (m, 4H), 6.25–6.22 (m, 1H), 4.79 (d, *J* = 15.8 Hz, 1H), 4.71 (d, *J* = 15.8 Hz, 1H), 3.79 (s, 1H), 3.72–3.49 (m, 2H), 2.60 (dq, *J* = 14.5, 7.2 Hz, 1H), 2.40 (dq, *J* = 14.5, 7.2 Hz, 1H), 1.27 (t, *J* = 7.2 Hz, 3H), 0.52 (t, *J* = 7.2 Hz, 3H); ^{13}C NMR (75 MHz, $CDCl_3$) δ 175.5, 174.3, 167.9, 150.6, 146.6, 143.0, 136.2, 135.0, 130.2, 129.5, 129.3, 128.4, 127.5, 127.4, 127.3, 126.6, 125.9, 121.9, 118.9, 108.6, 81.9, 69.4, 67.5, 60.6, 59.4, 44.0, 20.4, 13.2, 12.0; HRMS (ESI) m/z 586.1775 ($M+Na^+$), calc. for $C_{33}H_{29}N_3O_4SNa$ 586.1776.

The *ee* was determined by HPLC analysis. CHIRALPAK IC (4.6 mm i.d. x 250 mm); Hexane/2-propanol = 70/30; flow rate 1.0 mL/min; 25 °C; 254 nm; retention time: 28.5 min (minor) and 3401 min (major).

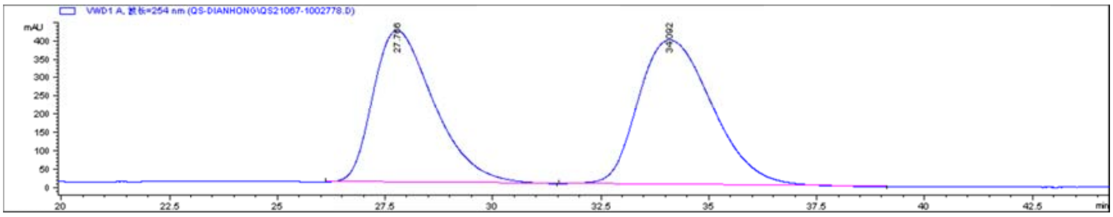

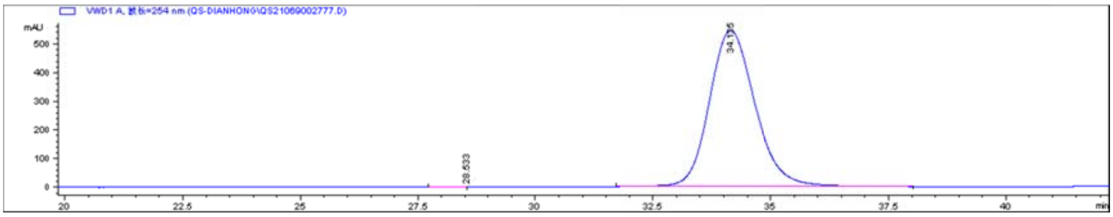

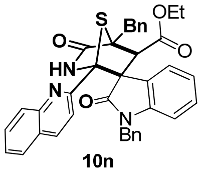

10n

Pale yellow solid, Mp 108.2–109.9 °C; 84% yield; 97% *ee*; $[\alpha]_D^{24}$ –101.0 (*c* 0.05, $CHCl_3$); ^1H NMR (300 MHz, $CDCl_3$) δ 7.98–7.89 (m, 3H), 7.73–7.64 (m, 3H), 7.58–7.48 (m, 3H), 7.37–7.30 (m, 3H), 7.24–7.22 (m, 1H), 7.06–6.92 (m, 3H), 6.86–6.78 (m, 5H), 6.24–6.21 (m, 1H), 4.79 (d, *J* = 15.9 Hz, 1H), 4.72 (d, *J* = 15.9 Hz, 1H), 3.85 (s, 1H), 3.81 (s, 1H), 3.76–3.53 (m, 2H), 0.54 (t, *J* = 7.2 Hz, 3H); ^{13}C NMR (75 MHz, $CDCl_3$) δ 175.1, 174.1, 168.1, 150.4, 146.5, 143.0, 139.1, 136.0, 135.0, 130.1, 130.0, 129.5, 129.4, 128.4, 128.3, 127.5, 127.3, 126.9, 126.6, 125.8, 122.0, 118.9, 108.7, 81.6, 70.7, 68.1, 60.7, 59.5, 44.0, 31.9, 13.3; HRMS (ESI) m/z 648.1932 ($M+Na^+$), calc. for $C_{38}H_{31}N_3O_4SNa$ 648.1933.

The *ee* was determined by HPLC analysis. CHIRALPAK IC (4.6 mm i.d. x 250 mm); Hexane/2-propanol = 70/30; flow rate 1.0 mL/min; 25 °C; 254 nm; retention time: 24.8 min (minor) and 44.5 min (major).

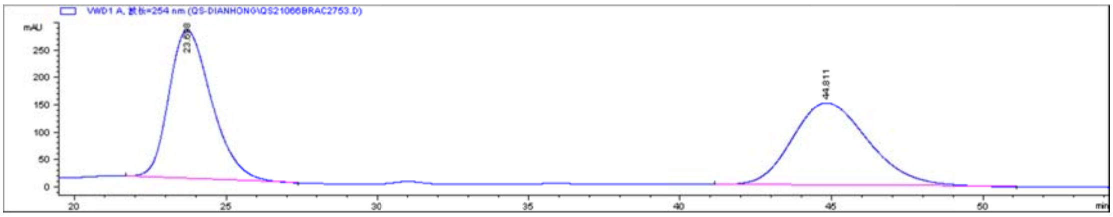

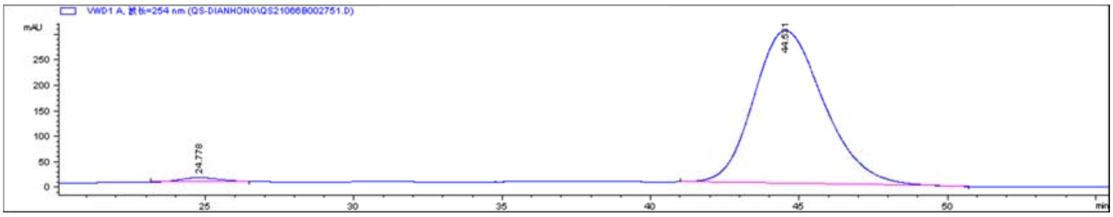

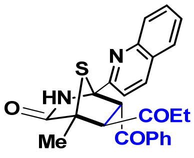

ent-**6l**

White solid, Mp 128.6–129.8 °C; 91% yield; 97% *ee*; $[\alpha]_D^{26}$ +183.4 (*c* 3.44, $CHCl_3$); ^{1}H NMR (300 MHz, $CDCl_3$) δ 8.13 (d, *J* = 8.5 Hz, 1H), 7.73–7.69 (m, 3H), 7.65–7.56 (m, 4H), 7.48 (t, *J* = 7.3 Hz, 1H), 7.35 (t, *J* = 7.3 Hz, 1H), 7.22 (d, *J* = 7.3 Hz, 1H), 5.70 (d, *J* = 5.5 Hz, 1H), 3.50 (d, *J* = 5.5 Hz, 1H), 2.66 (dq, *J* = 18.2, 7.2 Hz, 1H), 2.29 (dq, *J* = 18.2, 7.2 Hz, 1H), 1.72 (s, 3H), 1.04 (t, *J* = 7.2 Hz, 3H); ^{13}C NMR (75 MHz, $CDCl_3$) δ 207.9, 198.3, 176.6, 152.2, 146.8, 137.5, 137.0, 133.1, 129.9, 129.3, 128.4, 128.0, 127.6, 127.3, 127.2, 118.2, 79.6, 66.8, 62.3, 57.4, 39.1, 14.2, 7.6; HRMS (ESI) m/z 431.1430 (M+H$^+$), calc. for $C_{25}H_{23}N_2O_3S$ 431.1429.

The *ee* was determined by HPLC analysis. CHIRALPAK IC (4.6 mm i.d. x 250 mm); Hexane/2-propanol = 80/20; flow rate 1.0 mL/min; 25 °C; 254 nm; retention time: 19.7 min (major) and 22.8 min (minor).

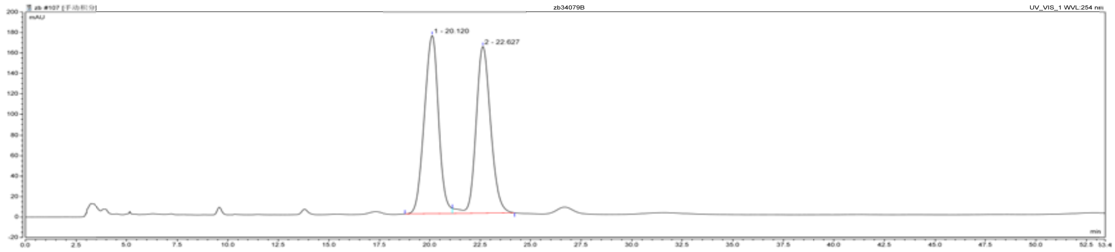

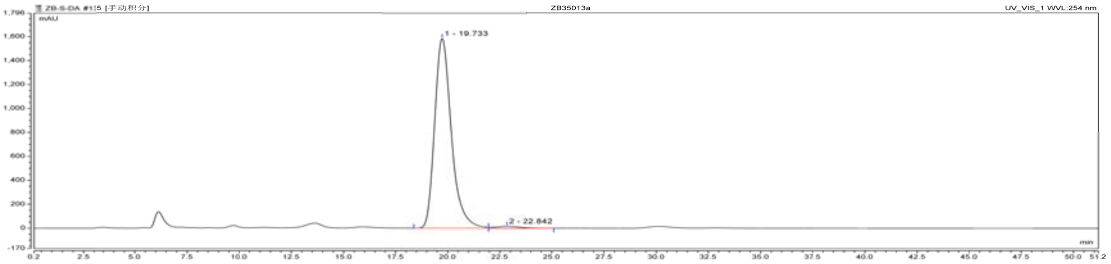

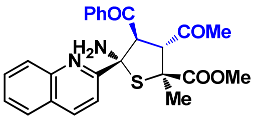

11

Green solid, Mp 121.9–123.0 °C; 73% yield; 98% *ee*; $[\alpha]_D^{26}$ +161.4 (*c* 0.94, $CHCl_3$); ^{1}H NMR (300 MHz, $CDCl_3$) δ 8.06 (d, *J* = 8.7 Hz, 1H), 7.92 (d, *J* = 8.7 Hz, 1H), 7.71 (d, *J* = 8.4 Hz, 2H), 7.58 (ddd, *J* = 8.4, 6.3, 1.4 Hz, 1H), 7.46 (ddd, *J* = 8.4, 6.3, 1.4 Hz, 3H), 6.99 (d, *J* = 7.5 Hz, 1H), 6.86 (t, *J* = 7.5 Hz, 2H), 5.42 (d, *J* = 12.5 Hz, 1H), 5.25 (d, *J* = 12.5 Hz, 1H), 3.91 (s, 3H), 2.28 (s, 3H), 1.75 (s, 3H); ^{13}C NMR (75 MHz, $CDCl_3$) δ 205.5, 198.3, 174.0, 158.5, 146.1, 137.5, 136.6, 131.9, 129.5, 129.2, 128.1, 127.4, 127.0, 126.8, 119.3, 77.3, 62.7, 61.9, 55.0, 53.5, 30.9, 23.7; HRMS (ESI) m/z 449.1539 (M+H$^+$), calc. for $C_{25}H_{25}N_2O_4S$ 449.1535.

The *ee* was determined by HPLC analysis. CHIRALPAK IC (4.6 mm i.d. x 250 mm); Hexane/2-propanol = 80/20; flow rate 1.0 mL/min; 25 °C; 254 nm; retention time: 30.5 min (major) and 37.9 min (minor).

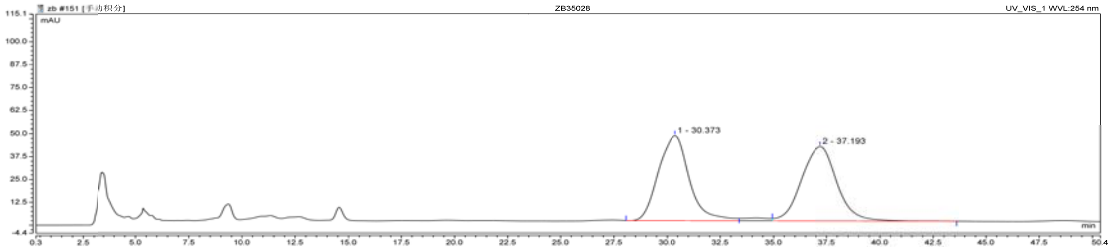

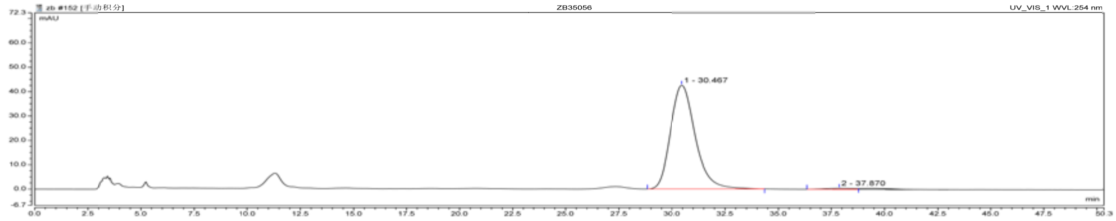

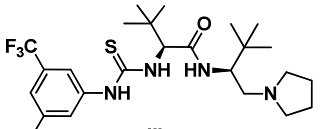

III

White solid, ^{1}H NMR (300 MHz, $CDCl_3$) δ 7.98 (s, 2H), 7.30 (s, 1H), 4.22 (s, 2H), 2.73 (s, 6H), 1.89 (s, 4H), 1.17 (s, 9H), 1.00 (s, 9H); ^{13}C NMR (75 MHz, $CDCl_3$) δ 182.0, 172.7, 140.2, 131.6, 131.2, 130.9, 130.7, 130.3, 128.8, 128.4, 124.8, 123.9, 121.2, 117.8, 67.0, 65.6, 55.7, 54.0, 34.7, 33.8, 27.3, 26.6, 23.4; HRMS (ESI) m/z 555.2593 ($M+H^+$), calc. for $C_{25}H_{37}\mathbf{F}_6N_4OS$ 555.2592.

14. Determination of the absolute configuration

(1) Absolute configurations of the [4+2] annulation adducts **3a-n** are determined by *X*-ray structure analysis of the product **3a** (CCDC 1419583)

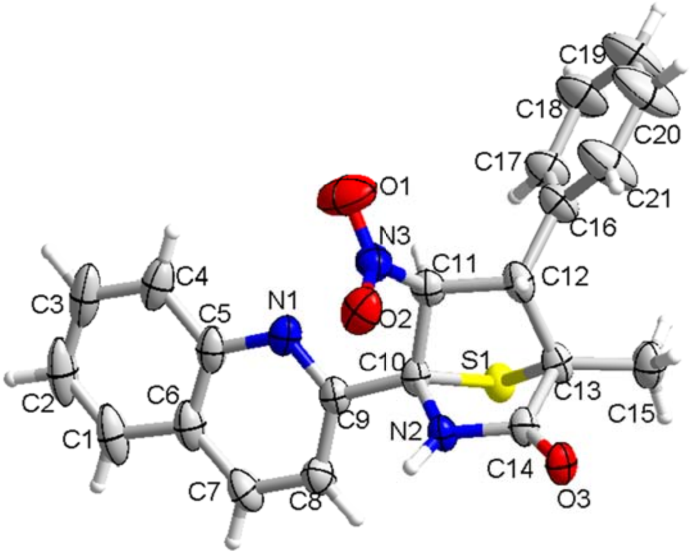

(2) Absolute configurations of the [4+2] annulation adducts **6a-s** and **9a-h** are determined by *X*-ray structure analysis of the product **6b** (CCDC 1419574)

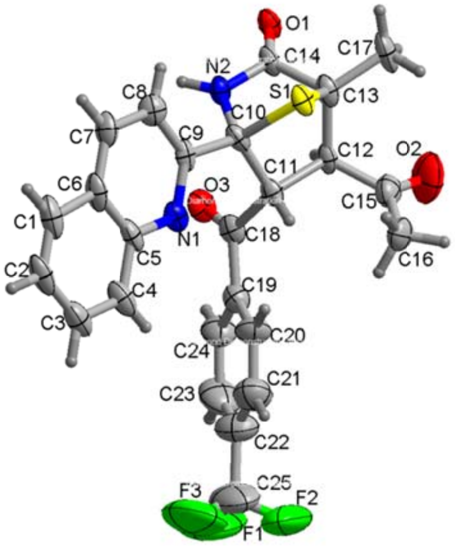

(3) Absolute configurations of the [4+2] annulation adducts **11a-n** are determined by *X*-ray structure analysis of the product **11d** (CCDC 1463891)

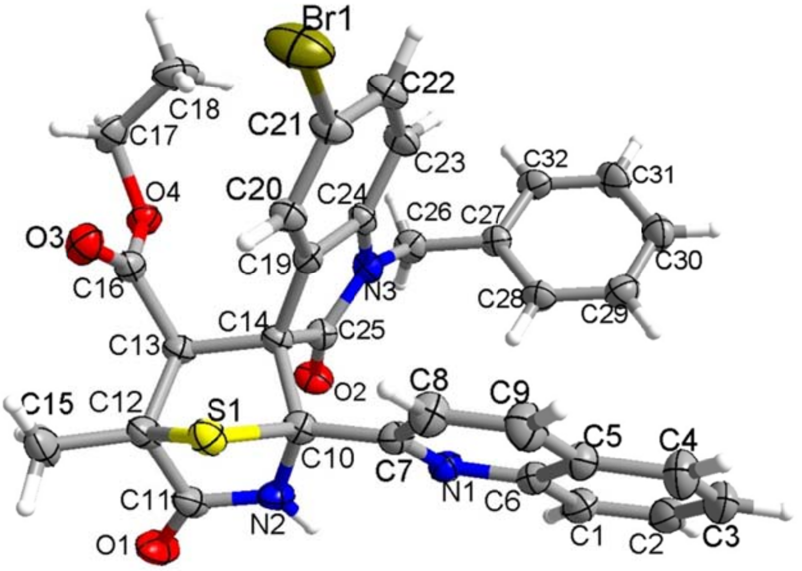
Br1
C22
C18
C21
C17
C23
C32
C31
C20
O4
C24
C26
C27
O3
C30
C19
C16
N3
C28
C29
C14
C25
C13
O2
C8
C9
C15
C12
S1
C5
C4
C10
C7
N1
C6
C11
N2
C1
C2
C3
O1

15. Computational methods

All quantum chemical calculations were carried out with Gaussian 09[1]. All Δ*G* values are reported relative to the individual free starting materials **1**, **2** and catalyst **III.**

Optimization of minimum and transition state (TS) structures were first carried out with Becke's three parameter[2] and Lee-Yang-Parr's[3] B3LYP density functional using Pople's[4] 6-31G(d,p) basis set under the SMD[5] polarizable continuum solvent model (toluene parameters). It is important to note that Simón and Goodman[6] showed that B3LYP/6-31G(d) is adequate to model a variety of organocatalytic reactions. Subsequently, Houk *et al*[7] applied the dispersion-corrected meta-hybrid M06-2X(D3) approach towards modelling stereoselective intramolecular Aldol reaction and thiourea-amine catalyzed Nazarov cyclization with excellent qualitative agreement to experiment.

The B3LYP/6-31G(d,p) thermal and vibrational corrections from frequency calculations were combined to higher level single-point energies of the optimized geometries. The single-point energies were evaluated with Peverati and Truhlar's screened-exchange density functional MN12-SX under same polarizable continuum solvent model toluene parameters, herein termed MN12-SX/SMD.[8] The newer meta-hybrid non separable gradient MN12-SX functional was assessed to perform better in chemical energetics and thus preferred over M06-2X.[9] In conjunction with the MN12-SX/SMD, the Wiegend and Aldrich's def2-TZVPP triple zeta quality basis set was used.[10] Second order derivative or Hessian of the completed calculations were checked to verify transition state structures having only one negative eigenvalue and none for minimum.

Gibbs free energies in solution were calculated from the geometries, frequencies and improved energies using a thermocycle in which the gas-phase was treated using the standard textbook formulae for an ideal gas under the harmonic oscillator / rigid rotor approximation, and the Gibbs free energies of solvation (in toluene) were then calculated using the SMD continuum solvation model. Corrections were included to consider passage of 1 atm gas into 1M in solution, $\Delta G^{1atm\rightarrow 1M}$ as follows[11]:

$$\Delta G^{1atm\rightarrow 1M} = dN*R*T*\ln(RT/P)$$

where dN is the number of moles of gas change from reactant to product and ln(RT/P) equals to 1.89 at 298 K. We have recently shown that this approach provides an excellent quantitative description of the temperature dependent behavior of solution-phase Diels-Alder reactions.[12]

Electron density topological analyses based on the reduced density gradient was carried out with NCIplot[13] to qualify regions of non-covalent interactions especially hydrogen bonding

(H-bonding) or regions of repulsive interactions. Regions which are attractive have sign of the second density Hessian eigenvalue, $sign(\lambda_2)$, as positive and repulsive as negative. The electron density ρ(r) is set at an isovalue of 0.4 au in the $sign(\lambda_2)\rho$(r) range of -0.4 to 0.4, which corresponds to blue-green-red color scale (see Figure S1). The 3-D molecular graphics for NCIplot were rendered with VMD[14] and graphics inset corresponding to the NCIplot showing key H-bond interactions in figure 3 of the manuscript were rendered with Cylview[15].

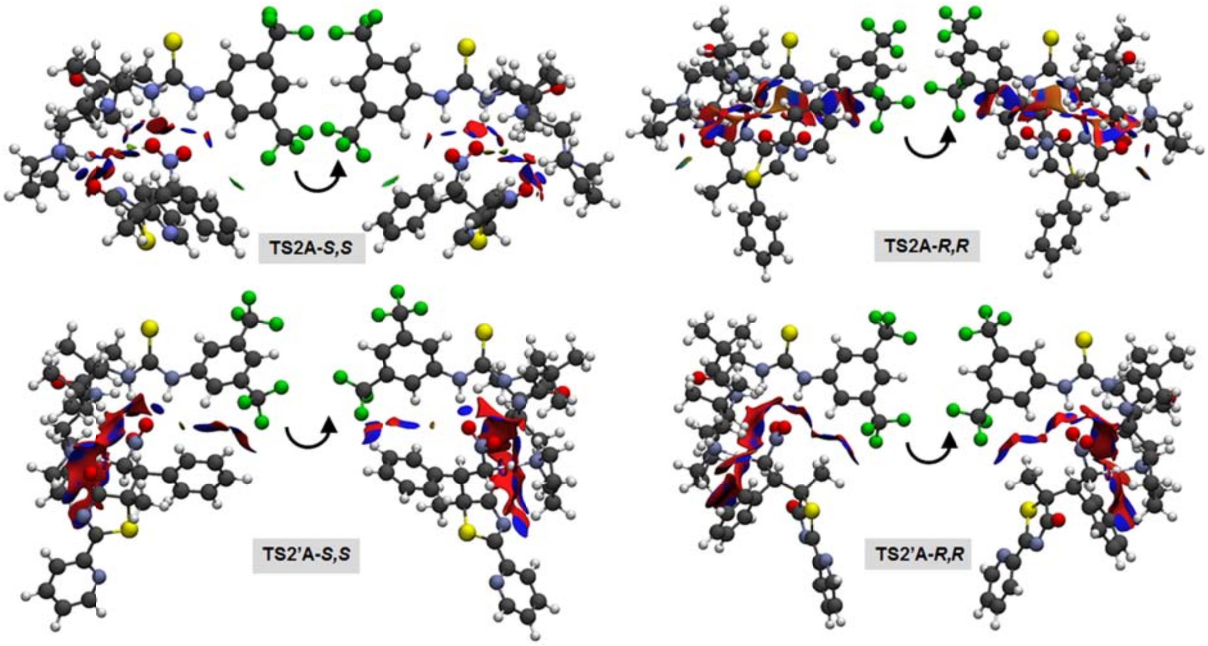

Figure S1. Non-covalent interaction isosurfaces of optimized TS structures of Mannich process (**TS2A-*S,S*** and **TS2A-*R,R***) and the protonation process (**TS2'A-*S,S*** and **TS2'A-*R,R***). Blue-green-red color scale from -0.4 < $sign(\lambda_2)\rho$(r) > 0.4 au, where blue (positive) is favorable and red (negative) is unfavorable interaction. Two images for each TS are flipped horizontally to allow viewing from the front and back sides.

References

1. Frisch, M. J.; Trucks, G. W.; Schlegel, H. B.; Scuseria, G. E.; Robb, M. A.; Cheeseman, J. R.; Scalmani, G.; Barone, V.; Mennucci, B.; Petersson, G. A.; Nakatsuji, H.; Caricato, M.; Li, X.; Hratchian, H. P.; Izmaylov, A. F.; Bloino, J.; Zheng, G.; Sonnenberg, J. L.; Hada, M.; Ehara, M.; Toyota, K.; Fukuda, R.; Hasegawa, J.; Ishida, M.; Nakajima, T.; Honda, Y.; Kitao, O.; Nakai, H.; Vreven, T.; Montgomery, J., J. A. ; Peralta, J. E.; Ogliaro, F.; Bearpark, M.; Heyd, J. J.; Brothers, E.; Kudin, K. N.; Staroverov, V. N.; Kobayashi, R.; Normand, J.; Raghavachari, K.; Rendell, A.; Burant, J. C.; Iyengar, S. S.; Tomasi, J.; Cossi, M.; Rega, N.; Millam, J. M.; Klene, M.; Knox, J. E.; Cross, J. B.; Bakken, V.; Adamo, C.; Jaramillo, J.; Gomperts, R.; Stratmann, R. E.; Yazyev, O.; Austin, A. J.; Cammi, R.; Pomelli, C.; Ochterski, J. W.; Martin, R. L.; Morokuma, K.; Zakrzewski, V. G.; Voth, G. A.; Salvador, P.; Dannenberg, J. J.; Dapprich, S.; Daniels, A. D.; Farkas, Ö.; Foresman, J. B.; Ortiz, J. V.; Cioslowski, J.; Fox, D. J. *Gaussian 09*, Revision D.01; Gaussian Inc.: Wallingford CT, 2013.
2. Becke, A. D., *J. Chem. Phys.* **1993,** *98*, 5648-5652.
3. Lee, C.; Yang, W.; Parr, R. G., *Phys. Rev. B* **1988,** *37*, 785-789.
4. (a) Ditchfield, R.; Hehre, W. J.; Pople, J. A., *J. Chem. Phys.* **1971,** *54*, 724-728; (b) Hehre, W. J.; Ditchfield, R.; Pople, J. A., *J. Chem. Phys.* **1972,** *56*, 2257-2261.
5. Marenich, A. V.; Cramer, C. J.; Truhlar, D. G., *J. Phys. Chem. B* **2009,** *113*, 6378-6396.

6. Simón, L.; Goodman, J. M., *Org. Biomol. Chem.* **2011,** *9*, 689-700.
7. (a) Lam, Y.-H.; Houk, K. N., *J. Am. Chem. Soc.* **2015,** *137*, 2116−2127; (b) Asari, A. H.; Lam, Y.-H.; Tius, M. A.; Houk, K. N., *J. Am. Chem. Soc.* **2015,** *137*, 13191–13199.
8. Peverati, R.; Truhlar, D. G., *Phys. Chem. Chem. Phys.* **2012,** *14*, 16187-16191.
9. Peverati, R.; Truhlar, D. G., *Phil. Trans. R. Soc. A* **2014,** *372*, 20120476.
10. Weigend, F.; Ahlrichs, R., *Phys. Chem. Chem. Phys.* **2005,** *7*, 3297−3305.
11. Ho, J.; Klamt, A.; Coote, M. L., **2010,** *114*, 13442–13444.
12. Oehlenschlaeger, K. K.; Guimard, N. K.; Brandt, J.; Mueller, J. O.; Lin, C. Y.; Hilf, S.; Lederer, A.; Coote, M. L.; Schmidt, F. G.; Barner-Kowollik, C., *Polym. Chem.* **2013,** *4*, 4348-4355.
13. (a) Johnson, E. R.; Keinan, S.; Mori-Sanchez, P.; Contreras-Garcia, J.; Cohen, A. J.; Yang, W., *J. Am. Chem. Soc.* **2010,** *132*, 6498–6506; (b) Contreras-Garcia, J.; Johnson, E. R.; Keinan, S.; Chaudret, R.; Piquemal, J.-P.; Beratan, D. N.; Yang, W., *J. Chem. Theory Comput.* **2011, ,** *7*, 625-632.
14. Humphrey, W.; Dalke, A.; Schulten, K., *J. Molec. Graphics* **1996,** *14*, 33-38.
15. Legault, C. *Cylview*, 1.0b; 2007.

Molecule	MN12SX E	B3LYP E	$G_{correction}$	$H_{correction}$	ZPE (298K)	G_{sol} (MN12SX)
**III**	-2265.238831	-2265.933466	0.526197	0.640233	0.60057	-2264.703614
**1**	-930.478833	-930.724803	0.119136	0.169065	0.157119	-930.359697
**2**	-513.963184	-514.176050	0.101575	0.146955	0.136956	-513.861609
**TS1A-*R,R***	-3709.692433	-3710.841985	0.796699	0.960728	0.899259	-3708.892727
**TS1A-*S,S***	-3709.695202	-3710.844099	0.796696	0.961218	0.899614	-3708.895499
**TS1B-*R,R***	-3709.69039	-3710.83715	0.797744	0.961166	0.899645	-3708.889639
**TS1B-*S,S***	-3709.688422	-3710.838464	0.797171	0.961137	0.899743	-3708.888244
**int1A-*R,R***	-3709.705159	-3710.854112	0.799168	0.96316	0.90194	-3708.902984
**int1A-*S,S***	-3709.714753	-3710.863604	0.800902	0.963818	0.902572	-3708.910844
**int1B-*R,R***	-3709.714366	-3710.860514	0.796381	0.961835	0.899977	-3708.914978
**int1B-*S,S***	-3709.713598	-3710.861334	0.798688	0.962503	0.901149	-3708.911903
**TS2A-*R,R***	-3709.699503	-3710.843566	0.804677	0.962433	0.902263	-3708.891819
**TS2A-*S,S***	-3709.698245	-3710.843839	0.799844	0.962093	0.901234	-3708.895394
**int2A-*R,R***	-3709.710927	-3710.853914	0.802751	0.964022	0.903051	-3708.905169
**int2A-*S,S***	-3709.71474	-3710.857422	0.800695	0.963549	0.902543	-3708.911038
**TS2'A-*R,R***	-3709.690718	-3710.836448	0.795499	0.957718	0.896357	-3708.892212
**TS2'A-*S,S***	-3709.693888	-3710.837503	0.798308	0.957859	0.897082	-3708.892573
**int2'A-*R,R***	-3709.712573	-3710.860005	0.792608	0.962243	0.899753	-3708.916958
**int2'A-*S,S***	-3709.712922	-3710.861514	0.795597	0.962489	0.900231	-3708.914318

*All energy values in hatrees.

Gaussian Archive Files

Catalyst **III**

```
1\1\GINC-R1102\FOpt\RB3LYP\6-31G(d,p)\C25H36F6N4O1S1\ROOT\09-Dec-2015\
0\\# freq=noraman rb3lyp/6-31g** opt=maxcyc=200 scf=maxcyc=200 scrf=(s
md,solvent=toluene)\\Catalyst III\\0,1\C,1.9345173079,0.1913285951,-1.
2675001586\O,2.7222233811,-0.0115586208,-2.1905041561\C,1.0106971573,1
.4394955675,-1.3269008674\H,0.645363335,1.4648738374,-2.355854527\N,1.
8184572705,-0.5798134111,-0.158914149\H,1.0738800145,-0.3245909597,0.4
718634769\C,2.6492454085,-1.7284155289,0.2519299286\H,2.4689319057,-1.
8051190086,1.3308211293\C,2.1719890722,-3.1040802263,-0.3402051028\C,0
.6437094855,-3.217673919,-0.1766577936\H,0.1121645481,-2.4877299589,-0
.7921152638\H,0.3374482578,-3.0747134033,0.8680231677\H,0.3043017939,-
4.2137057113,-0.4809826574\C,2.5322001042,-3.2642068952,-1.829019438\H
,2.1307405187,-2.4483810422,-2.4337829887\H,2.1251325727,-4.2074545727
,-2.2112858297\H,3.6154797385,-3.2896756892,-1.9864691966\C,2.81628368
24,-4.2514734696,0.4676217447\H,3.9049038954,-4.2836959,0.3649586651\H
,2.4316736996,-5.2156645468,0.1178654957\H,2.5824557205,-4.1737072856,
1.5365026558\C,4.1490892125,-1.4358323091,0.0511560015\H,4.7191826574,
-2.3523108328,0.28289469\H,4.3201618973,-1.1936768557,-0.9978704599\N,
4.636044141,-0.3165031866,0.8549308163\C,5.9845376224,0.1133510513,0.4
62546004\H,5.9653950685,0.5831364452,-0.5257586169\H,6.6786769798,-0.7
486247418,0.4079606765\C,6.4152538149,1.065952585,1.5896537744\H,7.495
9584795,1.0321948572,1.7529615673\H,6.15458027,2.098312689,1.340152949
1\C,4.7481579731,-0.5816959913,2.29408829\H,5.2546508957,-1.5492740071
,2.4818874798\H,3.7634183122,-0.6312522996,2.76944665\C,5.6066390903,0
.582485208,2.8266273613\H,6.2501935688,0.2545677982,3.6478160651\H,4.9
736191167,1.3868462685,3.2125657352\N,-0.1595420705,1.3332697755,-0.45
22302794\H,-0.1028967702,1.8337715807,0.4267243583\C,1.774772315,2.784
504687,-1.0410692578\C,2.8993065031,2.9798408282,-2.0762303819\H,3.382
1201824,3.9488390641,-1.9080383853\H,2.5041851409,2.9749525477,-3.0973
922727\H,3.657526666,2.1990607342,-2.0140345912\C,0.7869700397,3.96061
87813,-1.1910797265\H,1.3215996731,4.9099194184,-1.081037854\H,0.31018
95316,3.9585507434,-2.1772513457\H,-0.0082995073,3.949228743,-0.437976
7755\C,2.3846461119,2.790653866,0.3751985688\H,1.6248830384,2.71510565
84,1.1655085401\H,2.9159442158,3.7325868354,0.5487537325\H,3.094517527
2,1.9693082066,0.5168708945\C,-1.3734823931,0.8308135204,-0.8138098533
\N,-2.3408405425,1.0707516051,0.1409349841\H,-2.1224123162,1.795846689
7,0.8144984513\C,-3.6722429153,0.6197129142,0.2286350453\C,-6.34628870
77,-0.1817627483,0.5763075794\C,-4.5914077565,1.4627833248,0.868164970
7\C,-4.0936174068,-0.6367994761,-0.2249863883\C,-5.4239186959,-1.01734
21487,-0.0527575816\C,-5.9129009696,1.0586266636,1.0424324835\H,-4.277
5860816,2.441848976,1.2154116223\H,-3.3966674364,-1.299884231,-0.71585
38118\H,-7.3795857217,-0.4857340693,0.6915724522\C,-5.8582040808,-2.39
26746795,-0.4905093553\C,-6.8803771074,1.9477472162,1.7772181303\F,-5.
1116366962,-2.8626108081,-1.5100467778\F,-5.7534041112,-3.2895656308,0
.5206549455\F,-7.1495013658,-2.407385535,-0.8895254796\F,-6.9916248115
,1.5939605879,3.080272198\F,-6.4996084107,3.2446310506,1.7564853412\F,
-8.1213692613,1.8803497818,1.2496709851\S,-1.6294862095,0.0131052177,-
2.2613320743\\Version=ES64L-G09RevD.01\State=1-A\HF=-2265.9334657\RMSD
=3.062e-09\RMSF=3.879e-06\Dipole=1.5061837,1.3993149,2.5813559\Quadrup
ole=-5.5669599,10.3516241,-4.7846643,-1.1306324,8.696064,-1.3952121\PG
=C01 [X(C25H36F6N4O1S1)]\\@
```

Nucleophile **1**

```
1\1\GINC-R187\FOpt\RB3LYP\6-31G(d,p)\C9H8N2O1S1\ROOT\08-Dec-2015\0\\#
freq=noraman rb3lyp/6-31g** opt=(maxcyc=200) scf=maxcyc=200 scrf=(smd,
solvent=toluene)\\thialzone\\0,1\C,2.1554781473,0.2865250426,-1.087641
2741\N,0.7608895514,0.2468581964,-1.2569431491\C,0.1370455655,0.142805
6312,-0.1285469919\S,1.0717769464,0.0603537612,1.3773149285\C,2.614494
6534,0.151016418,0.3843888075\O,2.9433634364,0.4184260807,-2.000445150
5\C,3.5631509442,1.2777329633,0.7972476518\H,4.4126237243,1.2886236346
,0.1081342314\H,3.940527843,1.1268027194,1.8118754788\H,3.067386483,2.
2514982769,0.7525850411\C,-1.3338590343,0.088377536,-0.0324464268\C,-4
.0405584073,-0.0352659537,0.2560712795\C,-2.128222856,0.1264854004,-1.
1841882541\C,-3.1607699297,-0.0653097693,1.3431224334\C,-3.5109969468,
0.0627724193,-1.0293982231\H,-1.6581958472,0.2038071719,-2.1574254572\
H,-3.540957238,-0.1406251873,2.3592228336\H,-4.162067137,0.0893912875,
-1.8975925336\H,-5.1115667728,-0.0878153923,0.4216019107\H,3.123020701
8,-0.8148681183,0.477055709\N,-1.8356267876,-0.0062094085,1.2097094748
\\Version=ES64L-G09RevD.01\State=1-A\HF=-930.7248031\RMSD=3.756e-09\RM
SF=1.642e-05\Dipole=-1.6832476,-0.1296111,0.7592088\Quadrupole=11.3769
612,-3.1382543,-8.2387069,0.2600011,8.9690621,0.7530011\PG=C01 [X(C9H8
N2O1S1)]\\@
```

Electrophile **2**

```
1\1\GINC-R228\FOpt\RB3LYP\6-31G(d,p)\C8H7N1O2\ROOT\08-Dec-2015\0\\# fr
eq=noraman rb3lyp/6-31g** opt=maxcyc=200 scf=maxcyc=200 scrf=(smd,solv
ent=toluene)\\Nitro-olefin\\0,1\C,-1.2918462576,0.2423683973,0.0001013
845\C,-0.1927246612,-0.5281051296,-0.0004433292\N,1.1237692805,0.06778
88413,0.0004942712\O,1.2422449181,1.2976328932,0.0017773572\O,2.076183
0956,-0.7219065727,-0.0001516785\H,-0.1459416234,-1.6073583437,-0.0015
662802\H,-1.1285640753,1.3171325925,0.0012663455\C,-2.6785138305,-0.20
96379835,-0.0007085052\C,-3.6949747019,0.7642788139,0.0000757672\C,-3.
0475694702,-1.5700876464,-0.0022366964\C,-5.0374804338,0.3940614646,-0
.0006346018\H,-3.4216752009,1.8156329434,0.0012512356\C,-4.3878067081,
-1.9366763516,-0.0029424809\H,-2.2835799191,-2.3407096134,-0.002869839
7\C,-5.386927374,-0.9569120605,-0.0021452073\H,-5.8091603691,1.1576023
494,-0.0000122151\H,-4.6584773182,-2.9879737308,-0.0041181271\H,-6.432
6748107,-1.2489794834,-0.0027037997\\Version=ES64L-G09RevD.01\State=1-
A\HF=-514.1760499\RMSD=5.485e-09\RMSF=2.370e-05\Dipole=-2.5496758,-0.5
367424,-0.0011302\Quadrupole=-6.6220613,5.777527,0.8445344,-2.3985822,
-0.0040124,0.0046836\PG=C01 [X(C8H7N1O2)]\\@
```

TS1A-*R,R*

```
1\1\GINC-R2526\FTS\RB3LYP\6-31G(d,p)\C42H51F6N7O4S2\ROOT\09-Dec-2015\0
\\# freq=noraman rb3lyp/6-31g** opt=(ts,calcfc,noeigen,maxcyc=200) scf
=maxcyc=200 scrf=(smd,solvent=toluene) iop(1/8=1)\\TS for 1st C-C addi
tion conf. 1: R(elec),R(nuc) (tol opt; cat III)\\0,1\C,-1.2494256521,-
3.3217510012,1.5412784212\O,-1.8428046469,-4.3656027817,1.8276263161\C
,-0.4497597284,-2.559354909,2.6260201828\H,0.2602087816,-3.3012465511,
3.0030594856\N,-1.2451761302,-2.7862125825,0.28985455\H,-0.7808328521,
-1.8954887562,0.1743628054\C,-1.8576129575,-3.3601722141,-0.917472701\
H,-1.8278499219,-2.5357806327,-1.639196206\C,-1.0388791819,-4.51870663
23,-1.5987836517\C,0.4270887284,-4.0672780818,-1.73737922\H,0.90505582
77,-3.9265574591,-0.7649756148\H,0.5059624251,-3.1284151269,-2.2995787
67\H,1.0027685127,-4.8233342217,-2.2814790697\C,-1.0893396649,-5.84796
52111,-0.821879708\H,-0.6607399659,-5.755668468,0.1767218786\H,-0.5232
31928,-6.6112271309,-1.3679756012\H,-2.1121938814,-6.2231834928,-0.707
5903777\C,-1.6028345021,-4.7442357847,-3.0189780114\H,-2.6271332884,-5
.1329777883,-3.0139811842\H,-0.9905264953,-5.4817460953,-3.5476638974\
H,-1.58864617,-3.8229103883,-3.6137670951\C,-3.3356732842,-3.704894339
,-0.6492774884\H,-3.712009638,-4.4685146773,-1.3305665645\H,-3.4422252
642,-4.0690386299,0.3709633969\N,-4.2271605949,-2.5055327939,-0.812865
0749\C,-5.5429373154,-2.638086815,-0.078368513\H,-5.3825345194,-2.3733
92849,0.9675433067\H,-5.8572679963,-3.6833831058,-0.1440380173\C,-6.48
38171309,-1.7168153642,-0.8438946751\H,-7.5322605662,-1.9400666394,-0.
6338491797\H,-6.2844761741,-0.6767711186,-0.570893015\C,-4.5636787147,
-2.1539368045,-2.2613046213\H,-4.1991620575,-2.9499487617,-2.910442450
6\H,-4.0326332534,-1.2325820176,-2.4996800407\C,-6.0908788583,-1.98049
98185,-2.3063102216\H,-6.5659902056,-2.8989378935,-2.6659348678\H,-6.3
808054087,-1.1705739794,-2.9791623775\N,0.355853338,-1.4792963388,2.07
16994486\H,-0.0934681591,-0.5614948431,1.9574385303\C,-1.3503559441,-2
.097985181,3.8385825763\C,-1.6645688365,-3.3266129872,4.7163591637\H,-
2.3136754954,-3.0354634606,5.5504463822\H,-0.7472925285,-3.7501540105,
5.1411523283\H,-2.1625751477,-4.1100490194,4.1428874393\C,-0.573712967
1,-1.0787483301,4.6963719503\H,-1.1349220511,-0.8748932807,5.615190671
2\H,0.4098327327,-1.4626878847,4.9875683658\H,-0.4297618321,-0.1246994
225,4.1820154774\C,-2.6714933697,-1.4628475031,3.3618077972\H,-2.51466
1573,-0.6375496995,2.6615538247\H,-3.225530459,-1.0667376469,4.2204314
766\H,-3.3121451935,-2.2088172023,2.8792439051\C,1.6929735081,-1.56133
09879,1.8353043108\N,2.2268268077,-0.3588225478,1.447331538\H,1.628746
9459,0.4762828542,1.5451807061\C,3.5112711566,-0.0572026143,0.96667182
36\C,5.9927017002,0.7744437113,-0.0865689268\C,4.0027612568,1.23410303
1,1.2250618834\C,4.2722737321,-0.9268916298,0.1762284079\C,5.498010017
3,-0.5020733052,-0.3399626041\C,5.2274126513,1.6371374954,0.7016425452
\H,3.414015762,1.9196975698,1.8230915413\H,3.9072354731,-1.9224584426,
-0.0342573279\H,6.9427620745,1.0925781922,-0.4974455989\C,-2.876719734
7,0.7725079255,-0.7296040773\N,-1.6250849053,0.3985950223,-1.234024007
5\C,-1.1329174248,1.3261053536,-2.0113431405\S,-2.1280474027,2.7583278
343,-2.2627711835\C,-3.2399420224,2.1546194918,-1.0236388251\O,-3.6235
054464,-0.0191930259,-0.1010474722\H,-3.764430723,-1.6346671385,-0.418
089522\C,-4.6985294412,2.5164493832,-1.1077982451\H,-5.2088667029,2.21
57359513,-0.1892084646\H,-5.186176099,1.9939127496,-1.941758526\H,-4.8
53728159,3.5876853737,-1.2503322896\C,0.1664670238,1.2454598213,-2.697
1820778\C,2.5445635579,1.2218132697,-4.0443273747\C,1.0876826223,0.228
0506119,-2.4162892514\C,2.2995974688,0.2225963971,-3.1040007973\C,1.56
44456269,2.1967129969,-4.2518636983\H,0.8586920508,-0.5165441079,-1.66
3879087\H,3.0478925842,-0.5366240461,-2.9002194705\H,1.7240701047,2.99
51738219,-4.9732744165\H,3.4760470658,1.2560520986,-4.5994318758\C,-2.
5294928753,3.1141587213,0.8472111455\C,-1.124120071,3.1270503441,0.886
9131861\N,-0.4195127932,2.0467121519,1.3263483704\O,-1.0295281435,0.98
3573429,1.6635278118\O,0.8454863953,2.1233573491,1.4110303967\H,-0.508
```

4752287,3.9707213224,0.6163714551\H,-3.0058722822,2.3298805887,1.42725
10525\C,-3.2501971741,4.4047915483,0.8056479118\C,-4.4370828776,4.5504
138315,1.5456047124\C,-2.7831635948,5.5097256585,0.0692621475\C,-5.128
0036836,5.7612003981,1.5618536723\H,-4.8072117657,3.7097503149,2.12585
44666\C,-3.4801878098,6.7156873746,0.0787523428\H,-1.8756749689,5.4237
222575,-0.5195868372\C,-4.6539053112,6.8480186877,0.8257004523\H,-6.03
6795544,5.8547669059,2.1493452473\H,-3.1048998442,7.5562196481,-0.4977
862961\H,-5.1918718219,7.7913940977,0.8346089122\C,6.2338672307,-1.434
5456867,-1.2612432639\C,5.7720975297,3.0034018586,1.0196060122\F,5.597
1646813,-1.5460095147,-2.4611944401\F,6.3189001993,-2.6852967204,-0.76
13922435\F,7.4896759801,-1.0174832422,-1.5273128215\F,6.7265217715,2.9
538694761,1.9800900021\F,6.3508011139,3.5738079731,-0.0642401416\F,4.8
172723195,3.8504130839,1.4577153548\S,2.586894857,-2.9938714215,2.0216
885915\N,0.4031987412,2.2168281812,-3.5976451634\\Version=ES64L-G09Rev
D.01\State=1-A\HF=-3710.8419853\RMSD=2.761e-09\RMSF=2.005e-06\Dipole=-
6.6537827,-0.2097452,-3.366587\Quadrupole=17.2215166,-9.4653241,-7.756
1925,17.5997242,-2.5011595,16.9565079\PG=C01 [X(C42H51F6N7O4S2)]\\@

TS1A-*S,S*

1\1\GINC-R2536\FTS\RB3LYP\6-31G(d,p)\C42H51F6N7O4S2\ROOT\08-Dec-2015\0
\\# freq=noraman rb3lyp/6-31g** opt=(ts,calcfc,noeigen,maxcyc=200) scf
=maxcyc=200 scrf=(smd,solvent=toluene)\\TS for 1st C-C addition conf.
2: S(elec),S(nuc) (tol opt; cat III)\\0,1\C,1.0158485385,-3.7267428083
,0.3060019318\O,1.6919049613,-4.7444672011,0.4974735651\C,-0.087709557
2,-3.7157431807,-0.7931549157\H,-0.7778829127,-4.5051658274,-0.4796912
529\N,1.144266676,-2.5895557909,1.0305560215\H,0.5802611237,-1.7901271
804,0.7593720044\C,1.88116759,-2.3739553205,2.2856050266\H,1.934191190
3,-1.2833461682,2.3691135934\C,1.0985704846,-2.8539980721,3.5681699687
\C,-0.3559862141,-2.3533319577,3.4727860153\H,-0.9087437239,-2.8514277
945,2.6729473564\H,-0.401056048,-1.2728681749,3.2925661048\H,-0.884025
6361,-2.5563869678,4.4104911061\C,1.0988316661,-4.3857352958,3.7278514
508\H,0.6636284677,-4.8879510321,2.8614466398\H,0.5135381746,-4.667466
6049,4.61051337\H,2.1089975959,-4.7859991239,3.870016884\C,1.738617494
8,-2.2096779384,4.8174243155\H,2.7448800289,-2.5871574923,5.0277049442
\H,1.1301433599,-2.4299911654,5.7005963717\H,1.7989062279,-1.119109451
2,4.7237250257\C,3.3289579307,-2.8860826674,2.2527207341\H,3.751193894
,-2.7993536314,3.2537440353\H,3.3838708192,-3.923760604,1.9267960742\N
,4.2275927282,-2.0693443254,1.3616521661\C,4.1909800991,-2.4235041906,
-0.1184753797\H,3.5676250685,-1.6716170548,-0.5999864055\H,3.735473156
7,-3.4074906326,-0.2192969817\C,5.6551447134,-2.3790317519,-0.60023585
68\H,6.0364617708,-3.3948033308,-0.7429054896\H,5.746208067,-1.8516043
098,-1.5523438812\C,5.6748553376,-2.1311313829,1.7795831825\H,5.908309
3068,-3.1687579417,2.0369742995\H,5.8110391804,-1.4987015443,2.6585710
228\C,6.4233116267,-1.6770513103,0.5336290935\H,7.4804051956,-1.950283
558,0.569923244\H,6.3537160195,-0.5894397871,0.433769629\N,-0.86802681
05,-2.4855827039,-0.7873886927\H,-0.4253067533,-1.6409368323,-1.144480
7355\C,0.400246091,-4.0776249473,-2.2457853644\C,0.9681811556,-5.50967
11706,-2.284626333\H,1.2431718029,-5.7680533907,-3.3140379016\H,0.2210
608523,-6.2373129148,-1.9490269691\H,1.844829869,-5.6216369471,-1.6468
345077\C,-0.8130105552,-4.0210795775,-3.1973208927\H,-0.5147278109,-4.
3607900948,-4.1952242069\H,-1.6241083035,-4.6699341044,-2.8519630672\H
,-1.2140776345,-3.0088736344,-3.2993884849\C,1.4682169765,-3.084053593
1,-2.7386855655\H,1.1130449172,-2.0475417981,-2.7116333477\H,1.7366368
435,-3.3044352108,-3.7780895646\H,2.3830943587,-3.1496006497,-2.143650
1184\C,-2.1768439596,-2.370147984,-0.4119742043\N,-2.6101064814,-1.075
2918643,-0.5317388515\H,-1.8669355226,-0.409416706,-0.7801451727\C,-3.
8612449162,-0.4629895918,-0.3887406491\C,-6.267337465,1.0098727665,-0.
2117784311\C,-3.8763901475,0.9300696333,-0.6082720042\C,-5.0648560553,
-1.1074372187,-0.0759081033\C,-6.2458591158,-0.362835702,0.0058444885\
C,-5.0614546344,1.6469348686,-0.5207434016\H,-2.9530038511,1.439275484
2,-0.8632700415\H,-5.0719197649,-2.1741077341,0.0976657568\H,-7.192105
1579,1.5691812357,-0.1506475955\C,3.5137247339,1.3120607096,0.71545074
86\N,3.4353428663,0.8601419801,-0.6042412254\C,3.4214319428,1.85641395
44,-1.4490677248\S,3.5225398269,3.4730183392,-0.7633226355\C,3.3542839
932,2.7520146591,0.8449665478\O,3.7098302728,0.5397343035,1.6901447015
\H,3.9496490436,-1.0383955386,1.4430540095\C,3.9253973281,3.4552311098
,2.0434820277\H,3.6249286298,2.9306431336,2.9544919141\H,3.5970322241,
4.4940782489,2.1162480744\H,5.0229880012,3.4497909996,2.0128113058\C,3
.3518579301,1.7113473121,-2.911697518\C,3.2335848972,1.5786909953,-5.6
400166394\C,3.188463704,0.4585707505,-3.516951851\C,3.1291924925,0.398
0648797,-4.9067952571\C,3.3951073638,2.7804742037,-4.9443785616\H,3.10
22940419,-0.4305731663,-2.9051008988\H,2.9978145278,-0.5559111369,-5.4
083186836\H,3.480472593,3.7221685669,-5.4823219439\H,3.1895083823,1.57
62954582,-6.7240823519\C,1.1098952312,2.8023049541,1.1370603145\C,0.46
68257665,2.3323430245,-0.0225953194\N,0.2137412708,1.0044988827,-0.191
8649519\O,0.5792872568,0.161651533,0.6735014765\O,-0.4250486311,0.6232
322782,-1.2393300793\H,0.0888847191,2.9566785211,-0.8167322718\H,1.200

2137909,2.0793048308,1.94125414\C,0.930165457,4.2052586432,1.554847777
9\C,0.8842622772,4.5067868656,2.928511635\C,0.7690736788,5.2591247298,
0.6348467222\C,0.673961038,5.8120604308,3.3696767097\H,0.9985497954,3.
7042826597,3.6516924533\C,0.5677373189,6.5637831889,1.0777399269\H,0.8
094224362,5.0592091585,-0.4303484037\C,0.5167398418,6.846127392,2.4455
062158\H,0.6314944063,6.0205816898,4.4347092484\H,0.4467676066,7.36307
07278,0.3524627115\H,0.3538433451,7.8642772911,2.7864519847\C,-7.51481
15181,-1.1026915143,0.3377486562\C,-5.0508980167,3.1396914384,-0.70595
21086\F,-7.7779092498,-2.0736882391,-0.5676376078\F,-7.4374606391,-1.7
118700056,1.5436233014\F,-8.5930503938,-0.2884046249,0.3719672142\F,-5
.0616597656,3.7925309952,0.4816767995\F,-3.9593678233,3.5680172099,-1.
3774490829\F,-6.1378802626,3.5673785053,-1.3888464933\S,-3.0940101015,
-3.6822484954,0.1404994963\N,3.4535620403,2.8536921205,-3.6145975558\\
Version=ES64L-G09RevD.01\State=1-A\HF=-3710.8440986\RMSD=2.040e-09\RMS
F=5.596e-07\Dipole=6.5299047,0.8539562,-0.1043642\Quadrupole=-9.041444
6,-15.4603478,24.5017925,-26.1317886,3.2768767,3.9374612\PG=C01 [X(C42
H51F6N7O4S2)]\\@

TS1B-*R,R*

1\1\GINC-R3133\FTS\RB3LYP\6-31G(d,p)\C42H51F6N7O4S2\ROOT\14-Dec-2015\0
\\# freq=noraman rb3lyp/6-31g** opt=(ts,calcfc,noeigen,maxcyc=200) scf
=maxcyc=200 scrf=(smd,solvent=toluene) iop(1/8=1)\\TS for 1st C-C addi
tion conf. 2: R(elec),R(nuc) (tol opt; cat III)\\0,1\C,-1.0268779147,-
3.5958345421,-0.4230891725\O,-1.5544048367,-4.6817388658,-0.6873851355
\C,-0.0501149664,-3.5001277816,0.7764911213\H,0.6387244583,-4.33796733
66,0.6446746296\N,-1.2641244437,-2.454034009,-1.1227133582\H,-0.776437
5178,-1.6067730031,-0.8472267112\C,-2.1454375587,-2.2998791434,-2.2919
175902\H,-2.3003350157,-1.2178572758,-2.3570236978\C,-1.4976923284,-2.
7057555627,-3.667340745\C,-0.0712856307,-2.1272417023,-3.7330153773\H,
0.6022452581,-2.6270292745,-3.0332904283\H,-0.0566002703,-1.0549405656
,-3.5066449305\H,0.3409480954,-2.2611349594,-4.7386353711\C,-1.4373222
935,-4.2303352898,-3.8786680242\H,-0.8789687049,-4.7290670509,-3.08458
2344\H,-0.9475596136,-4.4515425459,-4.8337026474\H,-2.4344310886,-4.68
37286353,-3.9158049313\C,-2.324736349,-2.0645053265,-4.802055738\H,-3.
3537353899,-2.4380160234,-4.8466466858\H,-1.8661110238,-2.2893435135,-
5.7703705347\H,-2.3651146068,-0.9729718972,-4.7046304428\C,-3.51345765
55,-2.9646355472,-2.040904375\H,-4.0371865055,-3.1700488965,-2.9742300
338\H,-3.3622626565,-3.9026915215,-1.5103477409\N,-4.4377777525,-2.109
9956277,-1.2103532352\C,-5.4320915211,-2.900236137,-0.400841285\H,-4.9
051906024,-3.411007976,0.4063237222\H,-5.8674958245,-3.6466183802,-1.0
716883353\C,-6.4531336816,-1.8574302725,0.0585970593\H,-7.4395191895,-
2.3082838061,0.1852492368\H,-6.1407937884,-1.4301895074,1.0127491339\C
,-5.2899949962,-1.1419237577,-1.9951053348\H,-5.6587510401,-1.67887096
98,-2.8723077857\H,-4.6690530187,-0.3066054827,-2.3175029192\C,-6.4317
320156,-0.7545240228,-1.0383508111\H,-7.3739813091,-0.6971896879,-1.58
79445336\H,-6.2522507934,0.2238639111,-0.5887229946\N,0.7664280183,-2.
2972464947,0.7244383706\H,0.3344015602,-1.4121008421,0.986251783\C,-0.
7637120427,-3.6852918423,2.1682328201\C,-1.2811727611,-5.1308998083,2.
302548465\H,-1.7610433183,-5.2618782356,3.2794718092\H,-0.4607443986,-
5.8538586909,2.2343493817\H,-2.0039724888,-5.3785267136,1.5239266272\C
,0.2717950286,-3.4455984713,3.2845614298\H,-0.1851494403,-3.6381373567
,4.2617666033\H,1.1314448976,-4.1159000098,3.1805780092\H,0.6543505862
,-2.4213509478,3.2867909497\C,-1.9436238993,-2.7074043154,2.3276041389
\H,-1.6652365836,-1.6640113044,2.1554035467\H,-2.3597651447,-2.7776752
082,3.3386780898\H,-2.753445894,-2.9441881172,1.6302223111\C,2.0525229
421,-2.2417135832,0.2724895044\N,2.4137696423,-0.9500332776,-0.0195611
447\H,1.6108238866,-0.3395672393,-0.2303810639\C,3.6550474468,-0.40259
66911,-0.3629220536\C,6.0442659412,0.9331323781,-1.0394814964\C,3.6297
102861,0.7549935673,-1.1574827925\C,4.8889352131,-0.8889351707,0.09277
35392\C,6.0626706436,-0.2192841797,-0.2539909841\C,4.8116260095,1.4159
842794,-1.4795416842\H,2.6764871577,1.1346306782,-1.5093864208\H,4.927
9512469,-1.7709171654,0.7153517315\H,6.9628845072,1.4504885612,-1.2863
01911\C,-0.233318,1.2706287743,0.2570880369\N,-0.3745320886,0.80305303
53,1.568180898\C,-0.5424366178,1.7877194325,2.4112139065\S,-0.54514980
56,3.4098239739,1.7319620933\C,-0.491033217,2.6933514321,0.1163560769\
O,0.0984897971,0.5084669816,-0.6889005446\H,-3.8650631699,-1.502781157
5,-0.5566791805\C,0.1365346888,3.4520498057,-1.0190392383\H,-0.0576703
828,2.9318682403,-1.9610127206\H,1.225787303,3.5100941973,-0.899507471
5\H,-0.2446245144,4.4720055149,-1.1019730478\C,-0.6877303226,1.6396324
353,3.867742865\C,-0.9422987996,1.5087591521,6.587166069\C,-0.65811607
91,0.3833184947,4.4855636584\C,-0.7868529211,0.3236543437,5.8706751625
\C,-0.9583646048,2.7147214738,5.8798444273\H,-0.5383562575,-0.50997825
89,3.8871303326\H,-0.766288361,-0.6346866994,6.3805291538\H,-1.0741930
365,3.6604879708,6.4049981647\H,-1.0470962448,1.5069102992,7.667082192
4\C,-2.6831189544,2.5453816384,-0.418516486\C,-3.420343274,1.992904818
2,0.6415897705\N,-3.5899934534,0.6422670075,0.7687948414\O,-2.99607406
35,-0.1446448323,-0.0570344635\O,-4.3412508298,0.1785158664,1.66185442

05\H,-3.9528979789,2.5653631706,1.3852836669\H,-2.4231194814,1.8554607
836,-1.2146816203\C,-2.9417484917,3.9375780853,-0.8365372228\C,-2.8516
375777,4.2689705493,-2.2010437664\C,-3.3089763165,4.9495690665,0.07119
88035\C,-3.1295141813,5.5600635518,-2.6468766445\H,-2.5701728134,3.500
9239671,-2.9159093445\C,-3.5780376349,6.2410444912,-0.3749577572\H,-3.
3736060112,4.7285001506,1.1310284197\C,-3.4927840914,6.5520733112,-1.7
348234534\H,-3.0605614184,5.7909835502,-3.7059093706\H,-3.8561737414,7
.0083763035,0.3416516395\H,-3.7066060747,7.5596057056,-2.0790651567\C,
7.3842487947,-0.7846445212,0.1943434067\C,4.759651238,2.6283696698,-2.
3677397888\F,7.2892320863,-1.4230648133,1.3814744624\F,7.8736623094,-1
.6823356927,-0.6936744338\F,8.3255297942,0.1784246665,0.3303621142\F,4
.8525051812,2.2985491759,-3.6786883034\F,3.6010880724,3.3162947561,-2.
2253694373\F,5.769626383,3.4889251829,-2.1113743603\S,3.0370559422,-3.
6016942212,0.0595603102\N,-0.8331697299,2.7873673588,4.5548847073\\Ver
sion=ES64L-G09RevD.01\State=1-A\HF=-3710.8371502\RMSD=5.153e-09\RMSF=2
.423e-06\Dipole=-7.0887648,1.0666892,-0.4357827\Quadrupole=-3.4679904,
-19.1018329,22.5698233,20.8968404,19.795618,1.6488922\PG=C01 [X(C42H51
F6N7O4S2)]\\@

TS1B-*S,S*

1\1\GINC-R2649\FTS\RB3LYP\6-31G(d,p)\C42H51F6N7O4S2\ROOT\09-Dec-2015\0
\\# freq=noraman rb3lyp/6-31g** opt=(ts,calcfc,noeigen,maxcyc=200) scf
=maxcyc=200 scrf=(smd,solvent=toluene)\\TS for 1st C-C addition conf.
1: S(elec),S(nuc) (tol opt; cat III)\\0,1\C,0.870988939,-3.1308271147,
-1.9638942971\O,1.5060553727,-4.0579824836,-2.4742611134\C,-0.11268449
69,-2.2884894137,-2.8141336201\H,-0.8575332408,-3.0076475245,-3.168435
2361\N,0.9685370738,-2.7937712606,-0.6502250747\H,0.4039754789,-2.0142
659844,-0.3433791896\C,1.7489386406,-3.4880891003,0.3843327377\H,1.773
2521619,-2.7772004739,1.2186612001\C,1.0516306974,-4.7723361692,0.9703
470707\C,-0.3972097314,-4.4109587457,1.3497378509\H,-0.9980853208,-4.1
518585564,0.4749605207\H,-0.4325722578,-3.5661818823,2.0494039652\H,-0
.8800095878,-5.2619519928,1.8412115166\C,1.0369528256,-5.9582329853,-0
.0123650007\H,0.4881153082,-5.7257707663,-0.9258458456\H,0.556864438,-
6.8209408988,0.4634297181\H,2.046107685,-6.2678392044,-0.3042895172\C,
1.7883771185,-5.1948895591,2.259039696\H,2.8133480514,-5.5347590763,2.
0737066854\H,1.261131947,-6.0321053844,2.7279066385\H,1.8240352948,-4.
3818921333,2.9938664213\C,3.2032209281,-3.7050257137,-0.0894383178\H,3
.6330106595,-4.6215319439,0.3162863438\H,3.2275852443,-3.7741668687,-1
.1748322311\N,4.1189182019,-2.5857665212,0.3290695157\C,5.3964428045,-
2.5533385816,-0.4902586478\H,5.2194856784,-1.9353588233,-1.3717734099\
H,5.6122063257,-3.5772980507,-0.8061682646\C,6.4576007396,-2.029799938
5,0.47117995\H,7.4648802319,-2.2910842864,0.1379974495\H,6.3821612944,
-0.9433616426,0.5602135428\C,4.5322167296,-2.6311127974,1.801591314\H,
4.056921206,-3.4903482415,2.2723721937\H,4.1749781737,-1.7050226016,2.
2481757454\C,6.0646620261,-2.6985352144,1.7968237518\H,6.4046816522,-3
.7400266885,1.8147075126\H,6.4841201755,-2.1927260082,2.6694175117\N,-
0.8346693794,-1.3125426033,-2.0126853853\H,-0.3847433036,-0.4091869315
,-1.8325380984\C,0.5622785717,-1.6289005311,-4.0772222758\C,0.89709614
35,-2.720477347,-5.1133923583\H,1.3617521412,-2.2641150228,-5.99524905
17\H,-0.0106555575,-3.2366386322,-5.446738518\H,1.5768455607,-3.469040
0634,-4.7055664891\C,-0.4335908945,-0.6449243208,-4.7247360399\H,-0.02
49664855,-0.2863768518,-5.6760334373\H,-1.3941437369,-1.1263692548,-4.
9373099337\H,-0.624953455,0.2309223259,-4.0983640461\C,1.8469022868,-0
.8716145794,-3.6911633381\H,1.6687654155,-0.1246107306,-2.9109989116\H
,2.2510543148,-0.3468668341,-4.5641375189\H,2.6225944651,-1.555500048,
-3.3322677237\C,-2.1316115364,-1.4246888583,-1.6110196576\N,-2.5496668
177,-0.2932389833,-0.9587489604\H,-1.8238880459,0.4232626958,-0.855155
9244\C,-3.7662625394,0.0050771131,-0.3243040026\C,-6.1162289337,0.7927
326782,1.0125155205\C,-3.7019294376,0.8317444556,0.8067964454\C,-5.018
8517277,-0.417175689,-0.7931335613\C,-6.1731187493,-0.0276816879,-0.11
49468469\C,-4.8686259927,1.2213439664,1.4623241577\H,-2.7334742543,1.1
447786821,1.1834752743\H,-5.0824644567,-1.0480098566,-1.6675941216\H,-
7.0213763819,1.0811627275,1.5329507064\C,0.2766900213,1.8900942027,-0.
2441647281\N,0.3426903813,1.2006512512,0.9703896913\C,0.7533395975,1.9
784636959,1.9335904445\S,1.1057759718,3.6520044781,1.5076269733\C,0.84
45884176,3.2289676686,-0.191223101\O,-0.2231662365,1.3864296945,-1.280
0895161\H,3.6416997041,-1.6554221333,0.1620460893\C,0.344175537,4.2800
44108,-1.1434185684\H,0.3285185315,3.8798378548,-2.1597791171\H,0.9629
473132,5.1798872203,-1.1346860836\H,-0.6829129223,4.5729373475,-0.8906
403317\C,0.895880758,1.5449397039,3.3317457798\C,1.1610472024,0.849485
5255,5.9598126641\C,0.5930508687,0.2303836673,3.711434969\C,0.72995823
15,-0.1172125516,5.0522145426\C,1.4368786531,2.134135704,5.4825711931\
H,0.2521604204,-0.4763756526,2.9640391563\H,0.4998733434,-1.1250213763
,5.3848124068\H,1.7716851195,2.9148009385,6.1622179763\H,1.279277359,0
.6215267588,7.0139883093\C,2.8724385484,2.6236745992,-0.9188022706\C,3
.6220131293,2.0965981128,0.1490024554\N,3.6729656806,0.7687735099,0.44
38439522\O,3.0177088704,-0.0911139929,-0.250675924\O,4.4177410473,0.38

1755755,1.3919390625\H,4.2494969011,2.6909735548,0.7946132476\H,2.4565
24185,1.8934624711,-1.6052129631\C,3.3072108391,3.8913932417,-1.542952
9607\C,3.157855775,4.0520694049,-2.9320757249\C,3.8850718747,4.9441878
408,-0.8085313032\C,3.5838077132,5.2162097995,-3.5698592791\H,2.713608
1223,3.2490196601,-3.5136036005\C,4.3029335148,6.1098337967,-1.4461376
029\H,3.9982539917,4.8568912653,0.2673886314\C,4.1573260336,6.25064386
91,-2.8292340263\H,3.4661652669,5.3144747835,-4.6451380543\H,4.7439789
127,6.9120148998,-0.8615064949\H,4.4868629825,7.1598931811,-3.32322191
17\C,-7.5217377013,-0.4304792079,-0.6499347249\C,-4.779136293,2.150236
7042,2.6424816817\F,-7.4617037506,-1.5346500338,-1.4217439927\F,-8.069
3746519,0.5513786122,-1.4086013285\F,-8.4024907759,-0.6798705635,0.347
1857564\F,-3.6304404704,1.9820357488,3.3359427274\F,-4.8160799961,3.45
02831257,2.2618232882\F,-5.8020898038,1.969648852,3.5078561891\S,-3.07
25411061,-2.8131371949,-1.8566030575\N,1.3093362058,2.4822729131,4.201
6764882\\Version=ES64L-G09RevD.01\State=1-A\HF=-3710.838464\RMSD=2.589
e-09\RMSF=2.336e-06\Dipole=6.1762029,-0.8244549,2.0622529\Quadrupole=-
9.501287,1.4342856,8.0670014,-22.3064793,-0.9742518,-27.6542337\PG=C01
[X(C42H51F6N7O4S2)]\\@

int1A-*R,R*

1\1\GINC-R2851\FOpt\RB3LYP\6-31G(d,p)\C42H51F6N7O4S2\ROOT\09-Dec-2015\
0\\# freq=noraman rb3lyp/6-31g** opt=maxcyc=200 scf=maxcyc=200 scrf=(s
md,solvent=toluene)\\Intm 1 after conj. addition conf. 1: R(elec),R(nu
c) (tol opt; cat III)\\0,1\C,-2.0621530958,-3.0715646977,1.6208730863\
O,-2.9578835744,-3.8802330245,1.879925032\C,-1.1865747128,-2.462245921
3,2.7458989095\H,-0.6354107166,-3.315952282,3.1542315496\N,-1.76204462
23,-2.6731369714,0.3524871156\H,-1.0074346945,-2.0061870702,0.26954421
84\C,-2.3302232102,-3.1827379823,-0.9032542177\H,-2.0017715416,-2.4553
17672,-1.6539111422\C,-1.7471817151,-4.561511805,-1.3908953468\C,-0.21
0583612,-4.5005066592,-1.311325126\H,0.1454123931,-4.3939176925,-0.283
7577759\H,0.1896784326,-3.6654411366,-1.8999492096\H,0.2224116764,-5.4
208896982,-1.7167690334\C,-2.251094213,-5.7603191836,-0.5652587272\H,-
1.9689108028,-5.6813973555,0.4854824372\H,-1.8226222545,-6.6859945658,
-0.9655612976\H,-3.3410094569,-5.8635751671,-0.6068796707\C,-2.1398064
719,-4.7644623056,-2.8706426502\H,-3.2185971494,-4.8914246095,-3.01414
34923\H,-1.6633091363,-5.6704825092,-3.2584492372\H,-1.8089320048,-3.9
282234438,-3.4985396187\C,-3.8687039975,-3.1456422987,-0.8409814636\H,
-4.327437378,-3.8566272749,-1.5282322273\H,-4.1892297417,-3.3818123943
,0.1718021191\N,-4.4296111159,-1.7926733079,-1.2004671638\C,-5.8485188
363,-1.5711018156,-0.6747973546\H,-5.7638094642,-0.9415780941,0.211579
7885\H,-6.2560876399,-2.5382001015,-0.3803567173\C,-6.6332210664,-0.91
30437883,-1.8236110145\H,-7.2803487899,-1.652104063,-2.3054161041\H,-7
.2679052324,-0.1019463971,-1.4610075495\C,-4.4698905768,-1.4902513525,
-2.6846803401\H,-4.748898769,-2.4121951124,-3.2011737858\H,-3.47801223
91,-1.1742989362,-3.0084064065\C,-5.5547113751,-0.4258817806,-2.805919
6475\H,-5.9193586158,-0.3392834885,-3.8319371568\H,-5.1603704707,0.545
2301125,-2.4960473072\N,-0.1897690119,-1.5437666345,2.2198233127\H,-0.
5085522723,-0.5883967264,1.9763813859\C,-2.0246394963,-1.8219234984,3.
9201282408\C,-2.6651892999,-2.9439839832,4.7622924207\H,-3.2417030469,
-2.5077405959,5.586447816\H,-1.8980784408,-3.5918705902,5.2024516552\H
,-3.3293630018,-3.567709342,4.1625930641\C,-1.0716686125,-1.0198287217
,4.8300560928\H,-1.6169108039,-0.6726004043,5.71504026\H,-0.2365103223
,-1.6379855735,5.1774632742\H,-0.6551383493,-0.1404432063,4.3310984354
\C,-3.1251852852,-0.8858812028,3.3833351142\H,-2.716247683,-0.10134159
11,2.7385977158\H,-3.6418002012,-0.3979280734,4.2178442179\H,-3.877637
7591,-1.4456556947,2.8170350391\C,1.1379948189,-1.8095681978,2.0952466
605\N,1.8413763231,-0.6815839179,1.7543612749\H,1.3445336288,0.2183721
143,1.9346681676\C,3.1397686963,-0.5132612549,1.2667347385\C,5.6866818
46,0.0838605877,0.1895665449\C,3.6512509495,0.7998359895,1.3036760124\
C,3.9201186035,-1.5214304712,0.6817826618\C,5.1725599634,-1.2093718219
,0.1484877168\C,4.9066044095,1.0827569979,0.7777585452\H,3.0483146417,
1.589380008,1.7403935055\H,3.5520096753,-2.5373096979,0.653329173\H,6.
6646988477,0.3081829841,-0.2162265633\C,-2.2675926586,1.1982118662,-0.
9857913386\N,-1.2475953938,0.4930011785,-1.6294640774\C,-0.3642963563,
1.2859210186,-2.1603192753\S,-0.5964630452,3.0307113468,-1.9889726689\
C,-2.1350949619,2.7444846082,-1.0099533012\O,-3.308930047,0.6702816116
,-0.6003399492\H,-3.8356213459,-1.0362715457,-0.7903097326\C,-3.355842
7296,3.2881678086,-1.7763765476\H,-4.2623279249,3.0903202923,-1.197534
3729\H,-3.4530211014,2.8000005824,-2.7503707789\H,-3.2763768779,4.3635
655574,-1.9393519241\C,0.8213684631,0.7884790807,-2.8701492956\C,3.064
6504146,-0.0434086408,-4.1905300747\C,1.4322638578,1.5301835387,-3.895
1634216\C,1.3942835737,-0.4446179402,-2.5228069659\C,2.5408927679,-0.8
614663694,-3.1867747727\H,1.0075017017,2.4813327364,-4.210526983\H,0.9
612403188,-1.0336104907,-1.7222136352\H,3.042130945,-1.7841626404,-2.9
16207948\H,3.9653386174,-0.3394117564,-4.7238731649\C,-2.0603806566,3.
3548090783,0.4663773807\C,-0.7800306221,3.1365600514,1.2120371926\N,-0
.4117122003,1.93351992,1.5927474015\O,-1.115283137,0.8998201773,1.2154

946235\O,0.6282776783,1.7320780103,2.3369694557\H,-0.1448375946,3.9487
138774,1.5308126542\H,-2.8594222891,2.7960461228,0.9737821088\C,-2.420
1414634,4.8359925837,0.5016780192\C,-3.6486928347,5.248378691,1.033356
7409\C,-1.5371113909,5.817397026,0.0248451566\C,-3.9920886878,6.600691
2401,1.0838008161\H,-4.3386042906,4.502933312,1.4205990285\C,-1.879074
1464,7.1692192827,0.0704337271\H,-0.5693366117,5.5278396255,-0.3754843
866\C,-3.1086931256,7.565961626,0.5998089183\H,-4.9476399705,6.8981823
327,1.5064340071\H,-1.1803936291,7.9129310828,-0.3019467696\H,-3.37234
75704,8.6188163513,0.6394170754\C,5.9319818899,-2.3051673111,-0.546975
4412\C,5.4060231238,2.5021788231,0.7789846789\F,5.3618309285,-2.620233
8307,-1.7446370749\F,5.9561365209,-3.44858107,0.1706406131\F,7.2123130
462,-1.9651679861,-0.808299761\F,5.0656926912,3.1560404923,-0.36021186
28\F,4.9022501518,3.2227971812,1.80397118\F,6.7549652544,2.5627195081,
0.8676059438\S,1.8072722905,-3.3525831511,2.3513130808\N,2.5211634024,
1.1270904743,-4.5522642332\\Version=ES64L-G09RevD.01\State=1-A\HF=-371
0.8541121\RMSD=4.023e-09\RMSF=3.522e-06\Dipole=-7.5574469,0.2726446,-4
.6588921\Quadrupole=23.1053737,-8.6592515,-14.4461222,7.0969168,15.197
5033,19.669009\PG=C01 [X(C42H51F6N7O4S2)]\\@

int1A-*S,S*
1\1\GINC-R3044\FOpt\RB3LYP\6-31G(d,p)\C42H51F6N7O4S2\ROOT\09-Dec-2015\
0\\# freq=noraman rb3lyp/6-31g** opt=maxcyc=200 scf=maxcyc=200 scrf=(s
md,solvent=toluene)\\Intm 1 after conj. addition conf. 2: S(elec),S(nu
c) (tol opt; cat III)\\0,1\C,0.6496699057,-3.7208848616,-0.049112754\O
,1.2504327357,-4.7961857474,0.0921116979\C,-0.3358053849,-3.5331160611
,-1.2370775926\H,-1.0617692344,-4.3429570377,-1.11158297\N,0.789054672
3,-2.6731317852,0.7986482783\H,0.2748097617,-1.8051270962,0.615824133\
C,1.3911028312,-2.6725668421,2.1398201298\H,1.5124621605,-1.606490371,
2.3589808905\C,0.4345515925,-3.2226648974,3.2714676727\C,-0.9761333205
,-2.6419460161,3.0526489197\H,-1.4541357156,-3.0561077177,2.1620905574
\H,-0.9562030454,-1.5521492588,2.9462834819\H,-1.6133192747,-2.8863237
31,3.9091725705\C,0.3502894254,-4.7612614775,3.2860099451\H,0.03989247
89,-5.1617295448,2.3187047686\H,-0.3805565206,-5.084362963,4.035805820
4\H,1.3068263346,-5.2275987922,3.5489358327\C,0.9432633356,-2.72587001
81,4.6428405367\H,1.915029343,-3.1480442768,4.9212937968\H,0.238597611
7,-3.0200228078,5.4276251953\H,1.0272931004,-1.633212429,4.669581013\C
,2.7834525448,-3.3157275977,2.2109995805\H,3.1076755114,-3.3260718947,
3.2513627609\H,2.7820183236,-4.3310397371,1.8193754726\N,3.8483225238,
-2.5435585245,1.4645170518\C,3.9749771888,-2.8593504863,-0.0275348268\
H,3.6440825404,-1.971022516,-0.5647208121\H,3.3123570966,-3.6919280362
,-0.2559445696\C,5.4622020263,-3.1634639986,-0.2618249977\H,5.64160233
85,-4.2427084154,-0.2176732593\H,5.7913372198,-2.8133341607,-1.2427560
266\C,5.2277421462,-2.7260814436,2.0652769374\H,5.3022111592,-3.760257
2022,2.4116241651\H,5.3259378023,-2.0514281064,2.9174099069\C,6.174737
6462,-2.4599124833,0.9027831891\H,7.1759346785,-2.8503367829,1.0990486
916\H,6.2521747695,-1.3835943809,0.7160577436\N,-1.0854144658,-2.29504
1058,-1.1381933246\H,-0.5858063596,-1.4065509412,-1.2779925868\C,0.306
9947281,-3.6971694069,-2.6679173219\C,0.8377790962,-5.1304117372,-2.86
23252833\H,1.2563856464,-5.2346820963,-3.870423711\H,0.0309434608,-5.8
650900851,-2.7619274408\H,1.6071105493,-5.3868838019,-2.1335889489\C,-
0.7878910498,-3.4489026272,-3.725755283\H,-0.3894486682,-3.6615420446,
-4.7242122438\H,-1.6542686097,-4.0981152155,-3.565504614\H,-1.13838041
97,-2.4134008095,-3.7218737089\C,1.4480987269,-2.685906816,-2.88808281
72\H,1.1351268332,-1.6533712094,-2.6946462988\H,1.7949276962,-2.730053
1256,-3.9267027671\H,2.3084261052,-2.9059319038,-2.2497592659\C,-2.418
2712013,-2.1777897038,-0.8901072717\N,-2.786037878,-0.855841941,-0.861
6043828\H,-1.98242304,-0.214323935,-0.9629290557\C,-4.0145048783,-0.20
33354949,-0.7548635275\C,-6.3681882335,1.3593398098,-0.6110030712\C,-3
.9697044385,1.1962274973,-0.9325244549\C,-5.2526201057,-0.8063446371,-
0.4937162093\C,-6.40615675,-0.0184634567,-0.4289406873\C,-5.1285726601
,1.9563511878,-0.8629110462\H,-3.0181447424,1.6747267957,-1.1395309937
\H,-5.3049227745,-1.8767801451,-0.3516834126\H,-7.2722680661,1.9529754
846,-0.566133604\C,3.508273875,1.0940771586,1.0431114765\N,4.263612861
1,0.9562009781,-0.1090500583\C,4.3946851621,2.0975110103,-0.7323981095
\S,3.6393676773,3.528222299,-0.0621192705\C,2.9512487025,2.5042983797,
1.323851441\O,3.303624774,0.1706675796,1.8298651226\H,3.6307293074,-1.
5250895382,1.5449683622\C,3.4967015482,2.9963784586,2.6737496282\H,3.1
401850485,2.330898761,3.4654819157\H,3.1551523278,4.0097219861,2.89045
63562\H,4.5905548082,2.9896790523,2.6840255909\C,5.1601923022,2.237206
6407,-1.9828121387\C,6.5403090886,2.627026213,-4.2997473055\C,5.761438
2906,1.1313322184,-2.5935291752\C,6.4652468189,1.3377314665,-3.7782895
597\C,5.9043782521,3.6679979816,-3.6126807536\H,5.6673995547,0.1502556
277,-2.1448894144\H,6.9443065518,0.5060348234,-4.2850809596\H,5.942877
7155,4.6859990386,-3.992520852\H,7.0775165858,2.831224807,-5.220008577
5\C,1.374612973,2.4296476749,1.2940022879\C,0.8470504726,1.9379976423,
-0.0222170782\N,0.1797544374,0.8022704845,-0.1153827224\O,-0.027303266
7,0.0244020119,0.892468623\O,-0.3106278532,0.4482813696,-1.283682995\H

,0.8960445425,2.5143746406,-0.933805995\H,1.133512701,1.6511543246,2.0
235030052\C,0.7101113111,3.7253307576,1.757421029\C,0.1822813408,3.805
6762698,3.0540322541\C,0.5960637696,4.8480972906,0.923057018\C,-0.4301
909924,4.9736268148,3.5110235781\H,0.2437159906,2.9402357904,3.7083783
605\C,-0.0111864277,6.0184586804,1.3791025809\H,0.9695524775,4.8098018
268,-0.0955127662\C,-0.5250910057,6.0866403259,2.6753434718\H,-0.83756
27898,5.0098576215,4.5174153726\H,-0.0909872571,6.8749244901,0.7157390
416\H,-1.0029256279,6.9963856473,3.0270015153\C,-7.711027503,-0.714482
449,-0.1489262394\C,-5.0588823953,3.451333654,-1.0097321325\F,-7.98587
94496,-1.6592157288,-1.0784256501\F,-7.6942778906,-1.3475359404,1.0478
887438\F,-8.7594590865,0.1395068043,-0.130127902\F,-5.1036122226,4.078
3319087,0.1914201775\F,-3.9258264701,3.8593264491,-1.6215075432\F,-6.1
00082668,3.9328767027,-1.7299471305\S,-3.4456927738,-3.5103334044,-0.6
623517505\N,5.2284816009,3.4838733133,-2.480165471\\Version=ES64L-G09R
evD.01\State=1-A\HF=-3710.8636039\RMSD=5.242e-09\RMSF=8.243e-06\Dipole
=10.2658765,0.7845393,0.7609327\Quadrupole=10.7150398,-23.3844671,12.6
694274,-23.4973925,-14.8085621,-11.2147161\PG=C01 [X(C42H51F6N7O4S2)]\
\@

int1B-*R,R*

1\1\GINC-R2844\FOpt\RB3LYP\6-31G(d,p)\C42H51F6N7O4S2\ROOT\09-Dec-2015\
0\\# freq=noraman rb3lyp/6-31g** opt=maxcyc=200 scf=maxcyc=200 scrf=(s
md,solvent=toluene)\\Intm 1 after conj. addition conf. 2: R(elec),R(nu
c) (tol opt; cat III)\\0,1\C,-0.8482079714,-3.4573941896,1.5117919293\
O,-1.2943895085,-4.5078746734,1.9788488913\C,0.0656606131,-2.558769061
3,2.3877130158\H,0.7931964921,-3.2616742064,2.8010172564\N,-1.09136494
53,-3.0383325514,0.2432749805\H,-0.7266842964,-2.1356442285,-0.0232851
949\C,-1.8592977039,-3.7484101155,-0.7943541149\H,-1.9942655573,-2.989
8861666,-1.5712469197\C,-1.0892498804,-4.9207970268,-1.5062518109\C,0.
3385021241,-4.4508076,-1.8419478482\H,0.9441909329,-4.3113316327,-0.94
37513594\H,0.334364192,-3.5057561262,-2.3987457611\H,0.8419624854,-5.1
966537832,-2.4662525116\C,-1.0126355474,-6.2086437013,-0.6641338951\H,
-0.5021434438,-6.044402877,0.2854675075\H,-0.4682940901,-6.9801422932,
-1.2206548884\H,-2.0051661437,-6.6140141203,-0.4381984005\C,-1.8135971
357,-5.2283628269,-2.8351294255\H,-2.8430416736,-5.5733461105,-2.68981
61204\H,-1.2861768023,-6.023280884,-3.3726001728\H,-1.840165978,-4.351
381085,-3.4933256503\C,-3.2605110516,-4.1373187806,-0.2717805669\H,-3.
6452601532,-5.0300094627,-0.7683481255\H,-3.2026271842,-4.3459015198,0
.7949832428\N,-4.2658020349,-3.0452086595,-0.4834791805\C,-5.408641246
2,-3.0719995163,0.491802898\H,-5.053947655,-2.6655712735,1.4376186712\
H,-5.7051289216,-4.117200897,0.6211156353\C,-6.5159775907,-2.227006390
5,-0.1647473587\H,-7.4818402195,-2.7305380424,-0.0759589596\H,-6.58356
29392,-1.2563427868,0.3266883249\C,-4.9235933189,-3.0323188982,-1.8332
720129\H,-5.2685265994,-4.0488711206,-2.0483924767\H,-4.1992218597,-2.
7281358567,-2.5901202683\C,-6.0851334811,-2.0549305148,-1.6479913879\H
,-6.8926894375,-2.2628419115,-2.3536373542\H,-5.7345808957,-1.03576729
09,-1.8254586153\N,0.8271770079,-1.5924536936,1.6039771296\H,0.3927771
238,-0.6918718363,1.4277797742\C,-0.6621414784,-1.8628405697,3.5954033
018\C,-1.341265028,-2.9204937724,4.487640684\H,-1.7627107916,-2.427158
568,5.3707531559\H,-0.6257303574,-3.6737105468,4.8335729202\H,-2.14571
91534,-3.4457604068,3.9714599599\C,0.3994637864,-1.1364031909,4.446767
732\H,-0.0753224399,-0.6894789288,5.3271806242\H,1.1736559655,-1.82695
46229,4.7987458903\H,0.8932767364,-0.3309295482,3.8947907765\C,-1.7164
095391,-0.8466930213,3.1205292359\H,-1.2704552986,0.0176840952,2.61453
18943\H,-2.2597252378,-0.4457037593,3.9834178262\H,-2.4605000529,-1.27
78395054,2.445758614\C,2.1162833029,-1.7492773857,1.1845655862\N,2.552
1897624,-0.6462704252,0.4915242174\H,1.8150681911,0.0323588558,0.29063
89016\C,3.7639646443,-0.3364725177,-0.1402453273\C,6.093734659,0.52296
16848,-1.4867609118\C,3.7233616764,0.7478191292,-1.0364624299\C,4.9891
479649,-0.9795259467,0.0845617947\C,6.1294597367,-0.5470759909,-0.5969
76415\C,4.8730875859,1.1692702947,-1.6929239391\H,2.783548098,1.259013
0082,-1.2149660929\H,5.0438119582,-1.8039937348,0.7804315568\H,6.99013
1903,0.8544415669,-1.9952284063\C,-0.2968764985,1.9072342884,0.0846802
039\N,-0.0723374781,2.414501873,1.3528033619\C,-0.665947409,3.56430493
49,1.5208341476\S,-1.6071625878,4.2421042796,0.2032703454\C,-1.1787173
412,2.7697570773,-0.8429538725\O,0.1921714967,0.8504375664,-0.31221083
21\H,-3.766023552,-2.0692252244,-0.4188835204\C,-0.3385336638,3.209762
7799,-2.0538527404\H,-0.0301501418,2.3220086166,-2.614344406\H,0.56169
16913,3.7455182579,-1.7391864539\H,-0.9116737497,3.8593510735,-2.71757
71337\C,-0.5692516699,4.3269566182,2.7764733078\C,-0.4548982371,5.8334
89499,5.0426559208\C,0.1971889938,3.8568729727,3.8490768049\C,0.250598
5832,4.633008797,5.0052559532\C,-1.192340477,6.2155914889,3.9157547984
\H,0.7262778893,2.9152712514,3.7635201618\H,0.8339825416,4.3052851378,
5.8598517257\H,-1.7533408738,7.1471130061,3.9125417209\H,-0.4397318442
,6.467982629,5.9226513114\C,-2.4346944757,1.9173250388,-1.2518044291\C
,-3.195522128,1.4233617571,-0.0552513246\N,-3.5020150274,0.1460398228,
0.0967906293\O,-3.0605362294,-0.7519065978,-0.765998605\O,-4.228089615

```
7,-0.2650842757,1.067385237\H,-3.6338238521,2.0805003388,0.6817659453\
H,-2.0068941977,1.0295935665,-1.7254680632\C,-3.3213912092,2.610655227
,-2.2879886721\C,-3.2000183383,2.2773371607,-3.6446673695\C,-4.2785958
713,3.5701081057,-1.9233322471\C,-4.0005123455,2.8892831621,-4.6111636
158\H,-2.4754567573,1.5250855799,-3.9454638403\C,-5.0759390123,4.18738
11387,-2.8873606389\H,-4.4134962813,3.828642829,-0.8777982129\C,-4.939
4086953,3.8503789239,-4.2356069505\H,-3.8911189793,2.6121888962,-5.656
0569857\H,-5.8099272555,4.9280933623,-2.5825872024\H,-5.5638401476,4.3
283967306,-4.9848701237\C,7.4191688207,-1.2933746092,-0.3736089156\C,4
.7883669126,2.3001565758,-2.6817798232\F,7.5944506388,-1.6202962024,0.
9259541431\F,7.4549080967,-2.4515002724,-1.0739679\F,8.4974279412,-0.5
717644102,-0.7547228473\F,4.566664319,1.8545794894,-3.9417295039\F,3.7
810697628,3.156716693,-2.3905492852\F,5.9302467984,3.0215851943,-2.721
052126\S,3.013302031,-3.1515788115,1.481316873\N,-1.2516858735,5.48460
4181,2.8040230231\\Version=ES64L-G09RevD.01\State=1-A\HF=-3710.8605137
\RMSD=3.554e-09\RMSF=6.790e-06\Dipole=-4.375349,1.8393436,-0.121126\Qu
adrupole=-19.1380378,4.3575347,14.7805031,27.9974243,14.6659004,36.774
3227\PG=C01 [X(C42H51F6N7O4S2)]\\@
```


int1B-*S,S*


```
1\1\GINC-R2992\FOpt\RB3LYP\6-31G(d,p)\C42H51F6N7O4S2\ROOT\09-Dec-2015\
0\\# freq=noraman rb3lyp/6-31g** opt=maxcyc=200 scf=maxcyc=200 scrf=(s
md,solvent=toluene)\\Intm 1 after conj. addition conf. 1: S(elec),S(nu
c) (tol opt; cat III)\\0,1\C,2.1923914501,-3.4393816022,-0.7007314637\
O,3.1911352358,-4.1078546386,-0.9822222946\C,1.1835724327,-3.023437169
,-1.8015974682\H,0.6828295464,-3.9545452448,-2.0857808437\N,1.88138073
34,-3.0552387991,0.5619506813\H,1.0566839052,-2.4817241195,0.658966618
4\C,2.6317908187,-3.3480969325,1.7920520376\H,2.1912532466,-2.67183850
3,2.532643882\C,2.3945092012,-4.7900096504,2.376374905\C,0.8802177839
,-5.0741022258,2.3808767151\H,0.4673111065,-5.1147995901,1.3699639441\
H,0.3274428436,-4.3089263219,2.9397871252\H,0.679671417,-6.0380889634,
2.8602190075\C,3.117617842,-5.8970447122,1.586041763\H,2.7805473658,-5
.9471415079,0.5500807773\H,2.9280094991,-6.8678955801,2.0581404229\H,4
.2030240177,-5.7502948551,1.57067677\C,2.886981808,-4.8138664762,3.839
6134325\H,3.9695316825,-4.6720404358,3.9273464309\H,2.6595281215,-5.78
49906015,4.2913059717\H,2.3916999682,-4.0478569532,4.447988065\C,4.115
7740246,-2.9555352603,1.6277098394\H,4.7638125308,-3.5466159808,2.2770
728762\H,4.4266884352,-3.1222075047,0.5976193911\N,4.3625831515,-1.515
330075,1.9658517924\C,5.6906832331,-1.0166242005,1.4490256047\H,5.5558
773461,-0.7123593642,0.4100226288\H,6.4101361363,-1.8397618382,1.49908
11001\C,6.057649101,0.1111620739,2.4071669645\H,7.1218443306,0.3552714
105,2.3601202669\H,5.4765541033,1.0065665872,2.1717830235\C,4.32003541
56,-1.2103566002,3.4598161424\H,4.2316054235,-2.1489571976,4.007545347
\H,3.4432468432,-0.5851055934,3.6187257483\C,5.6207207062,-0.451325046
5,3.7681926779\H,6.3814981683,-1.1359311755,4.1592042699\H,5.456373361
2,0.3297949499,4.5138381858\N,0.1275617236,-2.1558285298,-1.2834588462
\H,0.347830495,-1.1651914554,-1.2131679947\C,1.8743622738,-2.440399531
8,-3.0937864306\C,2.506416072,-3.5962897394,-3.8962568235\H,3.00757247
73,-3.2003719681,-4.7872836883\H,1.7396073916,-4.3030342455,-4.2344325
965\H,3.2351036998,-4.1438333726,-3.2982503524\C,0.8087609516,-1.77642
99915,-3.9887576543\H,1.2649059986,-1.4868080177,-4.9417901705\H,-0.01
43578609,-2.4633185896,-4.2120973718\H,0.382111097,-0.8735866878,-3.54
23546951\C,2.9601316112,-1.4088593351,-2.7314828314\H,2.5962140539,-0.
648675071,-2.0323497539\H,3.3131208863,-0.9014782357,-3.6365736933\H,3
.822559249,-1.8930595474,-2.2655461988\C,-1.1853490657,-2.522648533,-1
.1440109045\N,-1.9812505063,-1.457845754,-0.8092505045\H,-1.4922451031
,-0.5599475471,-0.7623607257\C,-3.3504827403,-1.3418508291,-0.51871597
38\C,-6.0612379464,-0.8609168545,0.1227585026\C,-3.7686595905,-0.08290
72837,-0.0516070176\C,-4.3091866109,-2.3563964208,-0.673061295\C,-5.64
15355511,-2.1036284832,-0.3486665731\C,-5.1065726586,0.1444914283,0.26
30120522\H,-3.0435767713,0.7179333064,0.0674400597\H,-4.006103816,-3.3
286771819,-1.0314748635\H,-7.0982762143,-0.6822924408,0.3753758323\C,-
0.2485589148,2.0509595122,-0.7662963465\N,-1.16461693,2.6324626292,0.0
930647203\C,-0.9328150418,3.9069092642,0.2521587899\S,0.414517865,4.64
26184958,-0.5995129863\C,0.8229207225,2.9967463229,-1.3509346781\O,-0.
2865877616,0.8657061084,-1.0873357531\H,3.6284366075,-0.8813172099,1.4
637852621\C,0.652017749,3.0617909141,-2.8772975587\H,0.8138727174,2.06
63614384,-3.3011504216\H,1.3690227544,3.7515019384,-3.3259531595\H,-0.
35647715,3.3886830978,-3.1463069045\C,-1.7564603668,4.7627323567,1.122
2154502\C,-3.208110684,6.4468453028,2.696163658\C,-2.8200981946,4.2319
947222,1.8594624665\C,-3.5589608257,5.0996259368,2.6613630236\C,-2.123
5212594,6.8788950664,1.9230527417\H,-3.0496898508,3.1771390755,1.80270
88101\H,-4.3941818036,4.7241378282,3.2439800267\H,-1.8227617918,7.9239
267192,1.9287135859\H,-3.756557087,7.1564707134,3.3068994462\C,2.23252
55448,2.4579806095,-0.9080893565\C,2.3394248545,2.3498621964,0.5837982
298\N,2.66903028,1.2283060432,1.2034658273\O,2.8188612758,0.0982077102
,0.5356749832\O,2.8535132411,1.2090125325,2.4709789368\H,2.2649812111,
```

3.2079833531,1.2354736995\H,2.2559604287,1.4371899494,-1.2963074062\C,
3.3960200571,3.2212912576,-1.545082783\C,4.0327345097,2.6981008655,-2.
6796698957\C,3.8680781785,4.4347042461,-1.0209002964\C,5.1020818348,3.
3657336153,-3.2792428309\H,3.6920630051,1.7527849324,-3.0930011593\C,4
.9336885449,5.1067522761,-1.6207540533\H,3.4116197961,4.8543069843,-0.
1299727015\C,5.5548633083,4.5756851969,-2.7529642201\H,5.5815887735,2.
9368764079,-4.1548946388\H,5.282929766,6.0448164989,-1.1982910835\H,6.
3872889379,5.0973356231,-3.2164758475\C,-6.6674304492,-3.1830328916,-0
.5772059528\C,-5.498811725,1.4947020827,0.7961809929\F,-6.1437654201,-
4.4210568641,-0.4618082642\F,-7.2126296987,-3.1009086483,-1.8152805171
\F,-7.6926270214,-3.0942095751,0.3022466742\F,-4.9845077939,1.71263515
94,2.0384130481\F,-5.03752411,2.501043054,0.0178432724\F,-6.8349005537
,1.6449125836,0.8976857811\S,-1.7010700684,-4.1240718829,-1.3417897106
\N,-1.4097443433,6.061652061,1.1510847663\\Version=ES64L-G09RevD.01\St
ate=1-A\HF=-3710.8613339\RMSD=6.691e-09\RMSF=4.610e-06\Dipole=3.050112
8,2.5611217,2.221739\Quadrupole=4.6198761,-9.6817639,5.0618879,-30.094
7796,0.0765294,-9.9372846\PG=C01 [X(C42H51F6N7O4S2)]\\@

TS2A-*R,R*

1\1\GINC-R3203\FTS\RB3LYP\6-31G(d,p)\C42H51F6N7O4S2\ROOT\09-Dec-2015\0
\\# freq=noraman rb3lyp/6-31g** opt=(ts,calcfc,noeigen,maxcyc=200) # s
cf=maxcyc=200 scrf=(smd,solvent=toluene) iop(1/8=1)\\TS for 2nd C-C bo
nd addition forms DA pdt conf. 1: R(elec),R(nuc) (tol opt; cat III)\\0
,1\C,-0.9463022725,-3.5165523423,0.8638745443\O,-1.354655335,-4.678846
5091,0.9386132137\C,-0.1030651699,-2.9301930948,2.0215598382\H,0.64285
96761,-3.7054159405,2.2146983163\N,-1.2012512582,-2.7074720296,-0.2007
621722\H,-0.9112372324,-1.7391911918,-0.1418203459\C,-1.9113088072,-3.
0845146106,-1.4343816156\H,-2.0897810322,-2.1235231155,-1.9283507357\C
,-1.068219895,-3.9206301746,-2.4689040447\C,0.3095373313,-3.2547911335
,-2.6388578155\H,0.894257276,-3.2861644401,-1.7165570786\H,0.214364333
8,-2.2088198784,-2.9543339257\H,0.8836544735,-3.7779497992,-3.41087852
46\C,-0.8700777428,-5.3920636576,-2.0585338142\H,-0.3168848426,-5.4844
410681,-1.1233637117\H,-0.3107974468,-5.9166650381,-2.8415925379\H,-1.
8222416121,-5.9190232785,-1.9321247645\C,-1.789711254,-3.8719301096,-3
.8344976558\H,-2.7491765449,-4.4007110656,-3.8318199704\H,-1.170150562
7,-4.3556623148,-4.5965984477\H,-1.9672256288,-2.8416294598,-4.1651241
87\C,-3.2801217872,-3.7112839326,-1.0993643144\H,-3.6468317351,-4.3527
112141,-1.9012446754\H,-3.1935470477,-4.3083815852,-0.1930820943\N,-4.
3413254893,-2.6706416634,-0.8688055376\C,-5.5299672819,-3.1912359318,-
0.0971893219\H,-5.2560526643,-3.2696812908,0.9559973682\H,-5.766598472
8,-4.1868466641,-0.4841941653\C,-6.6263301226,-2.1814423916,-0.4097136
626\H,-7.6192410942,-2.574474646,-0.1805530455\H,-6.4671121148,-1.2729
495115,0.1769927873\C,-4.8999698529,-2.027909282,-2.1348356021\H,-4.64
73436516,-2.6655654053,-2.9818880068\H,-4.4044141663,-1.065498863,-2.2
590681486\C,-6.4210396909,-1.9052703969,-1.9108236915\H,-6.9469762611,
-2.6552119328,-2.5090322197\H,-6.7914690087,-0.9229371292,-2.210741659
3\N,0.6443650938,-1.7356256326,1.6464757225\H,0.1913575464,-0.82697685
83,1.7741385667\C,-0.936980717,-2.7311508837,3.346631387\C,-1.27273221
87,-4.1141627063,3.9407172151\H,-1.8498331128,-3.989928608,4.864256143
5\H,-0.3605346222,-4.6681603639,4.1892263548\H,-1.851167246,-4.7251547
305,3.2461322623\C,-0.0826180905,-1.9657159451,4.3772845275\H,-0.61193
72253,-1.9323090557,5.3359180881\H,0.8798356587,-2.4600656606,4.547087
8054\H,0.1156585025,-0.9328408151,4.0780211449\C,-2.2450490447,-1.9600
11647,3.0881098336\H,-2.0789259612,-0.9888138183,2.6157095773\H,-2.767
5582245,-1.7825693187,4.0350210406\H,-2.9227149404,-2.5332919565,2.447
0065165\C,1.9558159802,-1.7352734575,1.2837525787\S,2.8466097545,-3.15
77800035,1.0272132255\N,2.459123432,-0.4698924276,1.1129343287\H,1.791
1340978,0.3109393928,1.119942462\C,3.7450422126,-0.0909497499,0.677549
4282\C,6.2520089252,0.8136021855,-0.2298220513\C,3.8327408562,0.975660
6121,-0.2244972908\C,4.9235810229,-0.6825052178,1.1566603129\C,6.15773
58744,-0.2293666387,0.6951422177\C,5.0784676437,1.4142511597,-0.676475
3066\H,2.9267782225,1.4584352927,-0.5718888932\H,4.8713852867,-1.48778
19874,1.8747115404\H,7.2170970201,1.1500848136,-0.5883654387\C,7.42768
0206,-0.8142136376,1.2527811251\C,5.1271708821,2.49130159,-1.724360646
5\F,4.2113634613,3.4594200653,-1.5029484529\F,6.3369601198,3.085964301
4,-1.7966996453\F,4.8637319567,1.9935197835,-2.9641817336\F,7.23547534
31,-2.020801186,1.8230794593\F,8.3722646523,-0.964875558,0.2943556073\
F,7.9706458715,-0.0110366986,2.2015857931\C,-3.2901728992,0.6189450253
,-0.1604863817\N,-2.1437383378,0.4696812491,-0.8714638232\C,-1.7654261
795,1.6798508457,-1.3463996311\S,-3.1504709226,2.8275908429,-1.5883037
031\C,-3.6960051784,2.0948769833,0.0204972274\O,-3.9502204991,-0.30797
91264,0.3531252444\H,-3.9546327503,-1.8636458301,-0.3041234016\C,-5.16
07551571,2.2894003148,0.3606156438\H,-5.383377079,1.8140429155,1.32039
35992\H,-5.7964843165,1.822259988,-0.3965888414\H,-5.4231150987,3.3467
399944,0.4312816634\C,-0.5753286043,1.8404348397,-2.2100760394\C,1.613
4135953,2.2798587252,-3.7917796475\C,0.419615812,0.8566911144,-2.26969
16993\C,1.5313971928,1.0855450817,-3.0795687935\C,0.5620479644,3.19391

02165,-3.6764459413\H,0.3196964308,-0.0541323718,-1.6937050106\H,2.325
987605,0.3488495534,-3.1413440535\H,0.5855719119,4.1329871891,-4.22518
30752\H,2.4726082023,2.5054075945,-4.413611175\C,-2.6655990769,2.70654
82123,1.0992177877\C,-1.2727101321,2.6818791028,0.5156421694\N,-0.3578
970909,1.7958007058,1.0475465426\O,-0.7681602115,0.7970128299,1.705876
5726\O,0.8690045622,1.937096865,0.7777051567\H,-0.7952799001,3.5882373
041,0.1703379412\H,-2.6885155186,2.0151717358,1.9454928568\C,-3.082207
0062,4.0772331054,1.6175468235\C,-3.6994175447,4.1634858395,2.87460448
31\C,-2.8792938773,5.2636412449,0.8956218446\C,-4.1130376509,5.3910810
493,3.3927063446\H,-3.8531453662,3.2578177793,3.4556345413\C,-3.291519
0127,6.492566081,1.4130838351\H,-2.4018142077,5.2324890721,-0.07774586
02\C,-3.9108676553,6.5622752278,2.661913689\H,-4.586491818,5.430970939
4,4.3696529743\H,-3.1222627373,7.3984424789,0.8378102638\H,-4.22777371
62,7.5204729442,3.0633863003\N,-0.5074039631,2.9882828237,-2.906472808
5\\Version=ES64L-G09RevD.01\State=1-A\HF=-3710.8435663\RMSD=3.960e-09\
RMSF=1.886e-06\Dipole=-6.7126887,-0.3538909,-2.0756578\Quadrupole=9.30
10975,-12.9703199,3.6692224,30.6508612,-2.1779055,16.4546341\PG=C01 [X
(C42H51F6N7O4S2)]\\@

TS2A-*S,S*

1\1\GINC-R3090\FTS\RB3LYP\6-31G(d,p)\C42H51F6N7O4S2\ROOT\09-Dec-2015\0
\\# freq=noraman rb3lyp/6-31g** opt=(ts,calcfc,noeigen,maxcyc=200) # s
cf=maxcyc=200 scrf=(smd,solvent=toluene) iop(1/8=1)\\TS for 2nd C-C bo
nd addition forms DA pdt conf. 2: S(elec),S(nuc) (tol opt; cat III)\\0
,1\C,-1.6842813058,-3.5597508977,-0.3209492431\O,-2.5003078676,-4.4576
084508,-0.5528960983\C,-0.5593642772,-3.7779709313,0.7334822151\H,0.02
81910236,-4.6056764881,0.3240005654\N,-1.6733646296,-2.3672239707,-0.9
65562859\H,-0.9688943174,-1.6938766568,-0.6870686911\C,-2.4359650665,-
1.9605845084,-2.1574331101\H,-2.3475050644,-0.8696701893,-2.1631895734
\C,-1.7907753338,-2.4368841194,-3.515924424\C,-0.2733689335,-2.1739568
687,-3.4619952695\H,0.2290206844,-2.8163707076,-2.7350397442\H,-0.0522
667554,-1.1319739441,-3.2027876357\H,0.1751453778,-2.3743088133,-4.440
4438314\C,-2.0383204199,-3.9308809525,-3.7971168226\H,-1.6485847565,-4
.5663888567,-2.9990240463\H,-1.5439850357,-4.2204922367,-4.7311760991\
H,-3.1040293672,-4.157802875,-3.9113177258\C,-2.3751236892,-1.59103286
26,-4.6687406462\H,-3.4384076376,-1.7826719369,-4.8485857092\H,-1.8525
582133,-1.8248869056,-5.6021337108\H,-2.2547198635,-0.5175204204,-4.48
25440061\C,-3.9355714025,-2.2896182966,-2.0741516751\H,-4.3815387596,-
2.1466967651,-3.0582405564\H,-4.1033118695,-3.3157540278,-1.7491690718
\N,-4.6991842473,-1.3790345861,-1.1508913611\C,-4.5536914273,-1.672426
0021,0.326560889\H,-3.6609568753,-1.1565437377,0.6728843242\H,-4.43014
73868,-2.7510723123,0.4294334478\C,-5.8516975273,-1.1545822088,0.98366
49962\H,-6.3282313035,-1.9578171199,1.5512138454\H,-5.6391164446,-0.33
57844838,1.6734712547\C,-6.1815812475,-1.3852069643,-1.4087874606\H,-6
.5011464634,-2.4295853648,-1.4745935896\H,-6.3790568547,-0.8847632702,
-2.3586104953\C,-6.7543359901,-0.6820579359,-0.1823258496\H,-7.8074443
463,-0.9302011942,-0.0332012143\H,-6.6795927535,0.4014000984,-0.310449
0008\N,0.3595553889,-2.647629568,0.8093946777\H,0.026782923,-1.8063750
982,1.2702410963\C,-1.0501209779,-4.2207641073,2.1622363321\C,-1.78358
75101,-5.57470078,2.0920507966\H,-2.0469659549,-5.8983030812,3.1056636
124\H,-1.1417001432,-6.3470559872,1.6541177852\H,-2.6919691803,-5.5234
942865,1.4923702215\C,0.1841948695,-4.4014179003,3.0699135159\H,-0.129
5900406,-4.789632199,4.0451238199\H,0.8960688176,-5.1133761666,2.64069
91324\H,0.7146815673,-3.4612104574,3.2447774204\C,-1.9775472622,-3.162
6664843,2.7860130976\H,-1.4866956421,-2.1858474738,2.8709620648\H,-2.2
657689566,-3.4626256063,3.7996489105\H,-2.8982264583,-3.0388553665,2.2
08107421\C,1.6661363911,-2.6464241568,0.4020567105\S,2.4020434616,-3.9
985617229,-0.3000950456\N,2.253269392,-1.427161386,0.6180558507\H,1.60
30892676,-0.6891162001,0.9113445637\C,3.5592886322,-0.9495743145,0.431
1319858\C,6.0996370115,0.2521684802,0.1372065151\C,3.728083939,0.43706
06083,0.6066243396\C,4.6820716091,-1.7314862118,0.1237869853\C,5.92923
39666,-1.120300801,-0.023122148\C,4.9807285359,1.0216852928,0.46012007
77\H,2.8710214568,1.0523846315,0.8607110974\H,4.5768656171,-2.79920447
93,-0.0006876498\H,7.0726009744,0.7105574952,0.0165713015\C,7.10310496
21,-1.9759835609,-0.4221082008\C,5.137159785,2.4957916963,0.7200713452
\F,5.3517741904,2.7539073775,2.0334058267\F,6.1844353615,3.0201595982,
0.0454115822\F,4.0359997778,3.1942024284,0.3641423528\F,7.1183834463,-
2.2157917237,-1.7550917423\F,8.2843818731,-1.3919046196,-0.1171137097\
F,7.081853543,-3.181586578,0.1879398611\C,-3.3459265277,1.8017076395,-
0.6968436792\N,-3.1513496489,1.3397841352,0.5585642691\C,-2.552071396,
2.3075744125,1.2945919688\S,-2.9523451006,3.9876491297,0.729084142\C,-
2.7214318071,3.1924389418,-0.924911324\O,-3.8906502631,1.1670684305,-1
.6281895335\H,-4.364000415,-0.3779045047,-1.3076421824\C,-3.3004473444
,3.9446137373,-2.1068775454\H,-3.1206180066,3.3793315674,-3.0260763046
\H,-2.8530841459,4.9343651673,-2.2178318799\H,-4.3821477712,4.05810598
,-1.9960703459\C,-2.2806549949,2.1159871208,2.7387362701\C,-1.76167049
08,1.8759586341,5.4127035042\C,-2.3156203656,0.8423816841,3.3186483285

```
\C,-2.0520892315,0.7263569658,4.681891167\C,-1.7509841831,3.1025179138
,4.7428867858\H,-2.5570627405,-0.0190854195,2.7094914565\H,-2.07408375
54,-0.2460931317,5.1644480317\H,-1.5332275816,4.0223702049,5.281554910
5\H,-1.5491741473,1.8314981551,6.4758056619\C,-1.1319703739,2.92838639
67,-0.9995145314\C,-0.6623965507,2.3978401843,0.3398348287\N,-0.248478
3422,1.0775929985,0.3945693605\O,-0.6062598413,0.2713871413,-0.5000918
6\O,0.3973820479,0.6731160173,1.4157202403\H,-0.0890111965,3.020712905
9,1.0126759834\H,-1.0038824064,2.1332115915,-1.7382059027\C,-0.3351031
739,4.1314159037,-1.4864159433\C,0.126124475,4.1404441443,-2.811265342
3\C,-0.0350247538,5.234143622,-0.671392079\C,0.8567333687,5.2174182057
,-3.3137693977\H,-0.0841729393,3.2897093302,-3.4545652603\C,0.69580804
14,6.3121497723,-1.1728389874\H,-0.3753810966,5.2560506923,0.358147869
4\C,1.1435824383,6.3098701873,-2.4947890478\H,1.2054103679,5.198271560
6,-4.3424225828\H,0.9192728158,7.1544734443,-0.524208666\H,1.716257060
3,7.1482700201,-2.8805733148\N,-2.0006253105,3.2279555903,3.4385121114
\\Version=ES64L-G09RevD.01\State=1-A\HF=-3710.8438388\RMSD=4.158e-09\R
MSF=1.960e-06\Dipole=-5.7341306,0.5692837,0.3092312\Quadrupole=3.95605
43,-26.4885085,22.5324542,26.8575029,4.9977499,-6.3001326\PG=C01 [X(C4
2H51F6N7O4S2)]\\@
```

int2A-*R,R*

```
1\1\GINC-R3470\FOpt\RB3LYP\6-31G(d,p)\C42H51F6N7O4S2\ROOT\09-Dec-2015\
0\\# freq=noraman rb3lyp/6-31g** opt=maxcyc=200 scf=maxcyc=200 scrf=(s
md,solvent=toluene)\\Diels-Alder product (non protonated) conf. 1: R(e
lec),R(nuc) (tol opt; cat IV)\\0,1\C,-2.0722612703,-2.6687257708,0.830
8610355\O,-2.7343918474,-3.7115192172,0.8399954461\C,-1.1366628525,-2.
3800735104,2.0308335388\H,-0.6815223631,-3.3528843829,2.227764882\N,-2
.1099706586,-1.7690005046,-0.1861897398\H,-1.6319977097,-0.8710363404,
-0.0999868022\C,-2.896623028,-1.9007415533,-1.4233788656\H,-2.82197917
99,-0.9030533635,-1.8691667971\C,-2.3055849945,-2.8753507923,-2.510829
5434\C,-0.7816507208,-2.6744539581,-2.5913523844\H,-0.2772324222,-3.04
17850459,-1.6951201464\H,-0.5145454826,-1.6195291067,-2.7184684091\H,-
0.3779466381,-3.2262075064,-3.4471753214\C,-2.6011034406,-4.3633129924
,-2.2378277361\H,-2.2026499551,-4.6872093145,-1.2759776056\H,-2.147946
6596,-4.9762118709,-3.0253354778\H,-3.6753302168,-4.5804984487,-2.2414
06154\C,-2.9159152758,-2.4861665935,-3.8751878466\H,-4.0050410595,-2.6
027255419,-3.9031302683\H,-2.50908119,-3.1274379044,-4.6639596802\H,-2
.6824000443,-1.4487623661,-4.1428658491\C,-4.3774046179,-2.1749028292,
-1.1009089369\H,-4.9086687656,-2.6087300507,-1.9496027234\H,-4.4350811
652,-2.869454517,-0.2649327499\N,-5.1237336109,-0.9261730476,-0.718777
6312\C,-6.3415920969,-1.194982463,0.1502026118\H,-6.028882819,-1.08847
06437,1.1898490614\H,-6.6543828659,-2.2268393343,-0.0157338665\C,-7.41
59018191,-0.1772813891,-0.285090817\H,-8.2295792801,-0.6943544202,-0.8
018894184\H,-7.8470964113,0.3435434545,0.5721898421\C,-5.616303915,-0
.0951562505,-1.8779802631\H,-6.0346835822,-0.7759073537,-2.6253071581\
H,-4.7769130511,0.450366029,-2.3099978257\C,-6.6889073075,0.7843119141
,-1.2449462222\H,-7.3515656837,1.2213281869,-1.9952504478\H,-6.2093529
72,1.5909509865,-0.6860086615\N,-0.0413192798,-1.4797453272,1.69861249
77\H,-0.2624687136,-0.4917940309,1.6255385605\C,-1.8730222071,-1.93246
79026,3.3492835037\C,-2.9899248743,-2.9331537589,3.7025266556\H,-3.414
1078053,-2.6712537265,4.6784961981\H,-2.6103759303,-3.9575659638,3.764
0998547\H,-3.7989484491,-2.929370999,2.9689998091\C,-0.8411564482,-1.9
343585156,4.4951632217\H,-1.327370178,-1.656409715,5.4367694132\H,-0.3
919730421,-2.9242056553,4.6296518653\H,-0.0323126901,-1.2195901383,4.3
173617275\C,-2.4766443427,-0.5217486574,3.2176153009\H,-1.7037729617,0
.2466407909,3.1108544479\H,-3.0431135664,-0.2746352955,4.122777671 3\H,
-3.1540081213,-0.4282897668,2.3642128113\C,1.2431759454,-1.856595592,1
.4469512973\S,1.7420551173,-3.4729216614,1.4127268316\N,2.0642359426,-
0.7674745027,1.2691015107\H,1.6373336506,0.1246050385,1.5110640964\C,3
.3957609401,-0.6370823802,0.8507817683\C,6.0343166709,-0.1142871538,-0
.0085677001\C,3.9716873648,0.6347091162,1.0259964907\C,4.1526630109,-1
.6438382714,0.2348071522\C,5.4578287816,-1.3707747563,-0.1780121557\C,
5.2697648084,0.8859029965,0.5940324571\H,3.397163153,1.4298183505,1.49
18420539\H,3.7245088697,-2.6238028286,0.084377426\H,7.0469414858,0.082
8058686,-0.3372120626\C,6.271130349,-2.486539438,-0.7797831557\C,5.824
2269196,2.2797337144,0.7000571522\F,5.3060746274,2.9617746592,1.741463
8957\F,7.1682005211,2.2824688347,0.8391602162\F,5.5525139761,3.0084821
185,-0.4170508029\F,6.8669353217,-3.2444732119,0.1706139205\F,5.513341
2197,-3.3215863274,-1.5244819594\F,7.2575110127,-2.0191271638,-1.57993
5532\C,-2.9153123028,1.7503861699,0.0758185572\N,-1.7649226315,1.28251
86531,-0.3873749331\C,-0.9355072714,2.404830527,-0.7610253902\S,-2.152
5544133,3.7060140082,-1.4953914984\C,-2.9454933622,3.3032351108,0.1412
524855\O,-3.907535053,1.0818637518,0.490869138\H,-4.4836429595,-0.2640
876928,-0.1708007005\C,-4.304773752,3.9197438186,0.3862156298\H,-4.717
457616,3.5412051404,1.3262535011\H,-4.9987733427,3.6505846972,-0.41453
96606\H,-4.249334411,5.0089953027,0.4443762698\C,0.1854242244,2.063548
2354,-1.7153296667\C,2.2777140058,1.5166711873,-3.3949680068\C,-0.0780
```

636335,1.4271877009,-2.9344374787\C,0.9879120906,1.1522048822,-3.78566
88445\C,2.4418015793,2.1391063291,-2.1602664402\H,-1.0966418441,1.1718
339152,-3.202469759\H,0.8166165149,0.66428175,-4.7406544437\H,3.427935
2511,2.432239575,-1.8092942881\H,3.1365514476,1.3206929917,-4.02835524
47\C,-1.8224510993,3.6461136783,1.2103874301\C,-0.505533888,3.28936252
25,0.463548944\N,0.4881101759,2.6449886958,1.3982411306\O,0.2585803071
,1.5030940563,1.828155674\O,1.4733628173,3.293057848,1.7154167868\H,0.
0299755557,4.1699739408,0.1246680756\H,-1.9590407868,2.9397279889,2.03
5774778\C,-1.8541845478,5.0435152147,1.801141969\C,-2.2546985607,5.208
1113374,3.1347411727\C,-1.5170368435,6.1874366411,1.0604122784\C,-2.31
77418982,6.4749358449,3.7163832992\H,-2.5129970947,4.3329767361,3.7258
952215\C,-1.5821015483,7.4546467805,1.6396729394\H,-1.2086777348,6.088
8085226,0.0245815748\C,-1.9818874773,7.6039156002,2.9691181626\H,-2.62
388245,6.5772082401,4.7536004833\H,-1.3157596298,8.3273706847,1.050208
2336\H,-2.0257208576,8.5912935055,3.4195380598\N,1.4209755719,2.408325
8928,-1.3335413376\\Version=ES64L-G09RevD.01\State=1-A\HF=-3710.853913
7\RMSD=3.316e-09\RMSF=6.677e-06\Dipole=-6.5453333,1.5025983,-2.1238696
\Quadrupole=23.6854392,-32.6630229,8.9775837,15.2527424,3.5125071,12.6
167509\PG=C01 [X(C42H51F6N7O4S2)]\\@

int2A-*S,S*

1\1\GINC-R3505\FOpt\RB3LYP\6-31G(d,p)\C42H51F6N7O4S2\ROOT\09-Dec-2015\
0\\# freq=noraman rb3lyp/6-31g** opt=maxcyc=200 scf=maxcyc=200 scrf=(s
md,solvent=toluene)\\Diels-Alder product (non protonated) conf. 2: S(e
lec),S(nuc) (tol opt; cat IV)\\0,1\C,-1.4189449435,-3.1930664887,0.238
3783568\O,-2.1800947861,-4.1640017228,0.2879621596\C,-0.192671519,-3.1
149902939,1.193622236\H,0.4240710081,-3.9708664156,0.901475962\N,-1.55
75039246,-2.1687148122,-0.6410257334\H,-0.8504389285,-1.4464897568,-0.
5973461954\C,-2.4384491503,-2.0843466509,-1.8205517089\H,-2.4718007544
,-1.015186766,-2.0534932831\C,-1.8352343178,-2.780064706,-3.101725303\
C,-0.3610191687,-2.3568522452,-3.2547773794\H,0.2692805988,-2.76187619
45,-2.4593955889\H,-0.2494917778,-1.2660229824,-3.2587104182\H,0.03668
64191,-2.7293300351,-4.2044627525\C,-1.9123201226,-4.3166766242,-3.029
2363549\H,-1.3951558695,-4.7110505561,-2.1515176584\H,-1.4459674613,-4
.7543559553,-3.9189308585\H,-2.9460460964,-4.67792702,-2.9958456398\C,
-2.5966346262,-2.2833427962,-4.3504011726\H,-3.6365747899,-2.623689823
8,-4.385480285\H,-2.111268711,-2.6649859714,-5.2546771969\H,-2.5984496
425,-1.1889947134,-4.4135580981\C,-3.8872592431,-2.5221676388,-1.54472
8255\H,-4.4076618911,-2.6000217007,-2.4989125821\H,-3.9244973327,-3.48
45615327,-1.037135668\N,-4.6773302173,-1.5334200928,-0.7368786224\C,-4
.4466581071,-1.5692852748,0.7627009995\H,-3.7046953943,-0.8008965845,0
.982062169\H,-4.0570322677,-2.5543606787,1.0185911815\C,-5.82221494,-1
.2879926524,1.4071384573\H,-6.1968176693,-2.1901345689,1.9000597695\H,
-5.7606825507,-0.5002189713,2.1610865289\C,-6.1609715752,-1.6742112473
,-0.9366471541\H,-6.4143739918,-2.7383785788,-0.8878409331\H,-6.421049
991,-1.2838700648,-1.9225026861\C,-6.7435851189,-0.8954103448,0.235700
741\H,-7.7921678089,-1.14530589,0.4131232882\H,-6.6776351376,0.1777470
881,0.0328489476\N,0.6346125288,-1.9361454859,0.9461051021\H,0.3371887
628,-1.0781270362,1.3970888531\C,-0.5191070827,-3.277410352,2.72653331
38\C,-1.0938226601,-4.6810320979,3.0037886981\H,-1.2555422518,-4.80402
33695,4.0809106568\H,-0.3957668747,-5.4618825324,2.6820675006\H,-2.036
8359501,-4.8465648431,2.483816294\C,0.7935417034,-3.1406421954,3.52634
17565\H,0.6011950031,-3.3505200532,4.5837876205\H,1.5512637573,-3.8499
777413,3.178517122\H,1.2213309112,-2.1354777321,3.4665230226\C,-1.5171
307761,-2.2099819073,3.2114850928\H,-1.1626231018,-1.1871823488,3.0344
394803\H,-1.674209239,-2.306337841,4.2914020853\H,-2.491478325,-2.3183
306642,2.7277110894\C,1.9028796282,-1.9500183786,0.430739314\S,2.64015
23037,-3.3490253314,-0.1593932182\N,2.4589329074,-0.6947882886,0.42048
58991\H,1.8034286851,0.0728792549,0.5701375552\C,3.7488744756,-0.26651
16092,0.0585634433\C,6.2716953414,0.7908101182,-0.6158598474\C,3.87418
54783,1.0595911605,-0.3838218787\C,4.9006951042,-1.0554866612,0.182694
587\C,6.1413730606,-0.5217434537,-0.1651428463\C,5.1241430447,1.576977
2328,-0.7125946609\H,2.9932631202,1.6856711848,-0.4715455969\H,4.82803
52708,-2.0710538778,0.5413663275\H,7.2418999941,1.1928566158,-0.881000
3919\C,7.364445687,-1.3983257981,-0.0986334771\C,5.2538382537,3.020557
3907,-1.118398483\F,5.5811779519,3.8075685533,-0.0651344208\F,6.220412
9392,3.1953556863,-2.0470957798\F,4.105389526,3.5127367437,-1.63190414
06\F,7.6113927207,-2.0029564505,-1.2844165843\F,8.4737903151,-0.688617
3188,0.2124991636\F,7.2412521224,-2.3772864117,0.8231408047\C,-3.61500
68501,1.7151861003,-0.9068289125\N,-3.1157126814,1.4929721885,0.296755
5134\C,-2.4195321457,2.6713235902,0.7134423731\S,-3.4463795068,4.15163
17328,0.0962250563\C,-3.2835564947,3.1352729212,-1.4482526784\O,-4.227
3517745,0.8844996353,-1.6458624938\H,-4.4322586502,-0.5245945652,-1.07
80719736\C,-4.1085392525,3.5844645296,-2.6343244597\H,-3.9815542251,2.
8783627022,-3.4606703859\H,-3.8171596927,4.579469025,-2.9783627898\H,-
5.1712130165,3.5997696485,-2.3771362616\C,-2.1190968729,2.7798991342,2
.1894840902\C,-1.5617399086,3.1168253947,4.848594727\C,-2.6998838245,1

.9074968906,3.1134648248\C,-2.4144896276,2.0833489251,4.4670175375\C,-
1.0326079612,3.9367018899,3.8501575736\H,-3.3608944051,1.1261351063,2.
7614148704\H,-2.853247378,1.4240369702,5.2104049086\H,-0.3639387343,4.
7559538439,4.1069948025\H,-1.3103610199,3.2896151279,5.8899939439\C,-1
.7120377398,3.0777048358,-1.6635361315\C,-1.1531234466,2.8744161961,-0
.2340962194\N,-0.2820402618,1.6507001104,-0.1207327173\O,-0.4214932058
,0.7149184486,-0.9045074149\O,0.5198205619,1.6227242994,0.8270123518\H
,-0.5312670757,3.6806721179,0.146408348\H,-1.5137482515,2.1741020851,-
2.245551352\C,-1.1013024142,4.241494242,-2.4256902868\C,-0.7827397899,
4.0621294428,-3.7805199896\C,-0.8468863789,5.4938768587,-1.8447956729\
C,-0.2361171935,5.0982607069,-4.5377955872\H,-0.9608591934,3.095869617
3,-4.2457980922\C,-0.2985808664,6.5310607985,-2.6008413379\H,-1.085327
9012,5.6661912422,-0.8009640873\C,0.00813803,6.3392733749,-3.948712316
4\H,0.0050050396,4.9321271056,-5.5838881381\H,-0.1079474323,7.49156629
89,-2.1306574112\H,0.4385052819,7.1476276674,-4.5327530497\N,-1.299699
025,3.780126533,2.5508425336\\Version=ES64L-G09RevD.01\State=1-A\HF=-3
710.8574218\RMSD=6.207e-09\RMSF=3.334e-06\Dipole=-4.5193444,-0.2536463
,0.6928996\Quadrupole=-2.1661381,-21.3549103,23.5210484,30.1793774,1.1
771194,0.4394585\PG=C01 [X(C42H51F6N7O4S2)]\\@

TS2’A-***R,R***

1\1\GINC-R2954\FTS\RB3LYP\6-31G(d,p)\C42H51F6N7O4S2\ROOT\07-Mar-2016\0
\\# freq=noraman b3lyp/6-31G(d,p) opt=(ts,calcfc,noeigen,maxcyc=200) s
cf=maxcyc=200 scrf=(smd,solvent=toluene)\\TS for protonation of int1DA
 conf. 1: R(elec),R(nuc) (tol opt; cat III)\\0,1\C,-2.5947187433,-3.64
38747332,0.4112902557\O,-2.4787015037,-4.869440531,0.4636541744\C,-3.8
752612725,-2.9554720028,-0.1560244372\H,-4.5527111448,-2.8265709862,0.
6964598518\N,-1.6399374811,-2.7846082148,0.8570367267\H,-1.8490829565,
-1.797703187,0.8054564987\C,-0.3733175139,-3.0946915189,1.5408528835\H
,0.1437096694,-2.1307257605,1.5455918013\C,-0.5333714327,-3.4808701537
,3.0637095568\C,-1.6087641977,-2.5749288719,3.694731771\H,-2.606537514
5,-2.7817442831,3.2992314819\H,-1.3955048354,-1.5130851939,3.524676207
5\H,-1.6425987357,-2.735054083,4.7775541113\C,-0.9196799917,-4.9568528
842,3.2826180773\H,-1.8372395673,-5.2205150119,2.7536996863\H,-1.07045
40374,-5.1443811724,4.351819013\H,-0.1333151557,-5.6409670183,2.945048
3051\C,0.8009456969,-3.1981743482,3.787962705\H,1.6307666577,-3.802664
8338,3.4071203928\H,0.7026791869,-3.4294285172,4.8537943354\H,1.084291
6427,-2.1423267071,3.7084545363\C,0.5084774123,-4.0840653946,0.7609676
4\H,1.4060746931,-4.2656670014,1.3538789926\H,-0.0113636542,-5.0352839
102,0.6253313825\N,0.9863909513,-3.626038388,-0.5840906582\C,0.0307675
693,-3.8785823964,-1.7151985277\H,-0.7469917749,-3.119933097,-1.710021
5685\H,-0.4277979369,-4.8601251055,-1.5602787747\C,0.8966161078,-3.851
3355423,-2.9838929365\H,0.5294900657,-4.5792124145,-3.7112363026\H,0.8
534848204,-2.8687236609,-3.4596326176\C,2.2108871544,-4.3883292542,-0.
9894929541\H,2.0413131603,-5.4469300079,-0.7561809852\H,3.0671089499,-
4.0363054051,-0.4130660199\C,2.337582177,-4.1708425363,-2.5015369852\H
,2.7545170723,-5.0572178801,-2.9854577406\H,3.0108931753,-3.3376140649
,-2.7138961749\N,-3.5546690054,-1.6038741924,-0.6009100677\H,-2.819917
8755,-1.5160661802,-1.2959411063\C,-4.6494794407,-3.7910956587,-1.2398
526169\C,-5.3140032393,-5.0105113146,-0.5683878438\H,-5.8825222983,-5.
5800157658,-1.3123788245\H,-6.0192685818,-4.691853366,0.2083585296\H,-
4.5762157639,-5.6714510689,-0.1135844174\C,-5.765032847,-2.9131883123,
-1.8418505918\H,-6.3848739482,-3.5192243967,-2.5118239422\H,-6.4144444
031,-2.4955758169,-1.0667809291\H,-5.3632654717,-2.0803218968,-2.42666
81491\C,-3.7206693968,-4.2594491823,-2.3755860856\H,-3.2154351442,-3.4
167114929,-2.8643942835\H,-4.3044891329,-4.7687382441,-3.1502679306\H,
-2.9649920072,-4.9590154091,-2.011533367\C,-4.0497462285,-0.4369360222
,-0.0959800539\N,-3.3564804786,0.6449416208,-0.5834085523\H,-2.4362614
506,0.4186613704,-0.9657948623\C,-3.5258689247,2.0093361707,-0.2815549
439\C,-3.687353188,4.7780519111,0.1850377762\C,-2.3702248837,2.7584976
909,-0.0454824114\C,-4.7733605274,2.6489386888,-0.2839747975\C,-4.8397
28819,4.0198904351,-0.0436283355\C,-2.4542779015,4.1332419648,0.175846
1503\H,-1.4099528193,2.2551076334,-0.0192777456\H,-5.6743261479,2.0848
895259,-0.4797826654\H,-3.7528067156,5.8447061411,0.3588802374\C,3.668
3719064,1.1809680885,-1.8077224695\N,4.9718498138,1.6832392076,-1.7526
890767\C,5.2955058912,2.067287661,-0.5563973997\S,4.1239176086,1.90704
49357,0.7486479887\C,2.9027942582,1.1954367919,-0.4556035823\O,3.14940
3724,0.7828065681,-2.8358240803\H,1.2404135055,-2.3237760661,-0.633321
2537\C,1.7193637129,2.1727412259,-0.5982523987\H,1.0524883159,1.836999
8763,-1.3968034158\H,2.0793240126,3.1683555297,-0.8698731221\H,1.15964
48209,2.2582826227,0.3346688881\C,6.6172836241,2.6395683212,-0.2402501
621\C,9.0295489456,3.6994725756,0.4514746145\C,7.5803326635,2.81050825
76,-1.24151951\C,8.8116782653,3.3523485732,-0.8804673097\H,7.351767157
8,2.5215203351,-2.2604612336\H,9.5861281236,3.5006779615,-1.6265849103
\H,9.9740041339,4.1243018046,0.7752815391\C,2.4635527565,-0.2518420081
,0.0228355306\C,1.4063145835,-0.9010124101,-0.88092\N,0.0802290603,-0.
4476919734,-0.6888144996\O,-0.3089505619,-0.0612445999,0.4393195853\O,

-0.737965889,-0.5422994542,-1.6481360918\H,1.6255614568,-0.8665691755,
-1.9453813024\H,1.9860786594,-0.099700067,0.9939543257\C,3.6542552645,
-1.1855063197,0.2462871515\C,3.9391237392,-1.639416883,1.5427431051\C,
4.4815075829,-1.6203142264,-0.802241389\C,5.010253531,-2.4993233866,1.
7896832937\H,3.321527437,-1.3041883789,2.371777267\C,5.5604145285,-2.4
730025732,-0.5564669005\H,4.2905417215,-1.2994360864,-1.8201654274\C,5
.8285180897,-2.917494779,0.7388498009\H,5.211149613,-2.8311435579,2.80
43244336\H,6.190548779,-2.7890980262,-1.3829760973\H,6.6683897892,-3.5
79601988,0.9281148576\C,-6.182422629,4.6996961458,0.0080307058\C,-1.17
40493805,4.8881346782,0.4050265179\F,-7.1189057736,4.0344513696,-0.701
8396352\F,-6.1250319034,5.9598264632,-0.4831520945\F,-6.646885818,4.80
04669209,1.275766684\F,-0.4723315209,4.3744152743,1.4446655271\F,-0.35
42494644,4.8181294607,-0.6744931401\F,-1.3798088064,6.194396802,0.6656
339562\S,-5.3180386041,-0.3543147033,1.0134430467\N,6.8183443333,2.972
4489882,1.0451976704\C,8.0010799913,3.4903584252,1.3765415968\H,8.1375
098785,3.751995038,2.4232835418\\Version=ES64L-G09RevD.01\State=1-A\HF
=-3710.8364478\RMSD=2.588e-09\RMSF=8.943e-07\Dipole=5.9194107,-2.55729
43,-0.3602981\Quadrupole=9.1534184,-0.0374494,-9.115969,10.6816339,12.
1741268,4.871166\PG=C01 [X(C42H51F6N7O4S2)]\\@

TS2’A-***S,S***

1\1\GINC-R2615\FTS\RB3LYP\6-31G(d,p)\C42H51F6N7O4S2\ROOT\07-Mar-2016\0
\\# freq=noraman b3lyp/6-31G(d,p) opt=(ts,calcfc,noeigen,maxcyc=200) s
cf=maxcyc=200 scrf=(smd,solvent=toluene)\\TS for protonation of int1DA
 conf. 2: S(elec),S(nuc) (tol opt; cat III)\\0,1\C,0.2416416681,3.8266
590114,0.3338847397\O,-0.4729820406,4.8284151799,0.3938936957\C,1.5866
271949,3.8154212678,-0.4578321139\H,2.3346230751,4.230631042,0.2276024
873\N,-0.0439550594,2.6581942131,0.9656795562\H,0.6234781421,1.9104375
727,0.846581504\C,-1.1408520008,2.3347373416,1.8919051784\H,-1.1225816
301,1.2411225553,1.9358628944\C,-0.864132442,2.8018322,3.3719303526\C,
0.5378878713,2.3012942016,3.7751824704\H,1.328275467,2.7552323196,3.17
12609908\H,0.6200757596,1.2122619351,3.6742059476\H,0.7396315824,2.550
3417564,4.8221929151\C,-0.9244248608,4.3309692306,3.5450413093\H,-0.20
49869698,4.847556841,2.9069319023\H,-0.7035761989,4.5919066044,4.58651
58961\H,-1.9167323815,4.7322808502,3.314313515\C,-1.8930602219,2.14435
15099,4.3172238091\H,-2.8965563051,2.5674834774,4.2090027401\H,-1.5926
709042,2.3043954712,5.3583136756\H,-1.9684351447,1.0644365567,4.152570
3104\C,-2.5328416956,2.7254296617,1.3733391607\H,-3.2563437265,2.40126
2659,2.1204581517\H,-2.6143917828,3.8074387081,1.2437648001\N,-2.95336
51941,2.0712091771,0.0911181233\C,-2.4969526554,2.776030499,-1.1562017
524\H,-1.4525466974,2.5545371354,-1.3431323046\H,-2.607698932,3.852469
3296,-0.9931986706\C,-3.4334171251,2.2699687271,-2.2635787641\H,-3.640
9904702,3.0664552977,-2.9821217296\H,-2.9668064383,1.4496499686,-2.814
4537016\C,-4.4452851506,2.1113810312,-0.0477133315\H,-4.7733176969,3.1
306860067,0.1943469491\H,-4.9120727722,1.4238780071,0.6564419541\C,-4.
7114016133,1.7923536148,-1.5222298038\H,-5.6159017707,2.2955385508,-1.
872410126\H,-4.8637660643,0.7192087469,-1.6650151779\N,2.0182479913,2.
4417238261,-0.7091889326\H,1.3960359164,1.8545506772,-1.2642256401\C,1
.6120824474,4.7105719318,-1.7521093082\C,1.5798112317,6.1984999801,-1.
3463520338\H,1.6369021517,6.8287185787,-2.2414561066\H,2.4394864016,6.
449529975,-0.7138484967\H,0.6697426638,6.4464363892,-0.7999882393\C,2.
9299613238,4.4531522987,-2.5101159289\H,3.0128524218,5.1557671511,-3.3
46545259\H,3.801454429,4.5924412635,-1.8639096488\H,2.9772243267,3.441
5469957,-2.924274222\C,0.4322398276,4.3993984956,-2.6907218074\H,0.399
7692563,3.3388580101,-2.9684722511\H,0.5319608647,4.969773858,-3.62098
9623\H,-0.522364105,4.6713356533,-2.2350129467\C,3.1636488621,1.866328
9302,-0.2490795721\N,3.1737422165,0.5062635893,-0.4838494988\H,2.24761
10005,0.0797857559,-0.5287896851\C,4.2403819742,-0.3965938496,-0.34175
52009\C,6.2899312329,-2.3152976797,-0.1280090064\C,3.9553573467,-1.693
2566154,0.1016063306\C,5.5571615537,-0.0681306227,-0.6995630064\C,6.56
33932532,-1.0221340385,-0.5799455952\C,4.9775344172,-2.6400356314,0.20
25604778\H,2.9384050722,-1.9585591098,0.3727673971\H,5.788071393,0.920
7507503,-1.0687417489\H,7.0797282436,-3.0504949219,-0.0424792184\C,-4.
2191726716,-0.9725574426,1.5579297777\N,-5.5153444315,-0.8721738976,1.
036599587\C,-5.6974193112,-1.6795220602,0.0356428924\S,-4.3776420247,-
2.7312214149,-0.4767541984\C,-3.3749015859,-2.1240196442,0.9638338679\
O,-3.790968058,-0.2415045074,2.4321259138\H,-2.489200297,0.8181539741,
-0.011332473\C,-3.3407151614,-3.229846202,2.0431999243\H,-2.8221661639
,-2.8502318759,2.9293490942\H,-2.8218457333,-4.1197577077,1.6847225718
\H,-4.3547869316,-3.5154947334,2.3365366025\C,-6.9747908073,-1.7579287
939,-0.6970138695\C,-9.2856388022,-1.9908596759,-2.1212315831\C,-8.084
4437732,-1.0070422473,-0.2917605163\C,-9.263279865,-1.1327143207,-1.02
36405827\C,-8.1216160372,-2.6961995505,-2.446394984\H,-8.0084293327,-0
.3568215524,0.5717399776\H,-10.1478659924,-0.5708827152,-0.7406062269\
H,-8.1052144742,-3.3742556307,-3.2964340011\H,-10.1833868498,-2.118837
0265,-2.7169367286\C,-1.932594415,-1.648563573,0.6036580254\C,-1.88527
97017,-0.4680884166,-0.3852513119\N,-0.549129281,-0.0645888474,-0.6602

257239\O,0.3292778845,-0.2019290096,0.2308734328\O,-0.2730672322,0.493
8390202,-1.7466659132\H,-2.3717483807,-0.652056211,-1.3429587067\H,-1.
5394108531,-1.2923112662,1.5601946977\C,-1.0456986675,-2.8174755834,0.
1600796269\C,-0.1243014948,-3.3739316886,1.0580425042\C,-1.1161172369,
-3.3515125819,-1.1350485018\C,0.6987258858,-4.4355646283,0.6798860104\
H,-0.0425762834,-2.9617675578,2.0602265891\C,-0.2974934759,-4.41678220
45,-1.5155289312\H,-1.8023353034,-2.9288059808,-1.8627591987\C,0.61478
26304,-4.9605620004,-0.6109903677\H,1.4197258576,-4.8363597462,1.38455
65674\H,-0.3678555149,-4.8134084915,-2.5243409381\H,1.2636529383,-5.77
80073965,-0.9099938065\C,7.9844998754,-0.6451576457,-0.9047759394\C,4.
6328709301,-4.0335525195,0.6531904815\F,8.0527753275,0.3619497269,-1.8
021174016\F,8.6611464071,-0.2388188867,0.1950356111\F,8.6752491924,-1.
6896746665,-1.4190748967\F,3.8553816676,-4.0238158533,1.7645299979\F,3
.938596776,-4.7132662936,-0.2923733846\F,5.7278264283,-4.7681620947,0.
9405141792\S,4.3857613068,2.7100767013,0.5553757723\N,-6.9884074931,-2
.588023179,-1.7534303018\\Version=ES64L-G09RevD.01\State=1-A\HF=-3710.
8375027\RMSD=4.463e-09\RMSF=1.310e-06\Dipole=-7.1561926,-1.0594188,-1.
2302585\Quadrupole=21.5025381,-18.5386161,-2.963922,-5.4132273,13.8453
829,-2.3033073\PG=C01 [X(C42H51F6N7O4S2)]\\@

int2’A-*R,R*

1\1\GINC-R2461\FOpt\RB3LYP\6-31G(d,p)\C42H51F6N7O4S2\ROOT\13-Mar-2016\
0\\# freq=noraman b3lyp/6-31g(d,p) opt=maxcyc=200 scf=maxcyc=200 scrf=
(smd,solvent=toluene) iop(1/8=1)\\Conjugate addition-protonation produ
ct conf. 1: R(elec),R(nuc) (tol opt; cat III)\\0,1\C,-3.402912635,-1.5
754347151,1.0133751139\O,-4.3062359998,-2.2476391766,1.512422864\C,-2.
451760219,-0.7795495548,1.9491885297\H,-2.1826604738,-1.4905608918,2.7
32527785\N,-3.1690745474,-1.461865356,-0.3155553078\H,-2.378173639,-0.
8982389746,-0.5849131666\C,-3.9681308765,-1.9966020314,-1.4331515731\H
,-3.6504217087,-1.388313137,-2.2875148737\C,-3.5950306915,-3.472580153
3,-1.8259710359\C,-2.0602764525,-3.5891117982,-1.9113709724\H,-1.58782
95269,-3.4422709087,-0.9369133204\H,-1.6389953141,-2.85369347,-2.60899
64205\H,-1.7751700445,-4.5834809964,-2.2721119016\C,-4.1238945783,-4.5
096833877,-0.817621334\H,-3.7661679219,-4.3104028782,0.1944993863\H,-3
.7939150154,-5.5142003003,-1.1077891606\H,-5.2183300092,-4.5229765822,
-0.7840860051\C,-4.1697533992,-3.7841621629,-3.2248885893\H,-5.2634584
43,-3.806270664,-3.2359198753\H,-3.8235112502,-4.7679842115,-3.5605536
056\H,-3.8409645151,-3.0488287211,-3.9695282228\C,-5.4746309787,-1.749
0244003,-1.2262035331\H,-6.0205768717,-2.2100298168,-2.0688592594\H,-5
.7924819521,-2.2460903744,-0.3093011419\N,-5.8178222614,-0.3341601731,
-1.1085454687\C,-7.2143732956,-0.1234132511,-0.7128845267\H,-7.3744780
784,-0.4512057734,0.3191667713\H,-7.9068610093,-0.70114722,-1.35760336
61\C,-7.4350785952,1.3832475589,-0.9209842854\H,-8.4707037873,1.605045
7636,-1.1938684866\H,-7.2140706406,1.9320966394,-0.0010658106\C,-5.662
2780942,0.456129082,-2.3351680585\H,-6.1166342587,-0.0623908296,-3.203
3408942\H,-4.6076627238,0.6276617711,-2.5691820766\C,-6.4245653325,1.7
636187381,-2.0395020254\H,-6.9170781006,2.145200958,-2.938408812\H,-5.
7392879998,2.5420983144,-1.6912303315\N,-1.195187167,-0.3925605776,1.3
03891243\H,-1.1962417494,0.4759853664,0.7804484692\C,-3.14464329,0.455
9343395,2.6354853078\C,-4.3108243221,-0.0271851594,3.5203289669\H,-4.7
502132241,0.8304405745,4.0425586241\H,-3.9655502885,-0.739554896,4.277
2538034\H,-5.0906388667,-0.5182374971,2.9378039346\C,-2.1167754003,1.1
598870569,3.5447488164\H,-2.6053114592,1.967807339,4.1003403854\H,-1.6
868940306,0.4653113569,4.2746298818\H,-1.2906476176,1.6038190231,2.980
3519265\C,-3.6837909532,1.4543097747,1.5927706464\H,-2.8839491502,1.88
57009326,0.9793474305\H,-4.1806081278,2.289453789,2.0994710528\H,-4.40
53921923,0.988594002,0.9139115858\C,-0.0036642192,-1.0356969957,1.4542
164512\N,1.0443778354,-0.3396524825,0.8927868701\H,0.8790864222,0.6417
325378,0.6784436714\C,2.398615287,-0.7092539622,0.7739316721\C,5.13681
92413,-1.2818222947,0.4587267371\C,3.3559463673,0.3074853446,0.8909278
195\C,2.816731306,-2.0152712301,0.4808315005\C,4.1761601974,-2.2872122
066,0.3386039089\C,4.7099416655,0.0171653356,0.7254436572\H,3.04079221
25,1.3212713431,1.1103388423\H,2.0897843063,-2.8075918972,0.3784975745
\H,6.1906395556,-1.5084002945,0.353694013\C,1.6920485433,3.1736696452,
-0.8750319033\N,2.9529705175,3.6938242661,-0.6510921405\C,3.4138340348
,4.3207145993,-1.6976021682\S,2.4285854449,4.4045675855,-3.1505165074\
C,1.1204301585,3.4036030227,-2.2986504437\O,1.0669764762,2.53347238,-0
.0339908094\H,-2.2442996215,4.1731579991,-1.4967527358\C,1.0238136621,
2.0244153931,-2.9868668687\H,0.419541982,1.3406867033,-2.3865348369\H,
2.0171116517,1.5794731721,-3.0856893073\H,0.5870180167,2.1087918065,-3
.9849436446\C,4.7355611254,4.9638117958,-1.722975032\C,7.1555673126,6.
1957759197,-1.8968318989\C,5.5874778557,4.9086321371,-0.6141269258\C,6
.8245755803,5.5432820682,-0.711697272\H,5.2826769072,4.3760520693,0.27
7624474\H,7.5170127389,5.5248467033,0.1238559534\H,8.1087813092,6.7007
503041,-2.013502001\C,-0.240750392,4.1936209498,-2.3046941807\C,-1.380
2711563,3.5059074676,-1.5220966123\N,-1.9064240521,2.2541908664,-2.178
7838835\O,-2.2680158863,2.3276345748,-3.3466313234\O,-1.9784604376,1.2

343187799,-1.4883214321\H,-1.0914997935,3.2198964702,-0.5159691104\H,-
0.5628840804,4.2371353921,-3.3496507089\C,-0.1328213456,5.6315217891,-
1.7965795993\C,-0.3397936035,6.6928354968,-2.6883917104\C,0.1285461099
,5.9269543571,-0.4498521819\C,-0.274383555,8.0159771618,-2.2530569673\
H,-0.5488494618,6.4802312946,-3.733562222\C,0.1954726278,7.2515290905,
-0.0142920168\H,0.2781694557,5.1301449375,0.2718501905\C,-0.0043650346
,8.2992069785,-0.9134308553\H,-0.4340173117,8.8236820674,-2.9612282656
\H,0.401026954,7.4611302655,1.0312496263\H,0.0468836306,9.3288687008,-
0.5722258603\C,4.6211755244,-3.6791270133,-0.0255478245\C,5.7145713669
,1.1342155403,0.7824417395\F,3.7384661288,-4.6202051009,0.3649366075\F
,5.8152793601,-3.9844250632,0.5319128059\F,4.7756281962,-3.8172115703,
-1.366383565\F,5.8862952015,1.7158154807,-0.4324560078\F,5.3376099172,
2.1215613798,1.6297751322\F,6.9323562534,0.7068761021,1.1808506136\S,0
.1548735388,-2.5091633975,2.2635831013\N,5.047573852,5.5884591609,-2.8
718462287\C,6.2331097206,6.1891268591,-2.9504436229\H,6.4625034794,6.6
865178745,-3.8897633708\\Version=ES64L-G09RevE.01\State=1-A\HF=-3710.8
600045\RMSD=6.700e-09\RMSF=1.885e-06\Dipole=1.4688658,6.2017479,-2.451
0913\Quadrupole=11.7116175,0.7629992,-12.4746167,15.7469216,2.5435149,
11.6560419\PG=C01 [X(C42H51F6N7O4S2)]\\@

int2’A-*S,S*

1\1\GINC-R3432\FOpt\RB3LYP\6-31G(d,p)\C42H51F6N7O4S2\ROOT\07-Mar-2016\
0\\# freq=noraman b3lyp/6-31g(d,p) opt=maxcyc=200 scf=maxcyc=200 scrf=
(smd,solvent=toluene)\\Conjugate addition-protonation product conf. 2:
 S(elec),S(nuc) (tol opt; cat III)\\0,1\C,0.4562490917,-4.235874325,-0
.3624831532\O,1.2261175969,-5.1955527697,-0.3394156422\C,-0.8170112026
,-4.2509286892,-1.2663807798\H,-1.5509656846,-4.8556280504,-0.72288614
37\N,0.610708188,-3.1190774775,0.3973000042\H,-0.1358729303,-2.4437752
037,0.3334856184\C,1.5813717339,-2.8638981411,1.482973781\H,1.52815273
05,-1.7791811868,1.6278519675\C,1.1384291001,-3.48365874,2.8607514342\
C,-0.3143508829,-3.051900926,3.1474151083\H,-1.0165363881,-3.453262800
5,2.4108575219\H,-0.4154096865,-1.959424583,3.1549713877\H,-0.63056191
22,-3.4165545712,4.1305597892\C,1.2203002369,-5.0214562359,2.885714413
9\H,0.5753219643,-5.480952194,2.1337412864\H,0.9082422694,-5.392612915
6,3.8692625275\H,2.2389500134,-5.3800040711,2.7078381748\C,2.031546587
7,-2.910744309,3.9843693956\H,3.0433731194,-3.3283007969,3.969620286\H
,1.6015882903,-3.156328727,4.9617604454\H,2.1179051003,-1.8201967211,3
.9222313848\C,3.040624769,-3.155828624,1.0897371021\H,3.6364391119,-3.
0007389965,1.9911054375\H,3.1592275888,-4.2043571087,0.7883596612\N,3.
5856260706,-2.2475124751,0.06366008\C,3.4110082057,-2.706957693,-1.327
084712\H,2.3775836981,-2.5809426093,-1.6499727288\H,3.6614211249,-3.77
8475049,-1.4174380637\C,4.3992039012,-1.8554827402,-2.1440294012\H,4.8
15671362,-2.4278763524,-2.9774284391\H,3.8983862076,-0.9831239012,-2.5
741261266\C,5.0459710398,-2.0893849465,0.1972506798\H,5.5422864125,-3.
0720241681,0.3044030886\H,5.2827733677,-1.4982270347,1.0876383534\C,5.
4827620121,-1.4238067675,-1.1175995638\H,6.488749382,-1.7387201591,-1.
4088969023\H,5.5034263006,-0.3348722798,-1.016262952\N,-1.4137723937,-
2.9192558515,-1.3596821439\H,-0.8857010764,-2.222713239,-1.8760872026\
C,-0.618996142,-4.9197088142,-2.6784308489\C,-0.4266379868,-6.44054664
76,-2.5067139292\H,-0.32783456,-6.9146211901,-3.4900722132\H,-1.295229
518,-6.8897977234,-2.0108909162\H,0.4589684151,-6.6677988429,-1.914151
0099\C,-1.8896469702,-4.6970161359,-3.5239013847\H,-1.8014681324,-5.24
97440407,-4.4655338748\H,-2.7861790749,-5.0541938384,-3.0081448621\H,-
2.0441613877,-3.6435218606,-3.7765656026\C,0.5892796376,-4.3221739855,
-3.4223223688\H,0.495047892,-3.236998652,-3.5556330853\H,0.668453899,-
4.7583866234,-4.4242671416\H,1.5235296404,-4.5269612426,-2.8944928246\
C,-2.656588384,-2.5612831397,-0.9288730361\N,-2.8633296737,-1.20268361
65,-1.059784425\H,-2.010850585,-0.6483830376,-1.0791950503\C,-4.022515
689,-0.4597440211,-0.7640627051\C,-6.2503223273,1.158530254,-0.1925290
649\C,-3.8598813186,0.7687156086,-0.1125003398\C,-5.3054934097,-0.8704
044867,-1.1467886003\C,-6.4026733208,-0.0622122806,-0.8507752703\C,-4.
9668103217,1.5682044401,0.1655775889\H,-2.8689114859,1.0893619181,0.18
82046523\H,-5.4447742151,-1.8112567804,-1.6602950622\H,-7.1126096768,1
.7699924499,0.0431327439\C,3.2490979081,1.4063523821,2.3074692424\N,4.
6416874899,1.2728310043,2.3084485651\C,5.205795465,2.0715151003,1.4573
022162\S,4.2000475198,3.1564267064,0.4839561256\C,2.7105665171,2.56316
857,1.4133120283\O,2.4961200071,0.7142135607,2.9639588327\H,2.56584364
53,0.1163051717,0.0830578856\C,2.2210243438,3.6745152038,2.3635276779\
H,1.4002671134,3.2902529058,2.9773237741\H,1.8690504483,4.5458350721,1
.8087387964\H,3.0268209963,3.9919809136,3.0308968601\C,6.665316579,2.1
300533843,1.2608971716\C,9.3441629075,2.3297429458,0.8121517118\C,7.52
81074429,1.3407588226,2.0300712205\C,8.8972629119,1.4487297538,1.79489
50709\C,8.399702892,3.0740527201,0.096359483\H,7.1219803126,0.67483362
77,2.7824274514\H,9.6014299734,0.8555770307,2.3697541947\H,8.714558039
4,3.7702328737,-0.6773390114\H,10.4016660867,2.4447238356,0.5985577196
\C,1.5415502429,2.0405575596,0.5209632554\C,1.9866569512,0.9065005807,
-0.4158401456\N,0.7934514532,0.1608953464,-0.9616750981\O,-0.166329421

```
5,-0.0310190888,-0.2066846444\O,0.8464180863,-0.256190885,-2.112387263
\H,2.5360201287,1.2423742617,-1.2928026276\H,0.8607191455,1.5881637087
,1.2479068517\C,0.7770337068,3.1289146177,-0.2274143493\C,-0.515045535
2,3.4776939201,0.1901272078\C,1.3202945998,3.7916776606,-1.3391694206\
C,-1.2456157595,4.4613834715,-0.4788861871\H,-0.9576900477,2.973885359
,1.0447626039\C,0.5946229574,4.7797861245,-2.00544282\H,2.3146044692,3
.5410389373,-1.6974509079\C,-0.690691282,5.1166882903,-1.5782564863\H,
-2.2498152031,4.7023598198,-0.1468300984\H,1.0338399016,5.2821277174,-
2.862393671\H,-1.257340788,5.8811673047,-2.1015726758\C,-7.7718064909,
-0.48356942,-1.31658237\C,-4.7751581921,2.9021545891,0.8337772297\F,-8
.0272989463,-0.0394924816,-2.5719914176\F,-7.9110553003,-1.8250747085,
-1.3421794329\F,-8.7481413621,0.0120157998,-0.5238660724\F,-3.63431040
22,2.9498695914,1.5632770513\F,-4.6949768654,3.9132047726,-0.069848979
8\F,-5.7956977949,3.2055709472,1.6619790054\S,-3.774119613,-3.63523732
88,-0.2667245524\N,7.0877730379,2.9816976672,0.3113626551\\Version=ES6
4L-G09RevD.01\State=1-A\HF=-3710.8615141\RMSD=2.730e-09\RMSF=6.706e-06
\Dipole=5.4335346,3.2491989,-0.4477604\Quadrupole=24.9850364,-19.40095
46,-5.5840818,3.3260841,3.5229255,-0.877588\PG=C01 [X(C42H51F6N7O4S2)]
\\@
```

16. Copies of NMR Spectra

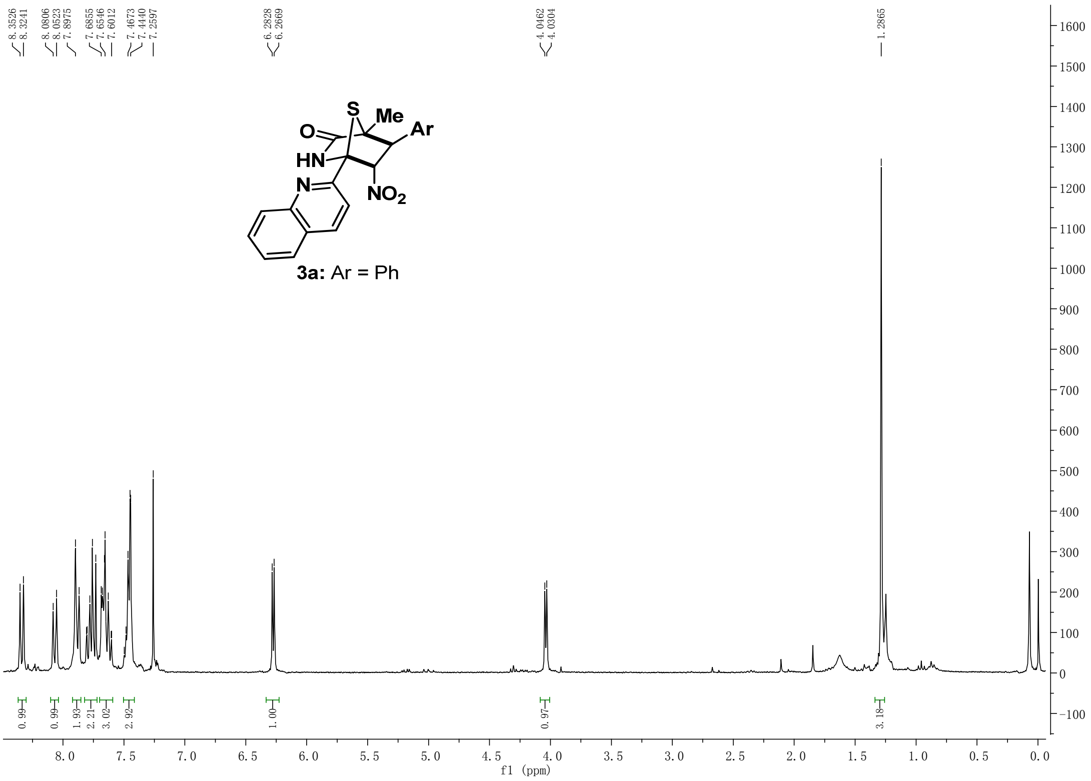

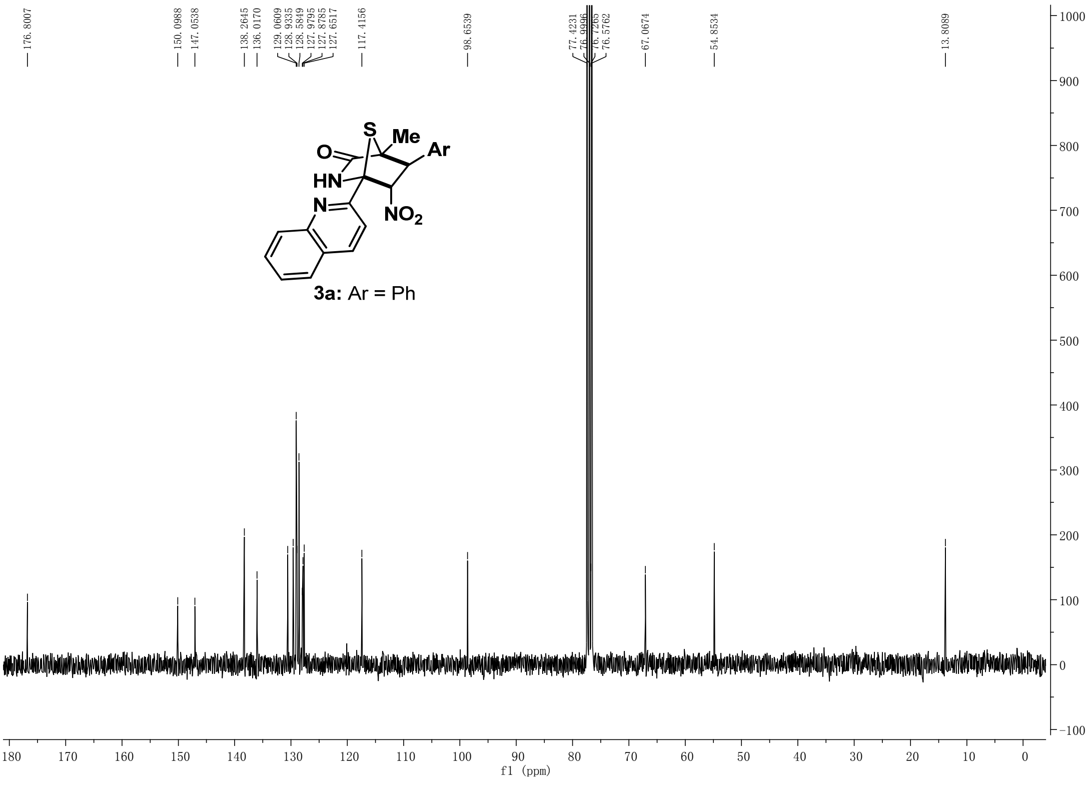

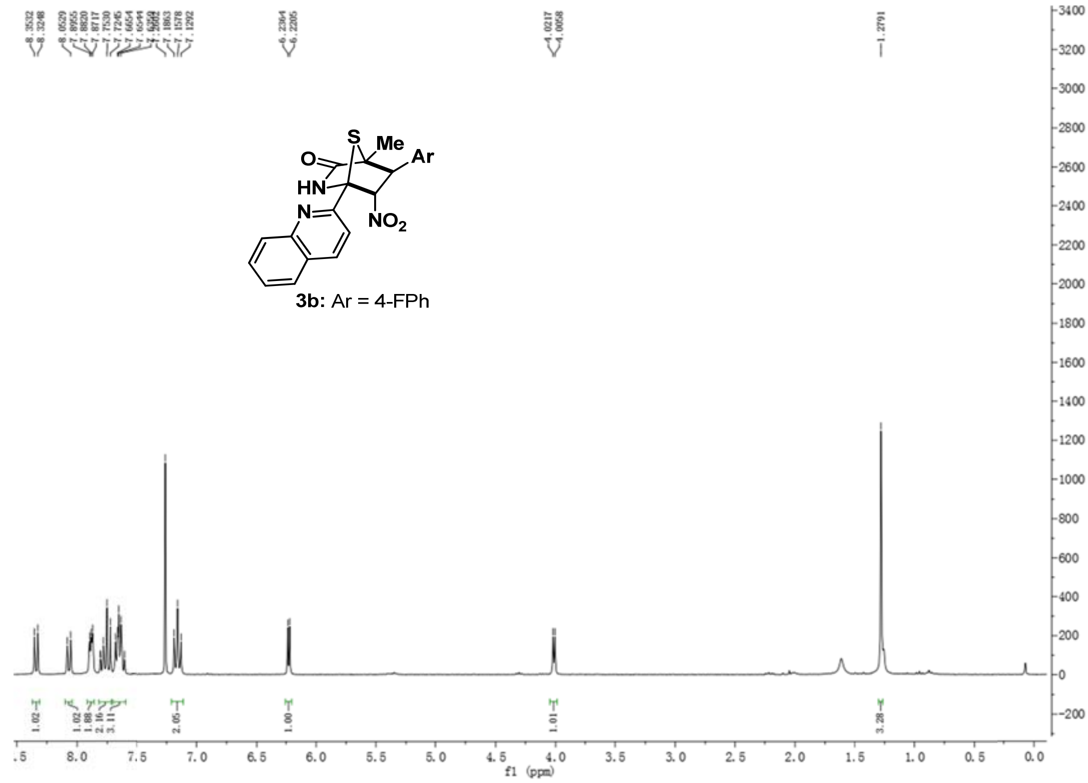
S
Me
Ar
O
HN
N
NO2
3b: Ar = 4-FPh
f1 (ppm)

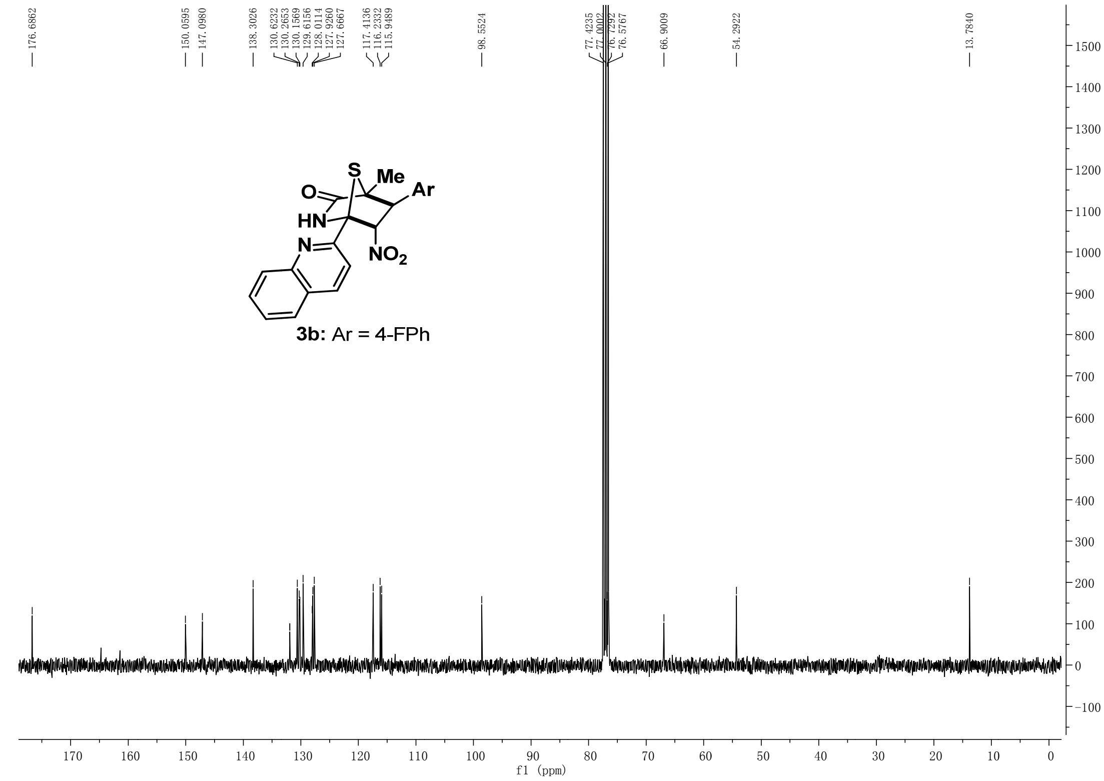
176.6862
150.0595
147.0980
138.3026
98.5524
66.9009
54.2922
13.7840
S
Me
Ar
O
HN
N
NO2
3b: Ar = 4-FPh
f1 (ppm)

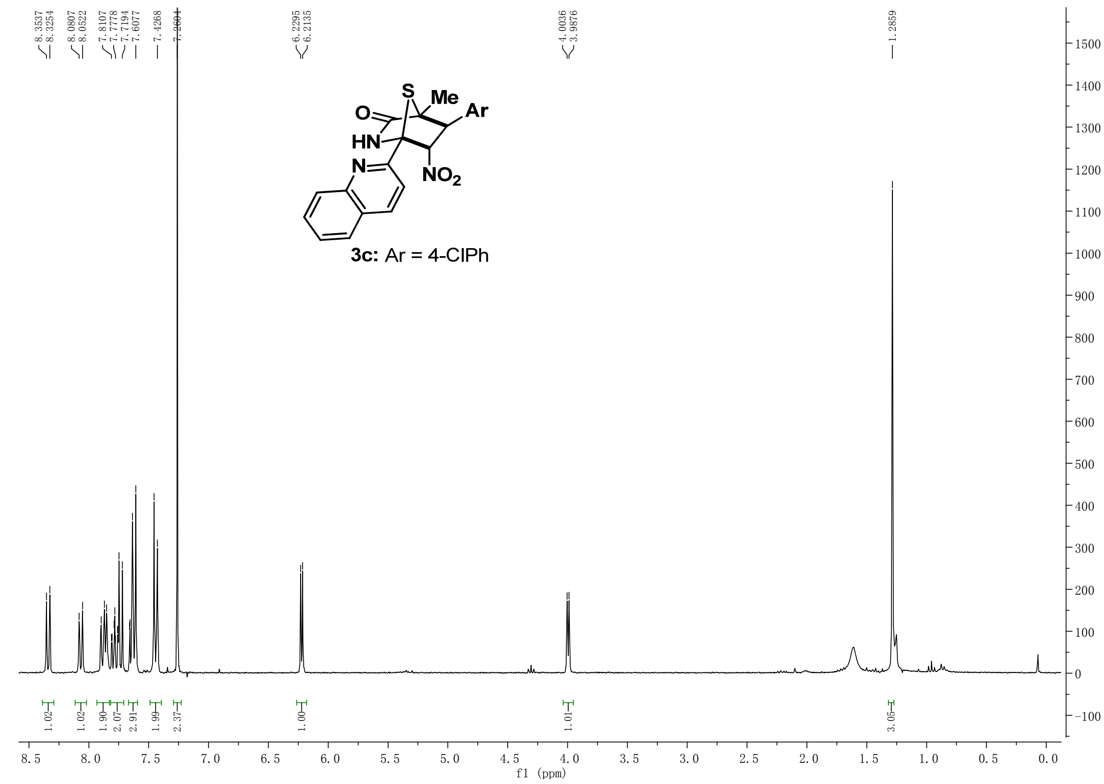

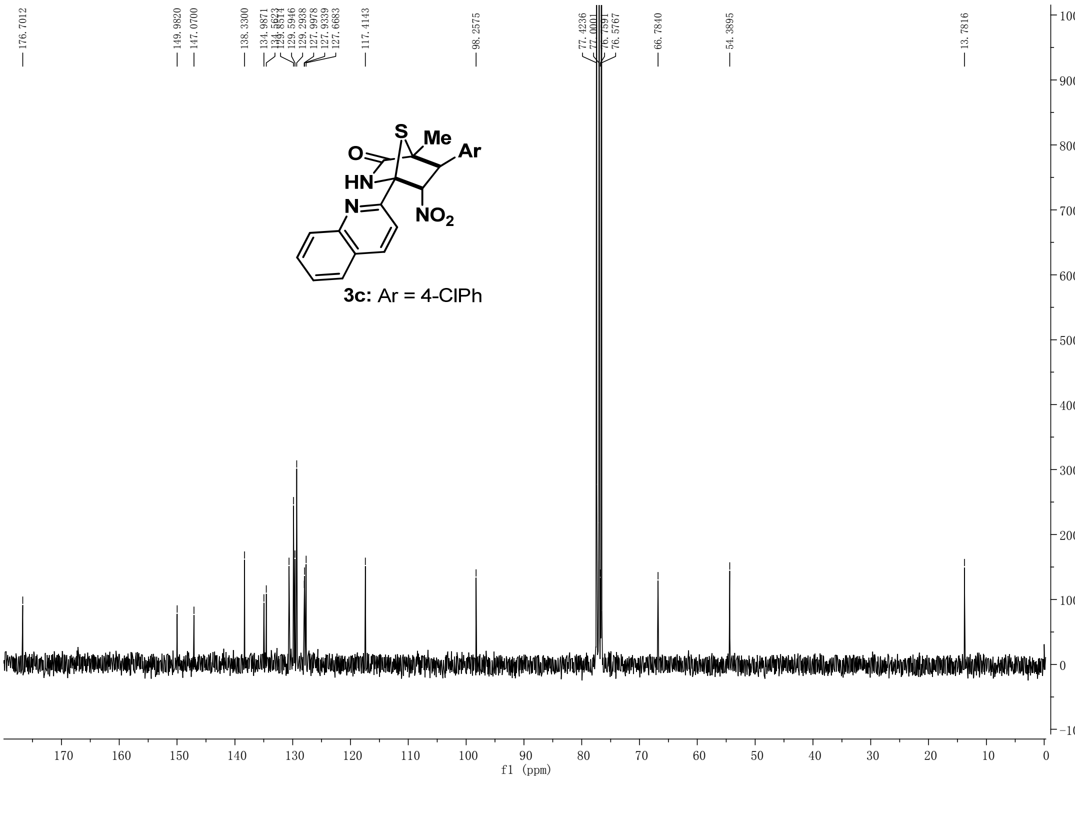

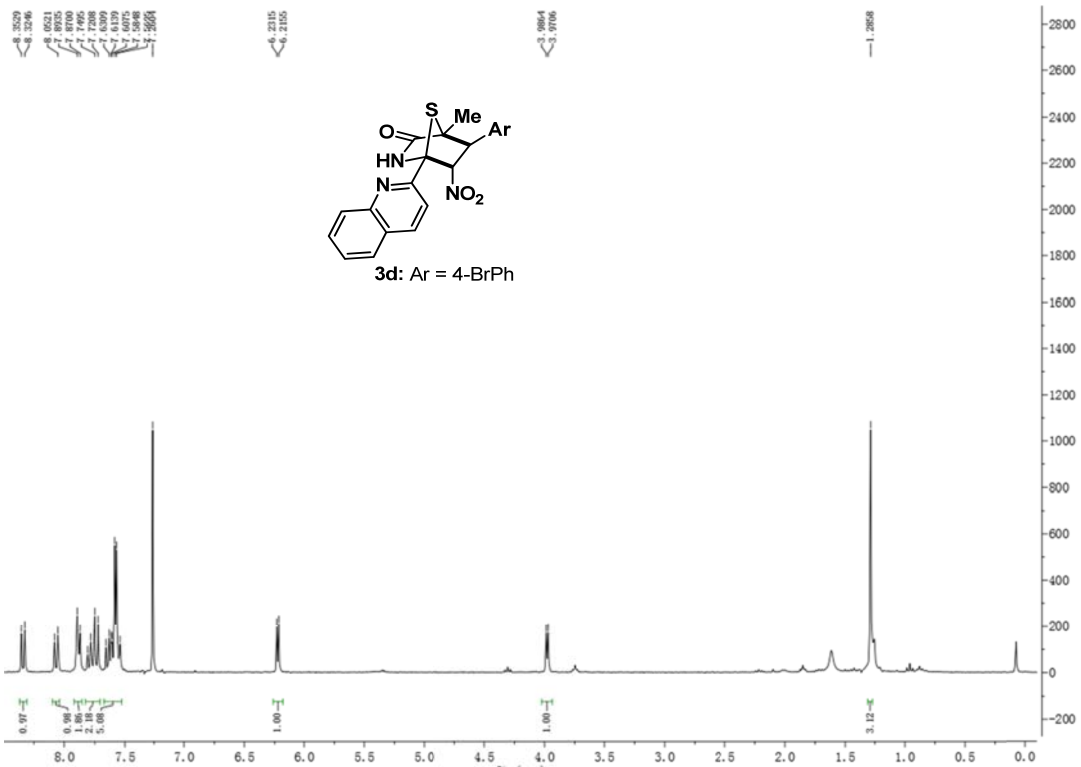
3d: Ar = 4-BrPh
f1 (ppm)

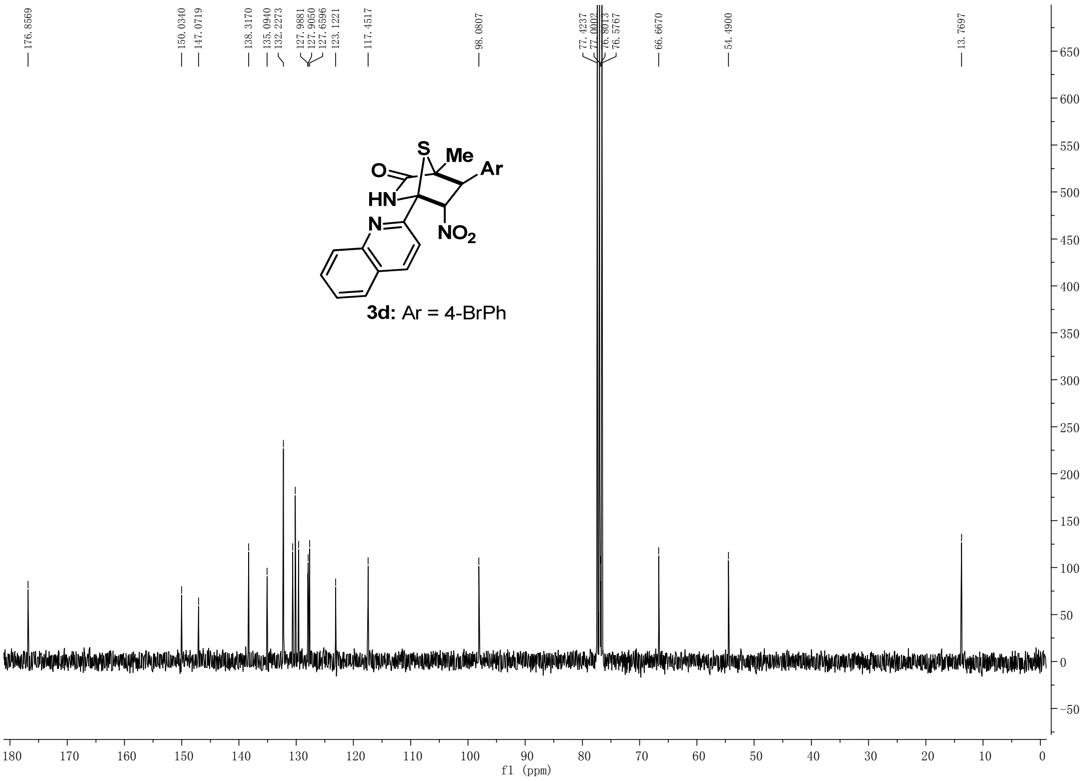
3d: Ar = 4-BrPh
f1 (ppm)

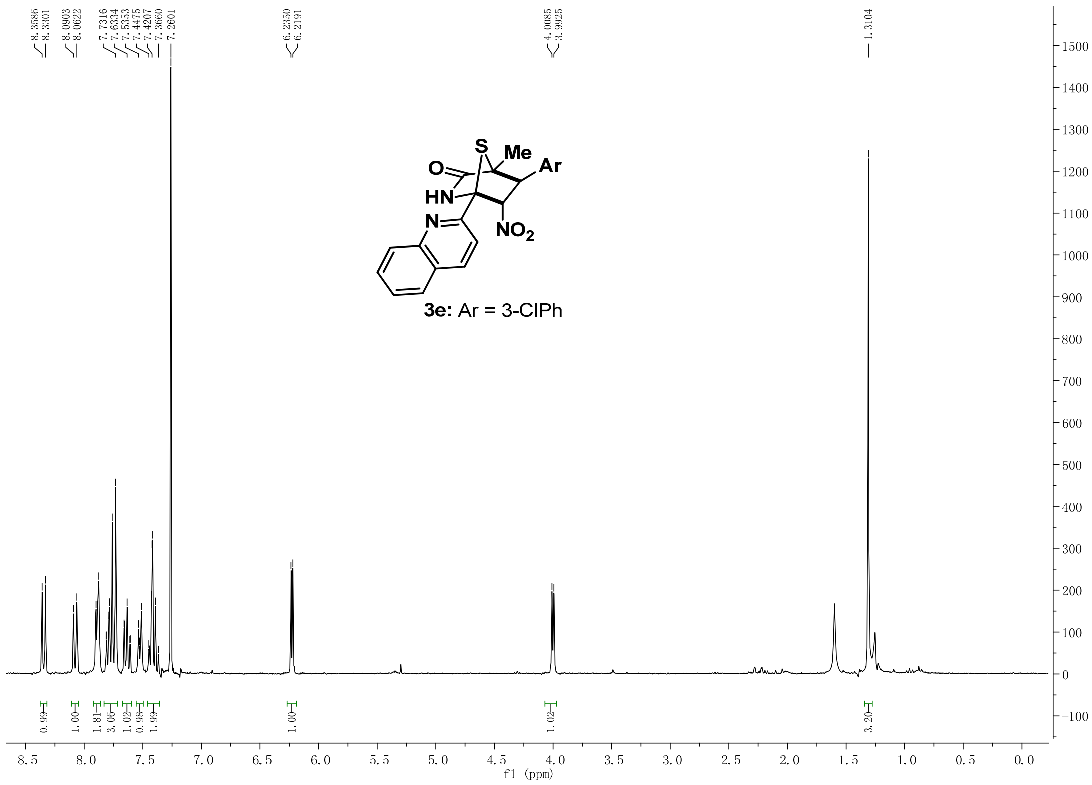
3e: Ar = 3-ClPh

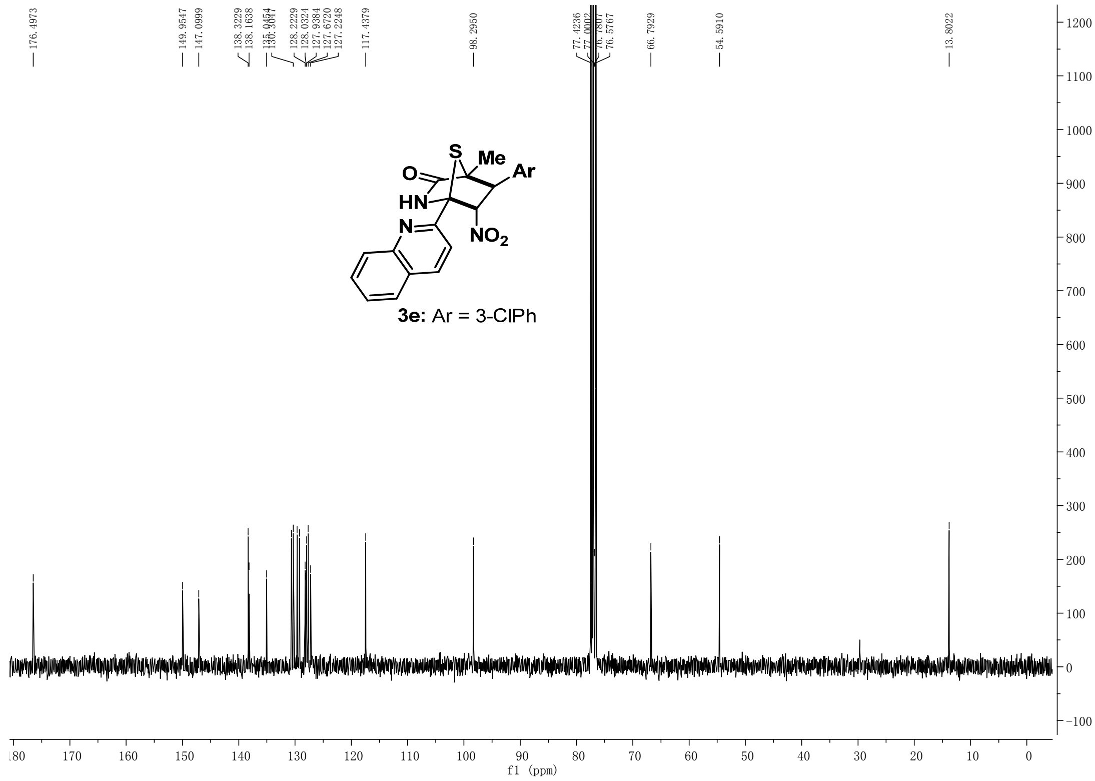
3e: Ar = 3-ClPh

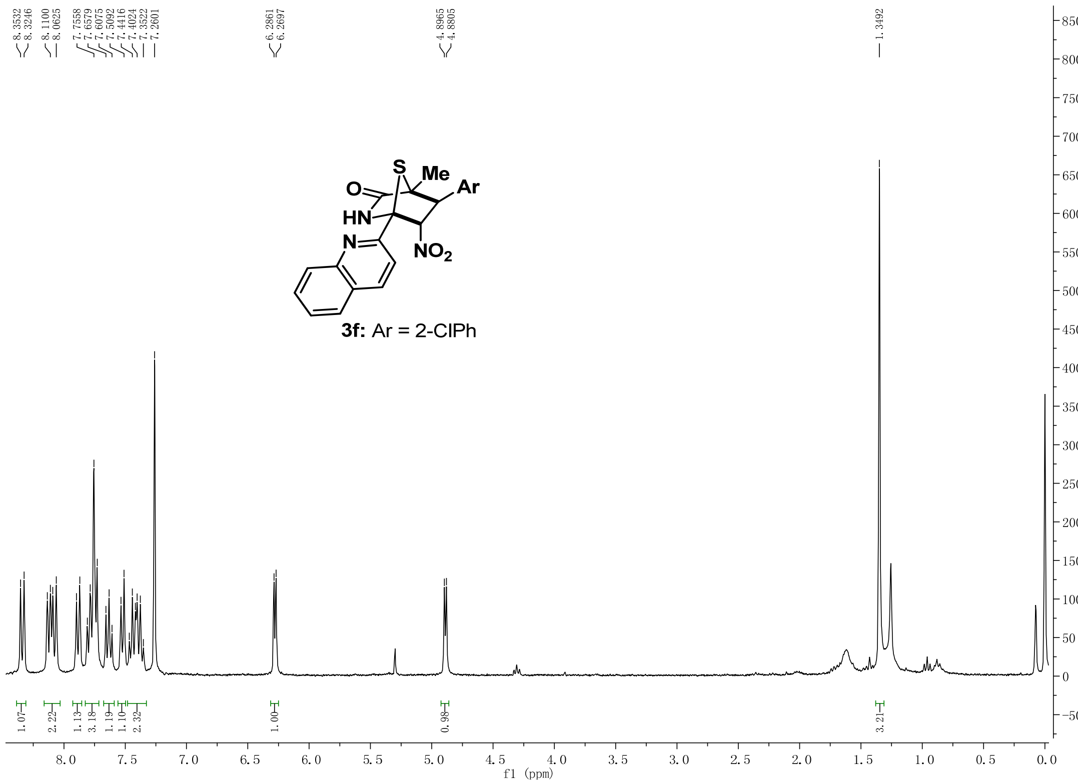
8.3532
8.3246
8.1100
8.0625
7.7558
7.6579
7.6075
7.5092
7.4416
7.4024
7.3522
7.2601
6.2861
6.2697
4.8965
4.8805
1.3492
3f: Ar = 2-ClPh
1.07
2.22
1.13
3.18
1.19
1.10
2.32
1.00
0.99
3.21
f1 (ppm)

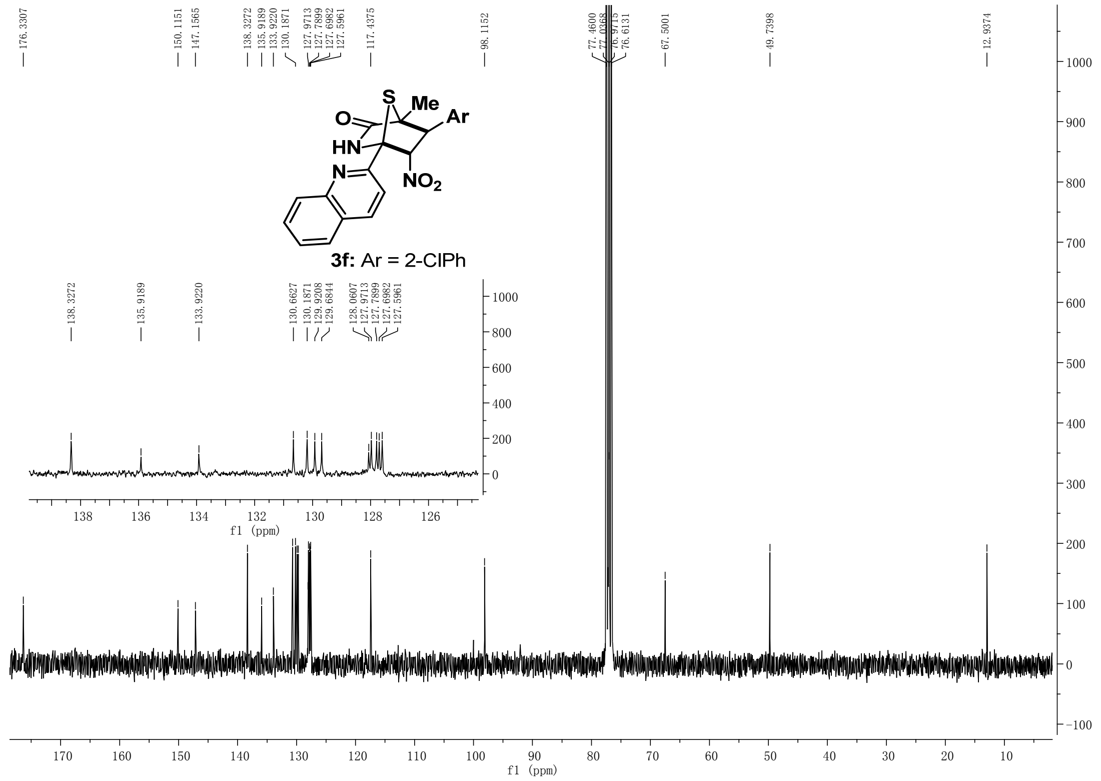
176.3307
150.1151
147.1565
138.3272
135.9189
133.9220
130.1871
127.9713
127.7899
127.6982
127.5961
117.4375
98.1152
77.4600
76.6131
67.5001
49.7398
12.9374
3f: Ar = 2-ClPh
130.6627
129.9208
129.6844
128.0607
f1 (ppm)

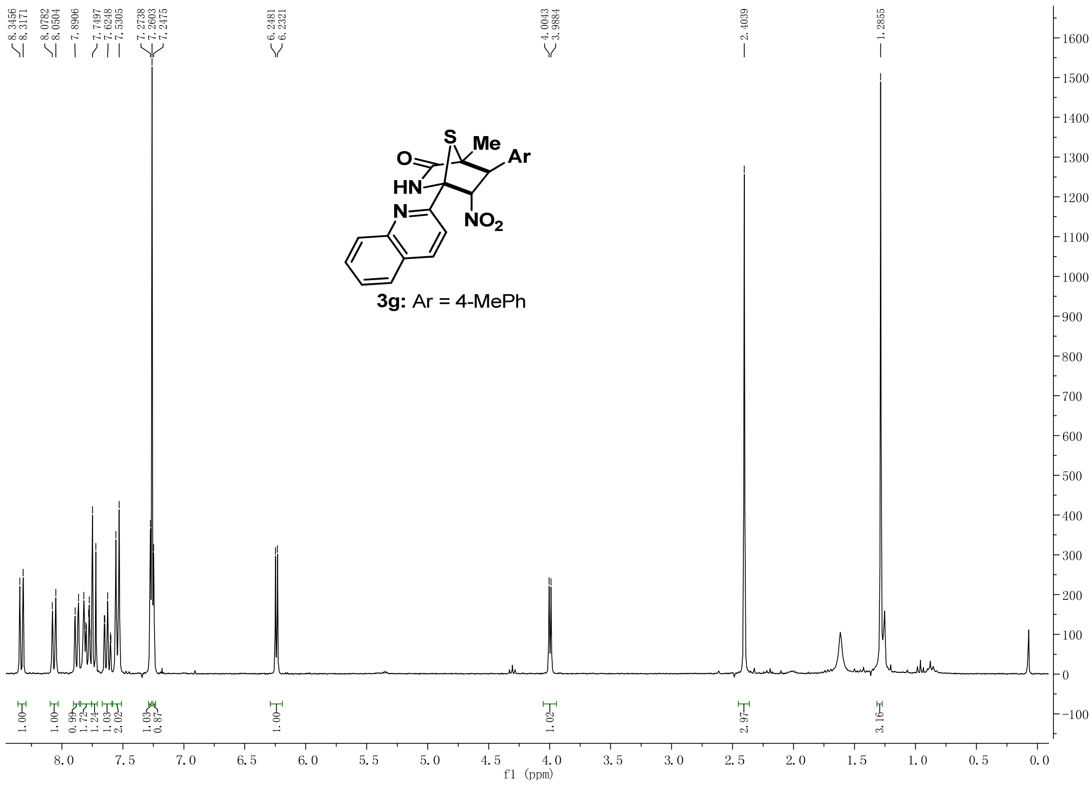
3g: Ar = 4-MePh
f1 (ppm)

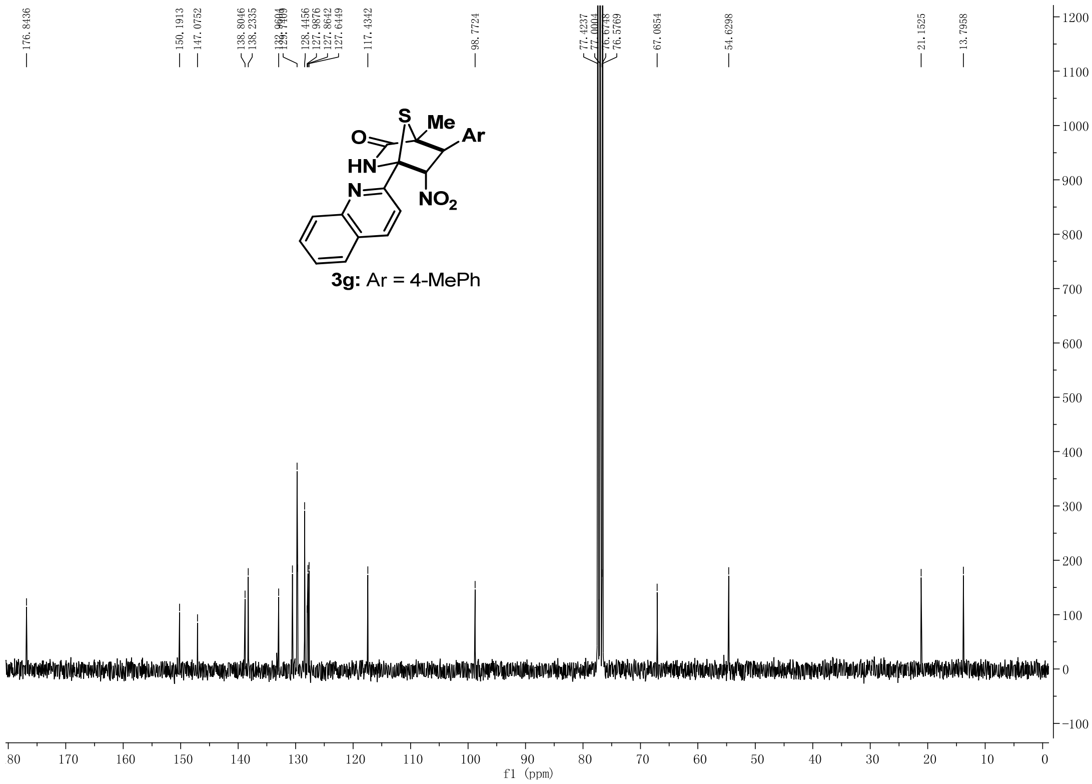
3g: Ar = 4-MePh
f1 (ppm)

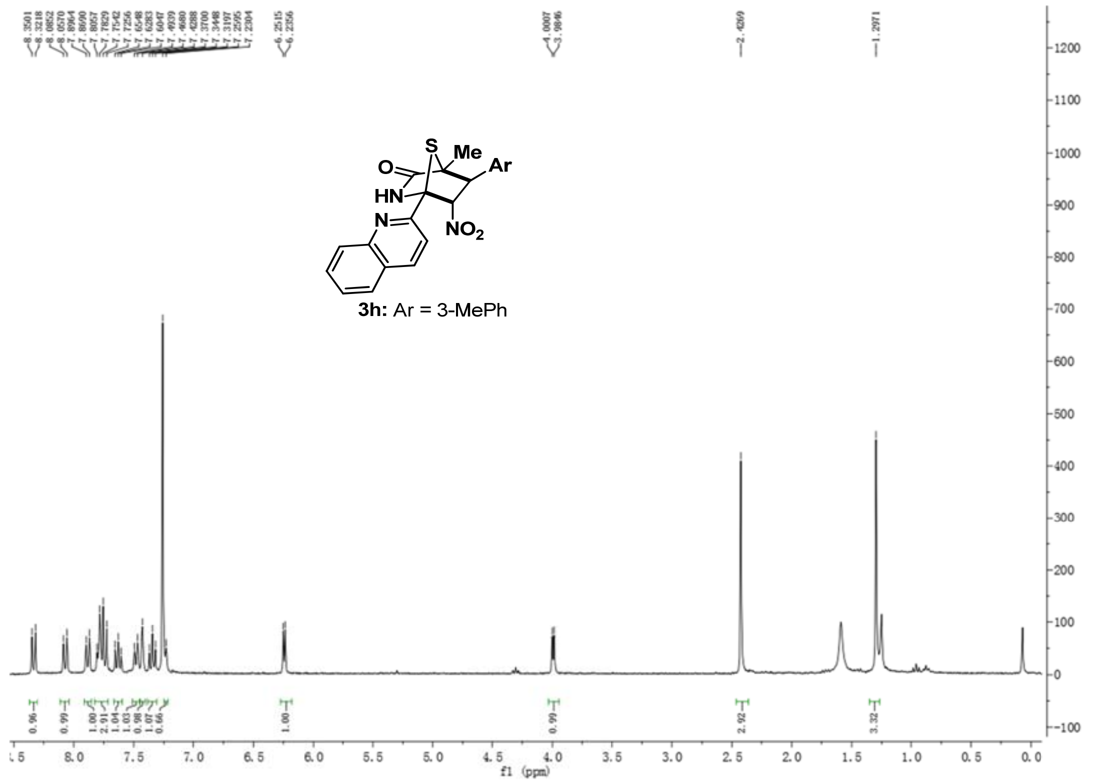
3h: Ar = 3-MePh
f1 (ppm)

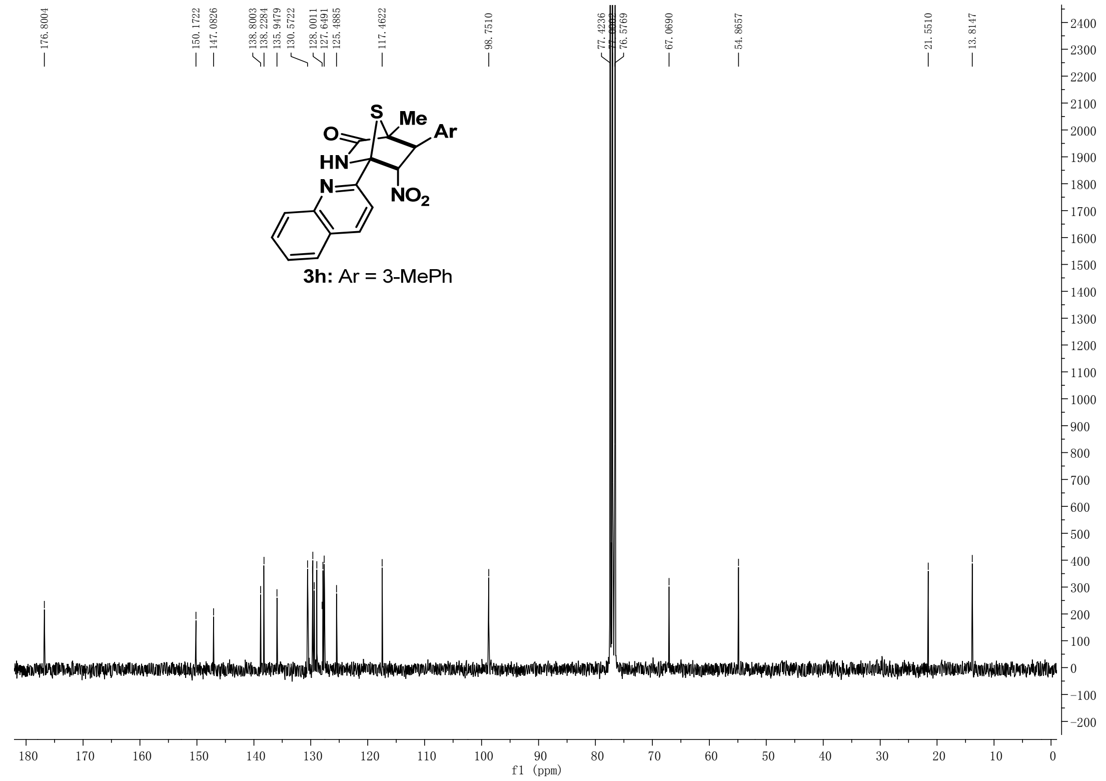
3h: Ar = 3-MePh
f1 (ppm)

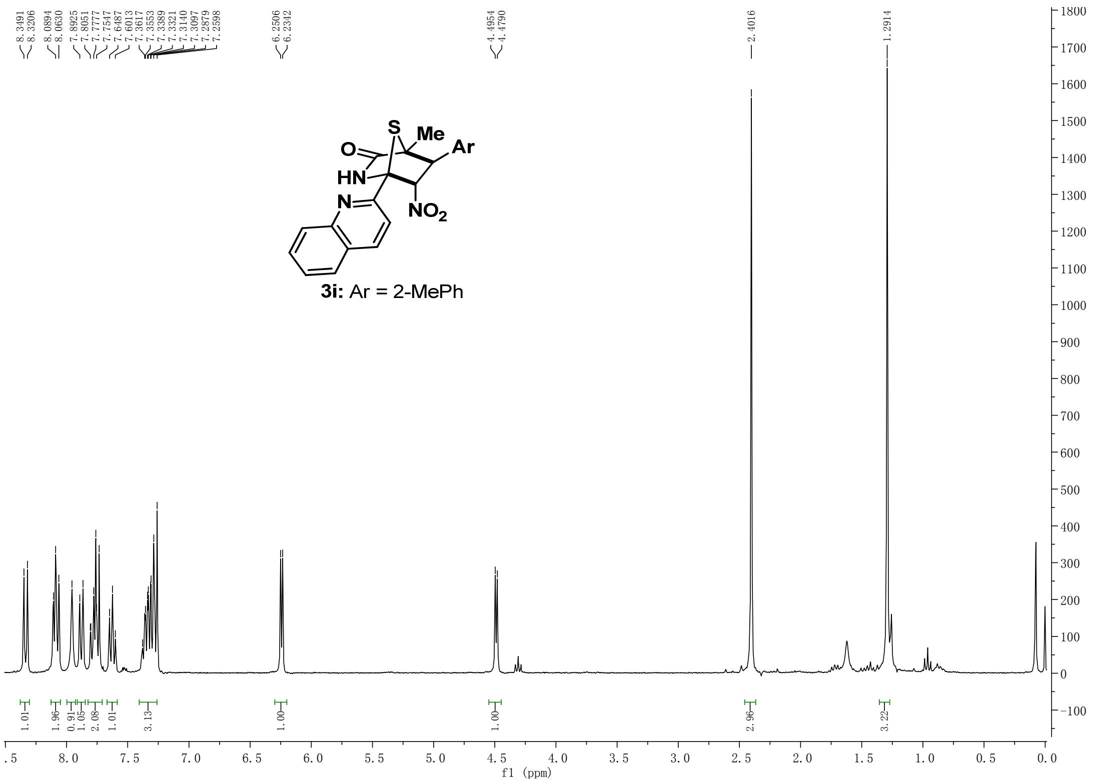
3i: Ar = 2-MePh
f1 (ppm)

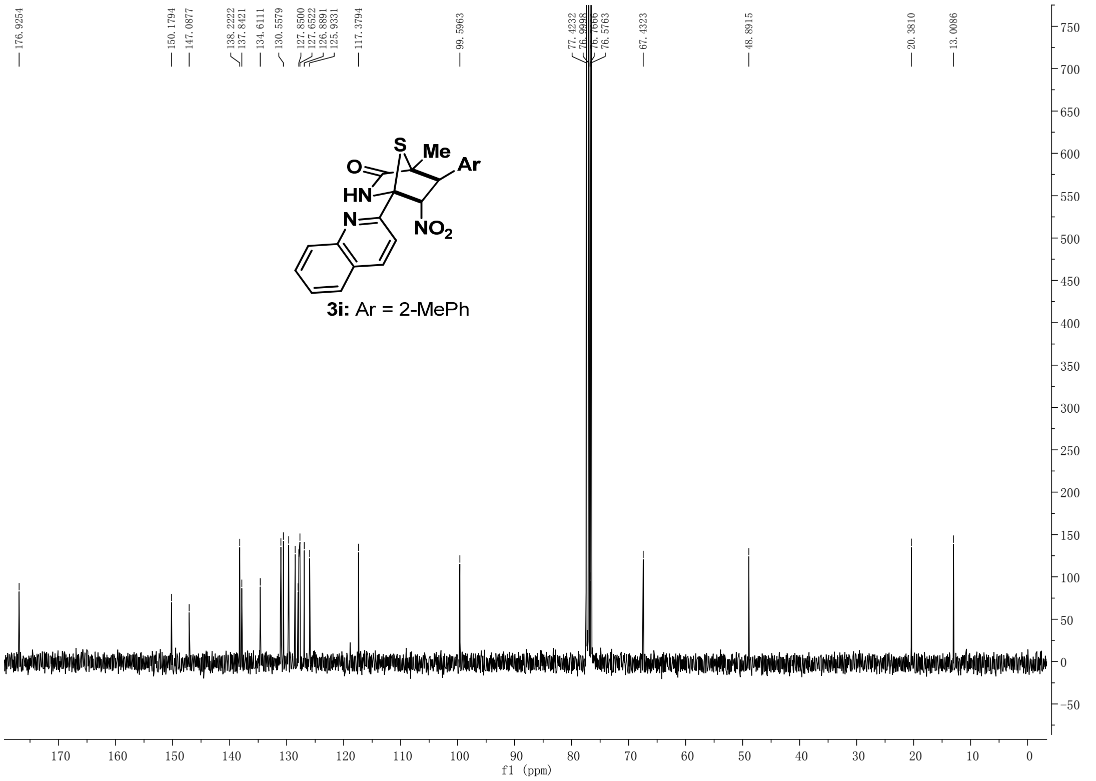
3i: Ar = 2-MePh
f1 (ppm)

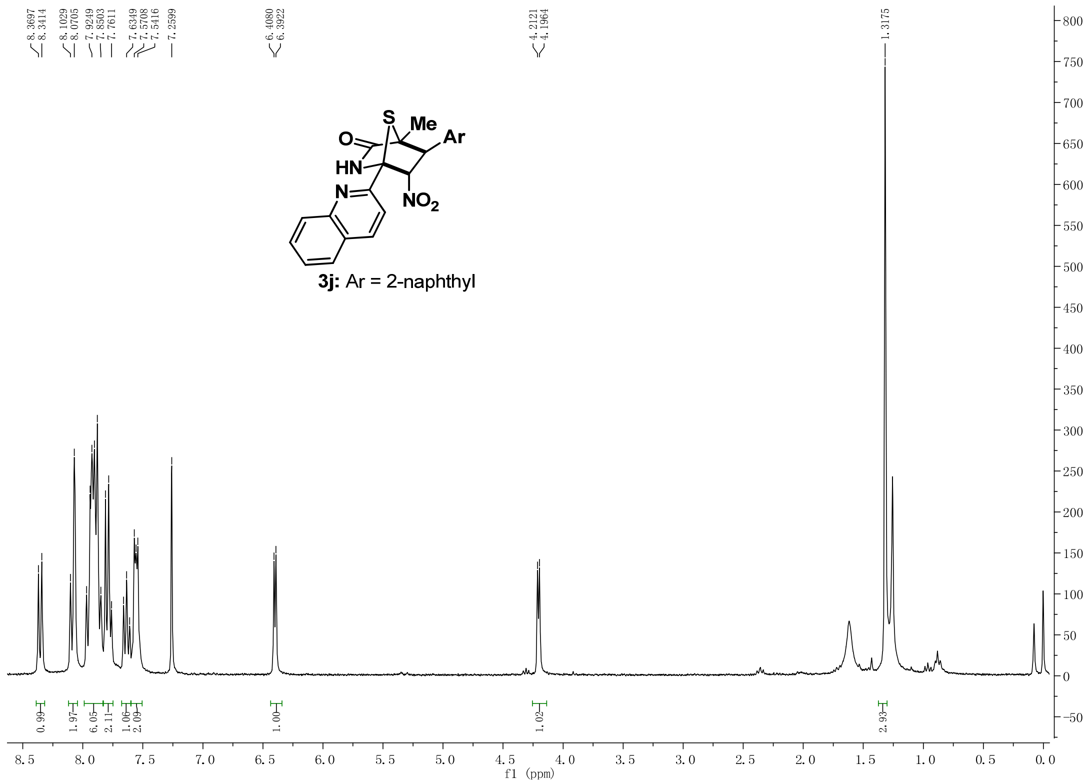
8.3697
8.3414
8.1029
8.0705
7.9249
7.8503
7.7611
7.6349
7.5708
7.5416
7.2599
6.4080
6.3922
4.2121
4.1964
1.3175
S
Me
Ar
O
HN
N
NO2
3j: Ar = 2-naphthyl
0.99
1.97
6.05
2.11
1.06
2.09
1.00
1.02
2.93
f1 (ppm)

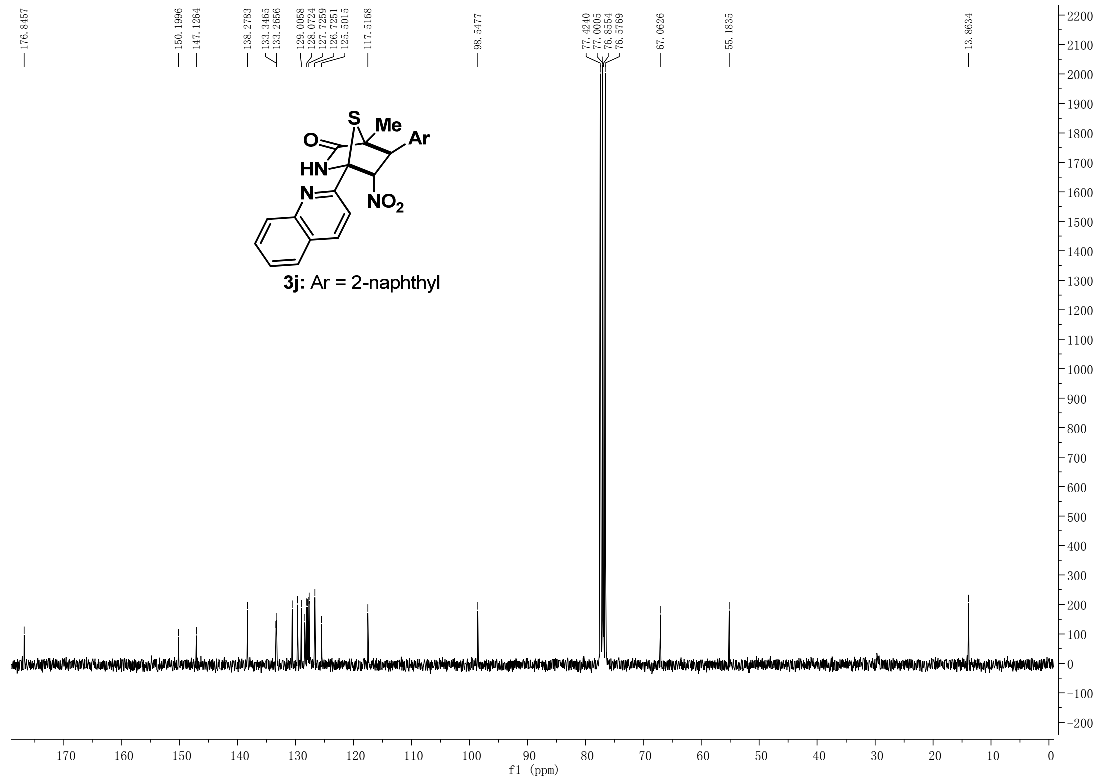
176.8457
150.1996
147.1264
138.2783
133.3465
133.2656
129.0058
128.0724
127.7259
126.7251
125.5015
117.5168
98.5477
77.4240
77.0005
76.8554
76.5769
67.0626
55.1835
13.8634
S
Me
Ar
O
HN
N
NO2
3j: Ar = 2-naphthyl
f1 (ppm)

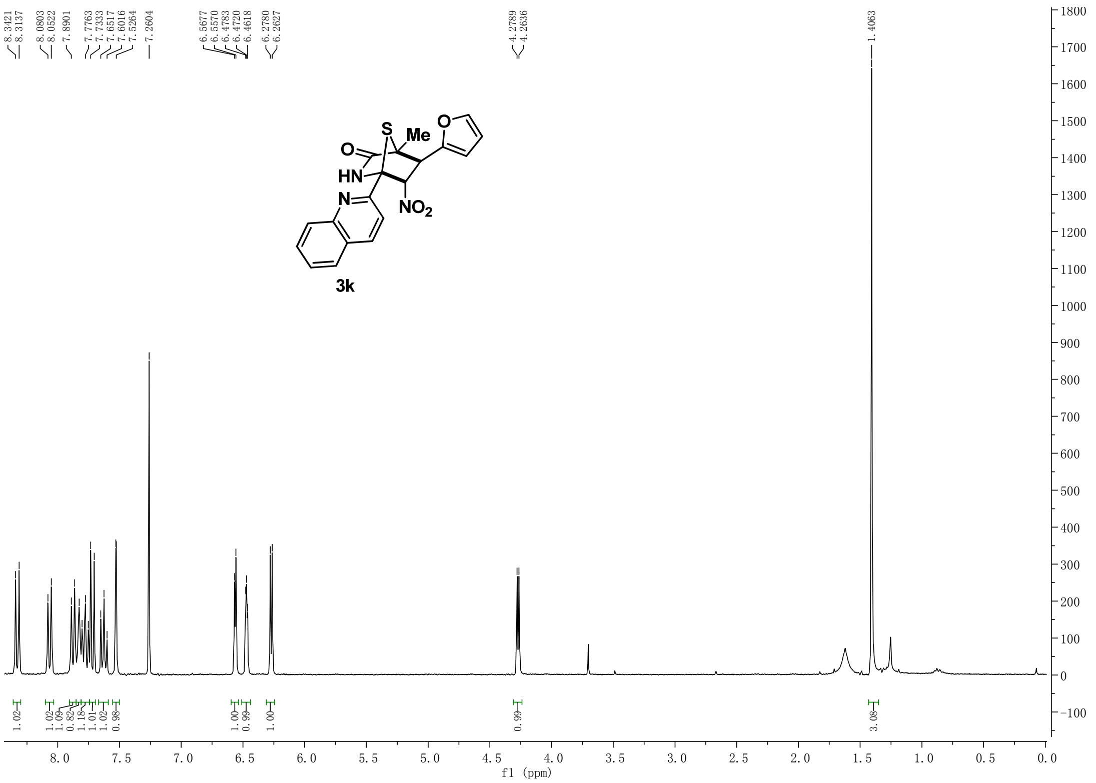
8.3421
8.3137
8.0803
8.0522
7.8901
7.7763
7.7333
7.6517
7.6016
7.5264
7.2604
6.5677
6.5570
6.4783
6.4720
6.4618
6.2780
6.2627
4.2789
4.2636
1.4063
S
Me
O
HN
N
NO_2
3k
1.02
1.02
1.09
0.82
1.18
1.01
1.02
0.98
1.00
0.99
1.00
0.99
3.08
f1 (ppm)

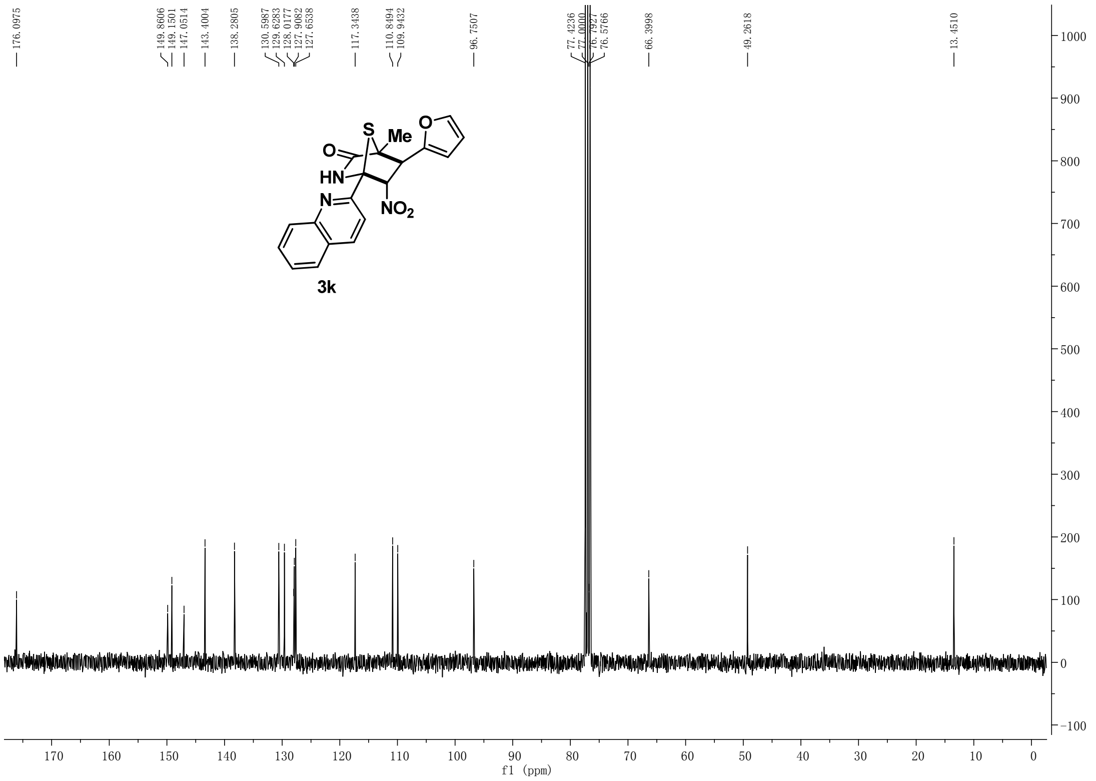
176.0975
149.8606
149.1501
147.0514
143.4004
138.2805
130.5987
129.6283
128.0177
127.9082
127.6538
117.3438
110.8494
109.9432
96.7507
77.4236
77.0000
76.7927
76.5766
66.3998
49.2618
13.4510
S
Me
O
HN
N
NO_2
3k
f1 (ppm)

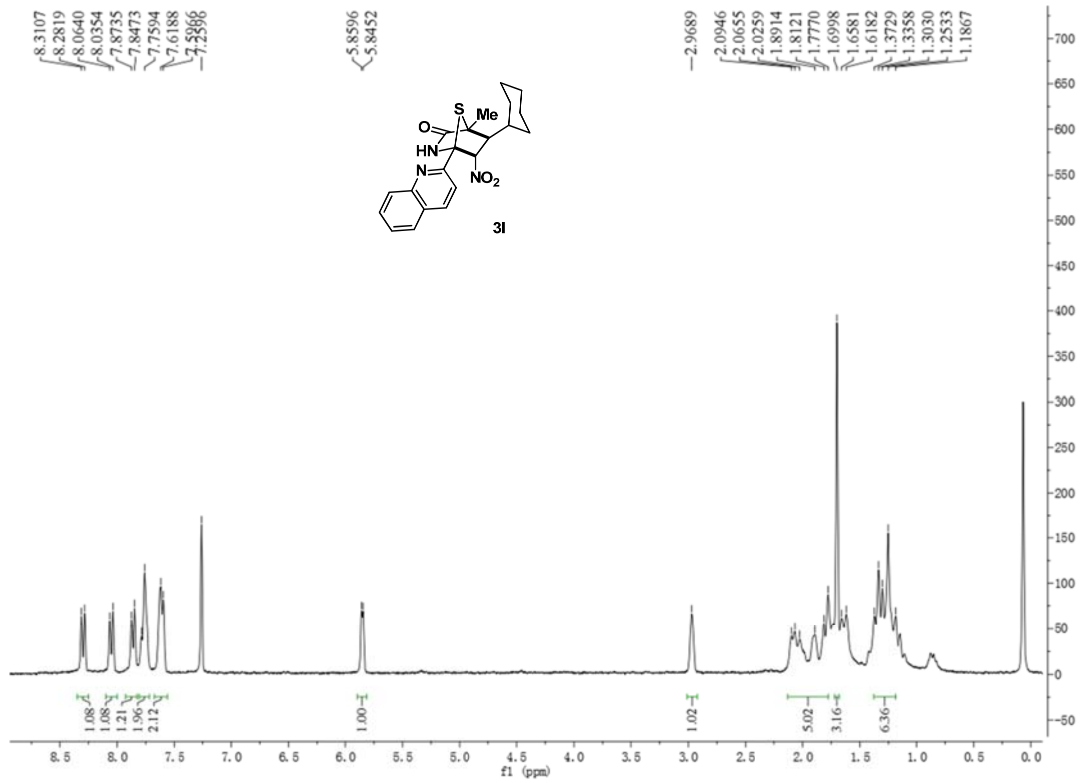

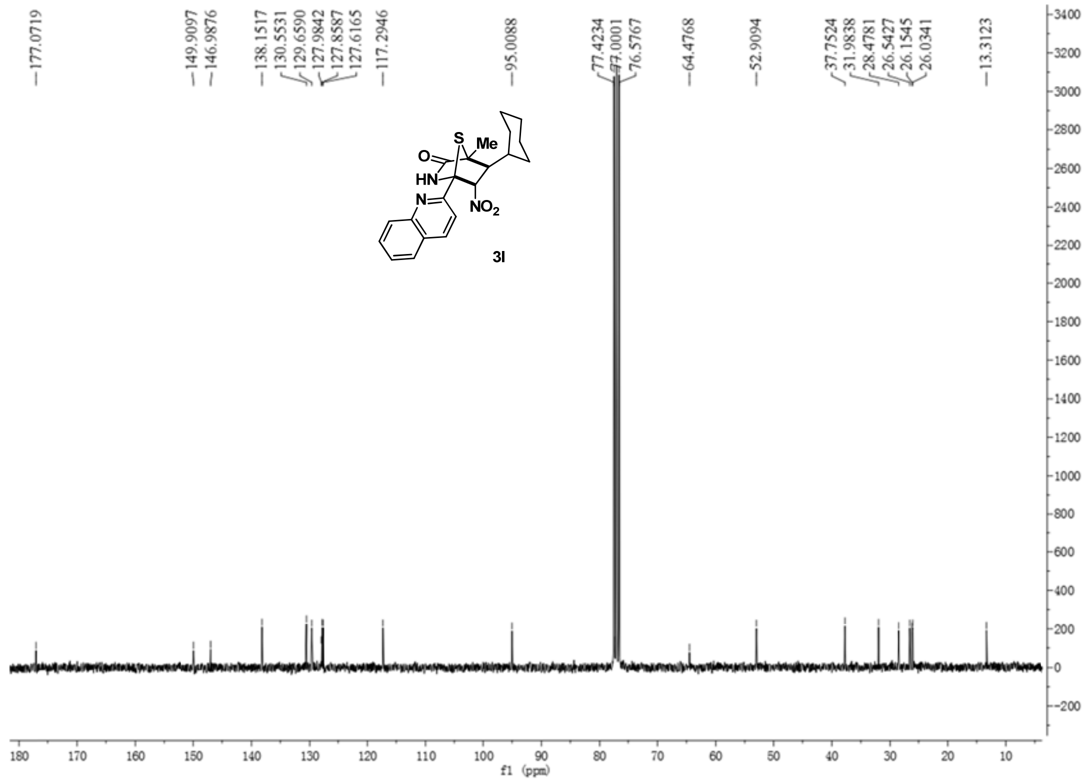

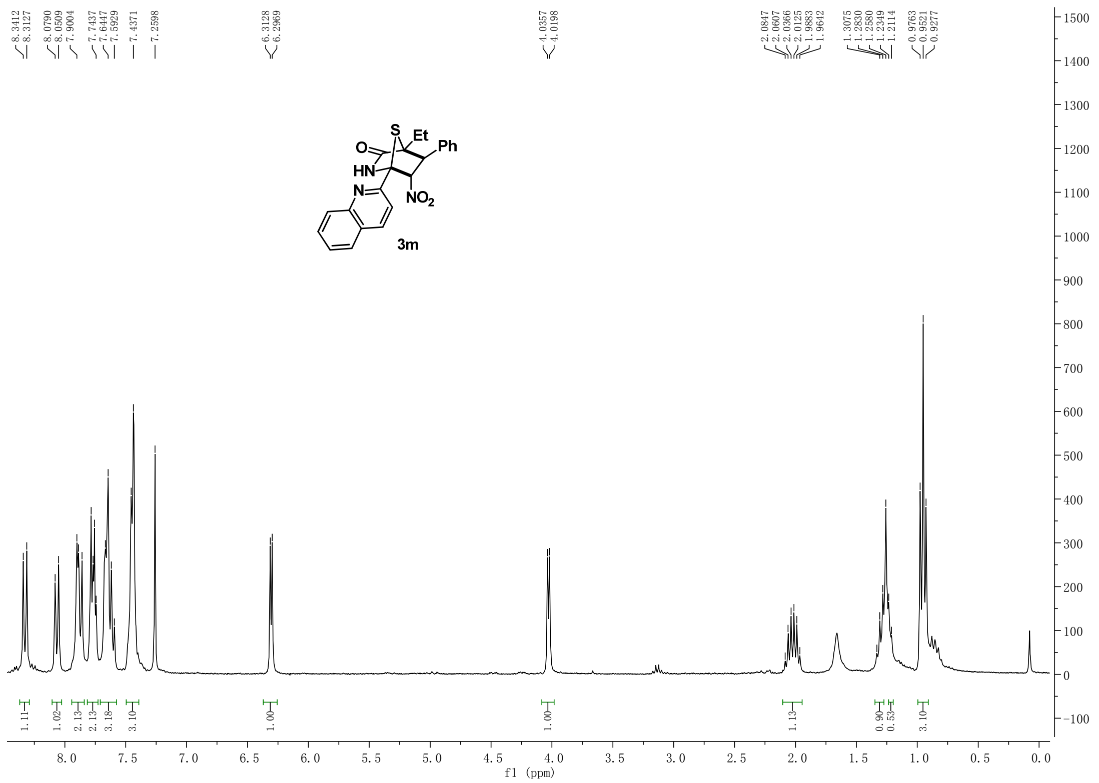

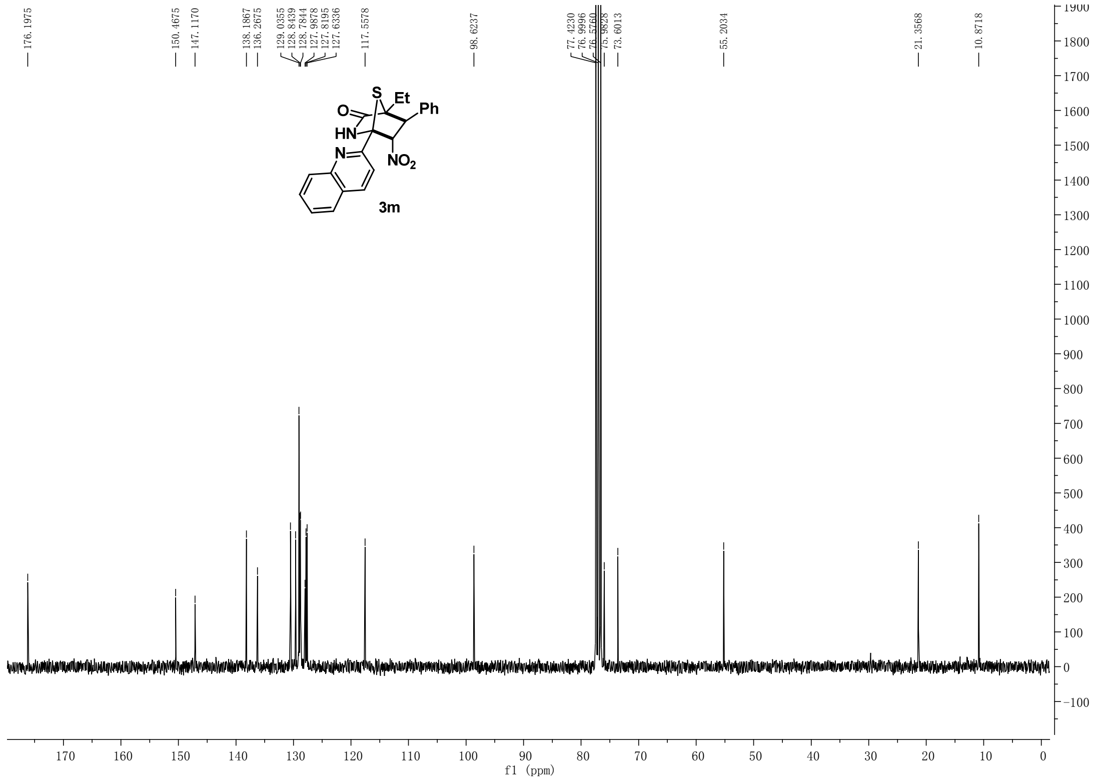

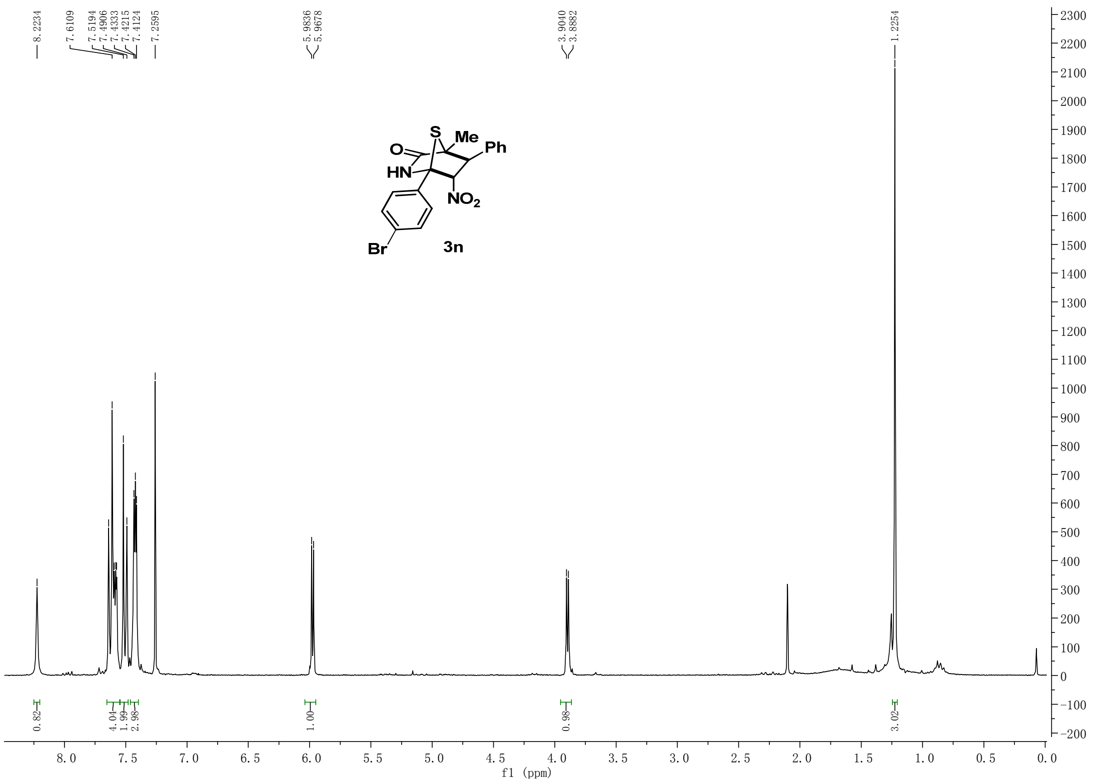
8.2234
7.6109
7.5194
7.4906
7.4333
7.4215
7.4124
7.2595
5.9836
5.9678
3.9040
3.8882
1.2254
S
Me
Ph
O
HN
NO2
Br
3n
0.82
4.04
1.99
2.98
1.00
0.98
3.02
f1 (ppm)

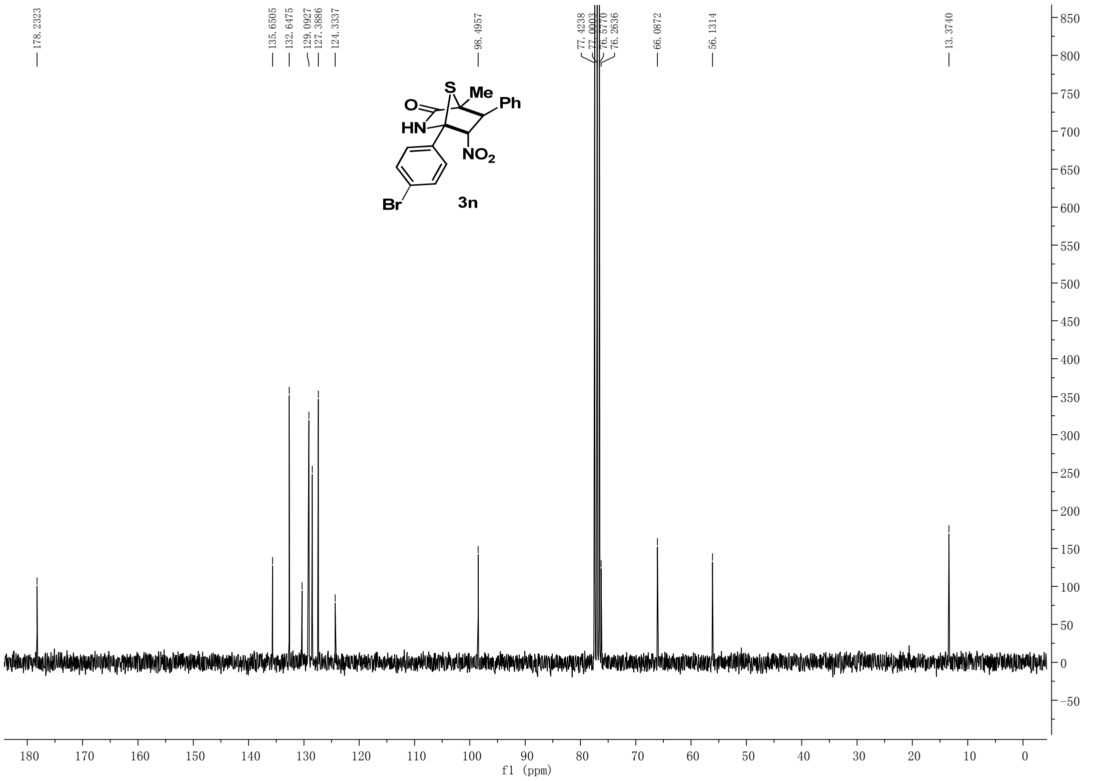
178.2323
135.6505
132.6475
129.0927
127.3886
124.3337
98.4957
77.4238
77.0003
76.5710
76.2636
66.0872
56.1314
13.3740
S
Me
Ph
O
HN
NO2
Br
3n
f1 (ppm)

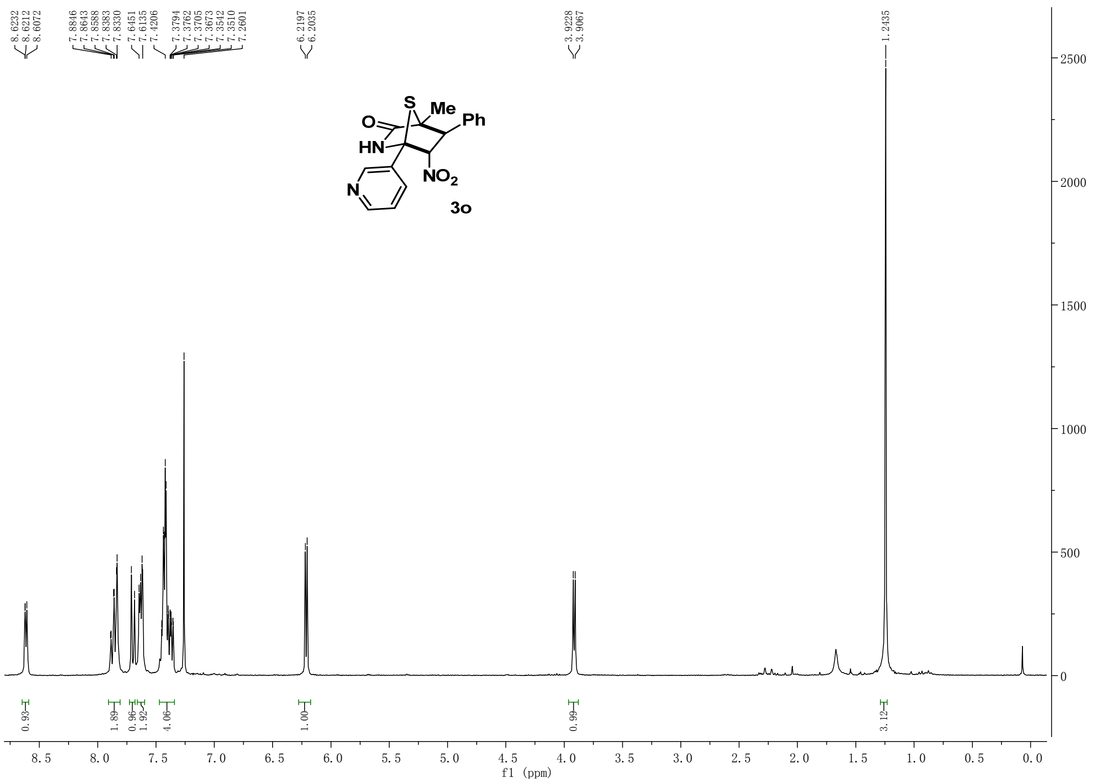
8.6232
8.6212
8.6072
7.8846
7.8643
7.8588
7.8383
7.8330
7.6451
7.6135
7.4206
7.3794
7.3762
7.3705
7.3673
7.3542
7.3510
7.2601
6.2197
6.2035
3.9228
3.9067
1.2435
S
Me
Ph
O
HN
N
NO2
3o
0.93
1.89
0.96
1.92
4.06
1.00
0.99
3.12
f1 (ppm)

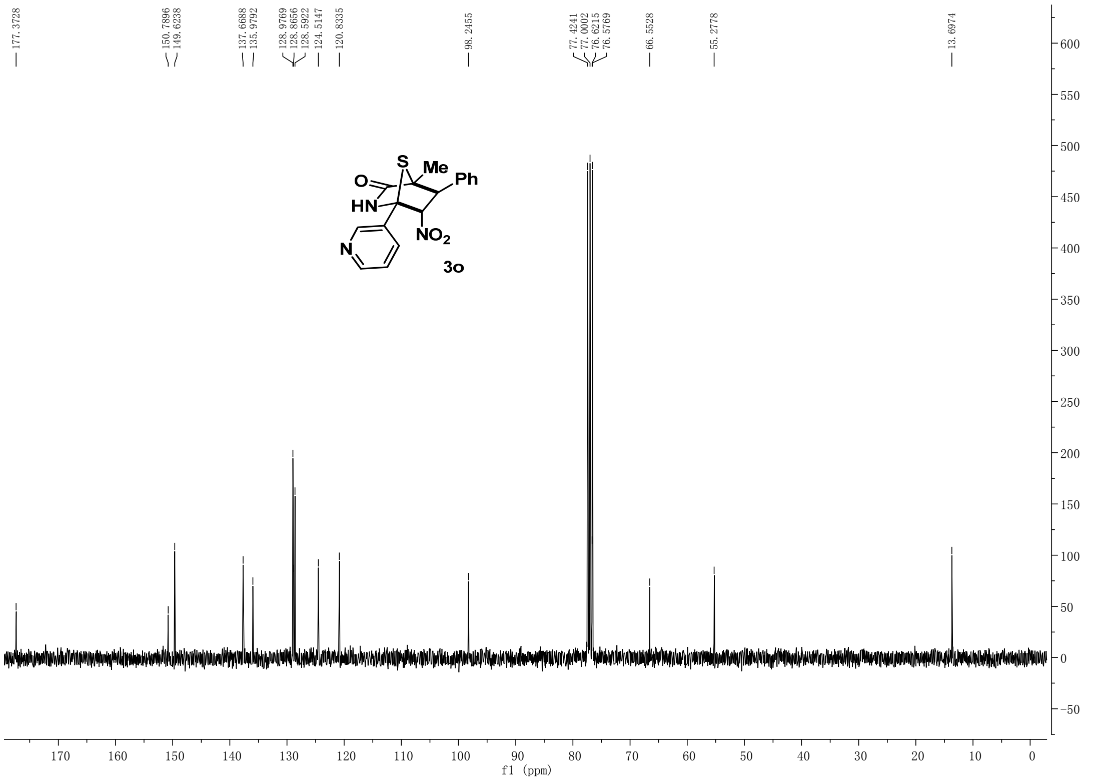
177.3728
150.7896
149.6238
137.6688
135.9792
128.9769
128.8656
128.5922
124.5147
120.8335
98.2455
77.4241
77.0002
76.6215
76.5769
66.5528
55.2778
13.6974
S
Me
Ph
O
HN
N
NO2
3o
f1 (ppm)

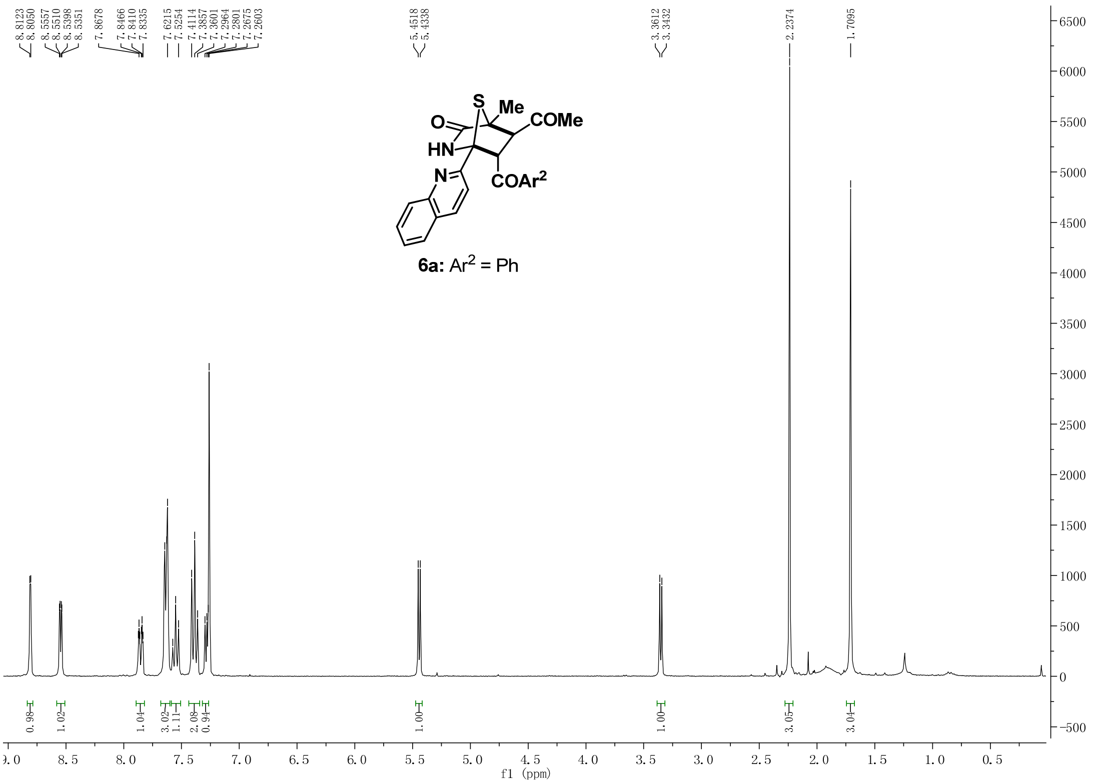

8.8123
8.8050
8.5557
8.5510
8.5398
8.5351
7.8678
7.8466
7.8410
7.8335
7.6215
7.5254
7.4114
7.3857
7.3601
7.2964
7.2891
7.2675
7.2603
5.4518
5.4338
3.3612
3.3432
2.2374
1.7095
S
Me
O
COMe
HN
N
COAr2
6a: Ar2 = Ph
0.98
1.02
1.04
3.02
1.11
2.08
0.94
1.00
1.00
3.05
3.04
f1 (ppm)

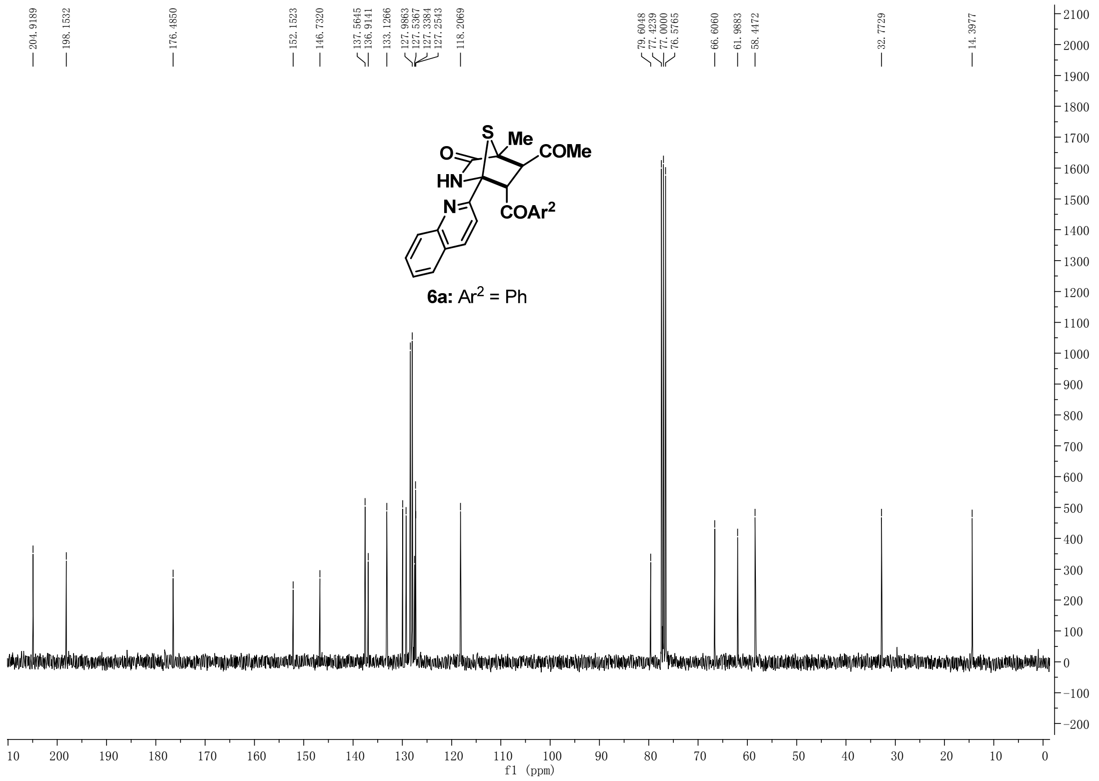

204.9189
198.1532
176.4850
152.1523
146.7320
137.5645
136.9141
133.1266
127.9863
127.5367
127.3384
127.2543
118.2069
79.6048
77.4239
77.0000
76.5765
66.6060
61.9883
58.4472
32.7729
14.3977
S
Me
O
COMe
HN
N
COAr2
6a: Ar2 = Ph
f1 (ppm)

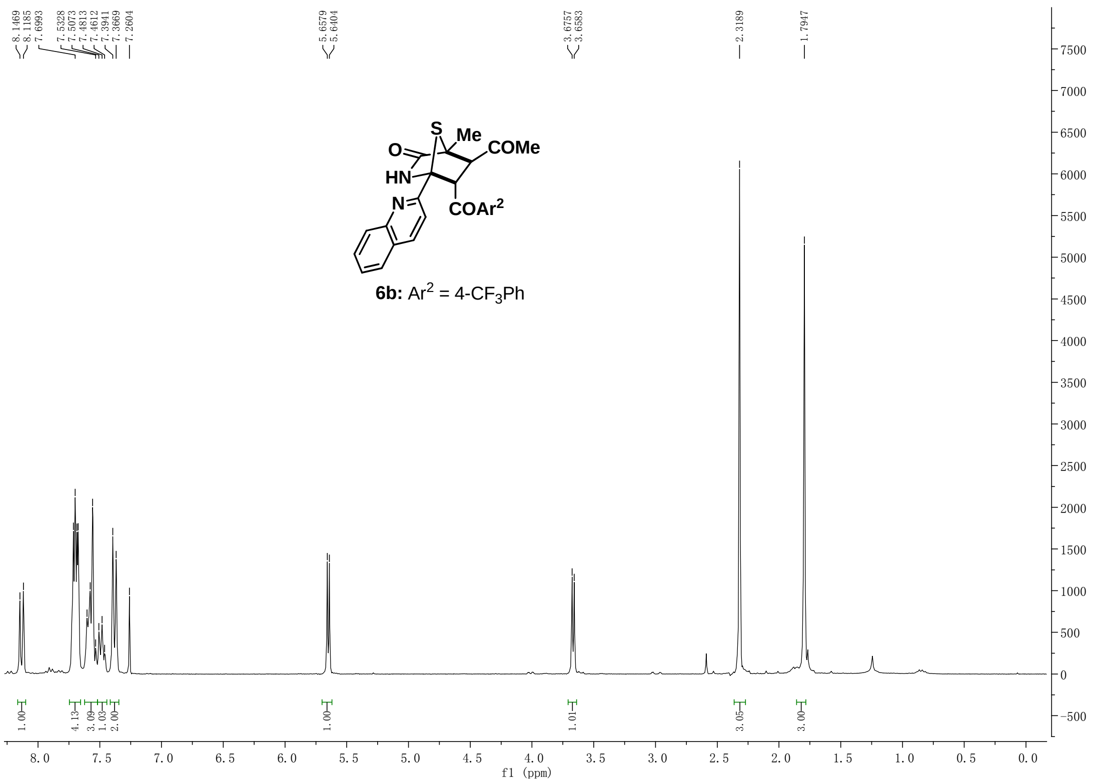
8.1469
8.1185
7.6993
7.5328
7.5073
7.4813
7.4612
7.3941
7.3669
7.2604
5.6579
5.6404
3.6757
3.6583
2.3189
1.7947
S
Me
COMe
O
HN
N
COAr2
6b: Ar2 = 4-CF3Ph
1.00
4.13
3.09
1.03
2.00
1.00
1.01
3.05
3.00
f1 (ppm)

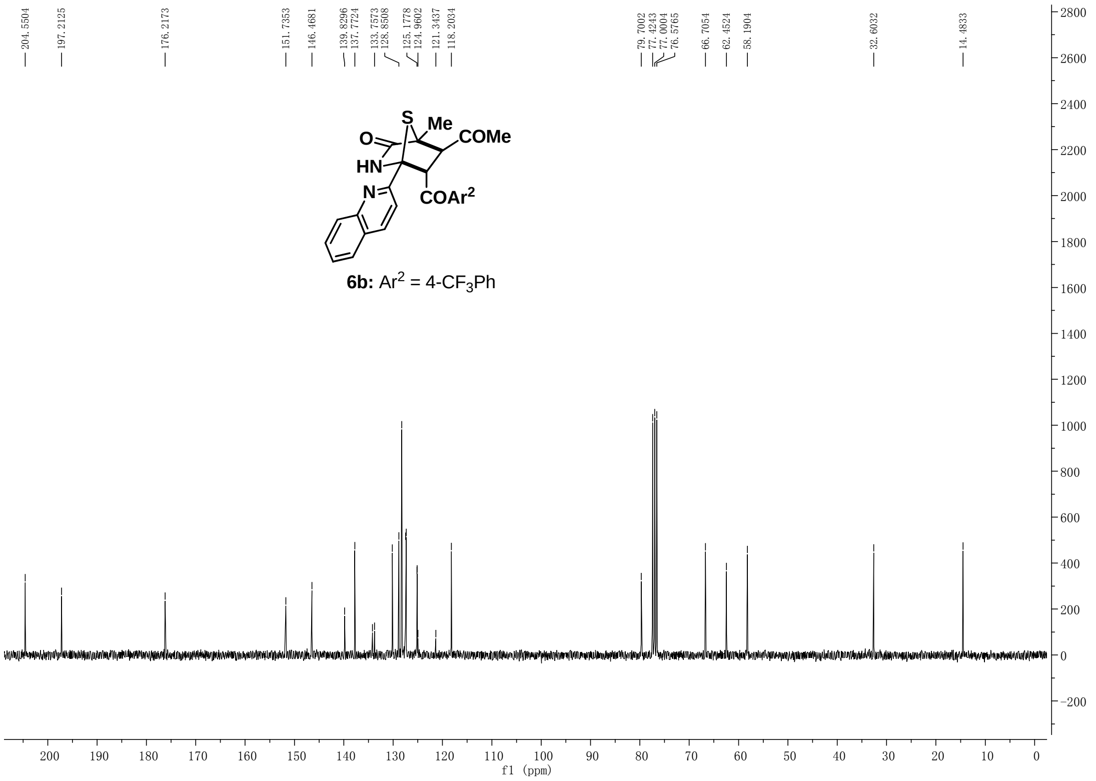
204.5504
197.2125
176.2173
151.7353
146.4681
139.8296
137.7724
133.7573
128.8508
125.1778
124.9602
121.3437
118.2034
79.7002
77.4243
77.0004
76.5765
66.7054
62.4524
58.1904
32.6032
14.4833
S
Me
COMe
O
HN
N
COAr2
6b: Ar2 = 4-CF3Ph
f1 (ppm)

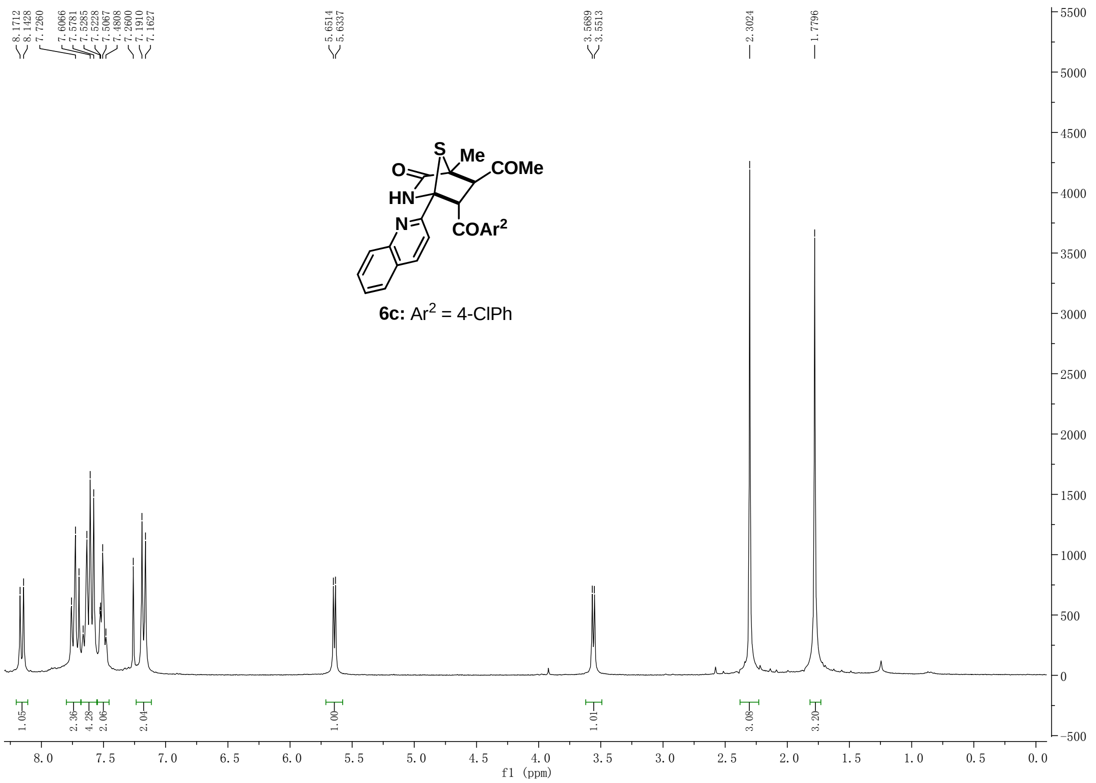
8.1712
8.1428
7.7260
7.6066
7.5781
7.5285
7.5228
7.5067
7.4808
7.2600
7.1910
7.1627
5.6514
5.6337
3.5689
3.5513
2.3024
1.7796
S
Me
O
COMe
HN
N
COAr2
6c: Ar2 = 4-ClPh
1.05
2.36
4.28
2.06
2.04
1.00
1.01
3.08
3.20
f1 (ppm)

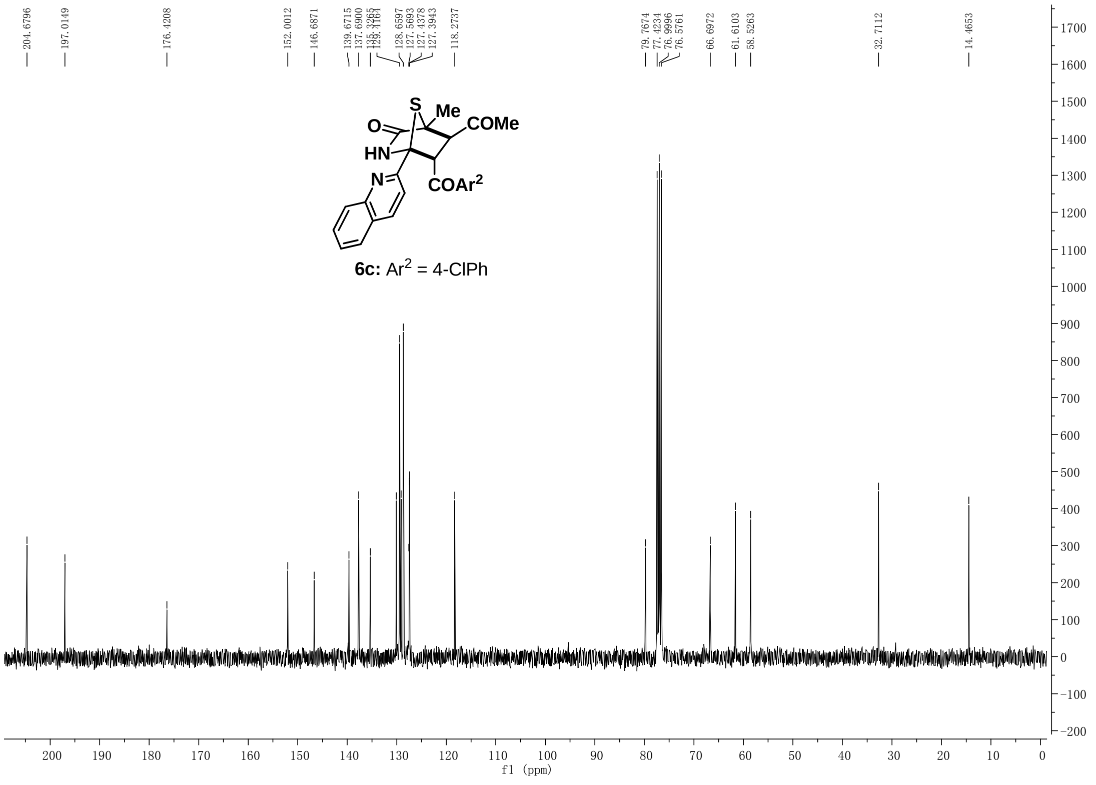
204.6796
197.0149
176.4208
152.0012
146.6871
139.6715
137.6900
128.6597
127.5693
127.4378
127.3943
118.2737
79.7674
77.4234
76.9996
76.5761
66.6972
61.6103
58.5263
32.7112
14.4653
S
Me
O
COMe
HN
N
COAr2
6c: Ar2 = 4-ClPh
f1 (ppm)

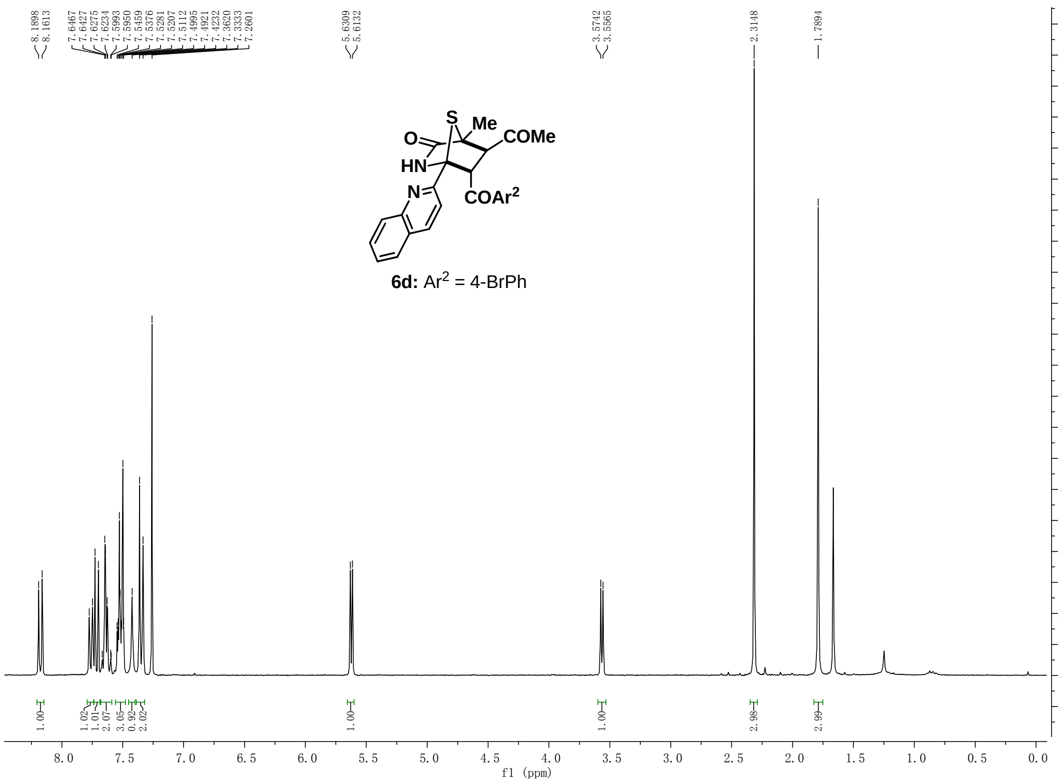
8.1898
8.1613
7.6467
7.6427
7.6275
7.6234
7.5993
7.5950
7.5459
7.5376
7.5281
7.5207
7.5112
7.4995
7.4921
7.4232
7.3620
7.3333
7.2601
5.6309
5.6132
3.5742
3.5565
2.3148
1.7894
S
Me
COMe
O
HN
N
COAr2
6d: Ar2 = 4-BrPh
1.00
1.02
1.01
2.07
3.05
0.92
2.02
1.00
1.00
2.98
2.99
f1 (ppm)

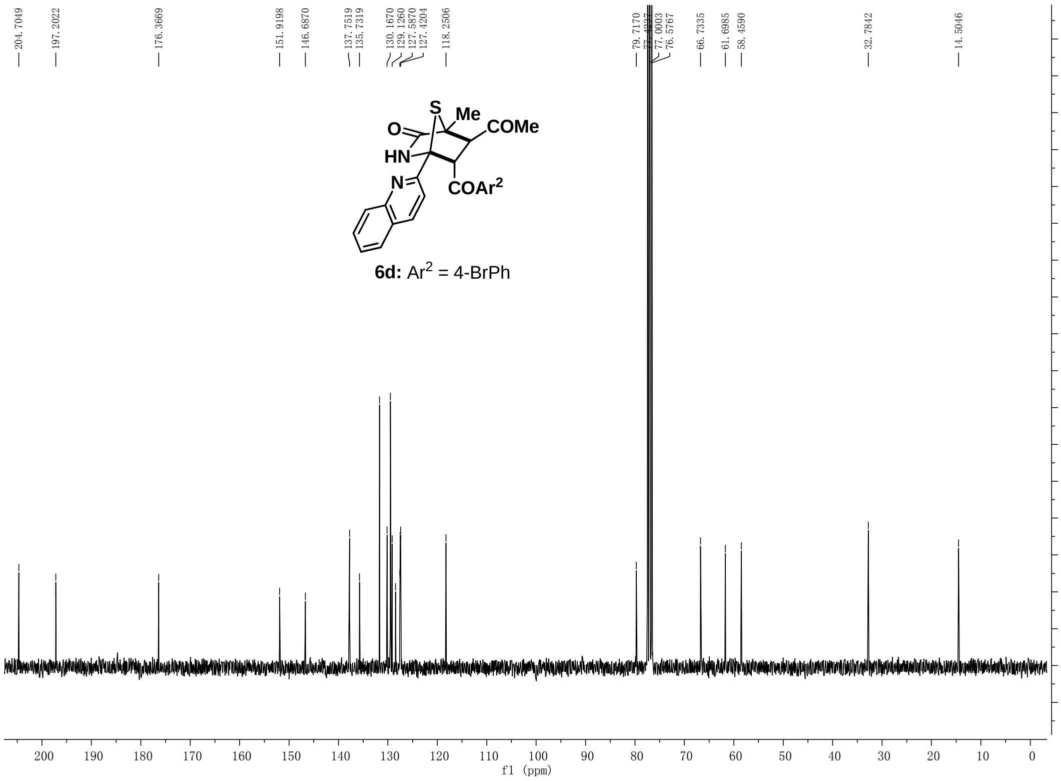
204.7049
197.2022
176.3669
151.9198
146.6870
137.7519
135.7319
130.1670
129.1260
127.5870
127.4204
118.2506
79.7170
77.0003
76.5767
66.7335
61.6985
58.4590
32.7842
14.5046
S
Me
COMe
O
HN
N
COAr2
6d: Ar2 = 4-BrPh
f1 (ppm)

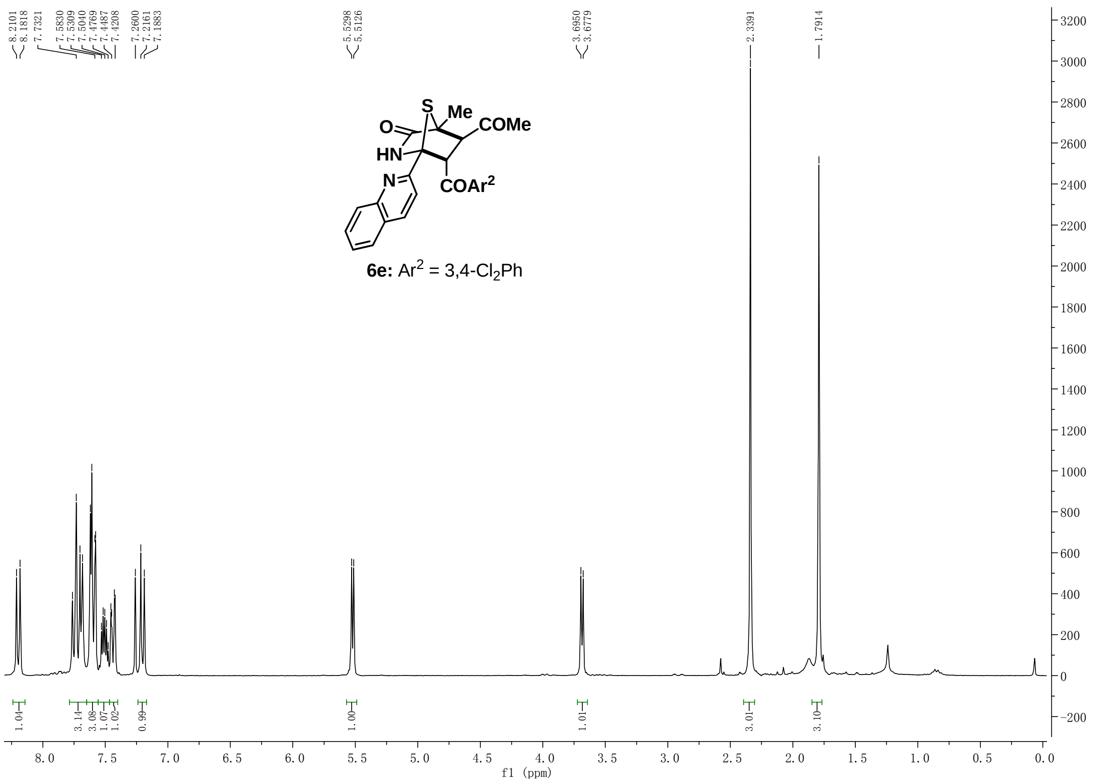
6e: Ar2 = 3,4-Cl2Ph
f1 (ppm)

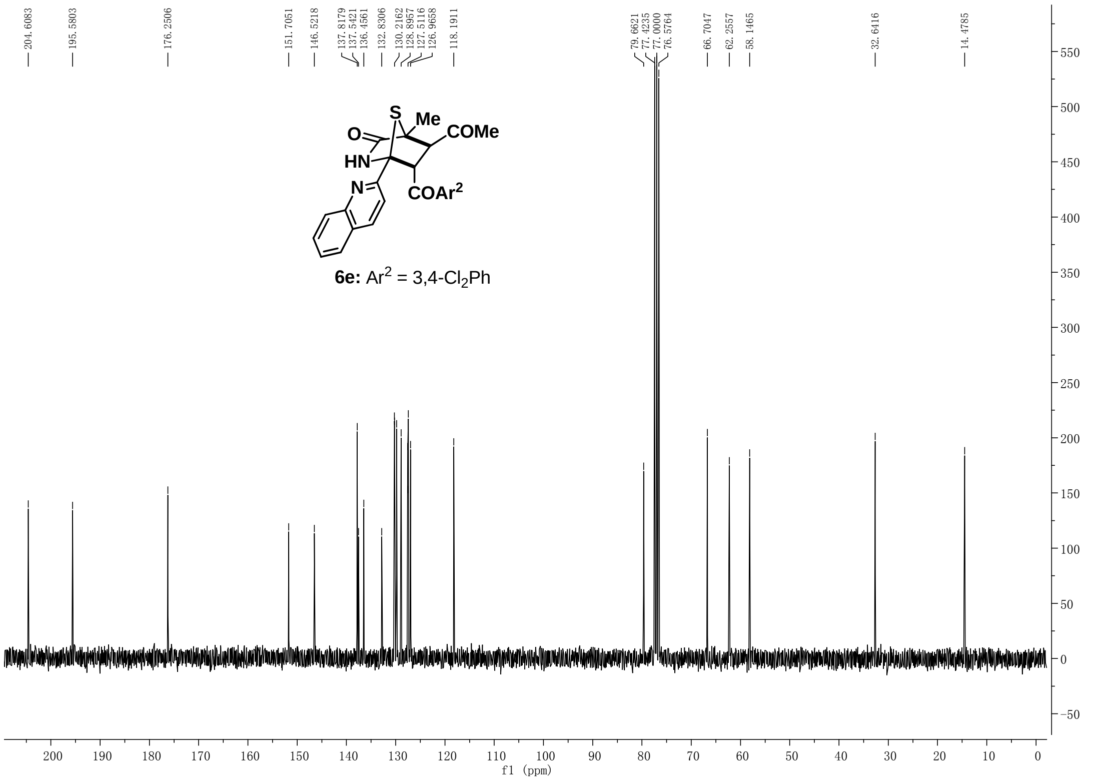
6e: Ar2 = 3,4-Cl2Ph
f1 (ppm)

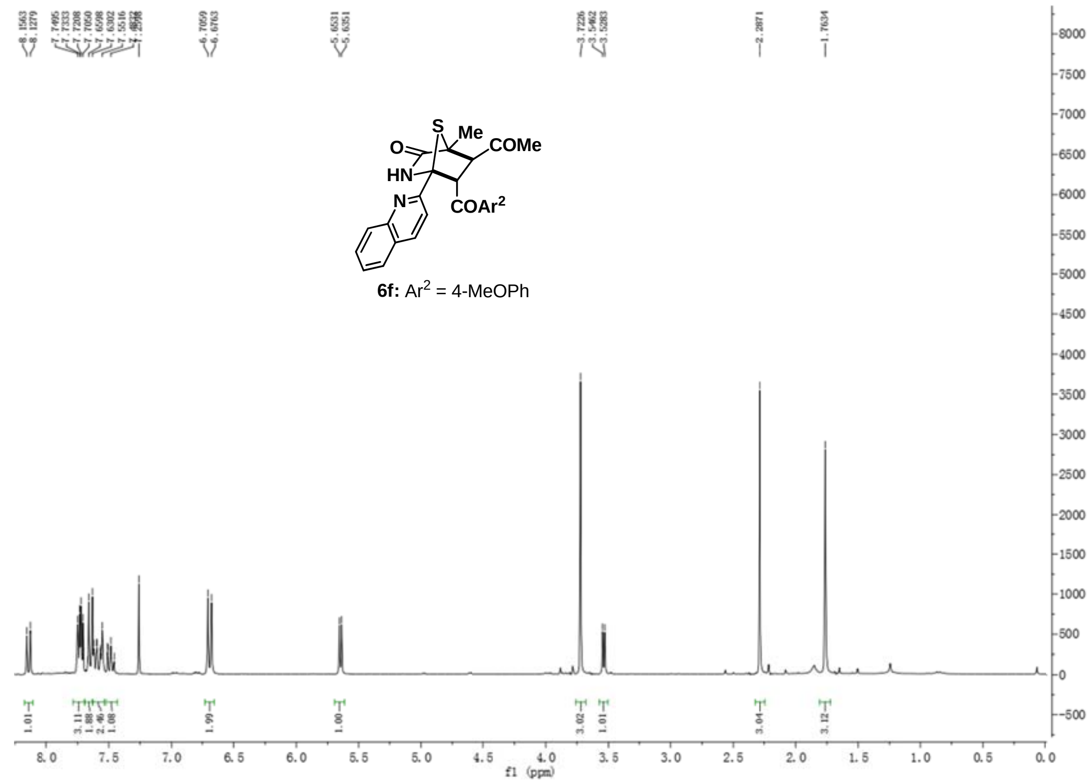
6f: Ar2 = 4-MeOPh
f1 (ppm)

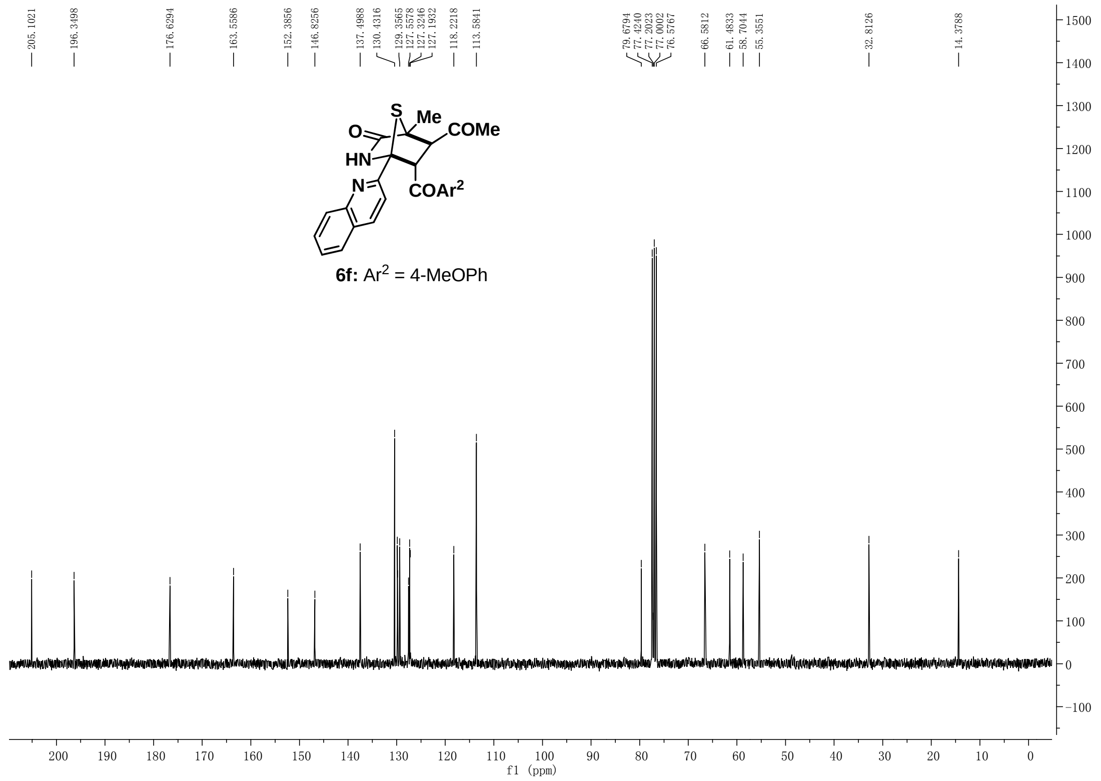
6f: Ar2 = 4-MeOPh
f1 (ppm)

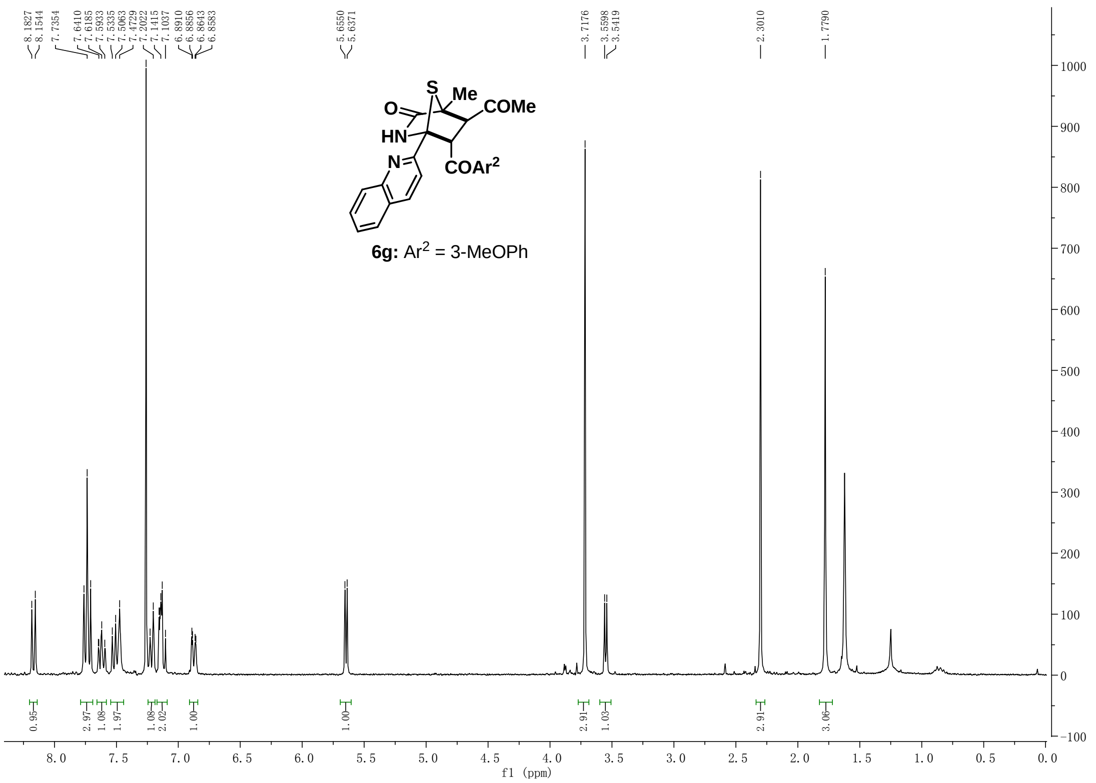

6g: Ar2 = 3-MeOPh
f1 (ppm)

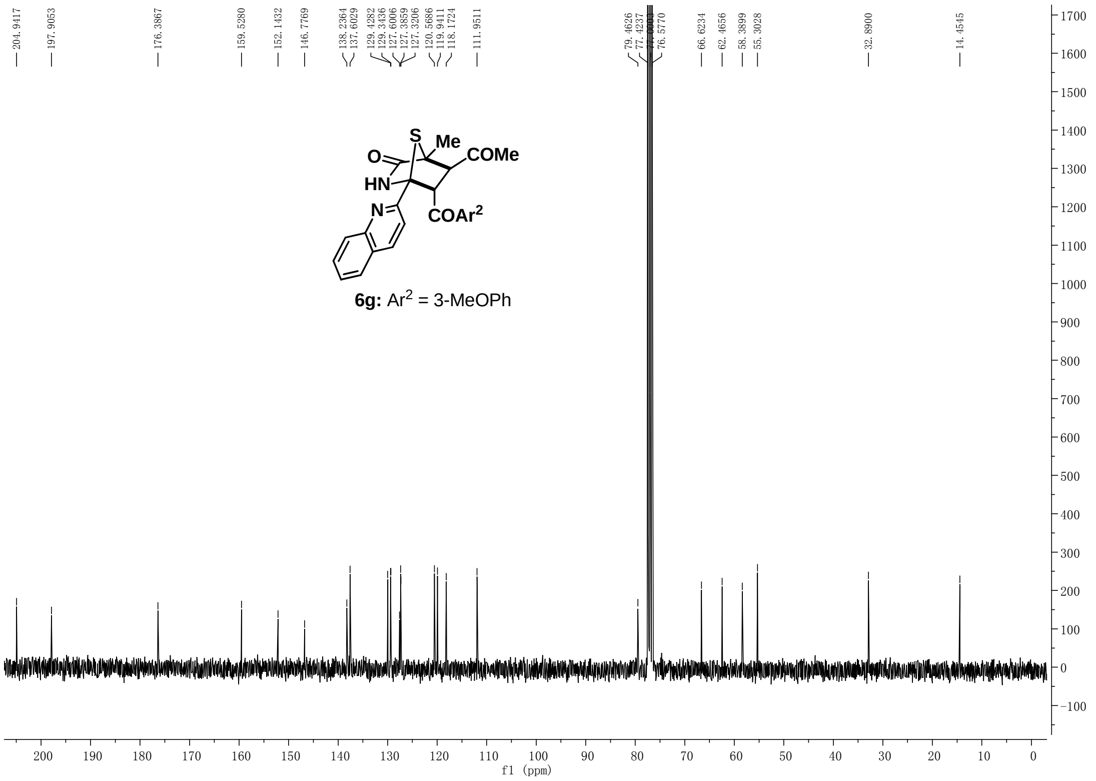

6g: Ar2 = 3-MeOPh
f1 (ppm)

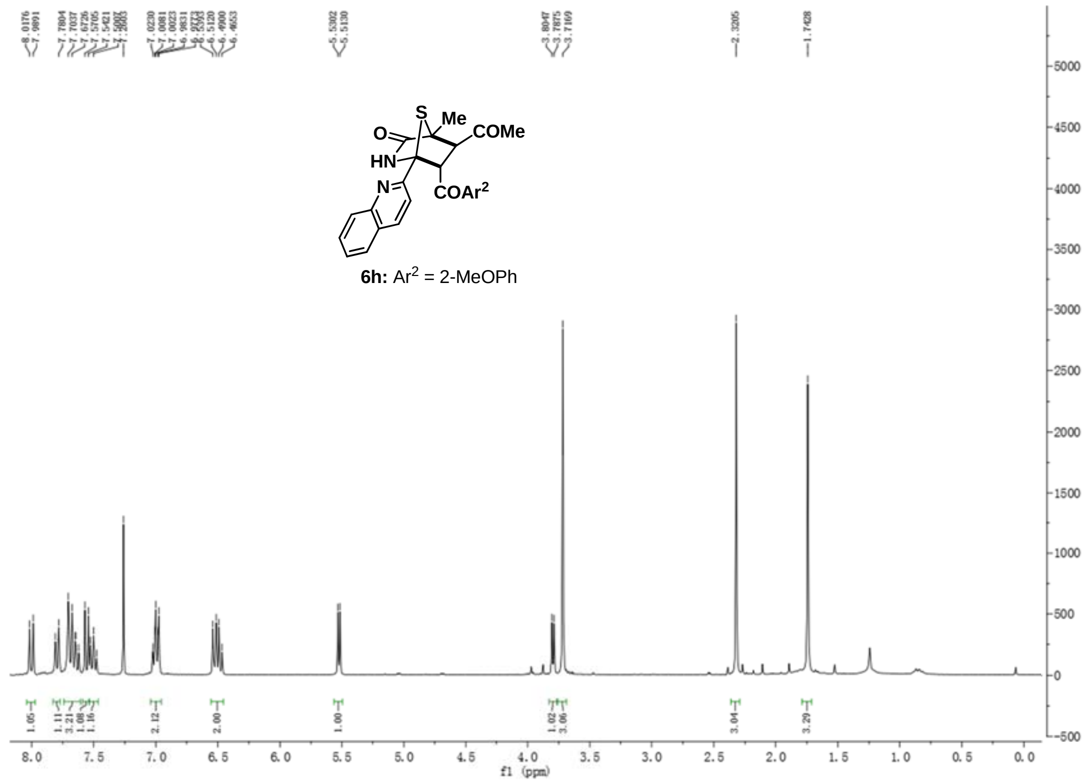
6h: Ar^2 = 2-MeOPh
f1 (ppm)

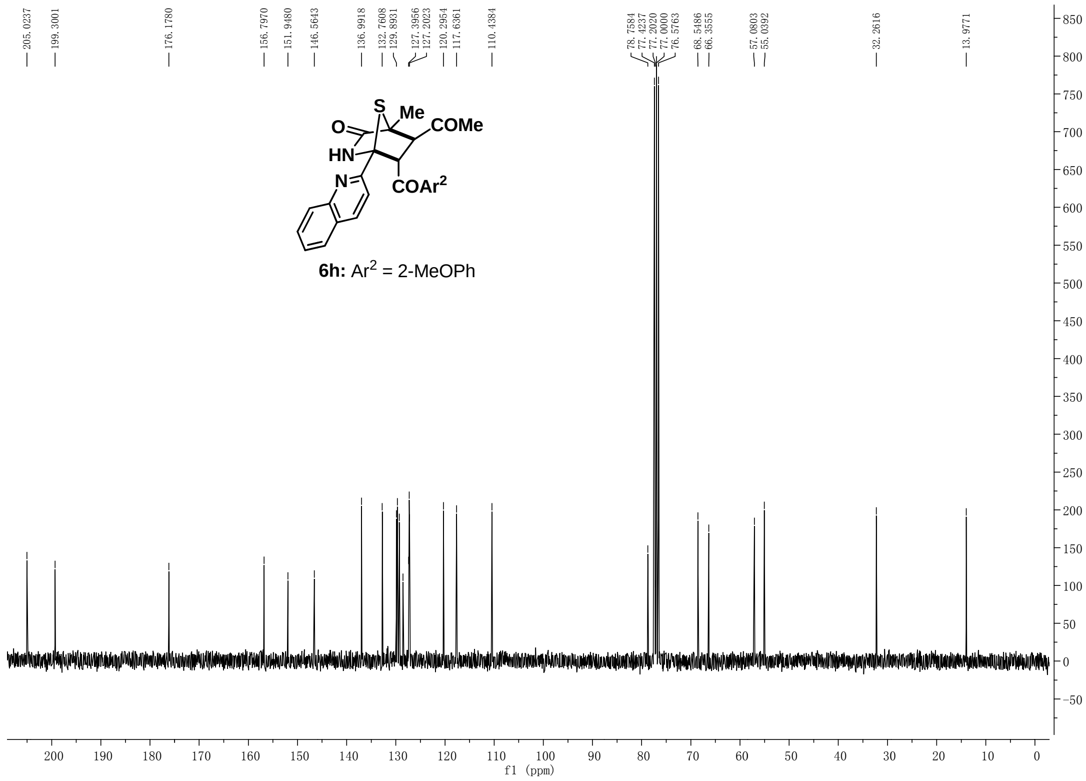
6h: Ar^2 = 2-MeOPh
f1 (ppm)

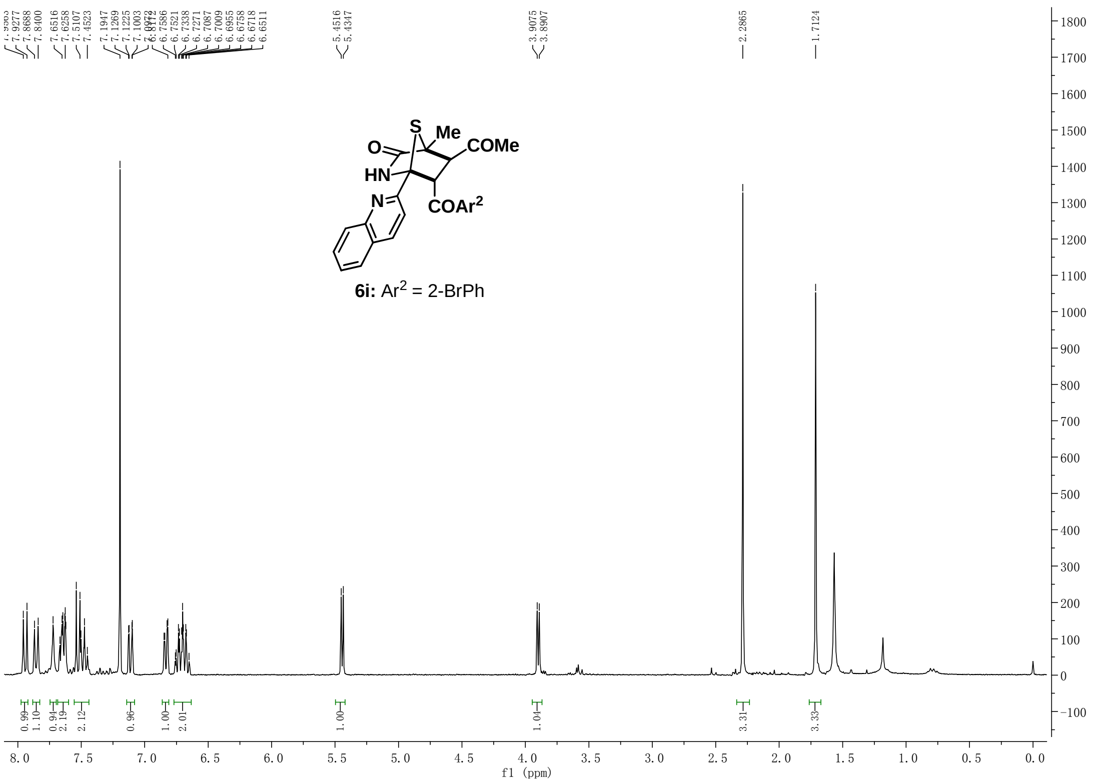

6i: Ar^2 = 2-BrPh
f1 (ppm)

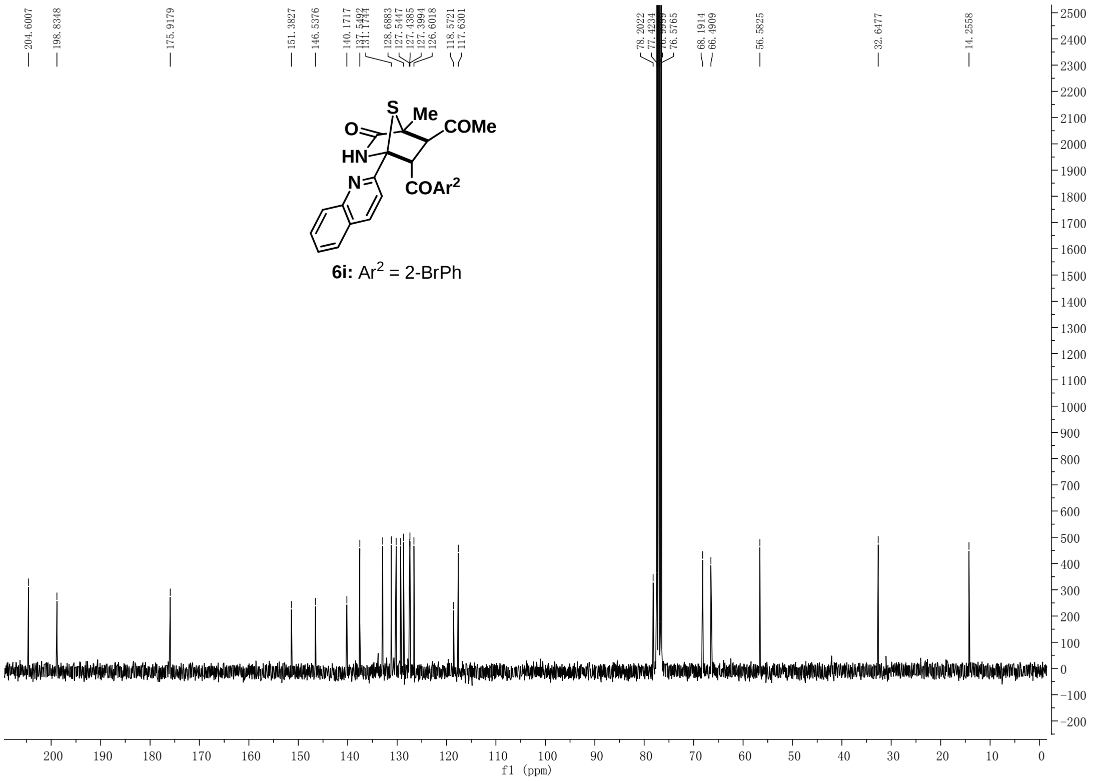

6i: Ar^2 = 2-BrPh
f1 (ppm)

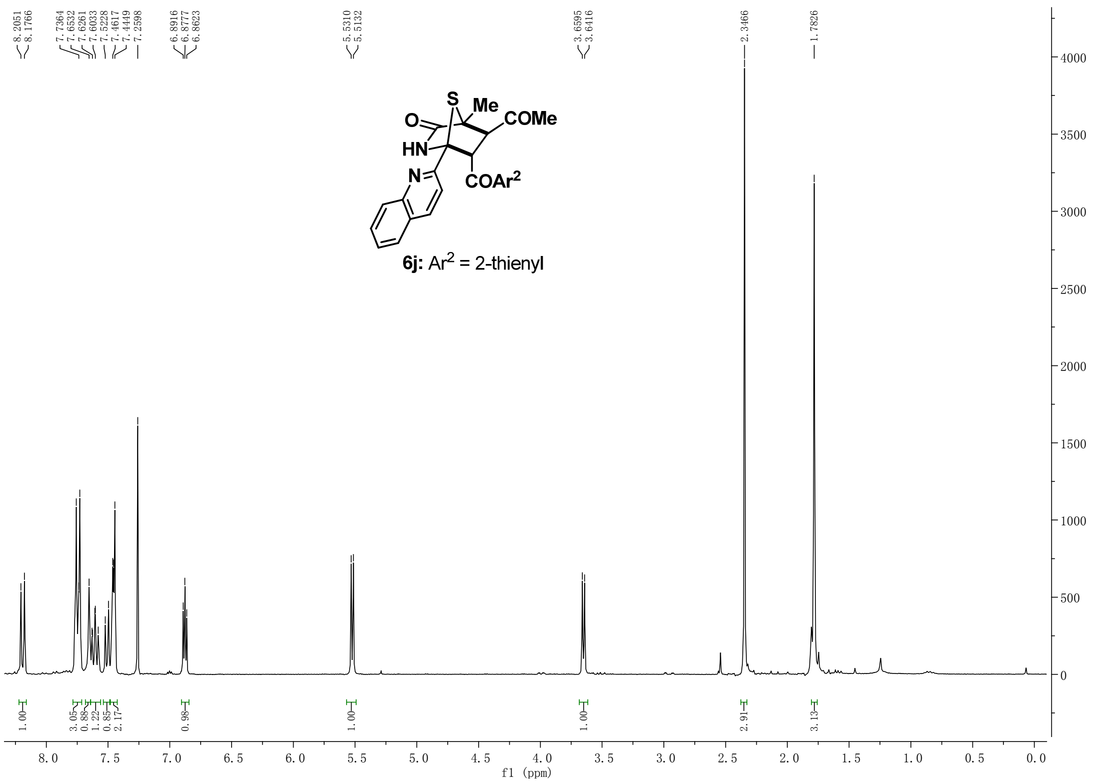
6j: Ar2 = 2-thienyl
f1 (ppm)

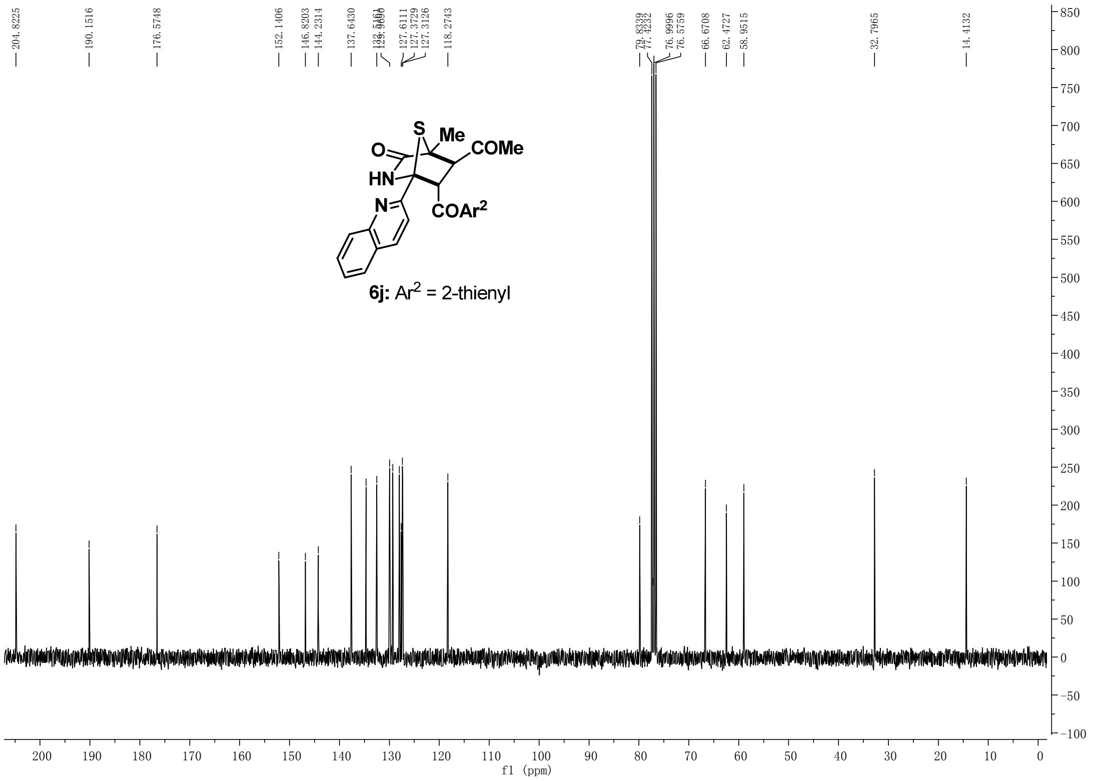
6j: Ar2 = 2-thienyl
f1 (ppm)

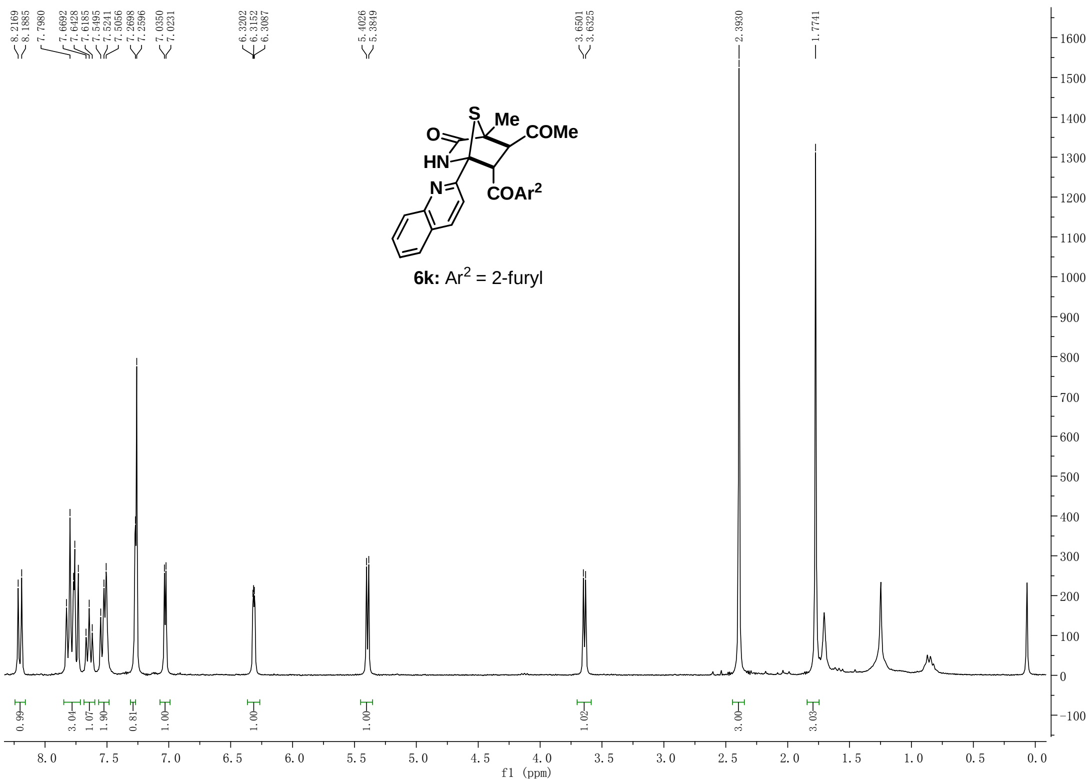
6k: Ar2 = 2-furyl
f1 (ppm)

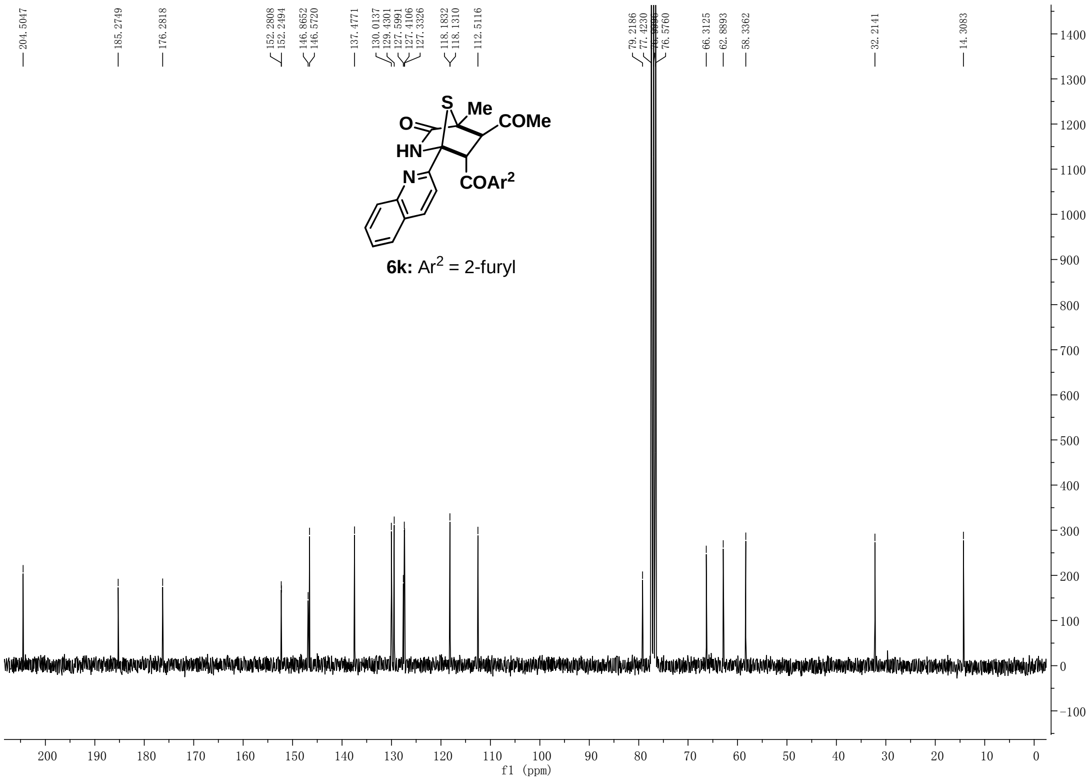
6k: Ar2 = 2-furyl
f1 (ppm)

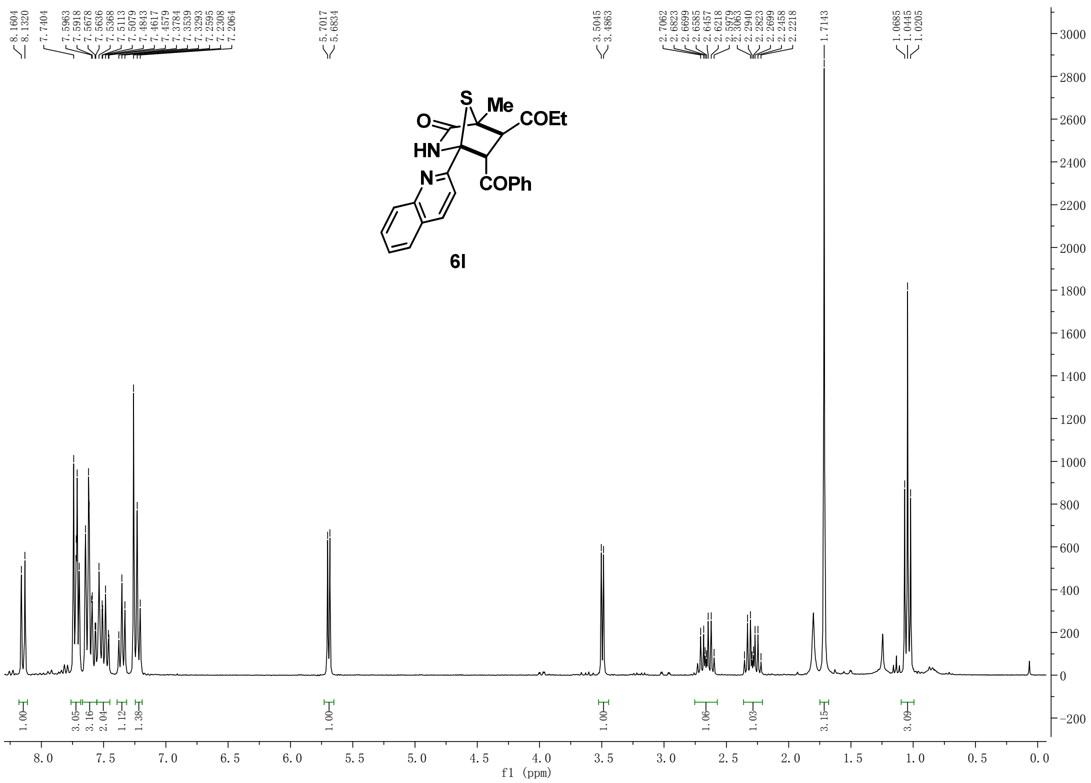

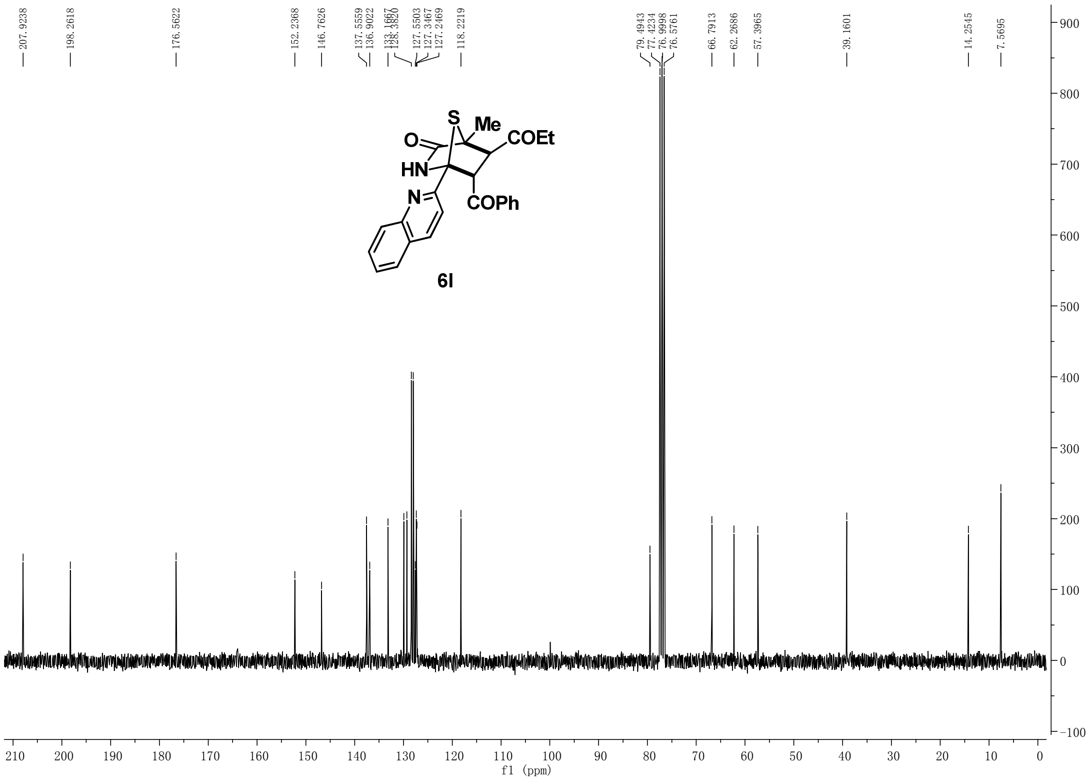

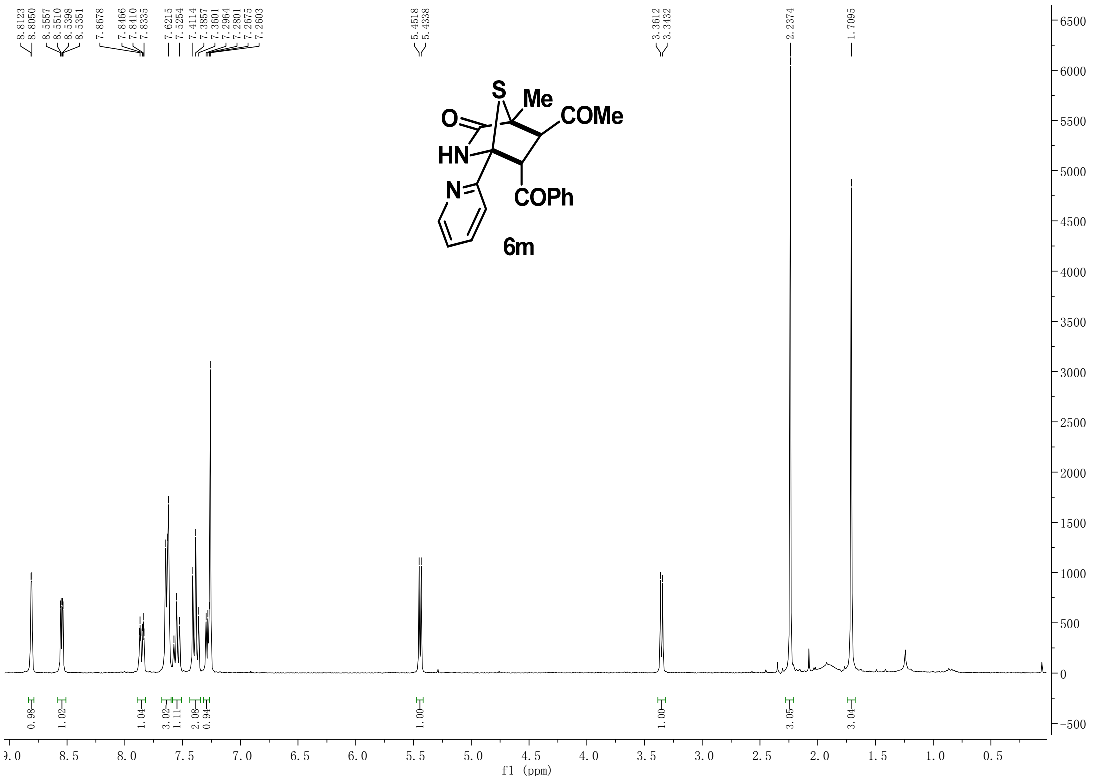

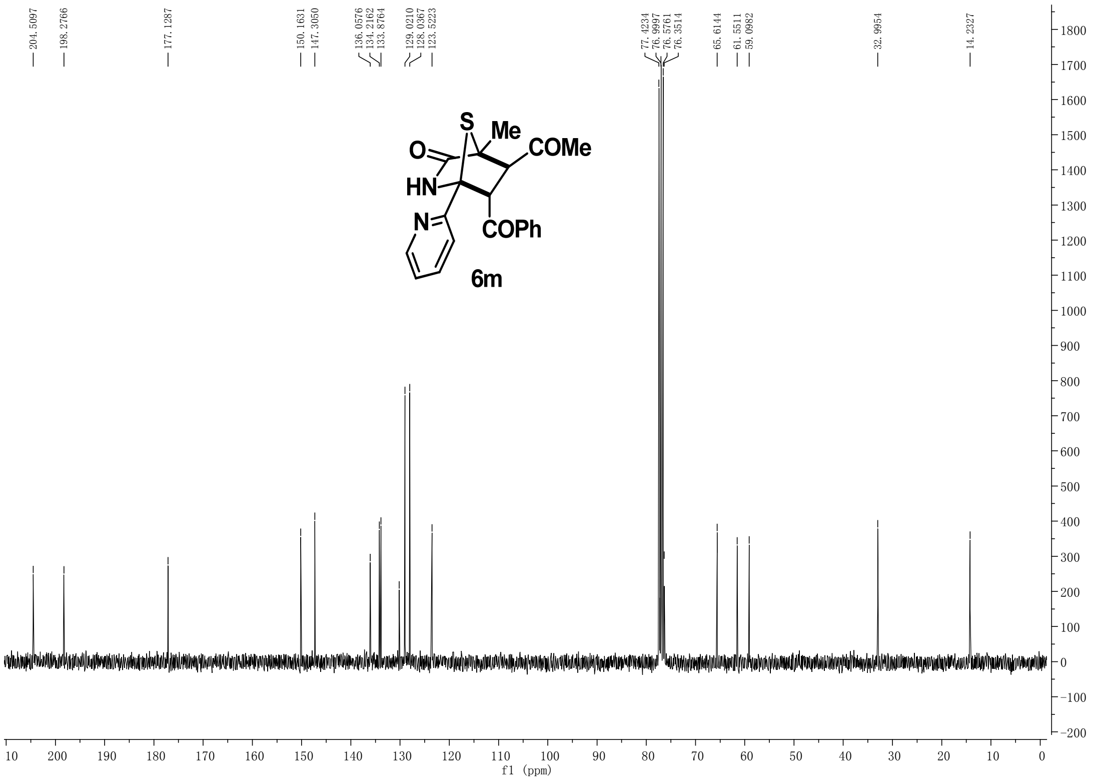

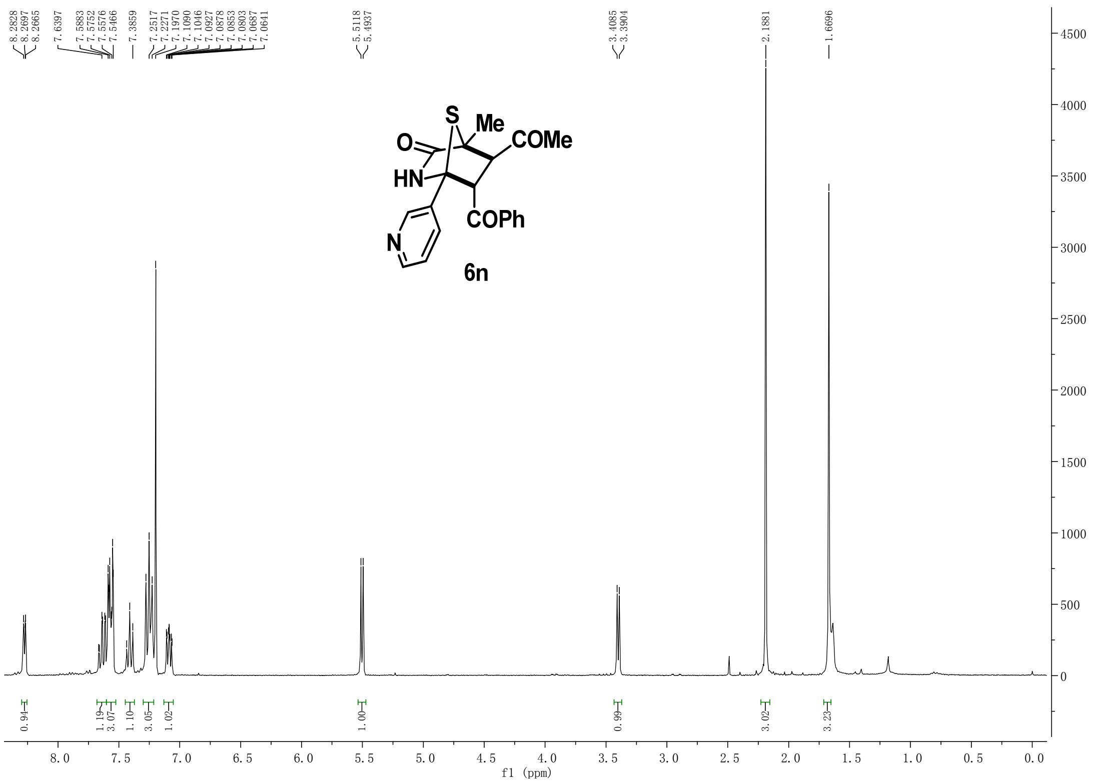

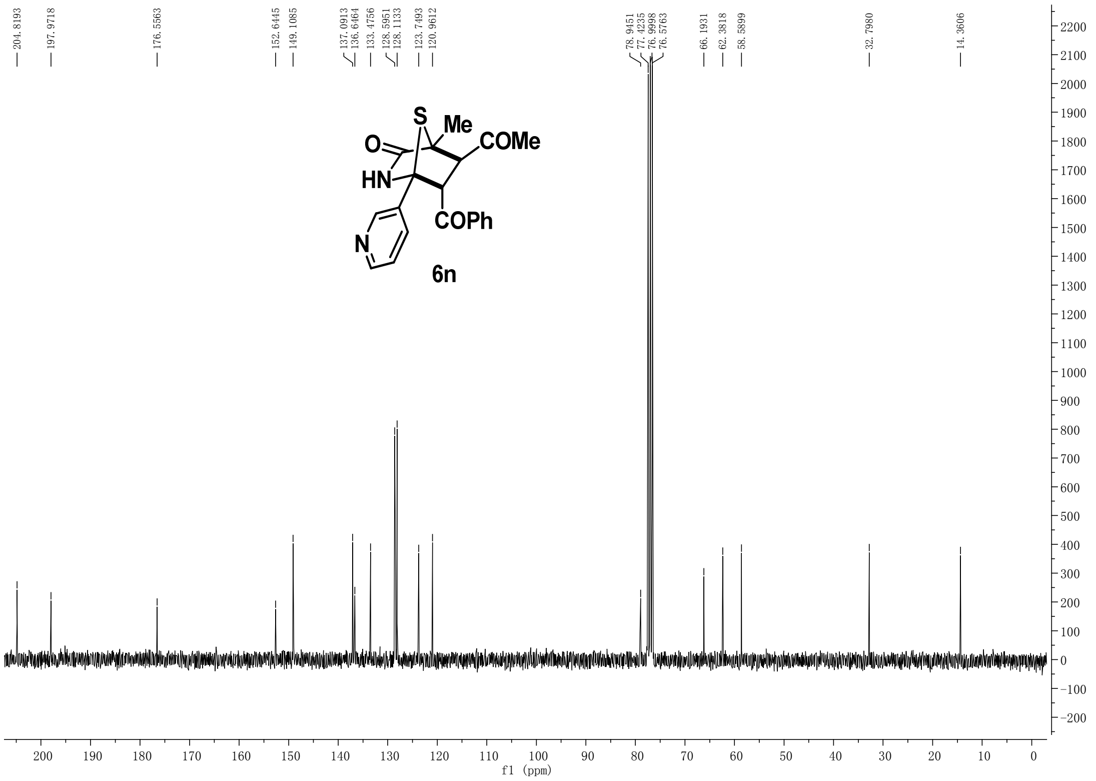

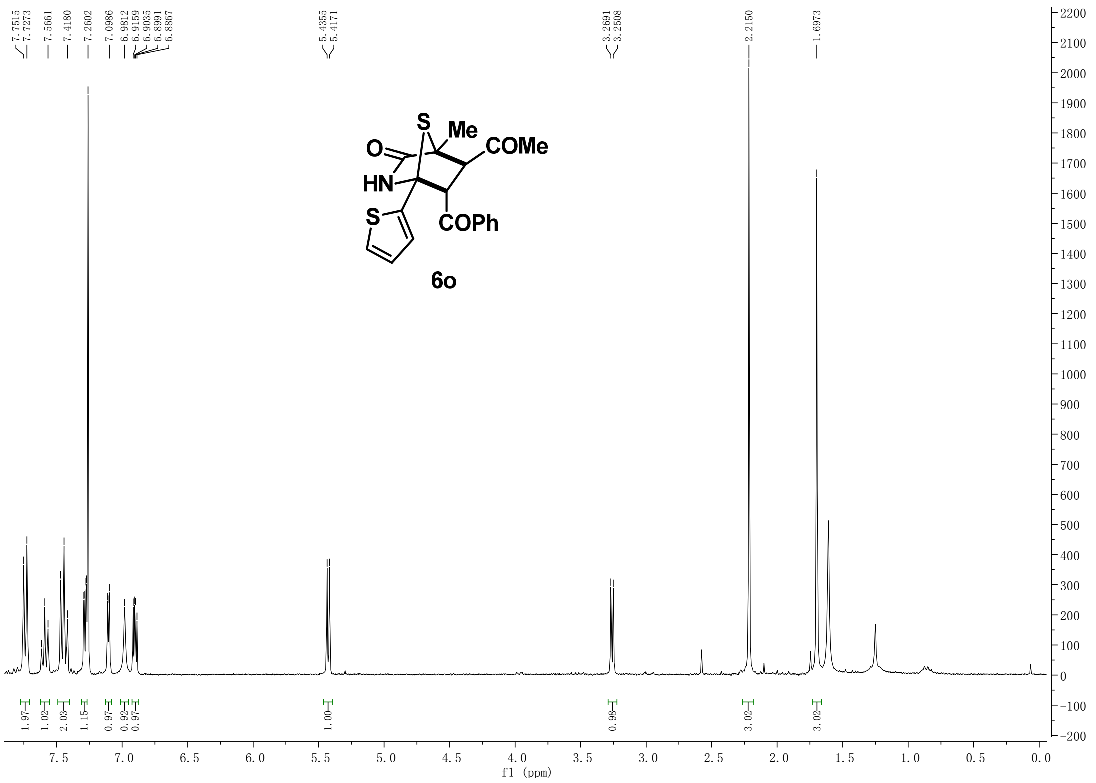

7.7515
7.7273
7.5661
7.4180
7.2602
7.0986
6.9812
6.9159
6.9035
6.8991
6.8867
5.4355
5.4171
3.2691
3.2508
2.2150
1.6973
S
Me
O
COMe
HN
S
COPh
6o
1.97
1.02
2.03
1.15
0.97
0.92
0.97
1.00
0.98
3.02
3.02
f1 (ppm)

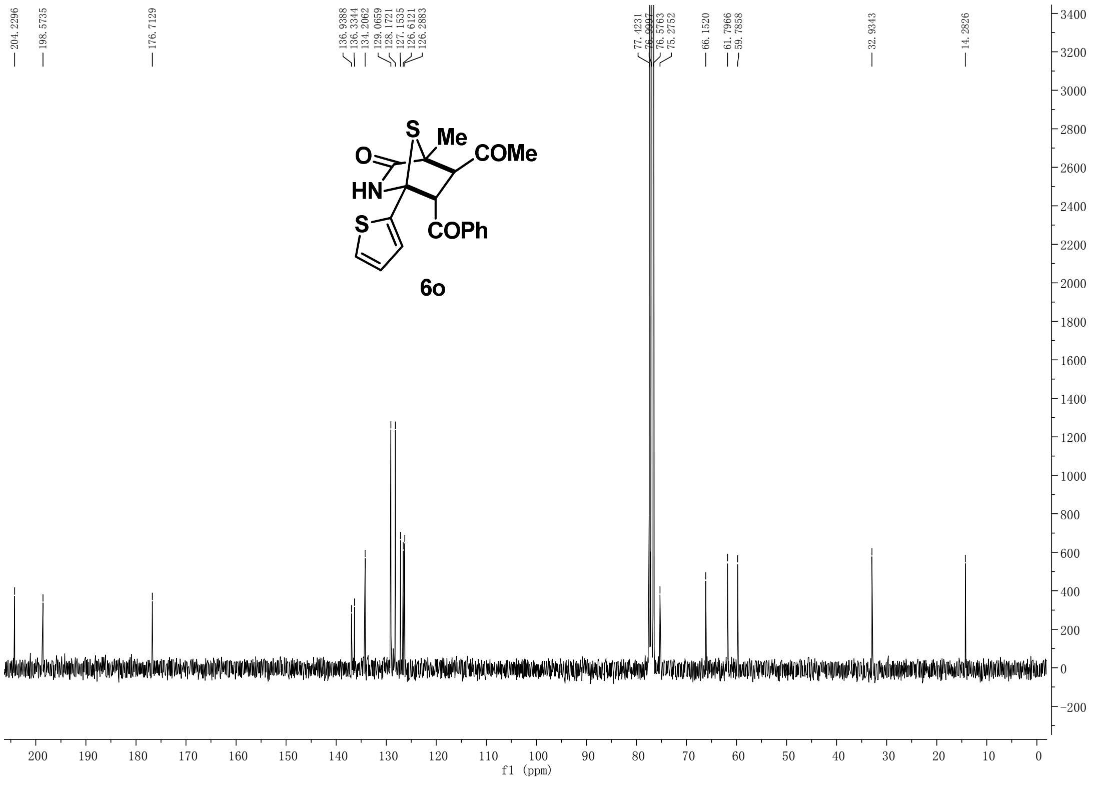

204.2296
198.5735
176.7129
136.9388
136.3344
134.2062
129.0659
128.1721
127.1535
126.6121
126.2883
77.4231
76.5763
75.2732
66.1520
61.7966
59.7858
32.9343
14.2826
S
Me
O
COMe
HN
S
COPh
6o
f1 (ppm)

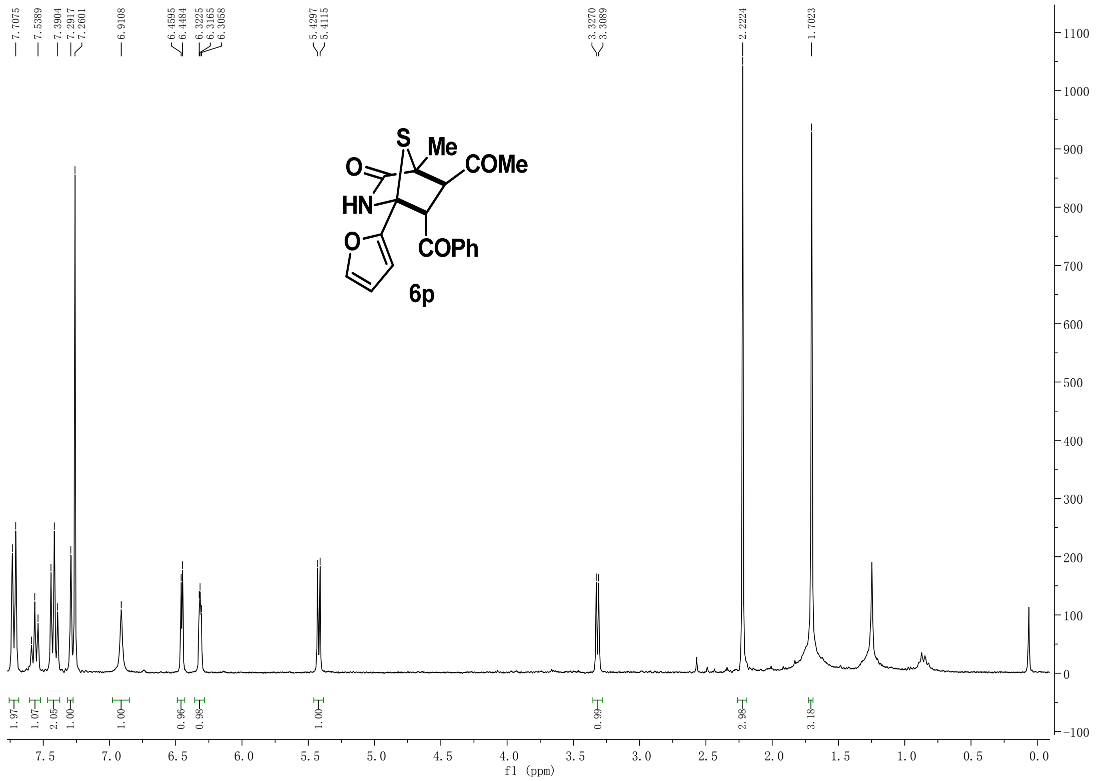

7.7075
7.5389
7.3904
7.2917
7.2601
6.9108
6.4595
6.4484
6.3225
6.3165
6.3058
5.4297
5.4115
3.3270
3.3089
2.2224
1.7023
S
Me
COMe
O
HN
O
COPh
6p
1.97
1.07
2.05
1.00
1.00
0.96
0.98
1.00
0.99
2.98
3.18
f1 (ppm)

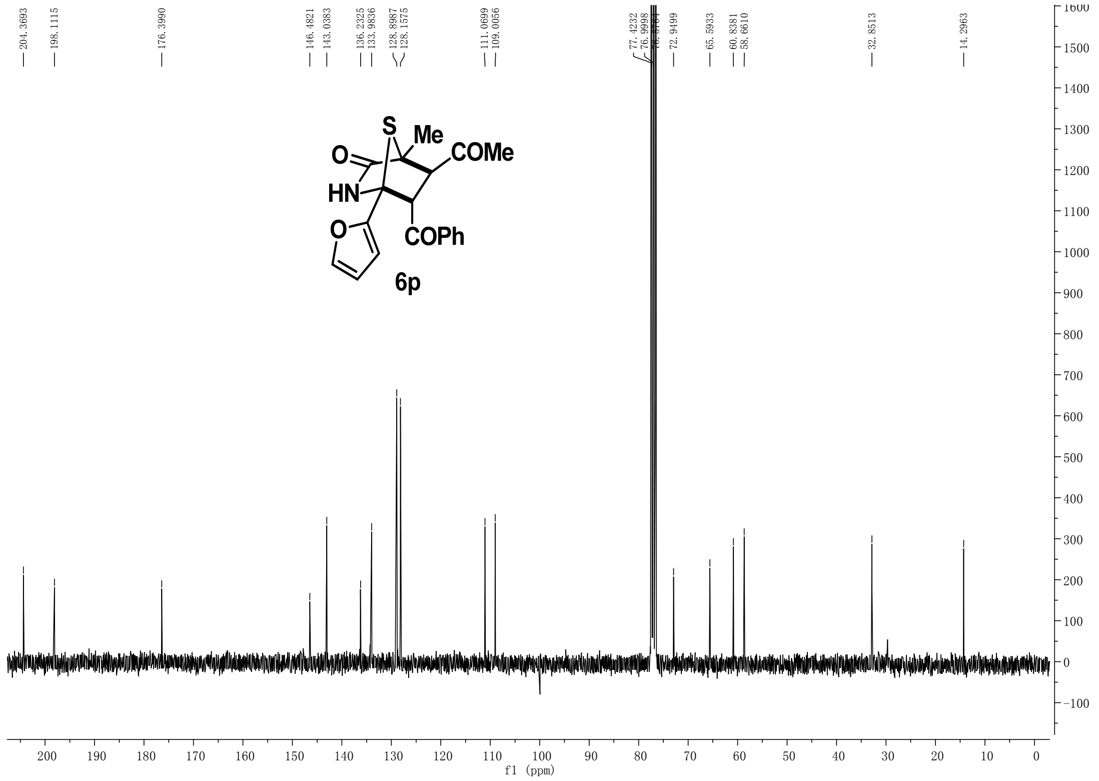

204.3693
198.1115
176.3990
146.4821
143.0383
136.2325
133.9836
128.8987
128.1575
111.0699
109.0056
77.4232
76.9998
72.9499
65.5933
60.8381
58.6610
32.8513
14.2963
S
Me
COMe
O
HN
O
COPh
6p
f1 (ppm)

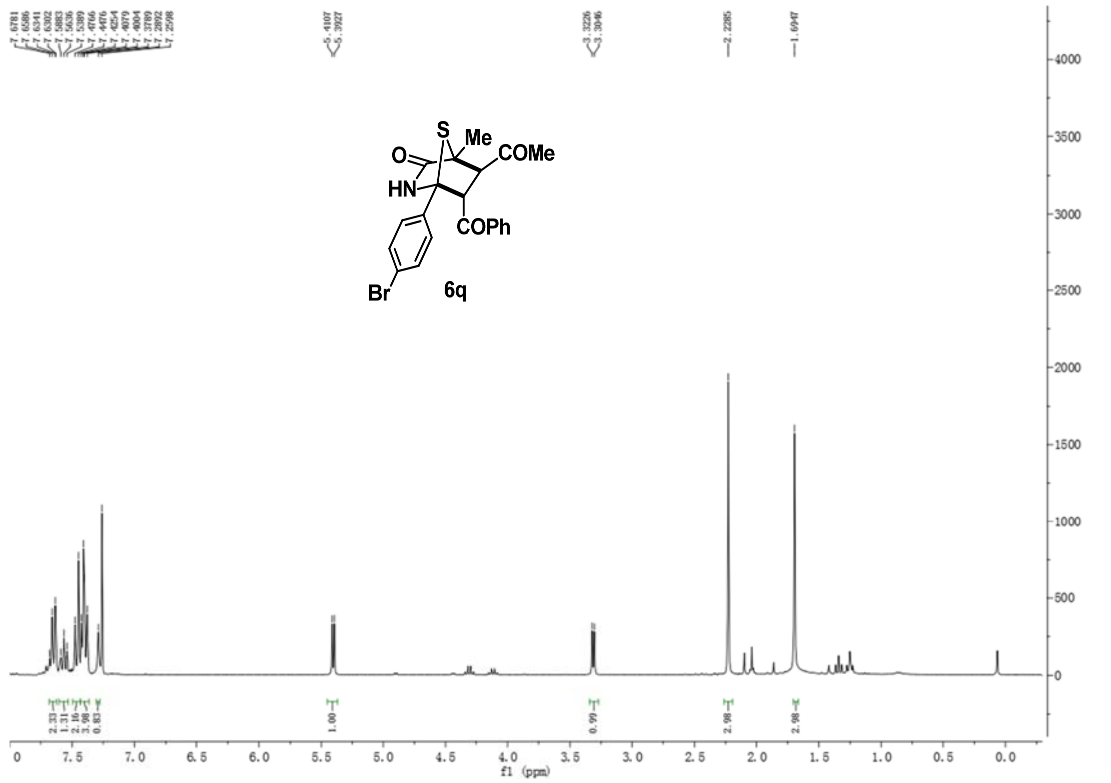
S
Me
COMe
O
HN
COPh
Br
6q
f1 (ppm)

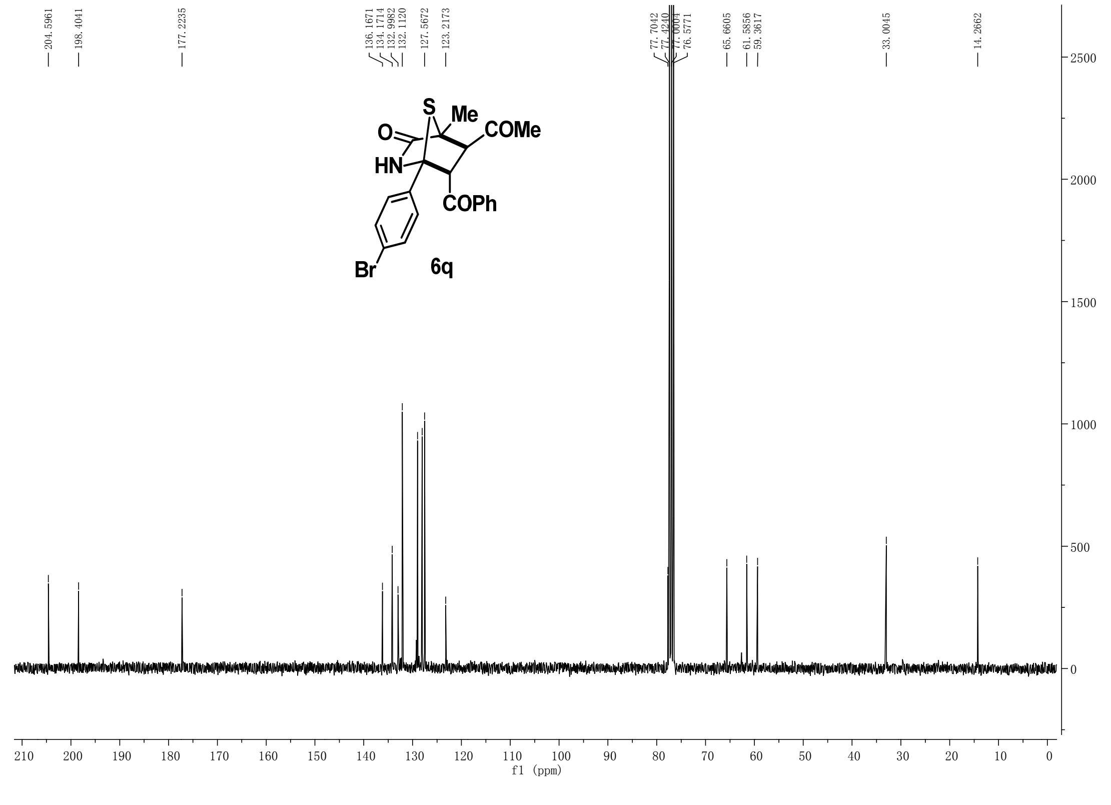
S
Me
COMe
O
HN
COPh
Br
6q
f1 (ppm)

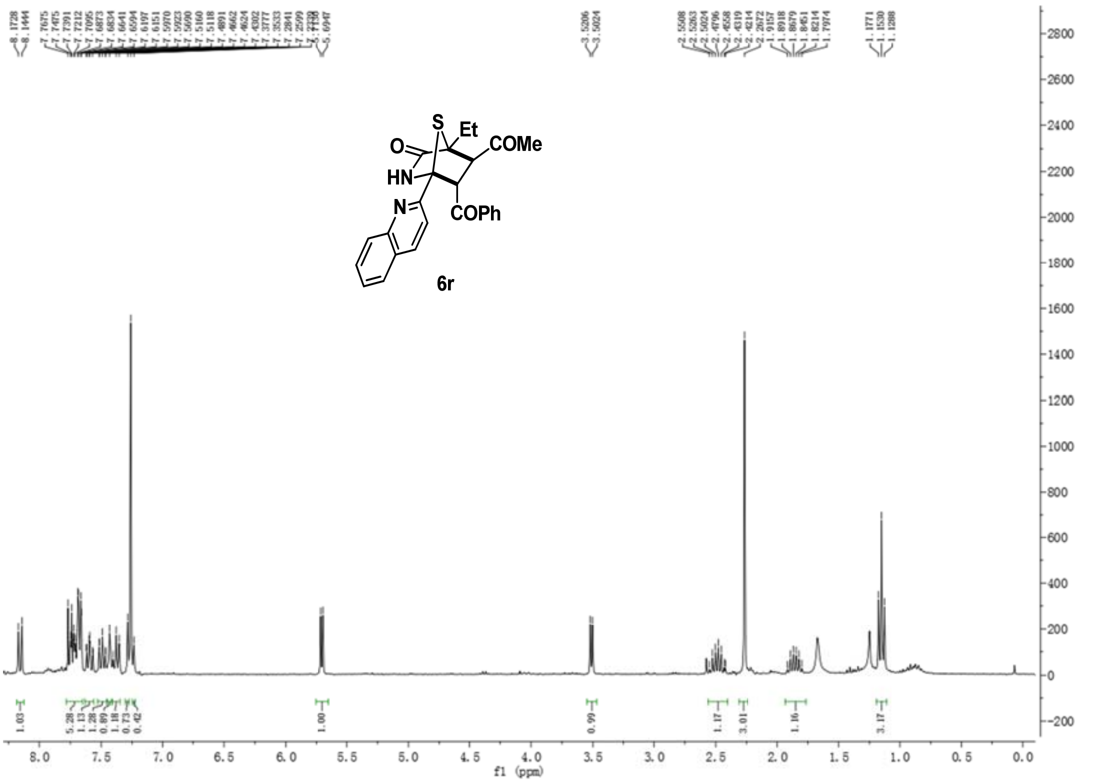

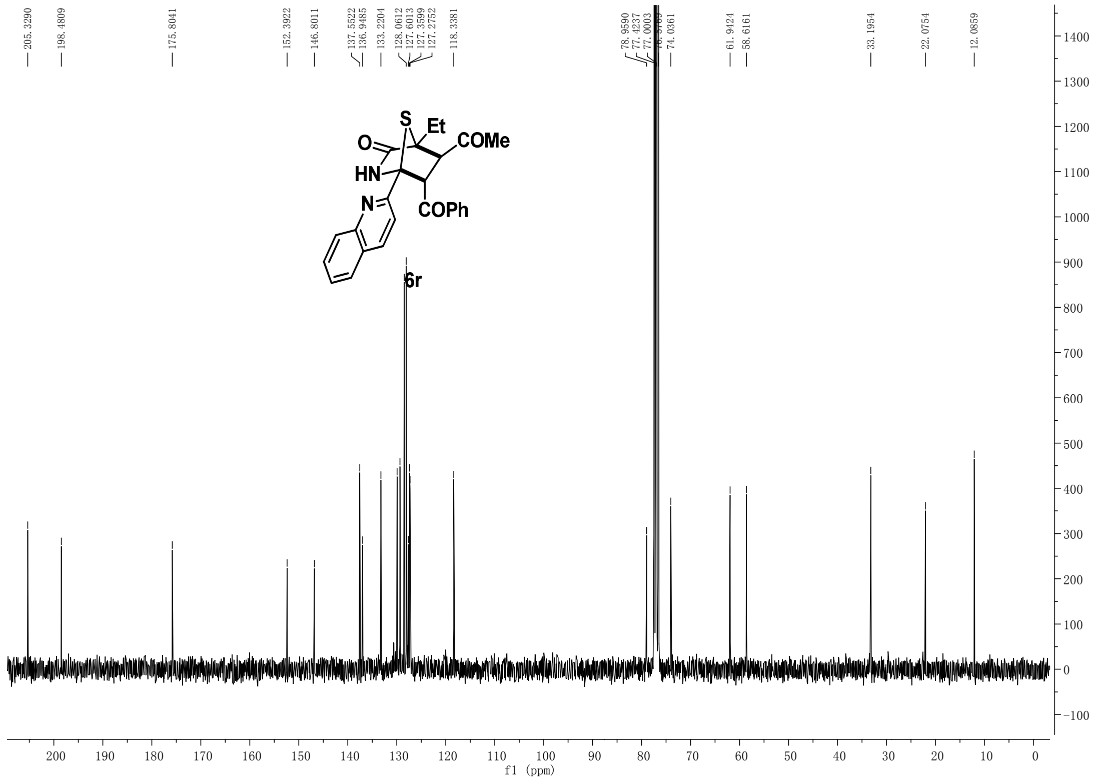

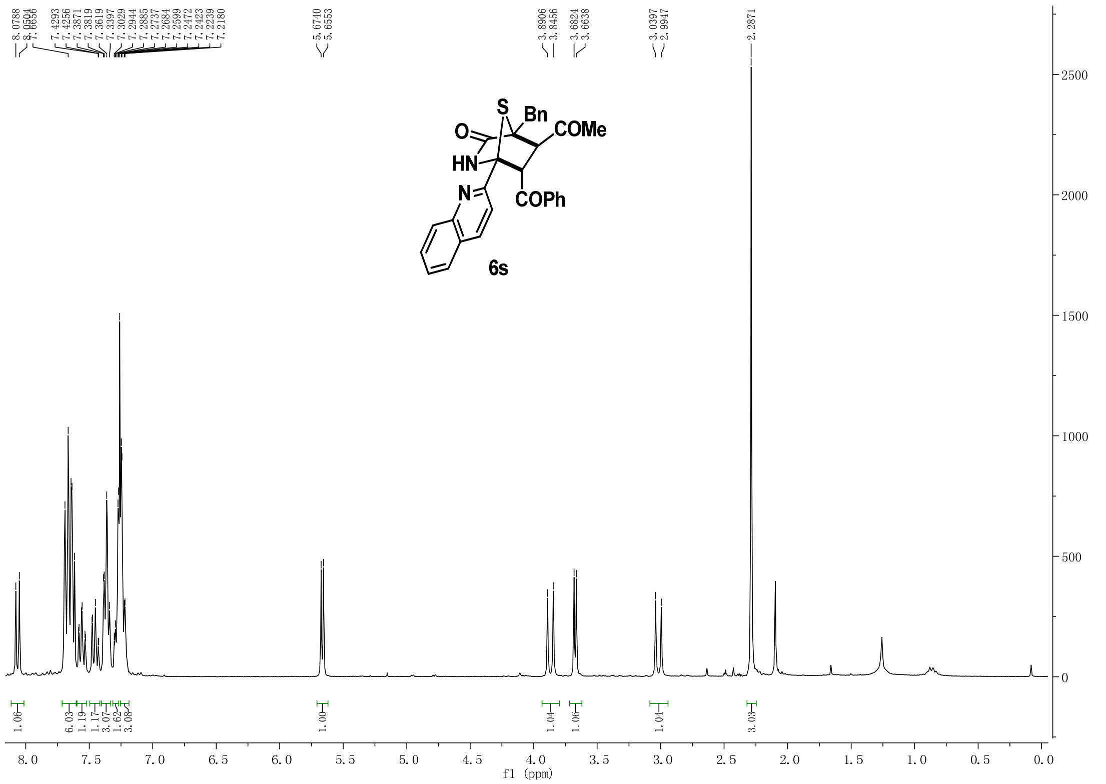

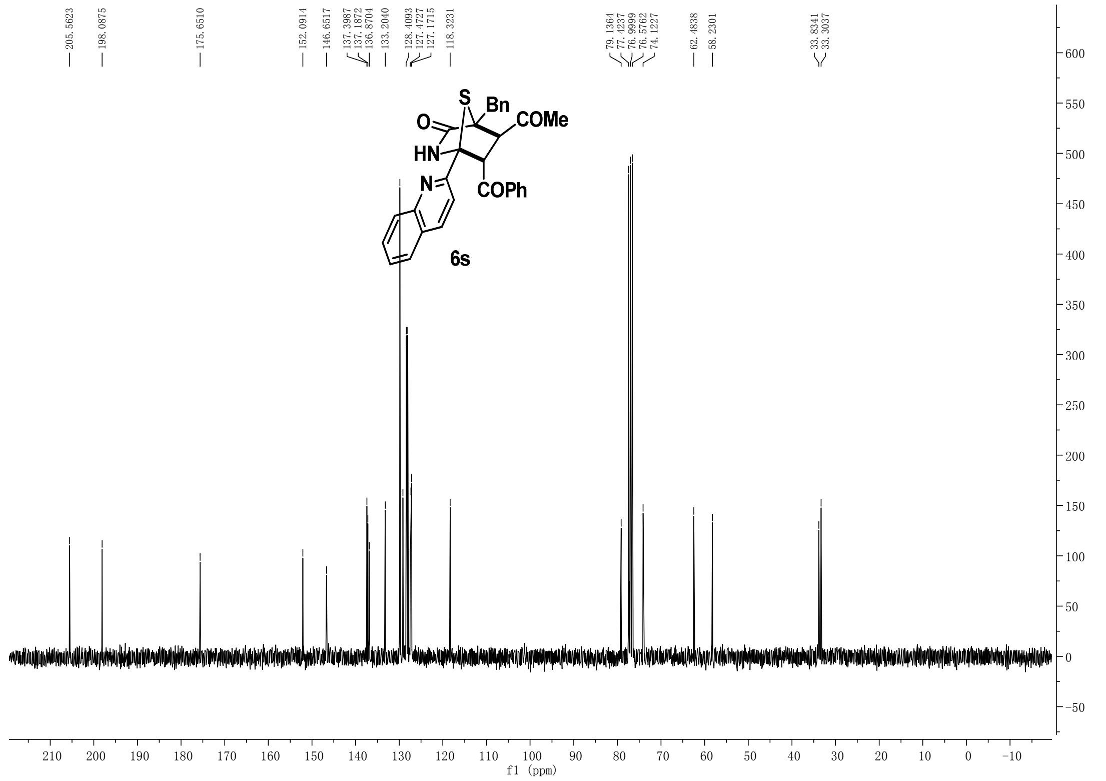

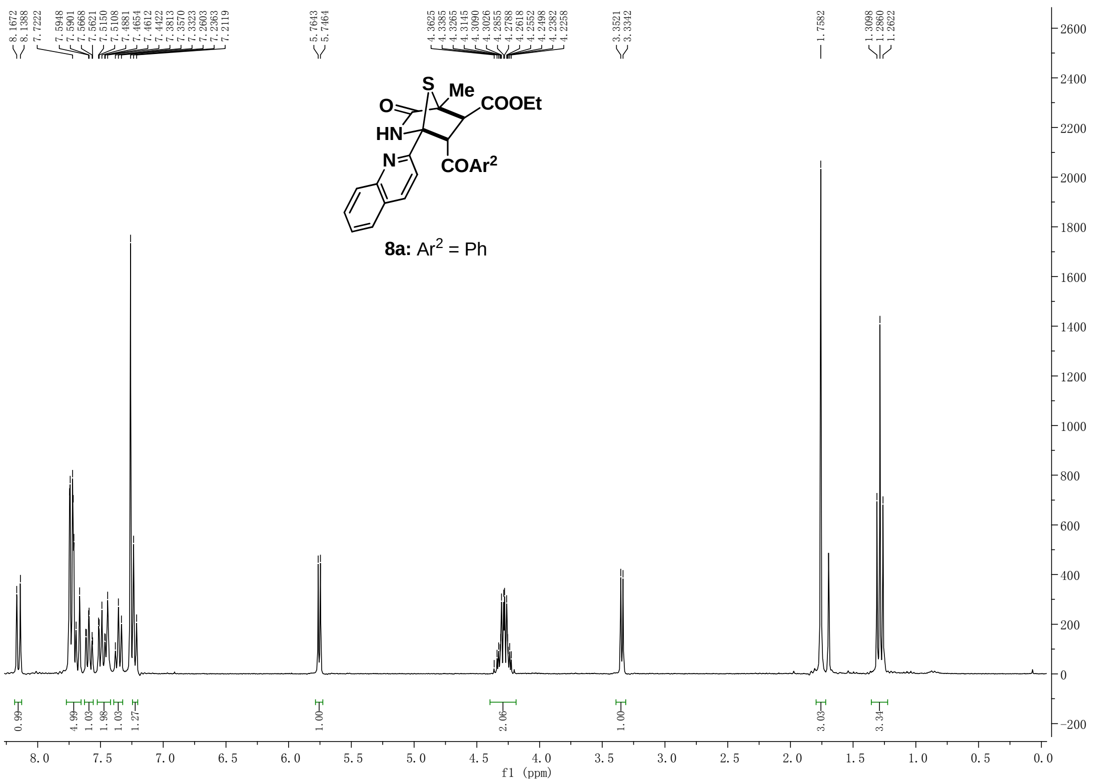

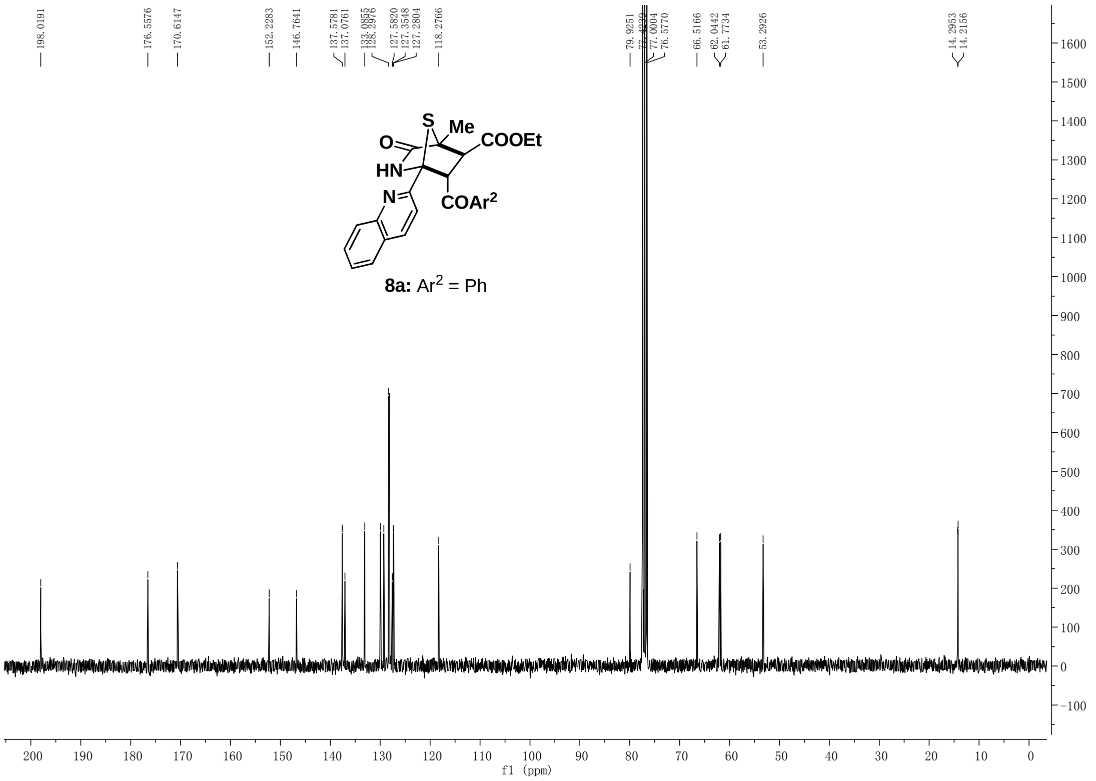

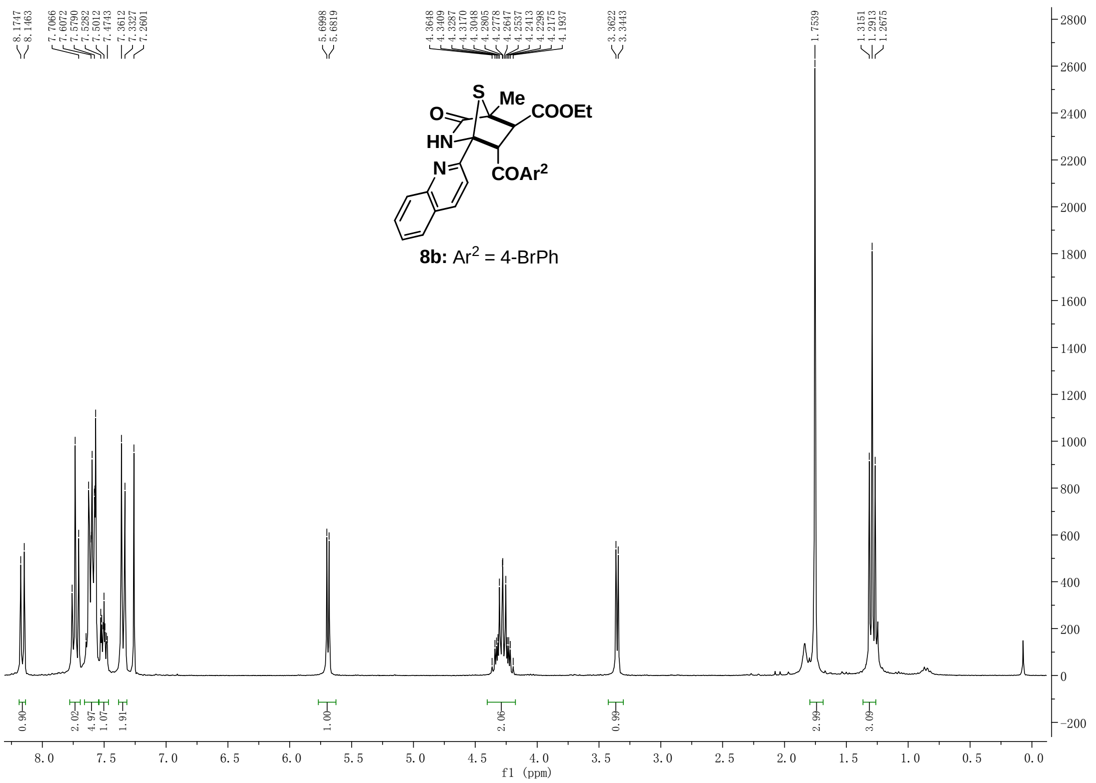
8b: Ar2 = 4-BrPh
COOEt
COAr2
f1 (ppm)

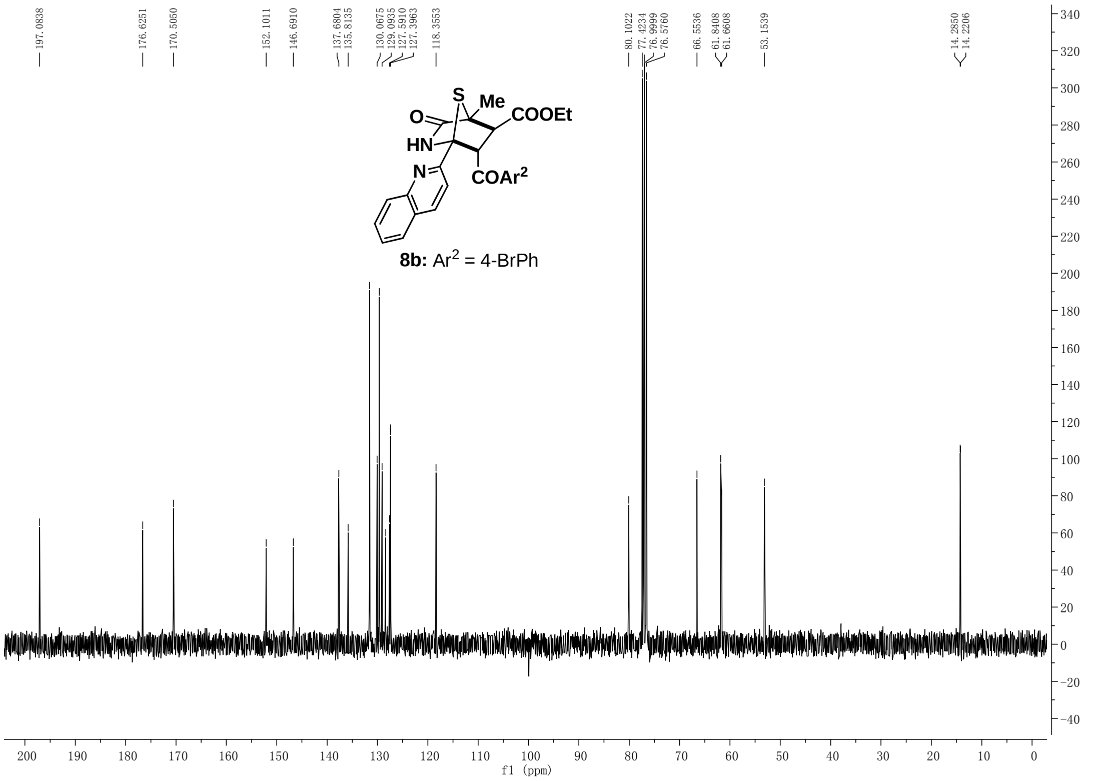
8b: Ar2 = 4-BrPh
COOEt
COAr2
f1 (ppm)

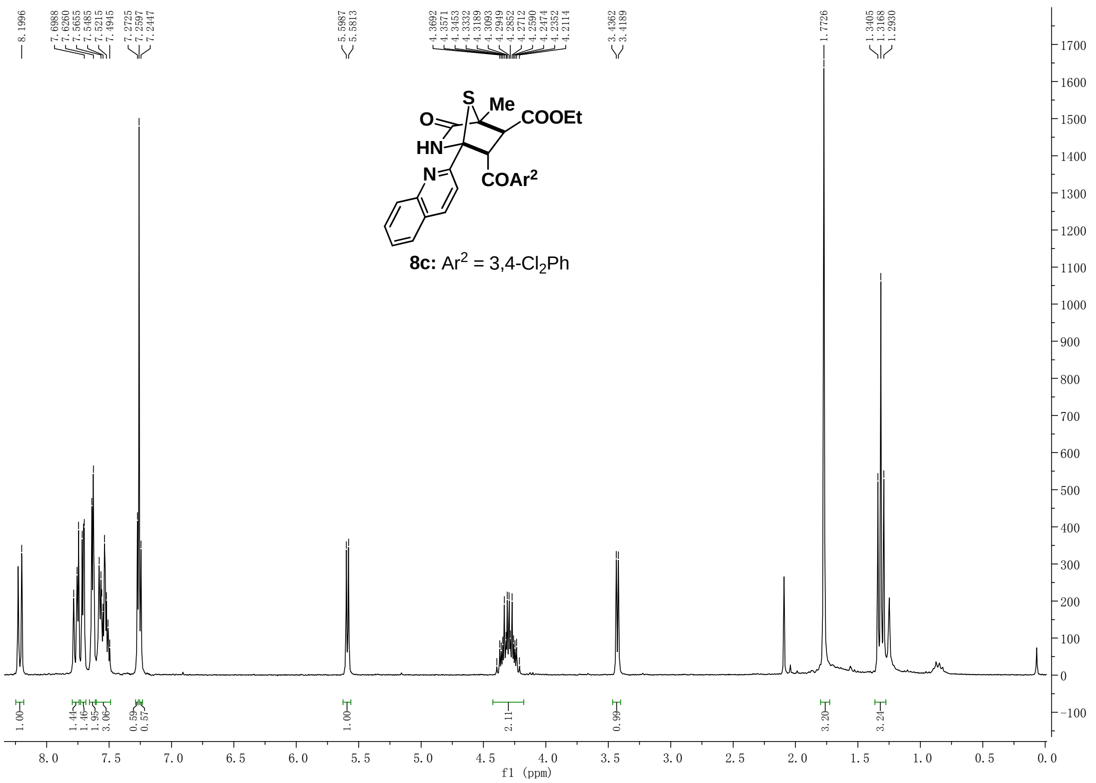
8c: Ar2 = 3,4-Cl2Ph
f1 (ppm)

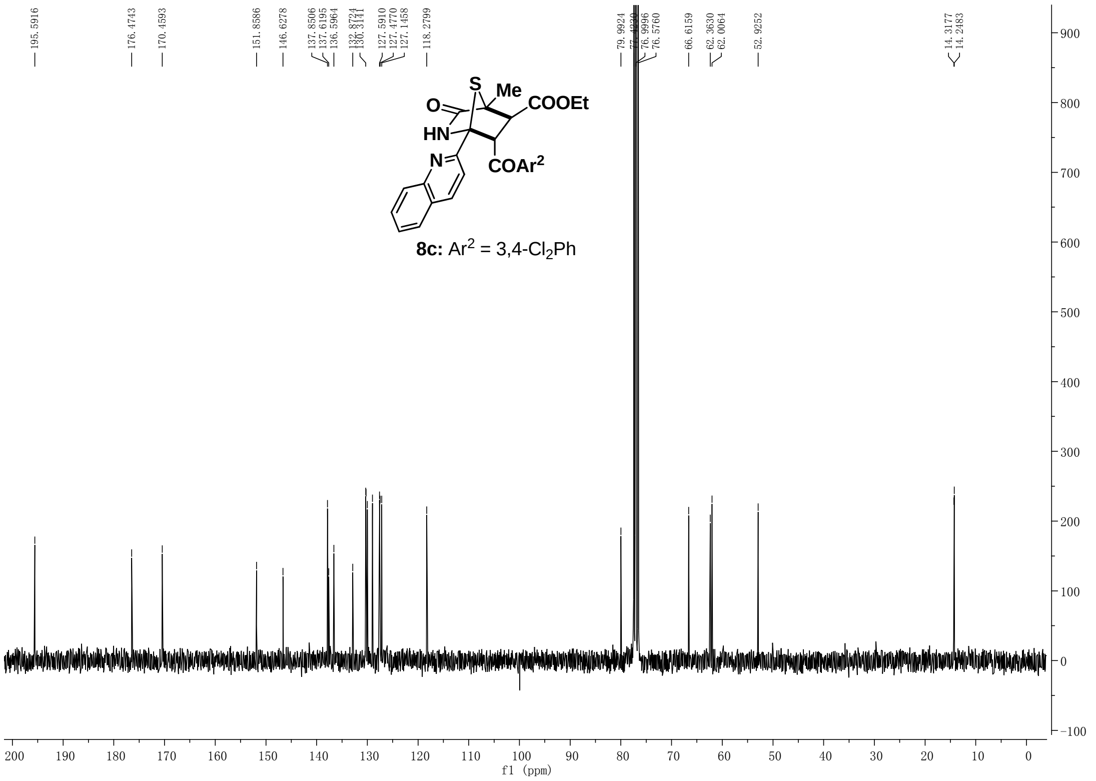
8c: Ar2 = 3,4-Cl2Ph
f1 (ppm)

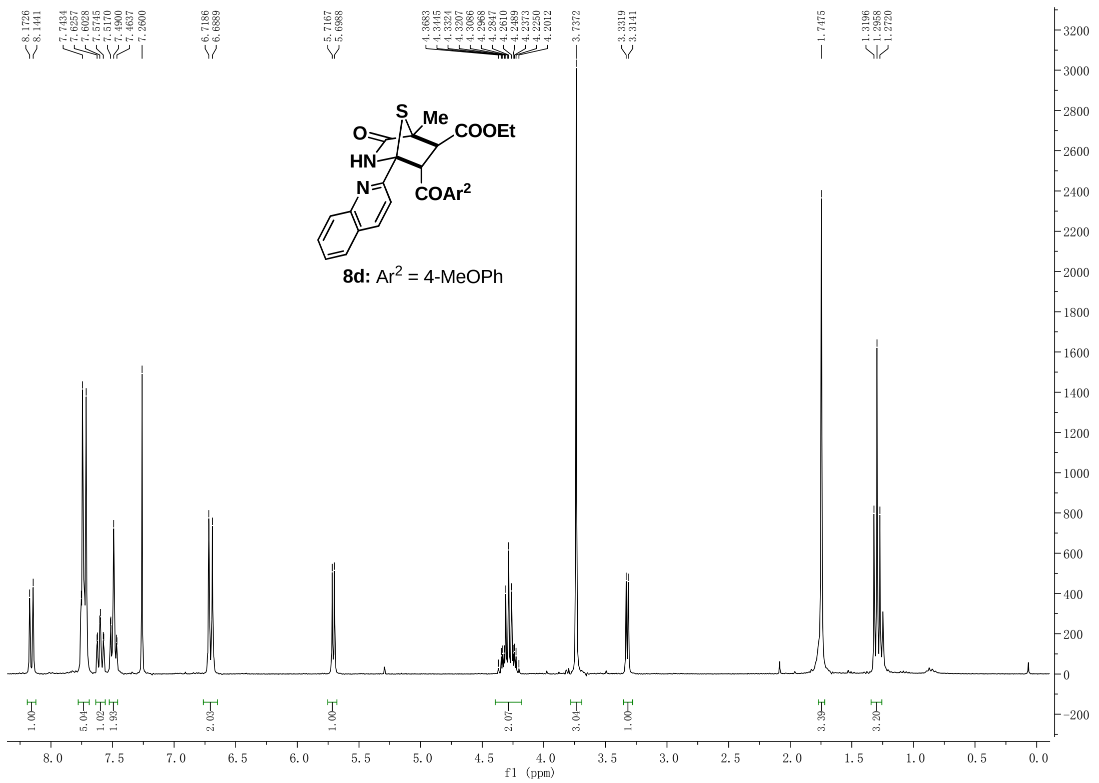

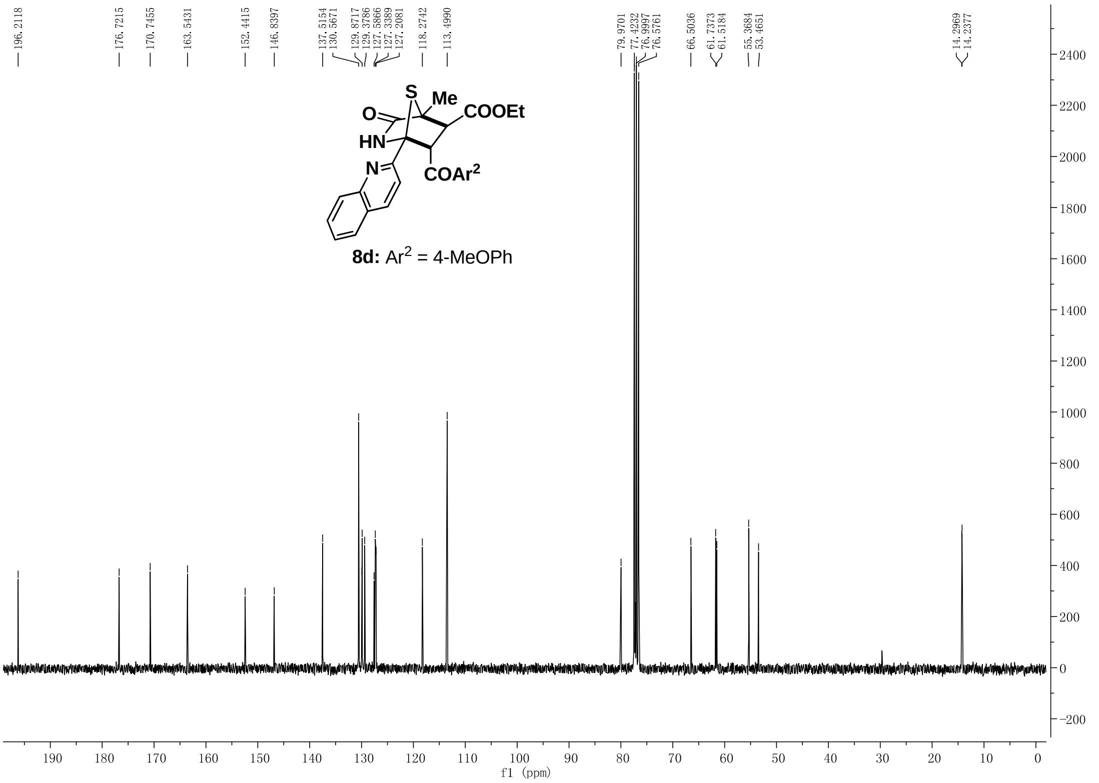

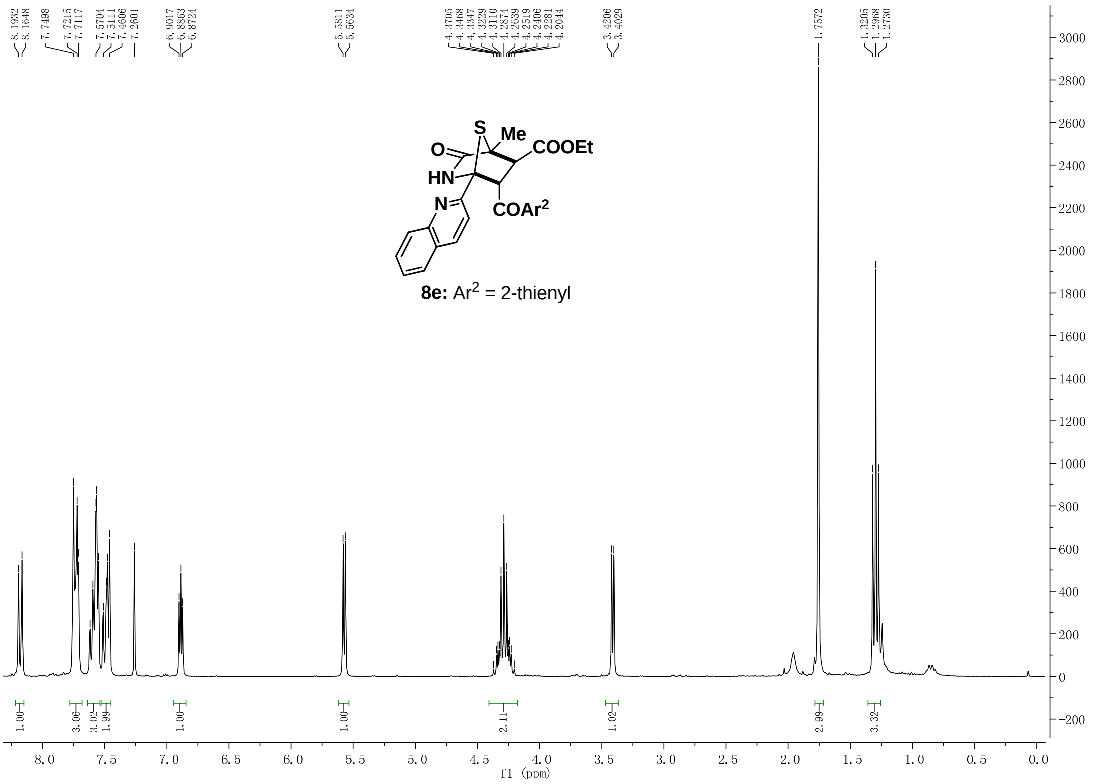
8.1932
8.1648
7.7498
7.7215
7.7117
7.5704
7.5111
7.4606
7.2601
6.9017
6.8863
6.8724
5.5811
5.5634
4.3705
4.3468
4.3347
4.3229
4.3110
4.2874
4.2639
4.2519
4.2406
4.2281
4.2044
3.4206
3.4029
1.7572
1.3205
1.2968
1.2730
S
Me
COOEt
O
HN
N
COAr2
8e: Ar2 = 2-thienyl
1.00
3.06
3.02
1.99
1.00
1.00
2.11
1.02
2.99
3.32
f1 (ppm)

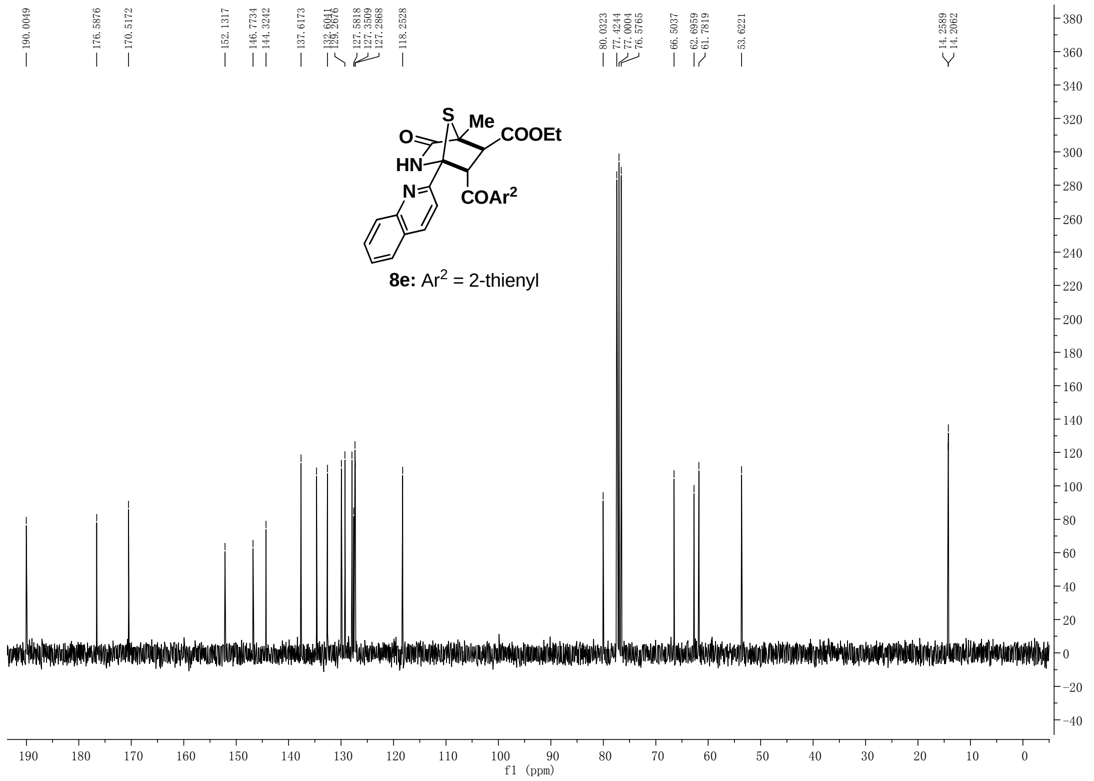
190.0049
176.5876
170.5172
152.1317
146.7734
144.3242
137.6173
132.6041
129.2676
127.5818
127.3509
127.2868
118.2528
80.0323
77.4244
77.0004
76.5765
66.5037
62.6959
61.7819
53.6221
14.2589
14.2062
S
Me
COOEt
O
HN
N
COAr2
8e: Ar2 = 2-thienyl
f1 (ppm)

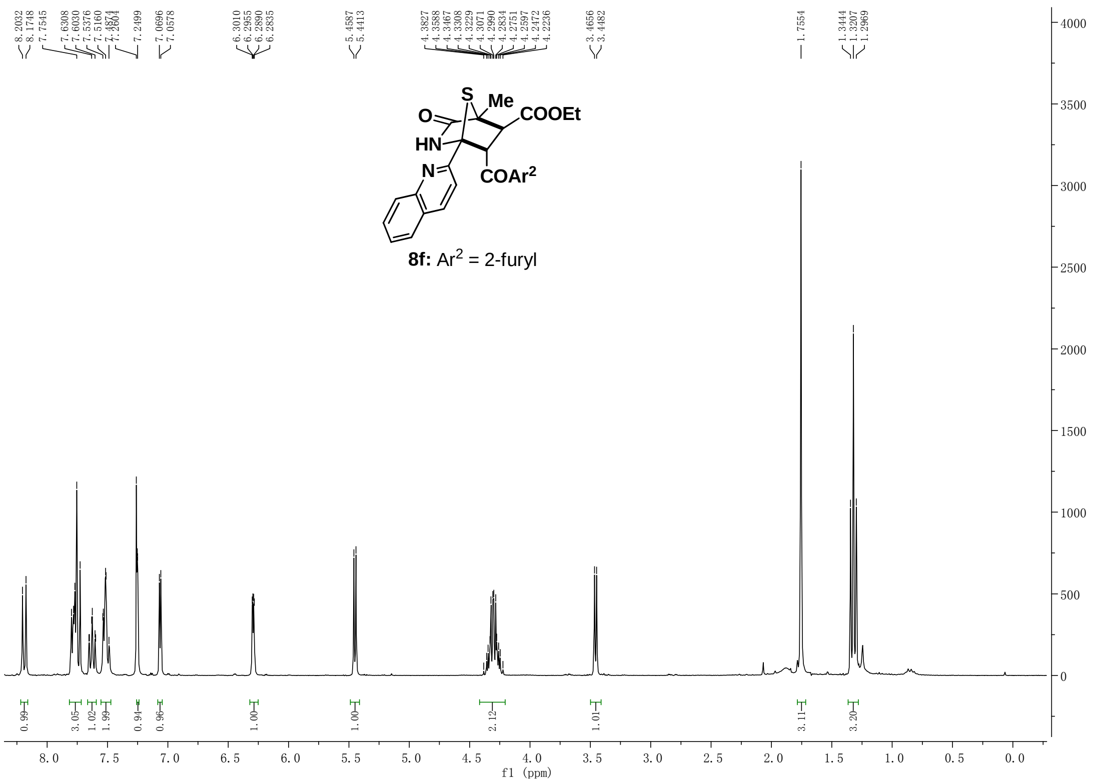
8.2032
8.1748
7.7545
7.6308
7.6030
7.5376
7.5160
7.2499
7.0696
7.0578
6.3010
6.2955
6.2890
6.2835
5.4587
5.4413
4.3827
4.3588
4.3467
4.3308
4.3229
4.3071
4.2990
4.2834
4.2751
4.2597
4.2472
4.2236
3.4656
3.4482
1.7554
1.3444
1.3207
1.2969
S
Me
COOEt
O
HN
N
COAr2
8f: Ar2 = 2-furyl
0.99
3.05
1.02
1.99
0.94
0.96
1.00
1.00
2.12
1.01
3.11
3.20
f1 (ppm)

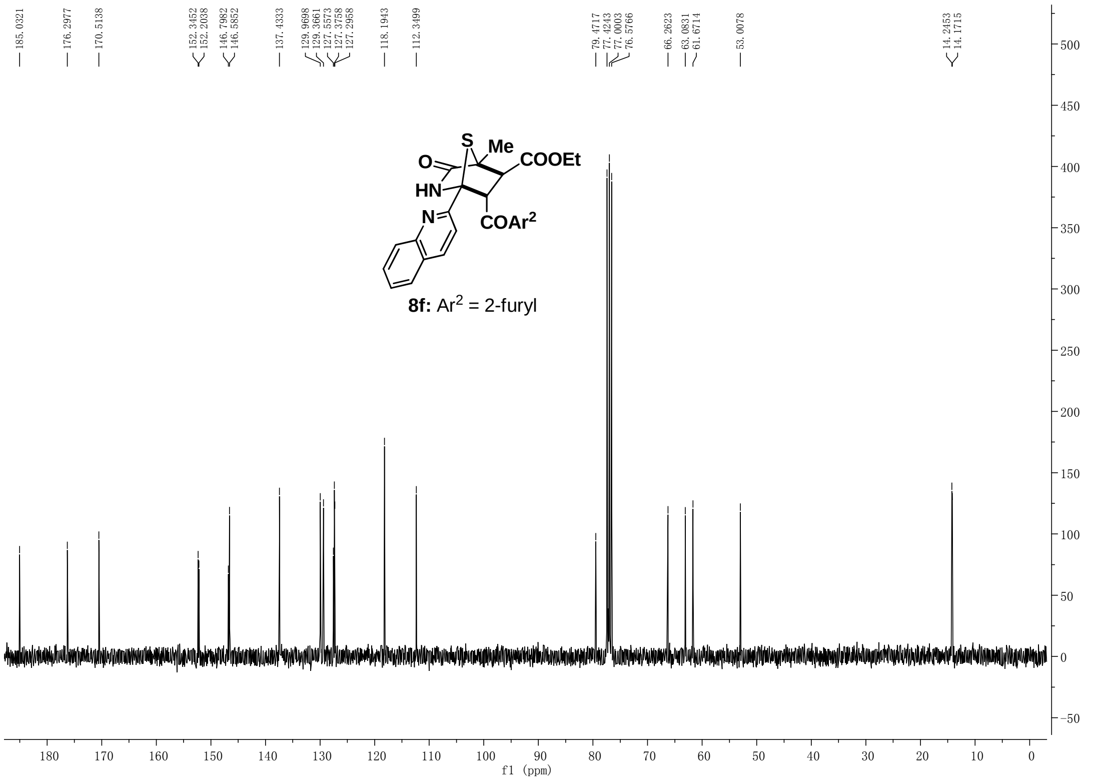
185.0321
176.2977
170.5138
152.3452
152.2038
146.7982
146.5852
137.4333
129.9698
129.3661
127.5573
127.3758
127.2958
118.1943
112.3499
79.4717
77.4243
77.0003
76.5766
66.2623
63.0831
61.6714
53.0078
14.2453
14.1715
S
Me
COOEt
O
HN
N
COAr2
8f: Ar2 = 2-furyl
f1 (ppm)

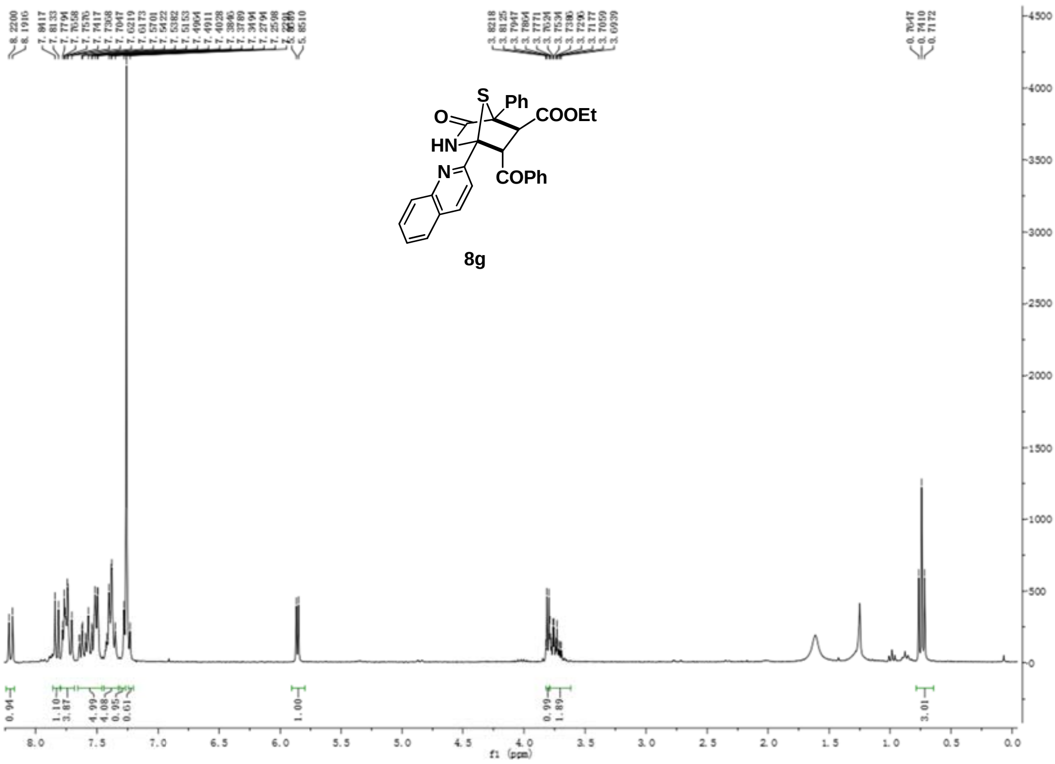
S
Ph
COOEt
O
HN
N
COPh
8g
f1 (ppm)

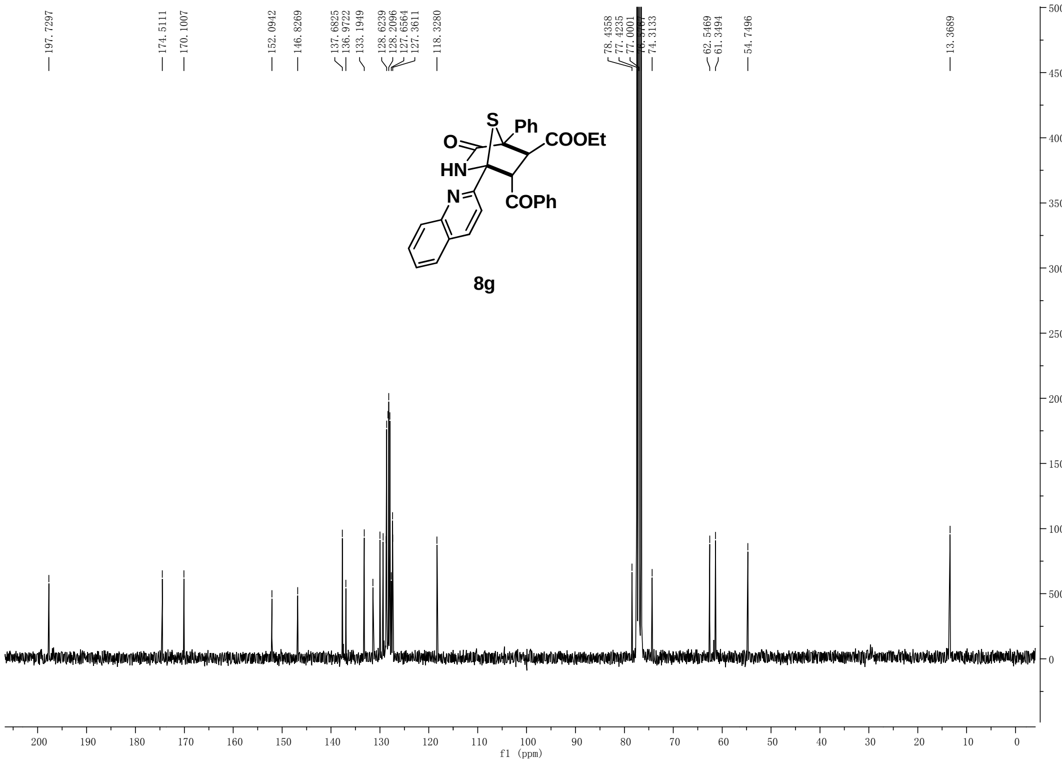
197.7297
174.5111
170.1007
152.0942
146.8269
137.6825
136.9722
133.1949
128.6239
128.2096
127.6564
127.3611
118.3280
78.4358
77.4235
77.0001
74.3133
62.5469
61.3494
54.7496
13.3689
S
Ph
COOEt
O
HN
N
COPh
8g
f1 (ppm)

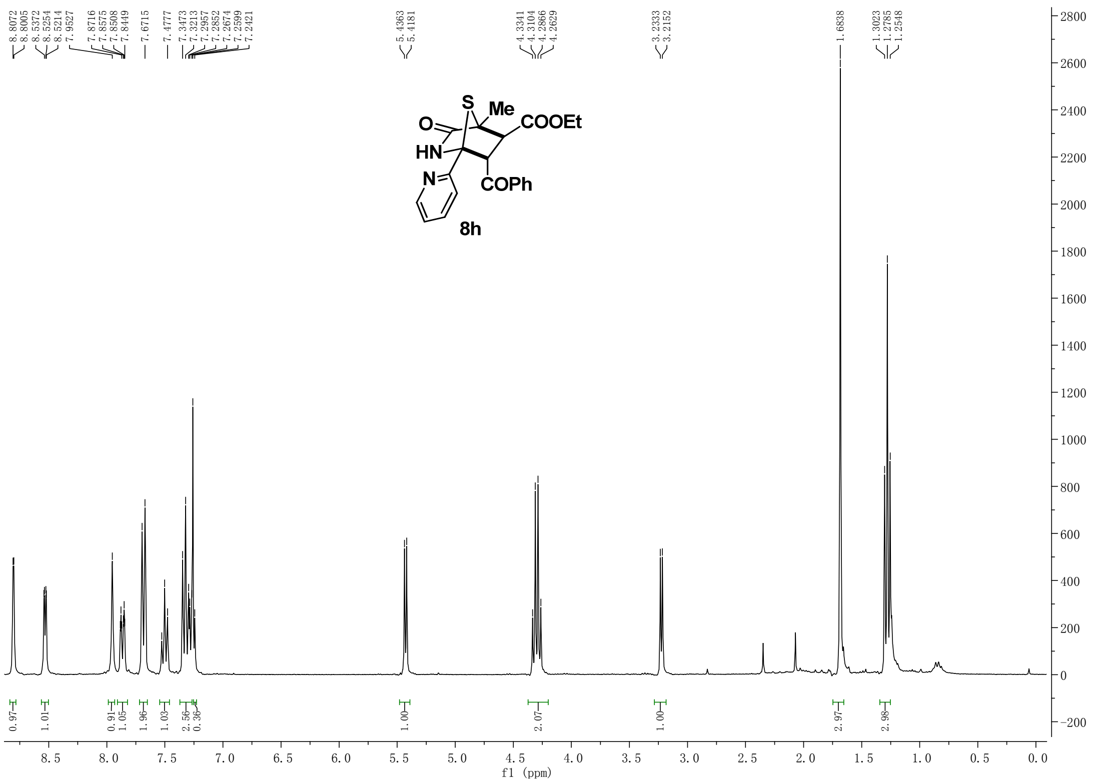

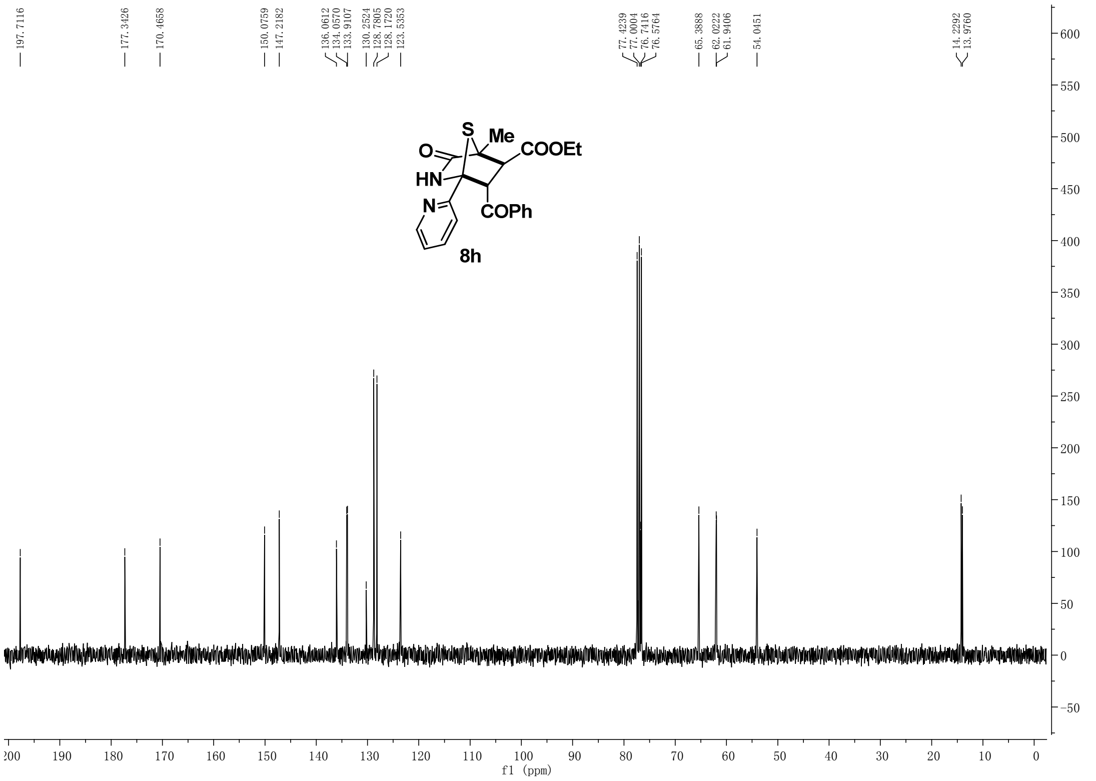

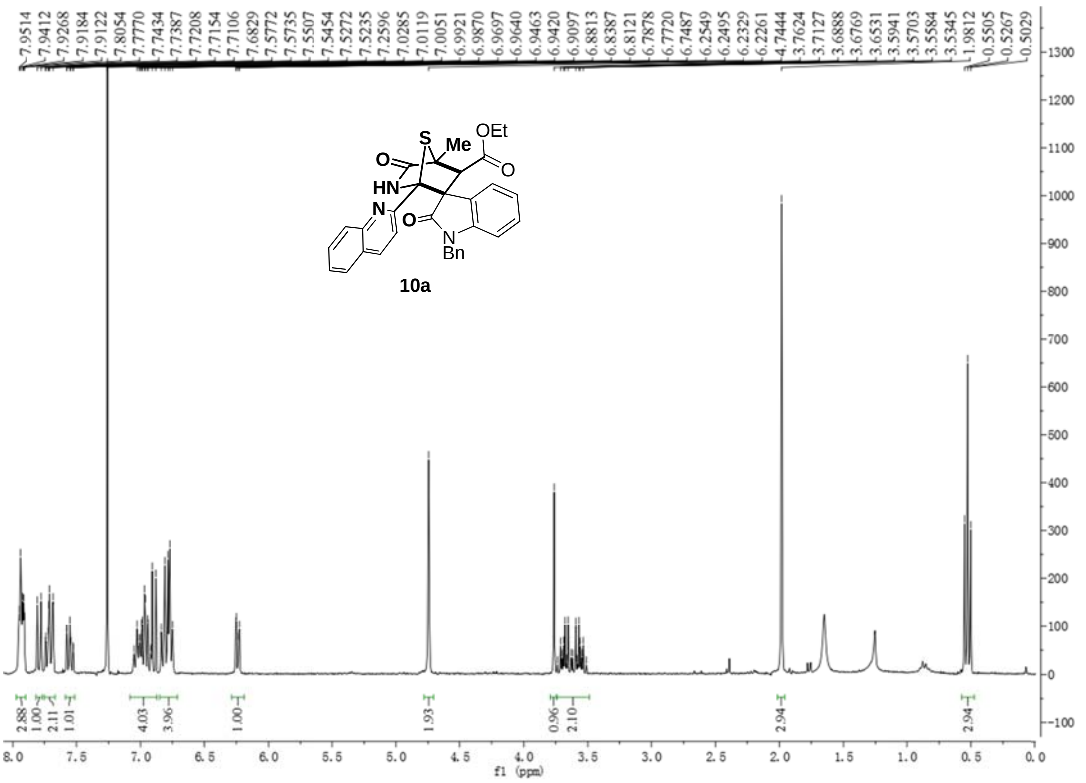
10a
f1 (ppm)

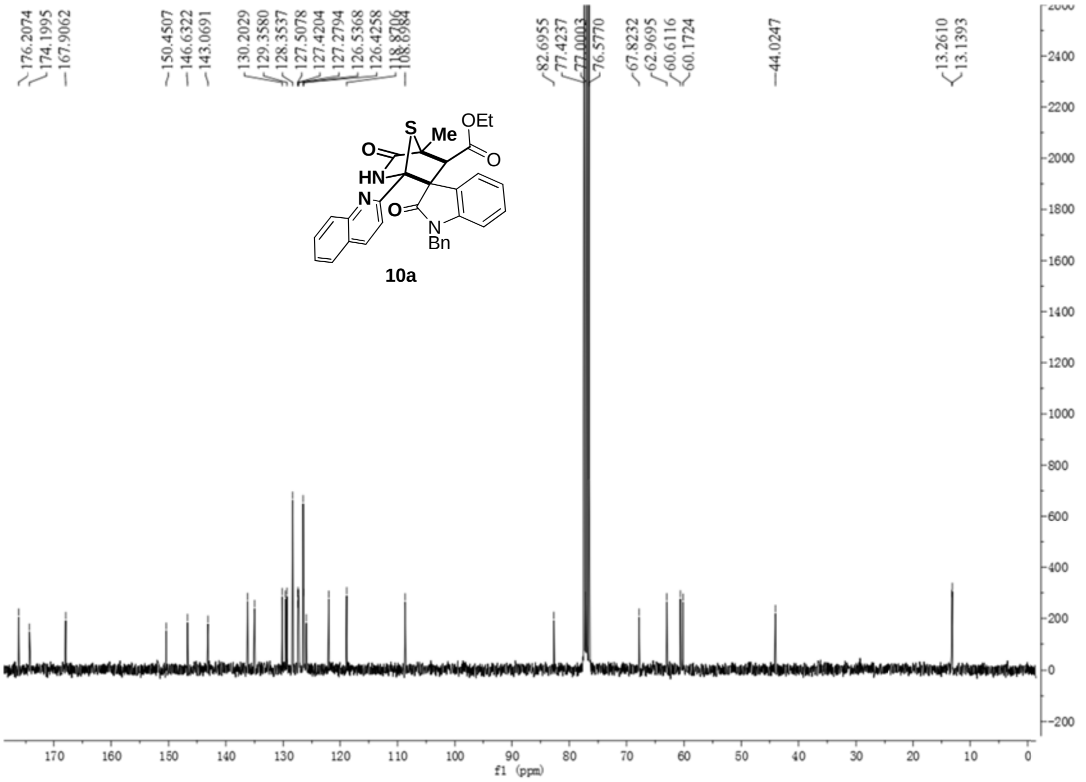
10a
f1 (ppm)

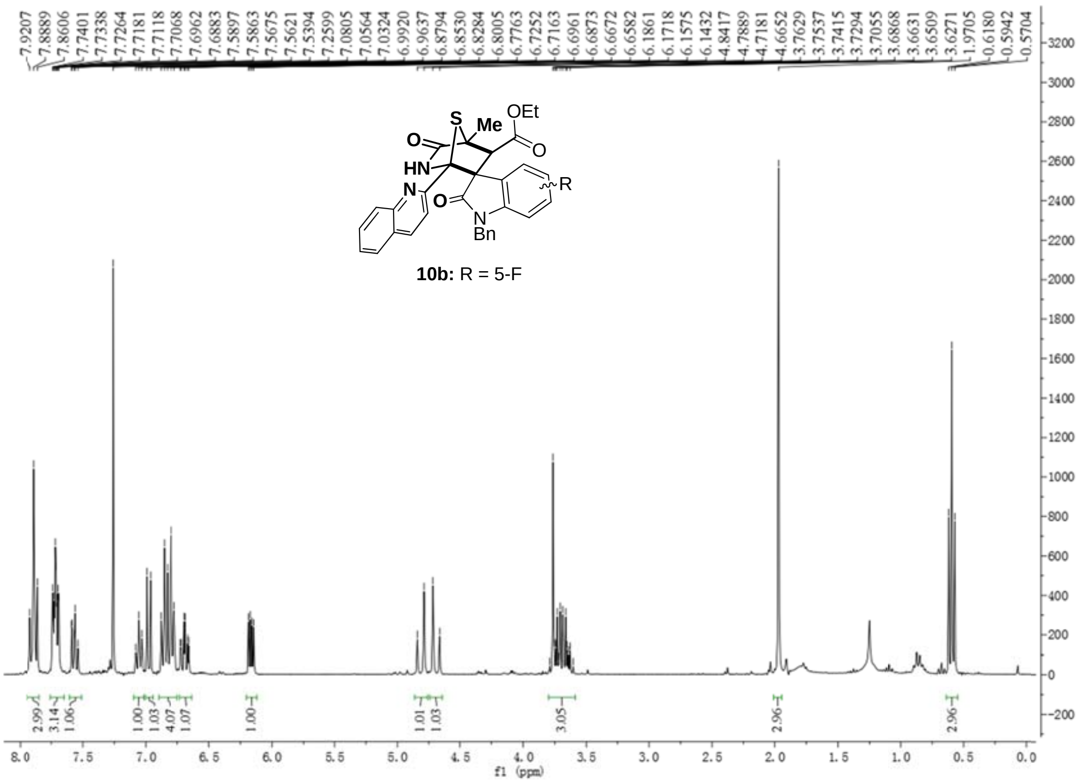
7.9207
7.8889
7.8606
7.7401
7.7338
7.7264
7.7181
7.7118
7.7068
7.6962
7.6883
7.5897
7.5863
7.5675
7.5621
7.5394
7.2599
7.0805
7.0564
7.0324
6.9920
6.9637
6.8794
6.8530
6.8284
6.8005
6.7763
6.7252
6.7163
6.6961
6.6873
6.6672
6.6582
6.1861
6.1718
6.1575
6.1432
4.8417
4.7889
4.7181
4.6652
3.7629
3.7537
3.7415
3.7294
3.7055
3.6868
3.6631
3.6509
3.6271
1.9705
0.6180
0.5942
0.5704
S
Me
OEt
O
HN
N
O
N
Bn
R
10b: R = 5-F
2.99
3.14
1.06
1.00
1.03
4.07
1.07
1.00
1.01
1.03
3.05
2.96
2.96
f1 (ppm)

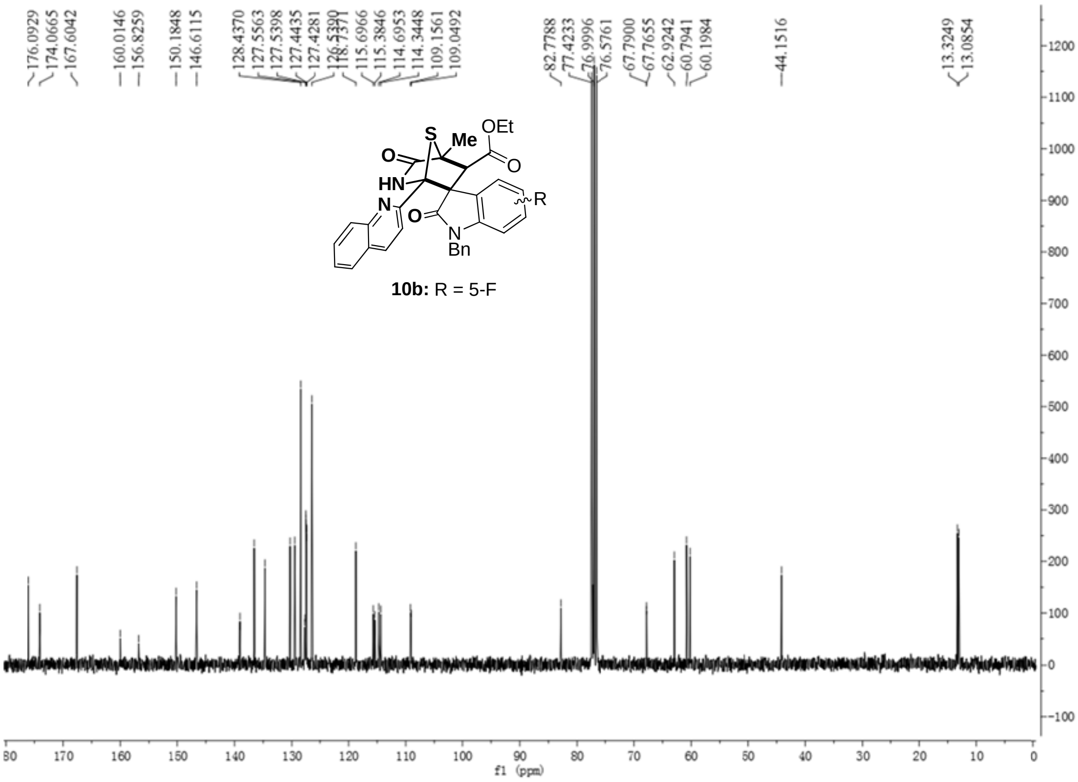
176.0929
174.0665
167.6042
160.0146
156.8259
150.1848
146.6115
128.4370
127.5563
127.5398
127.4435
127.4281
126.5390
118.7371
115.6966
115.3846
114.6953
114.3448
109.1561
109.0492
82.7788
77.4233
76.9996
76.5761
67.7900
67.7655
62.9242
60.7941
60.1984
44.1516
13.3249
13.0854
S
Me
OEt
O
HN
N
O
N
Bn
R
10b: R = 5-F
f1 (ppm)

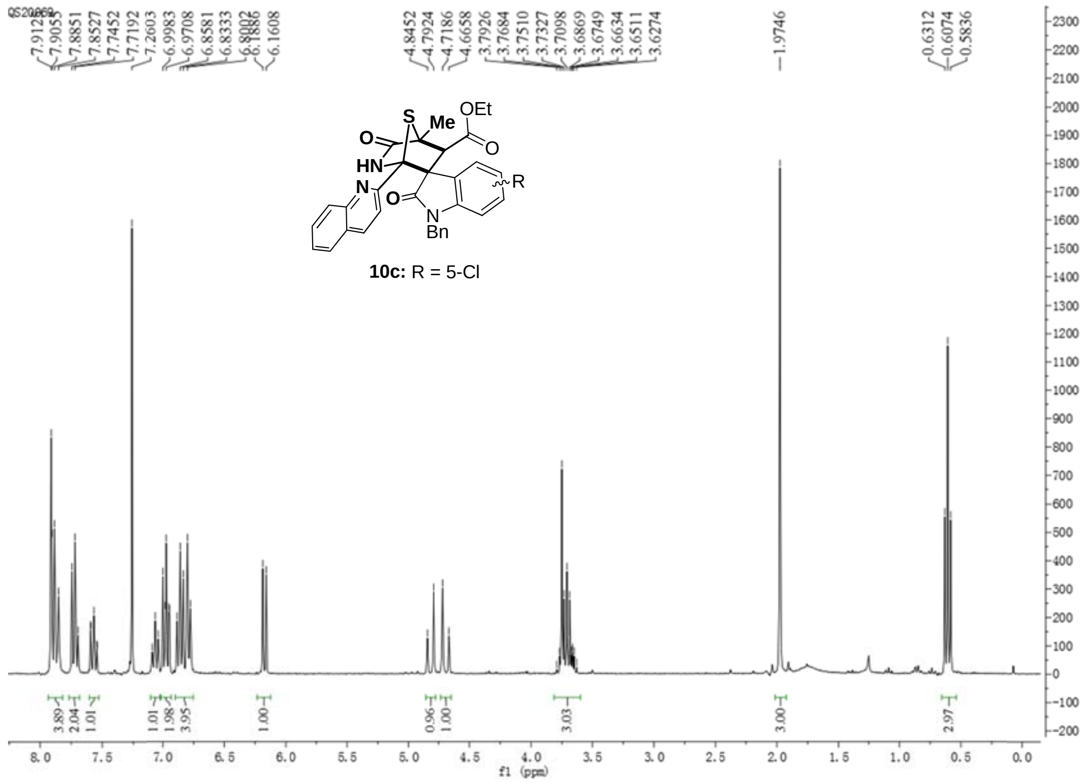
QS20069
10c: R = 5-Cl
f1 (ppm)

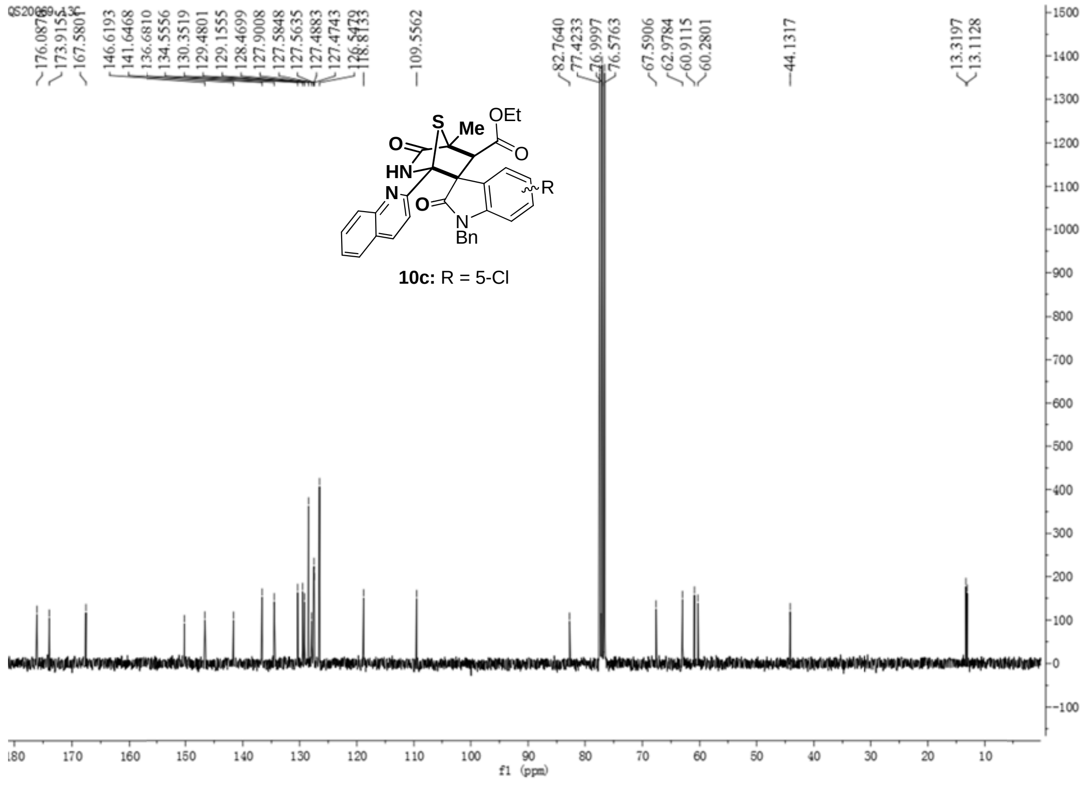
QS20069-13C
10c: R = 5-Cl
f1 (ppm)

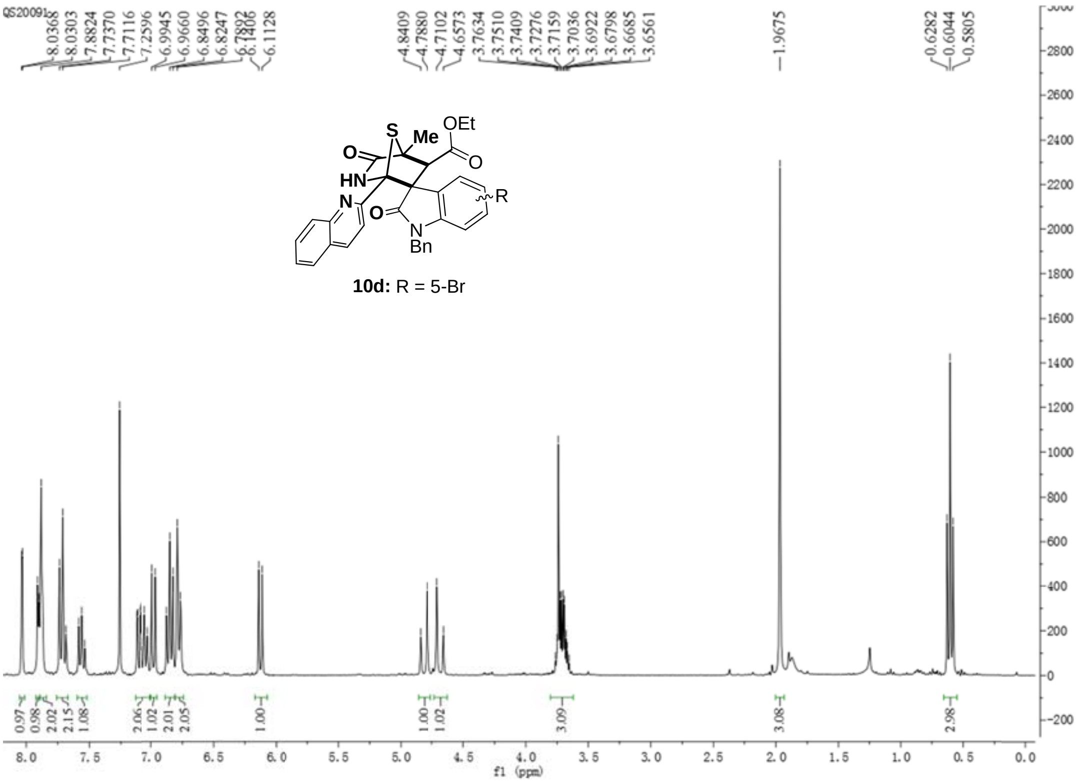
QS20091
8.0368
8.0303
7.8824
7.7370
7.7116
7.2596
6.9945
6.9660
6.8496
6.8247
6.7892
6.1406
6.1128
4.8409
4.7880
4.7102
4.6573
3.7634
3.7510
3.7409
3.7276
3.7159
3.7036
3.6922
3.6798
3.6685
3.6561
1.9675
0.6282
0.6044
0.5805
S
Me
OEt
O
O
HN
N
R
O
N
Bn
10d: R = 5-Br
f1 (ppm)

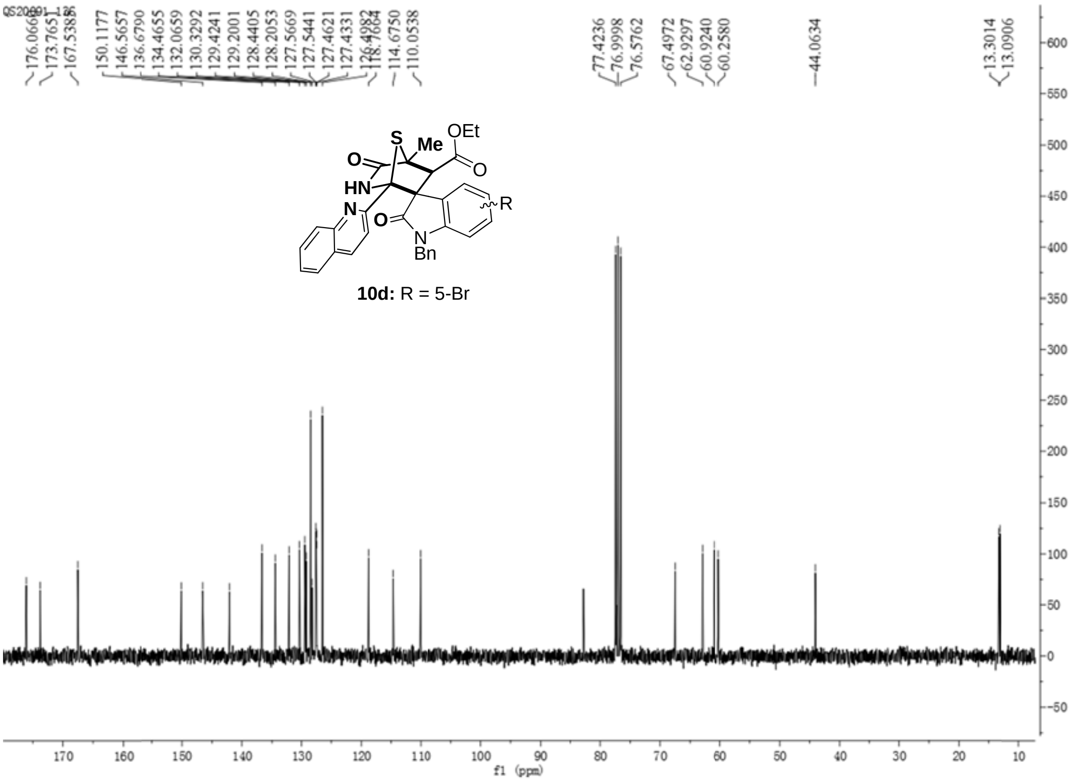
176.0660
173.7651
167.5385
150.1177
146.5657
136.6790
134.4655
132.0659
130.3292
129.4241
129.2001
128.4405
128.2053
127.5669
127.5441
127.4621
127.4331
126.4982
118.7684
114.6750
110.0538
77.4236
76.9998
76.5762
67.4972
62.9297
60.9240
60.2580
44.0634
13.3014
13.0906
S
Me
OEt
O
O
HN
N
R
O
N
Bn
10d: R = 5-Br
f1 (ppm)

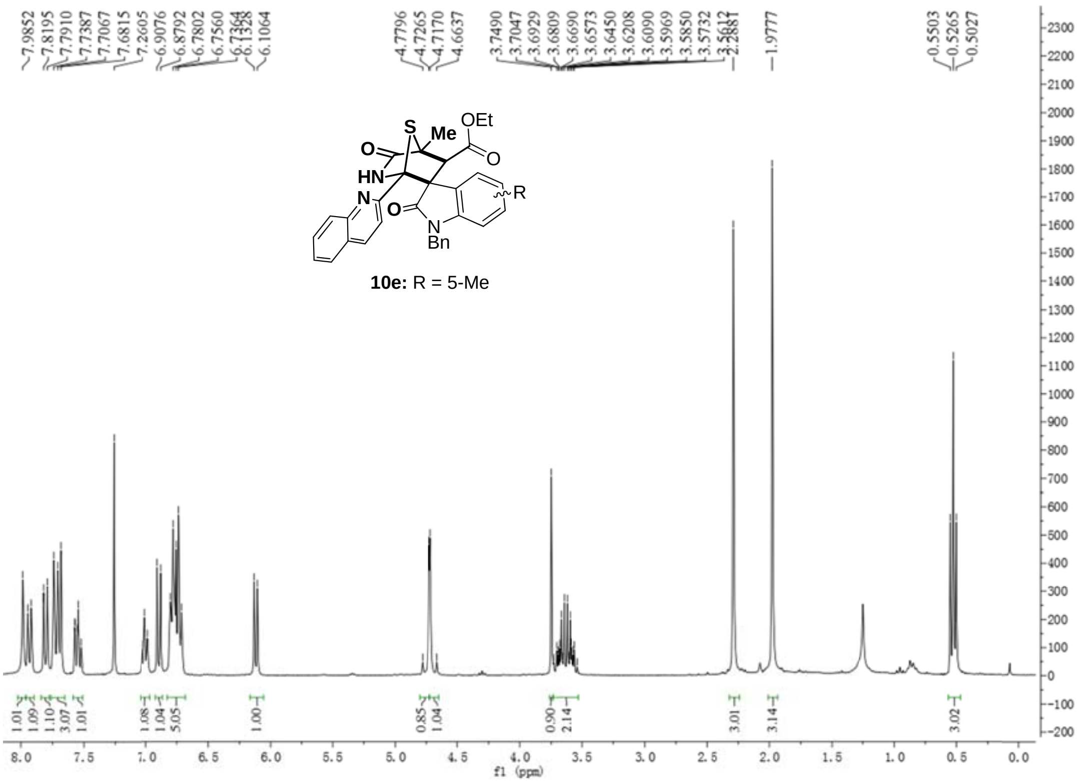
7.9852
7.8195
7.7910
7.7387
7.7067
7.6815
7.2605
6.9076
6.8792
6.7802
6.7560
6.1328
6.1064
4.7796
4.7265
4.7170
4.6637
3.7490
3.7047
3.6929
3.6809
3.6690
3.6573
3.6450
3.6208
3.6090
3.5969
3.5850
3.5732
1.9777
0.5503
0.5265
0.5027
OEt
S
Me
O
O
HN
N
O
N
R
Bn
10e: R = 5-Me
f1 (ppm)

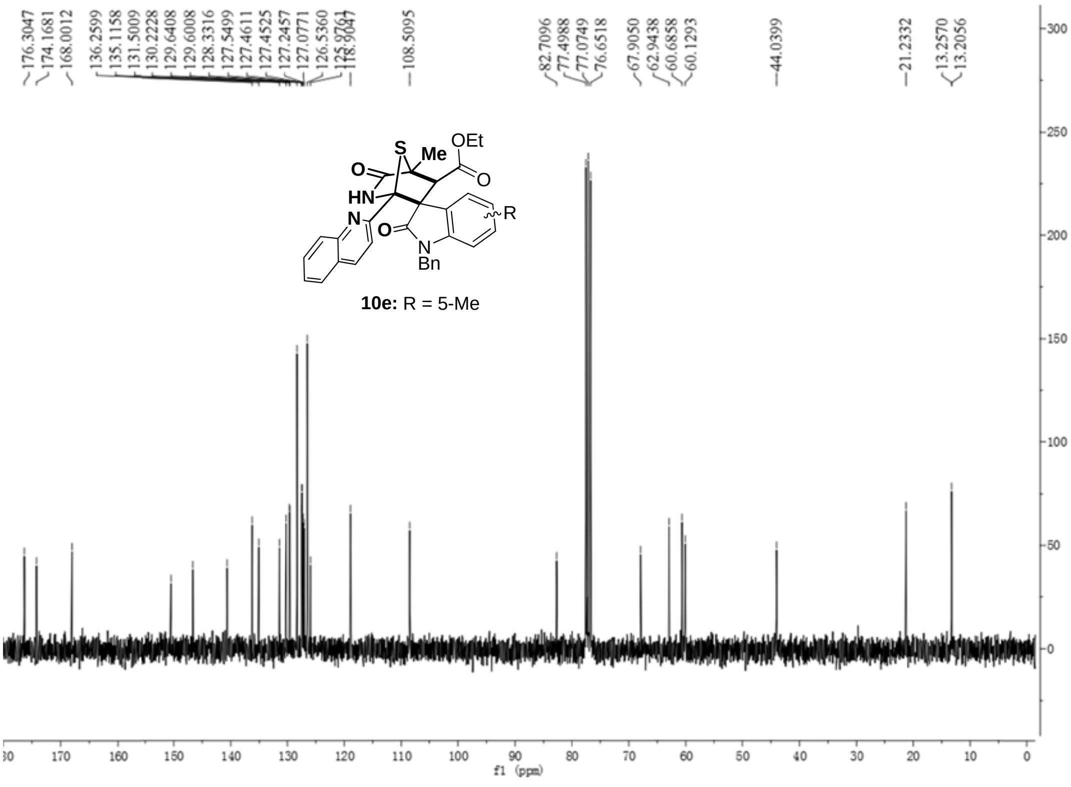
176.3047
174.1681
168.0012
136.2599
135.1158
131.5009
130.2228
129.6408
129.6008
128.3316
127.5499
127.4611
127.4525
127.2457
127.0771
126.5360
108.5095
82.7096
77.4988
77.0749
76.6518
67.9050
62.9438
60.6858
60.1293
44.0399
21.2332
13.2570
13.2056
OEt
S
Me
O
O
HN
N
O
N
R
Bn
10e: R = 5-Me
f1 (ppm)

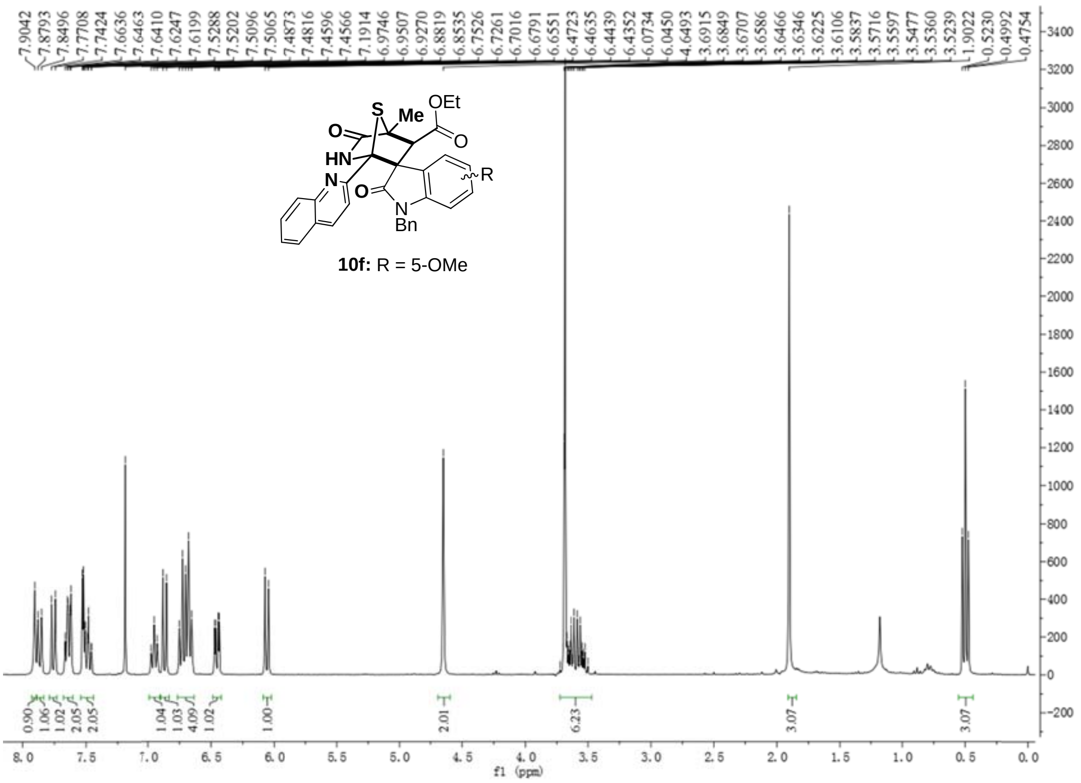
7.9042
7.8793
7.8496
7.7708
7.7424
7.6636
7.6463
7.6410
7.6247
7.6199
7.5288
7.5202
7.5096
7.5065
7.4873
7.4816
7.4596
7.4566
7.1914
6.9746
6.9507
6.9270
6.8819
6.8535
6.7526
6.7261
6.7016
6.6791
6.6551
6.4723
6.4635
6.4439
6.4352
6.0734
6.0450
4.6493
3.6915
3.6849
3.6707
3.6586
3.6466
3.6346
3.6225
3.6106
3.5837
3.5716
3.5597
3.5477
3.5360
3.5239
1.9022
0.5230
0.4992
0.4754
S
Me
OEt
O
O
HN
N
R
O
N
Bn
10f: R = 5-OMe
0.90
1.06
1.02
2.05
2.05
1.04
1.03
4.09
1.02
1.00
2.01
6.23
3.07
3.07
f1 (ppm)

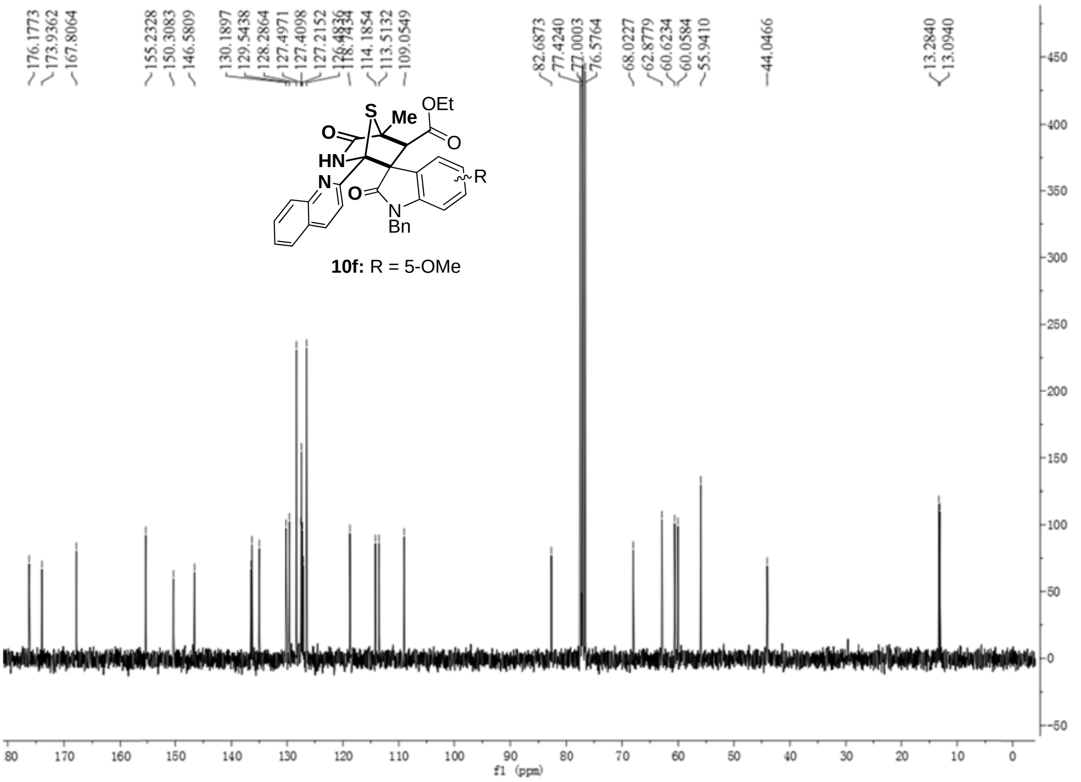
176.1773
173.9362
167.8064
155.2328
150.3083
146.5809
130.1897
129.5438
128.2864
127.4971
127.4098
127.2152
126.4836
118.7434
114.1854
113.5132
109.0549
82.6873
77.4240
77.0003
76.5764
68.0227
62.8779
60.6234
60.0584
55.9410
44.0466
13.2840
13.0940
S
Me
OEt
O
O
HN
N
R
O
N
Bn
10f: R = 5-OMe
f1 (ppm)

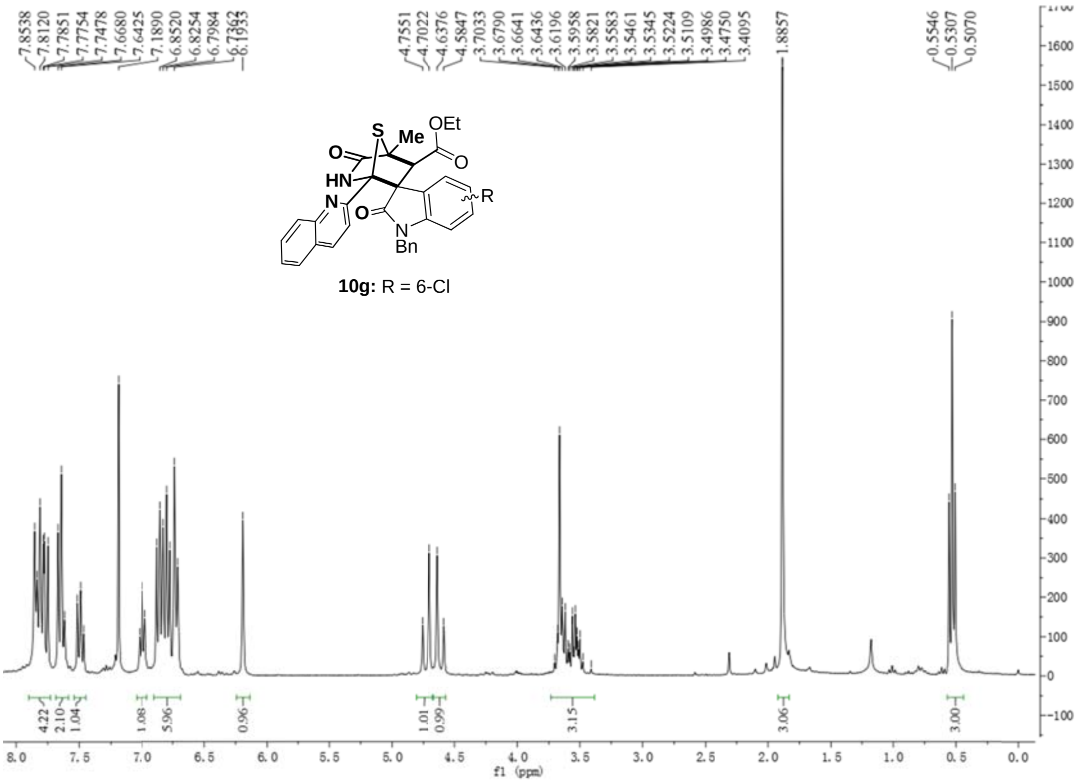
7.8538
7.8120
7.7851
7.7754
7.7478
7.6680
7.6425
7.1890
6.8520
6.8254
6.7984
6.7362
6.1953
4.7551
4.7022
4.6376
4.5847
3.7033
3.6790
3.6641
3.6436
3.6196
3.5958
3.5821
3.5583
3.5461
3.5345
3.5224
3.5109
3.4986
3.4750
3.4095
1.8857
0.5546
0.5307
0.5070
S
Me
OEt
O
O
HN
N
O
N
Bn
R
10g: R = 6-Cl
4.22
2.10
1.04
1.08
5.96
0.96
1.01
0.99
3.15
3.06
3.00
f1 (ppm)

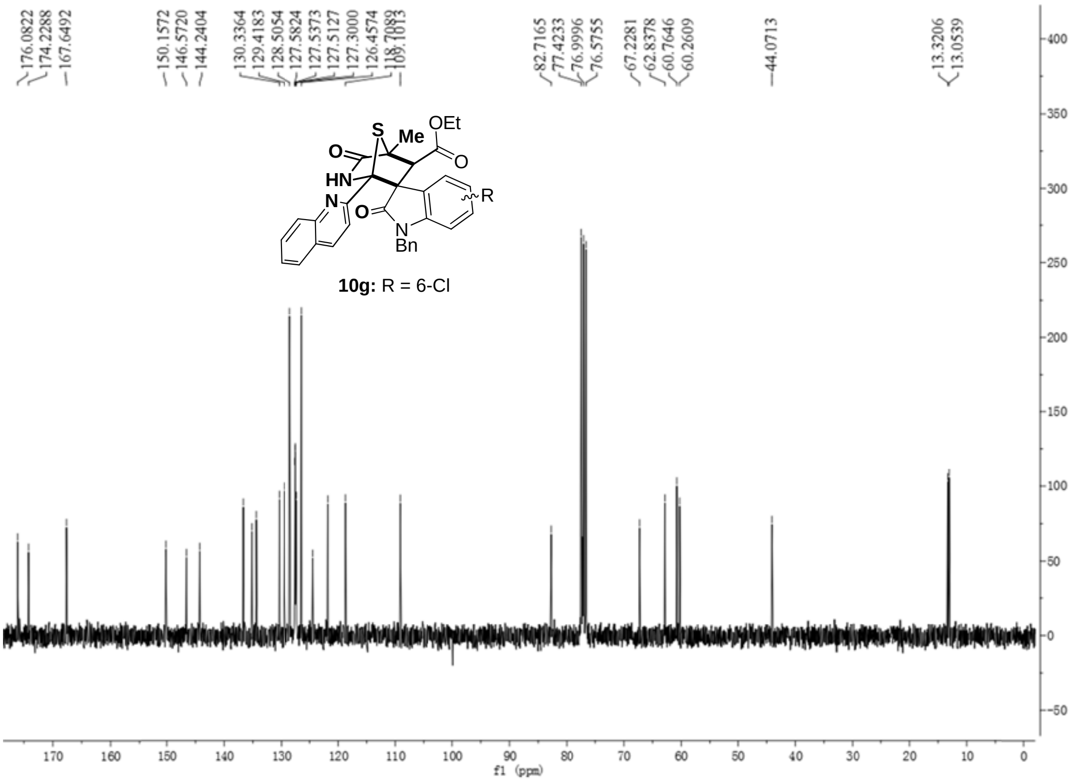
176.0822
174.2288
167.6492
150.1572
146.5720
144.2404
130.3364
129.4183
128.5054
127.5824
127.5373
127.5127
127.3000
126.4574
118.7089
109.1013
82.7165
77.4233
76.9996
76.5755
67.2281
62.8378
60.7646
60.2609
44.0713
13.3206
13.0539
S
Me
OEt
O
O
HN
N
O
N
Bn
R
10g: R = 6-Cl
f1 (ppm)

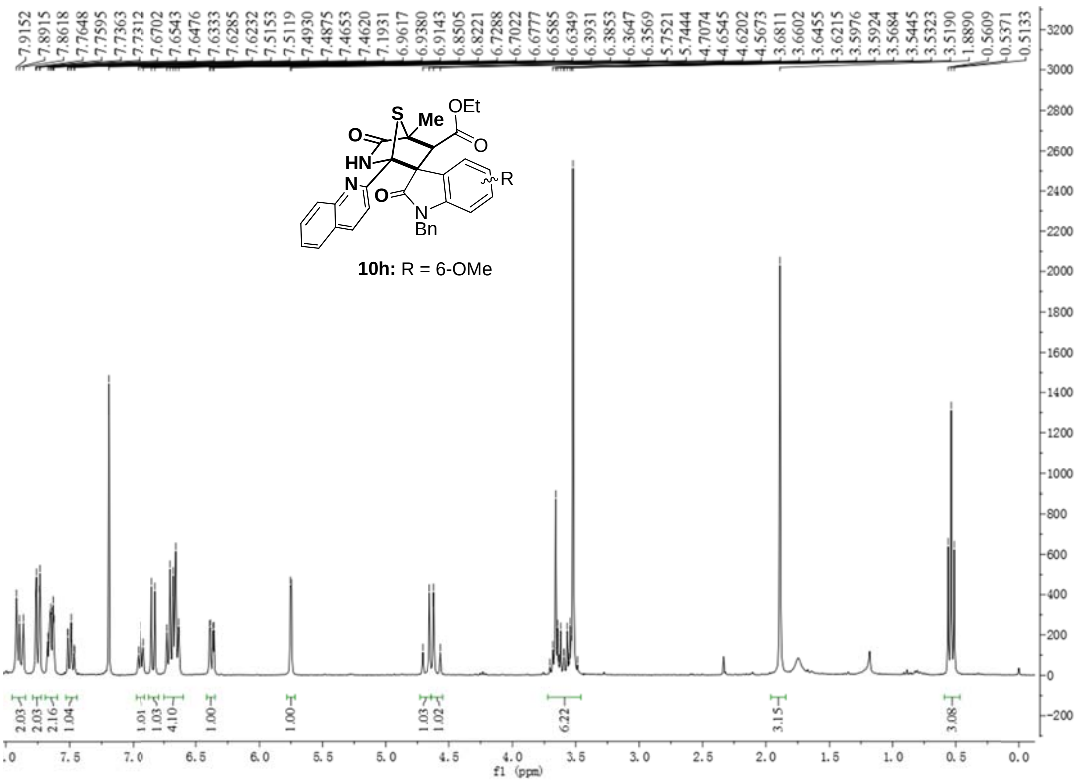

10h: R = 6-OMe
f1 (ppm)

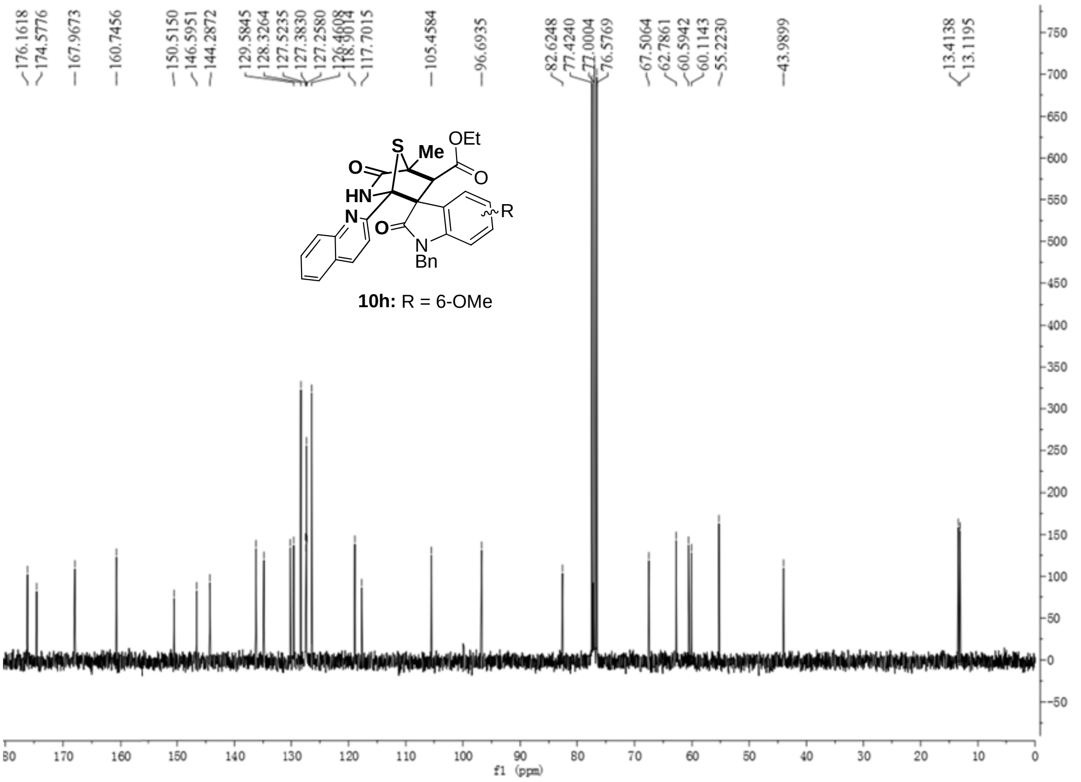

10h: R = 6-OMe
f1 (ppm)

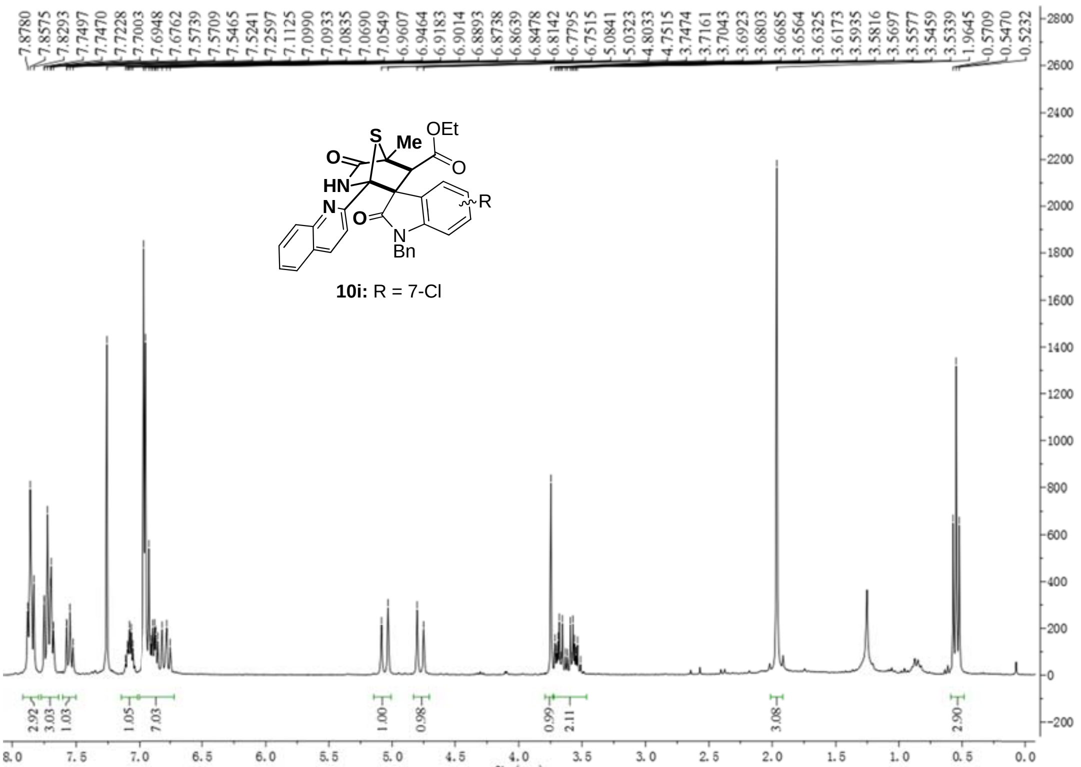

10i: R = 7-Cl

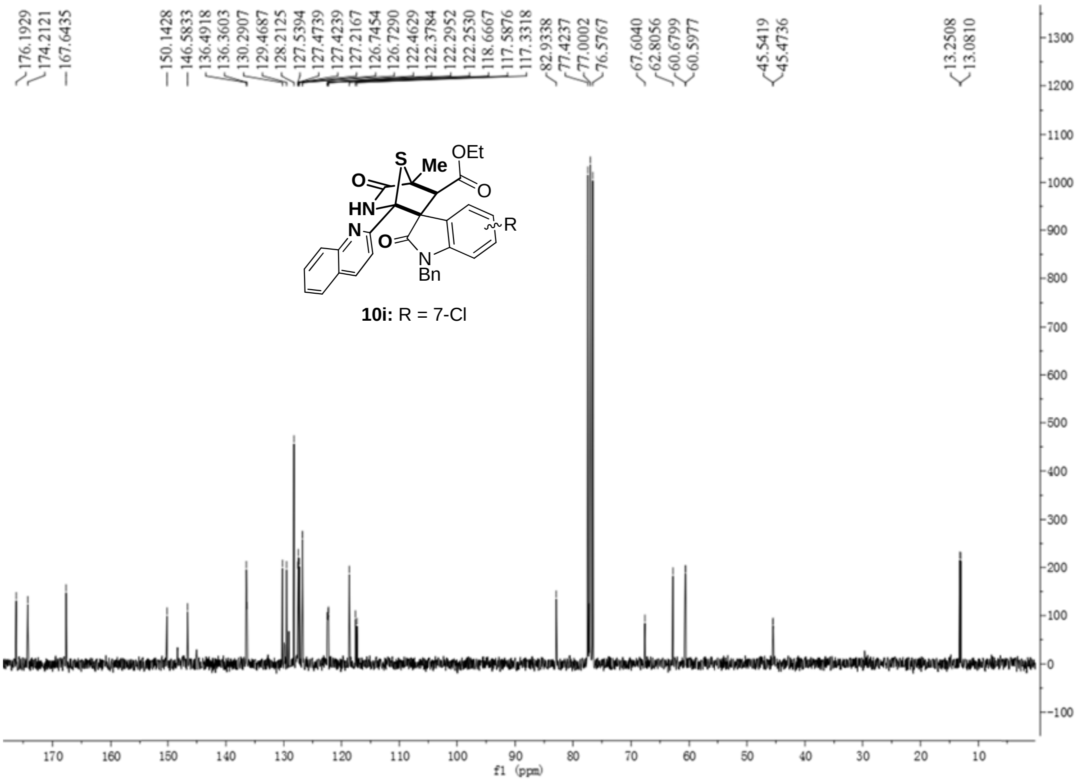

10i: R = 7-Cl

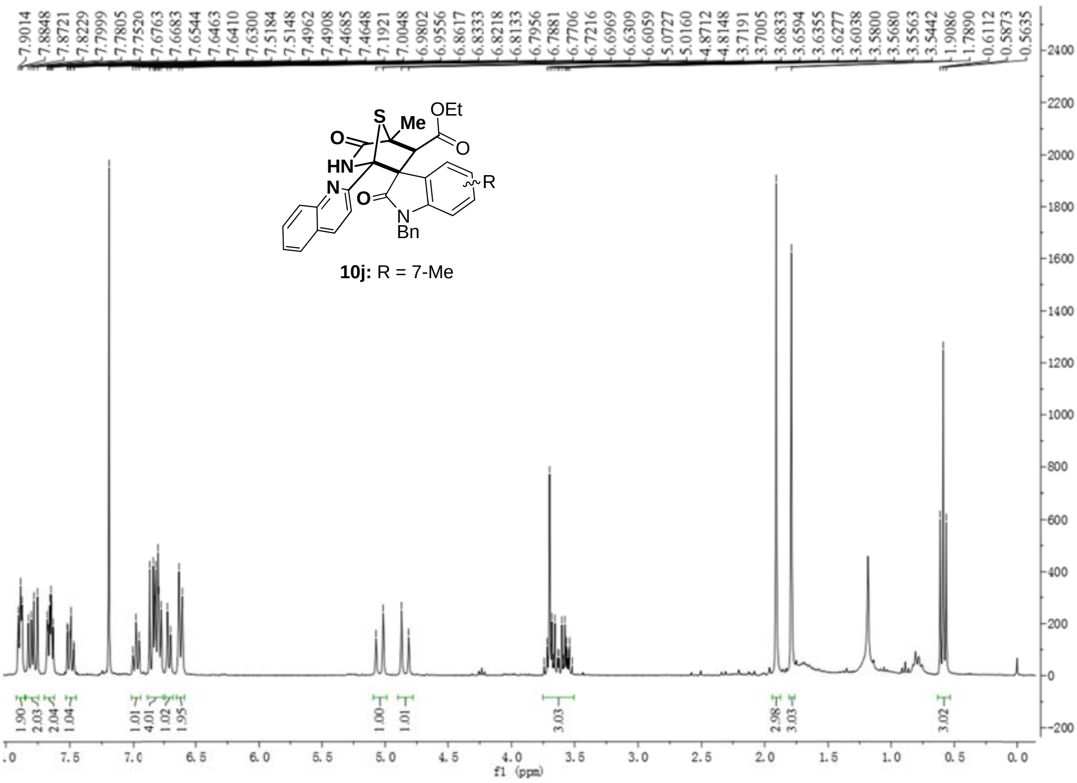

10j: R = 7-Me
f1 (ppm)

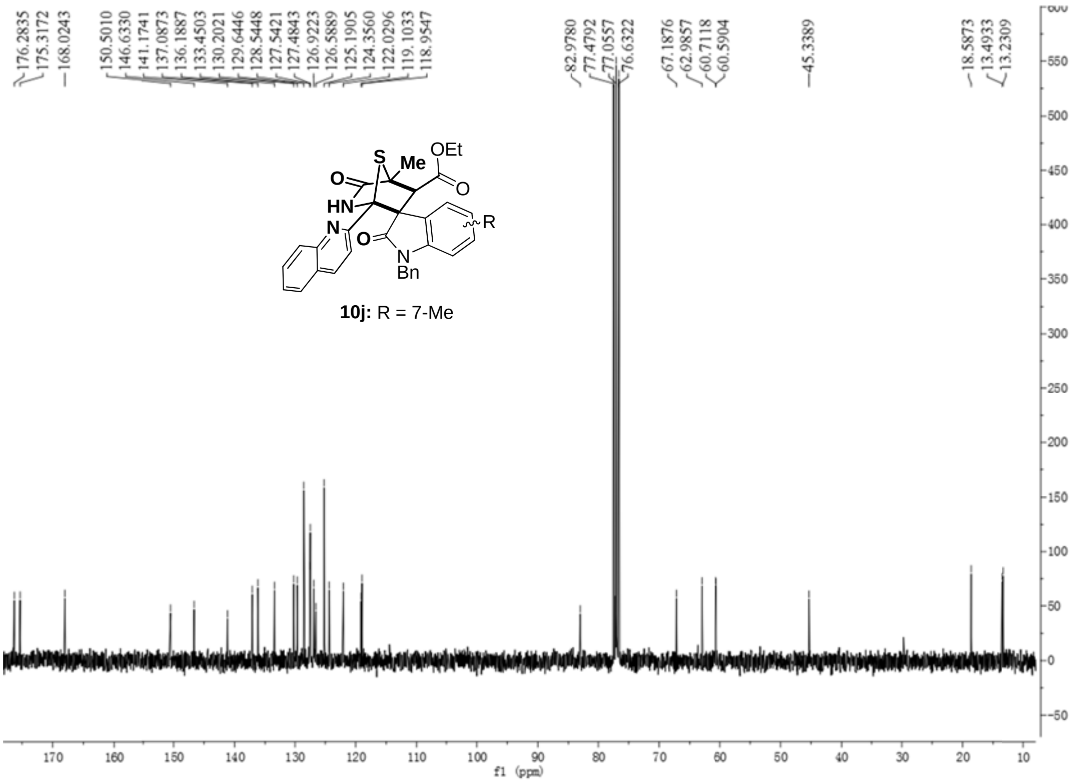

10j: R = 7-Me
f1 (ppm)

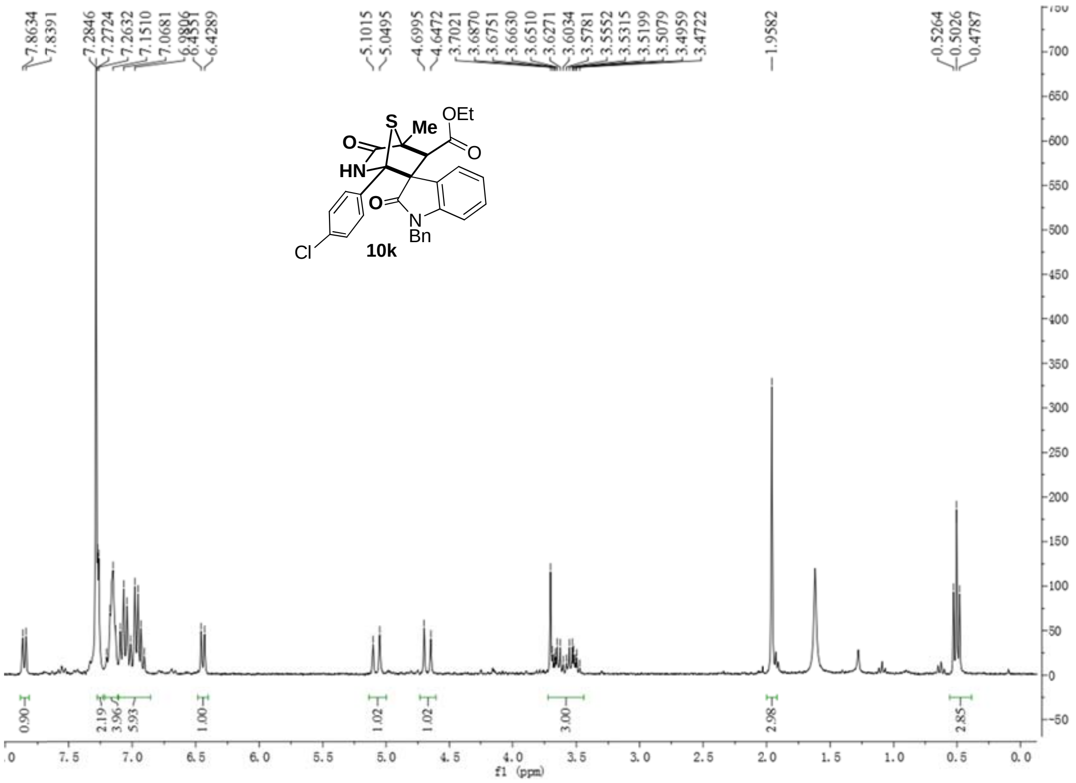
S
Me
OEt
O
O
HN
O
N
Bn
Cl
10k
f1 (ppm)

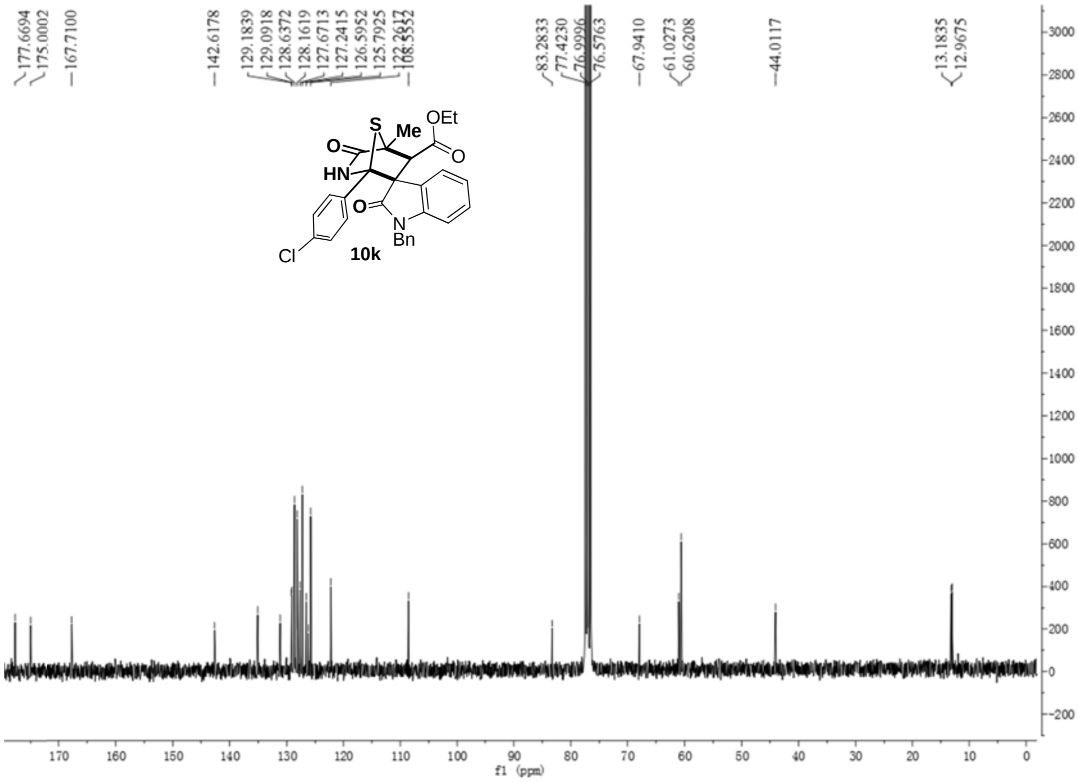
S
Me
OEt
O
O
HN
O
N
Bn
Cl
10k
f1 (ppm)

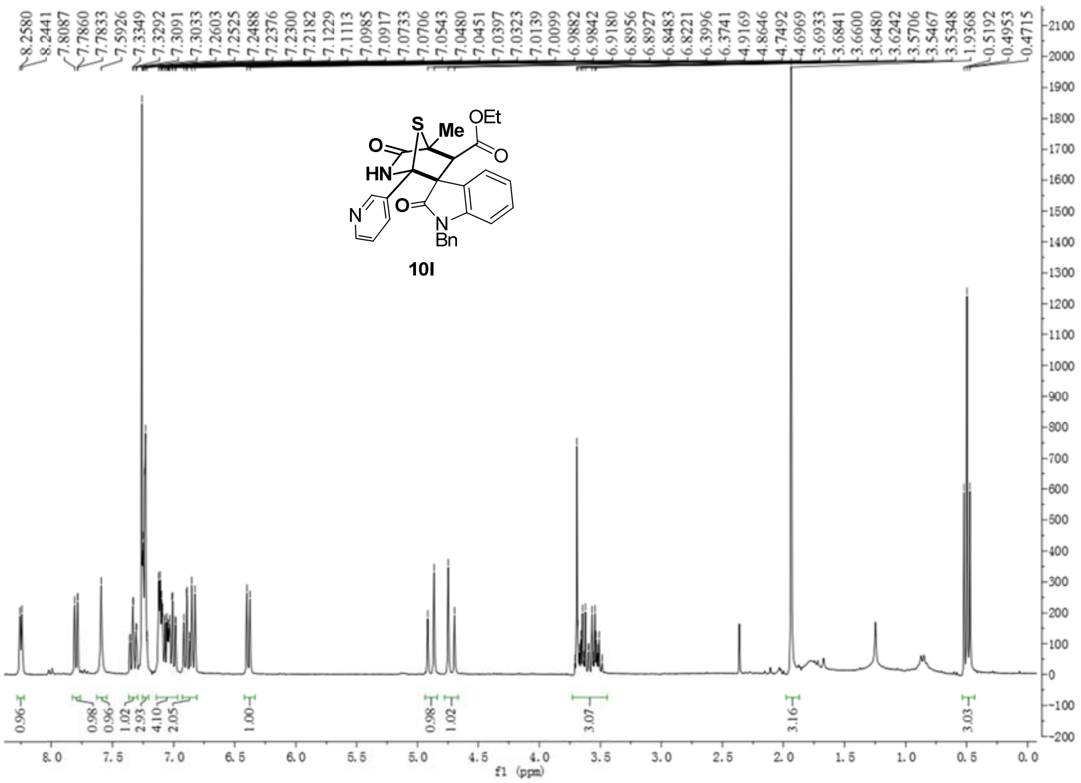
8.2580
8.2441
7.8087
7.7860
7.7833
7.5926
7.3349
7.3292
7.3091
7.3033
7.2603
7.2525
7.2488
7.2376
7.2300
7.2182
7.1229
7.1113
7.0985
7.0917
7.0733
7.0706
7.0543
7.0480
7.0451
7.0397
7.0323
7.0139
7.0099
6.9882
6.9842
6.9180
6.8956
6.8927
6.8483
6.8221
6.3996
6.3741
4.9169
4.8646
4.7492
4.6969
3.6933
3.6841
3.6600
3.6480
3.6242
3.5706
3.5467
3.5348
1.9368
0.5192
0.4953
0.4715
S
Me
OEt
O
O
HN
N
O
N
Bn
10l
0.96
0.98
0.96
1.02
2.93
4.10
2.05
1.00
0.98
1.02
3.07
3.16
3.03
f1 (ppm)

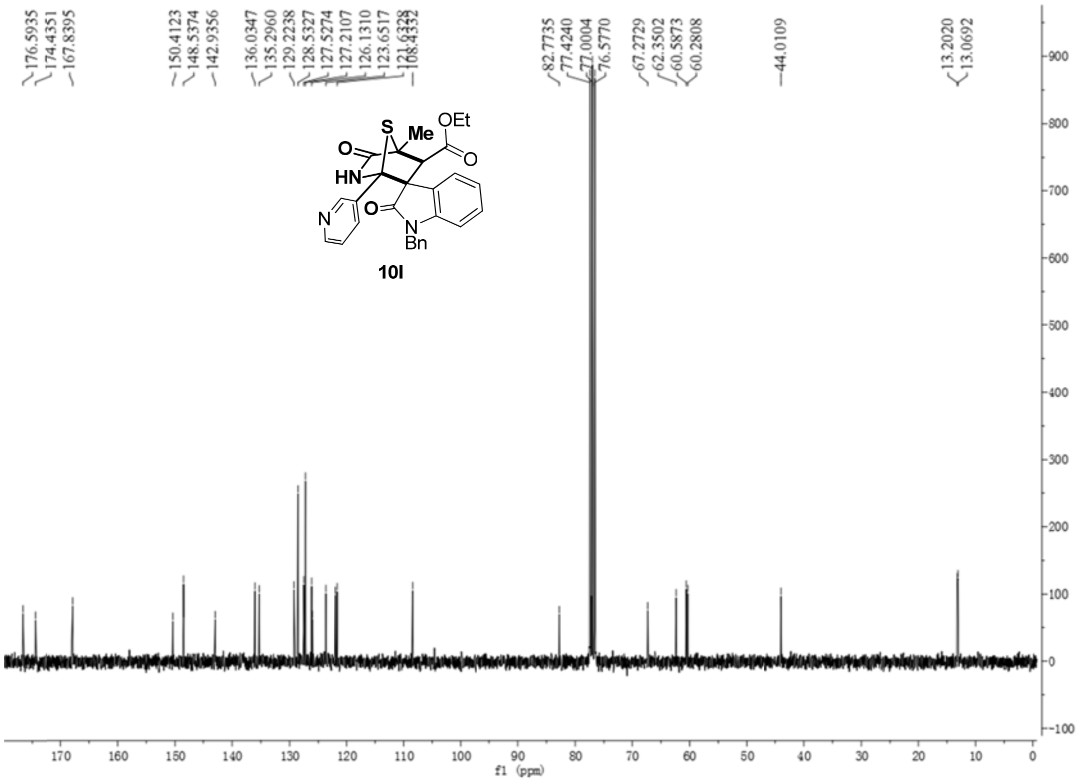
176.5935
174.4351
167.8395
150.4123
148.5374
142.9356
136.0347
135.2960
129.2238
128.5327
127.5274
127.2107
126.1310
123.6517
82.7735
77.4240
77.0004
76.5770
67.2729
62.3502
60.5873
60.2808
44.0109
13.2020
13.0692
S
Me
OEt
O
O
HN
N
O
N
Bn
10l
f1 (ppm)

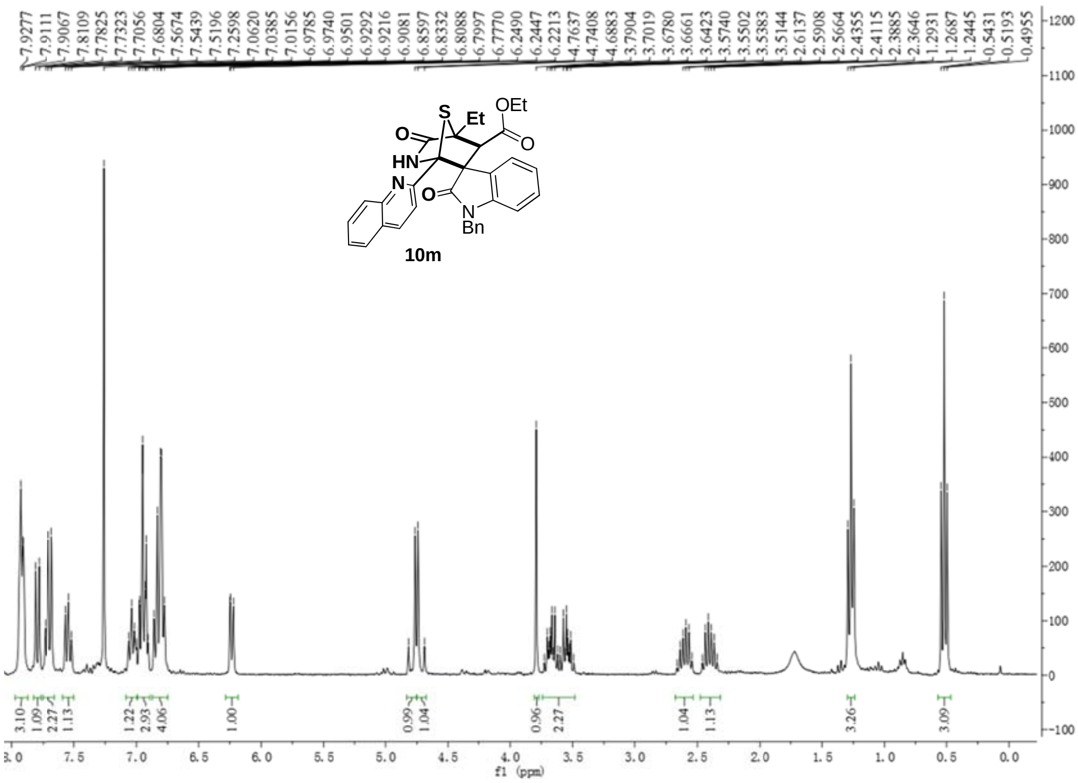
7.9277
7.9111
7.9067
7.8109
7.7825
7.7323
7.7056
7.6804
7.5674
7.5439
7.5196
7.2598
7.0620
7.0385
7.0156
6.9785
6.9740
6.9501
6.9292
6.9216
6.9081
6.8597
6.8332
6.8088
6.7997
6.7770
6.2490
6.2447
6.2213
4.7637
4.7408
4.6883
3.7904
3.7019
3.6780
3.6661
3.6423
3.5740
3.5502
3.5383
3.5144
2.6137
2.5908
2.5664
2.4355
2.4115
2.3885
2.3646
1.2931
1.2687
1.2445
0.5431
0.5193
0.4955
OEt
S
Et
O
O
HN
N
O
N
Bn
10m
3.10
1.09
2.27
1.13
1.22
2.93
4.06
1.00
0.99
1.04
0.96
2.27
1.04
1.13
3.26
3.09
f1 (ppm)

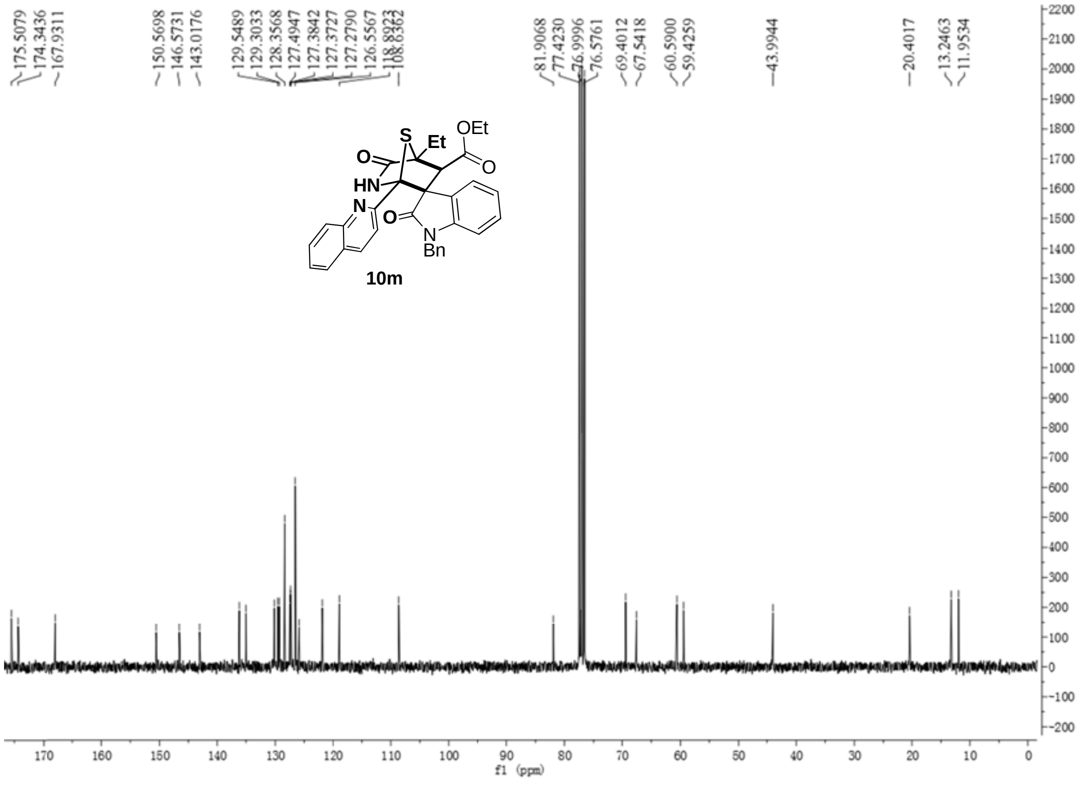
175.5079
174.3436
167.9311
150.5698
146.5731
143.0176
129.5489
129.3033
128.3568
127.4947
127.3842
127.3727
127.2790
126.5567
118.8923
108.8362
81.9068
77.4230
76.9996
76.5761
69.4012
67.5418
60.5900
59.4259
43.9944
20.4017
13.2463
11.9534
OEt
S
Et
O
O
HN
N
O
N
Bn
10m
f1 (ppm)

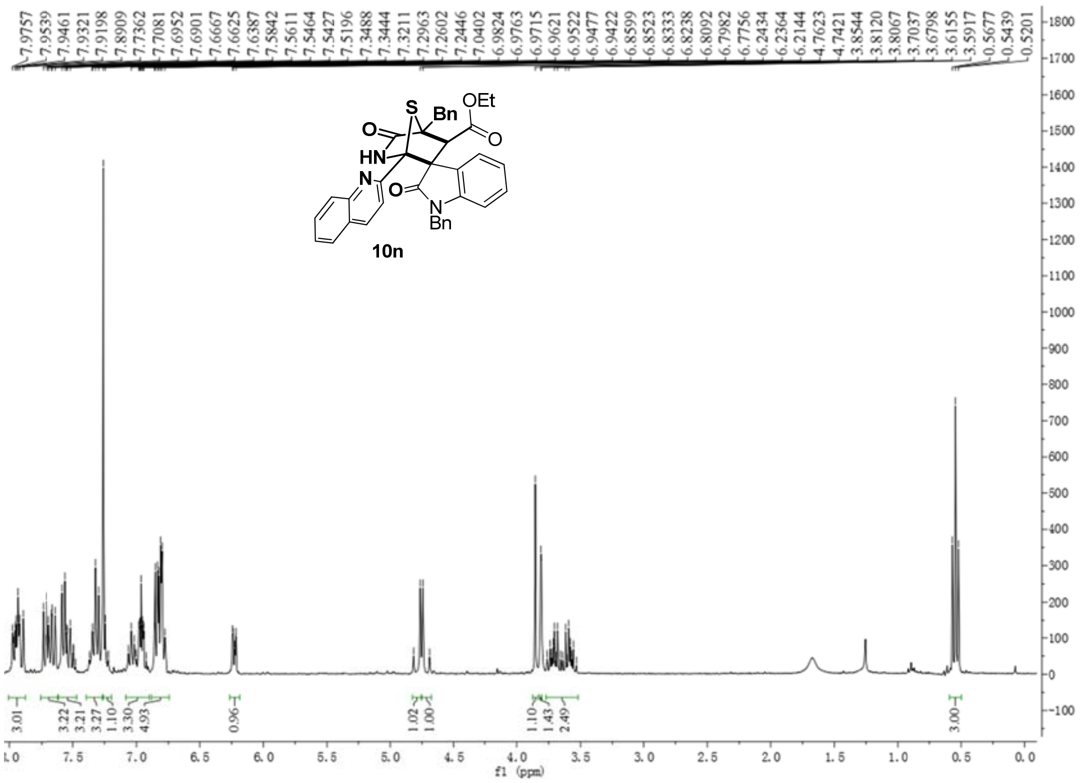

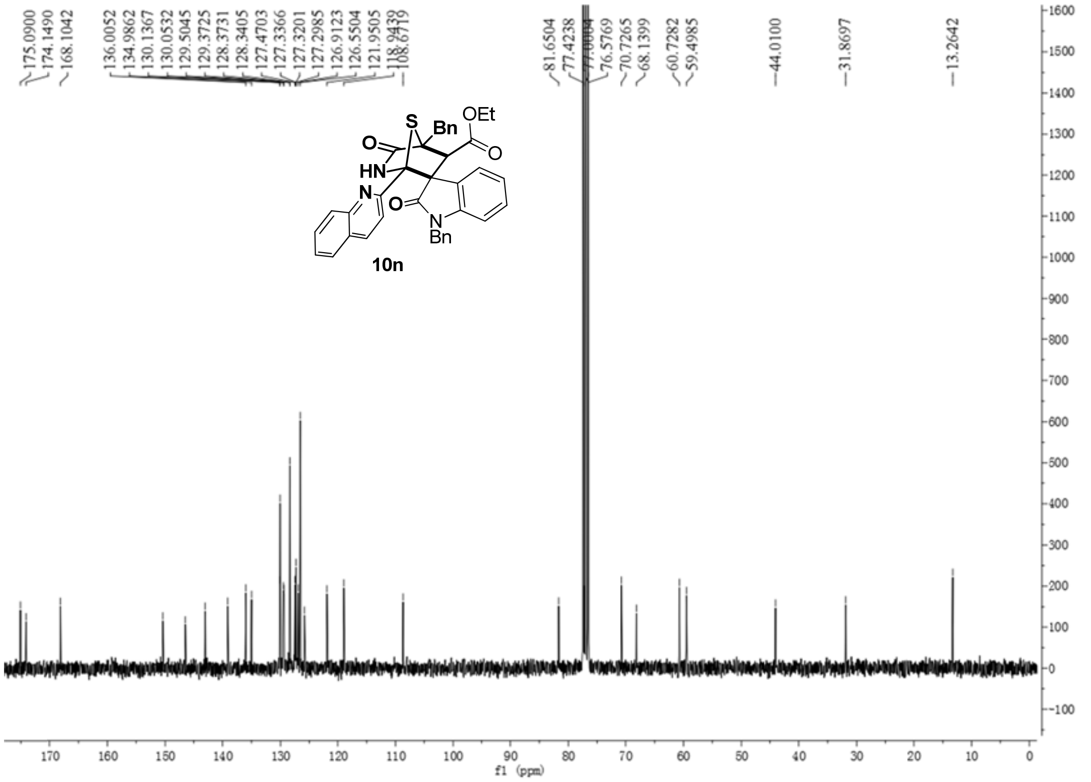

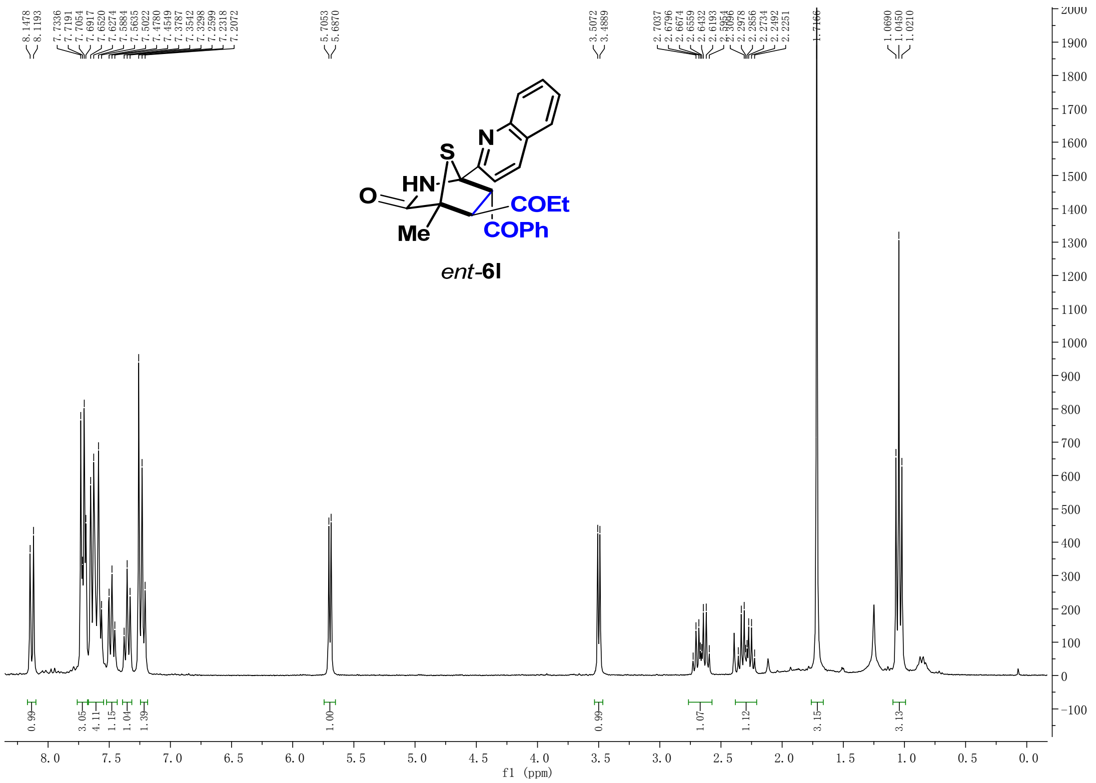
8.1478
8.1193
7.7336
7.7191
7.7054
7.6917
7.6520
7.6274
7.5884
7.5635
7.5022
7.4780
7.4549
7.3787
7.3542
7.3298
7.2599
7.2318
7.2072
5.7053
5.6870
3.5072
3.4889
2.7037
2.6796
2.6674
2.6559
2.6432
2.6193
2.2978
2.2856
2.2734
2.2492
2.2251
1.7166
1.0690
1.0450
1.0210
S
N
HN
O
Me
COEt
COPh
ent-6l
0.99
3.05
4.11
1.15
1.04
1.39
1.00
0.99
1.07
1.12
3.15
3.13
f1 (ppm)

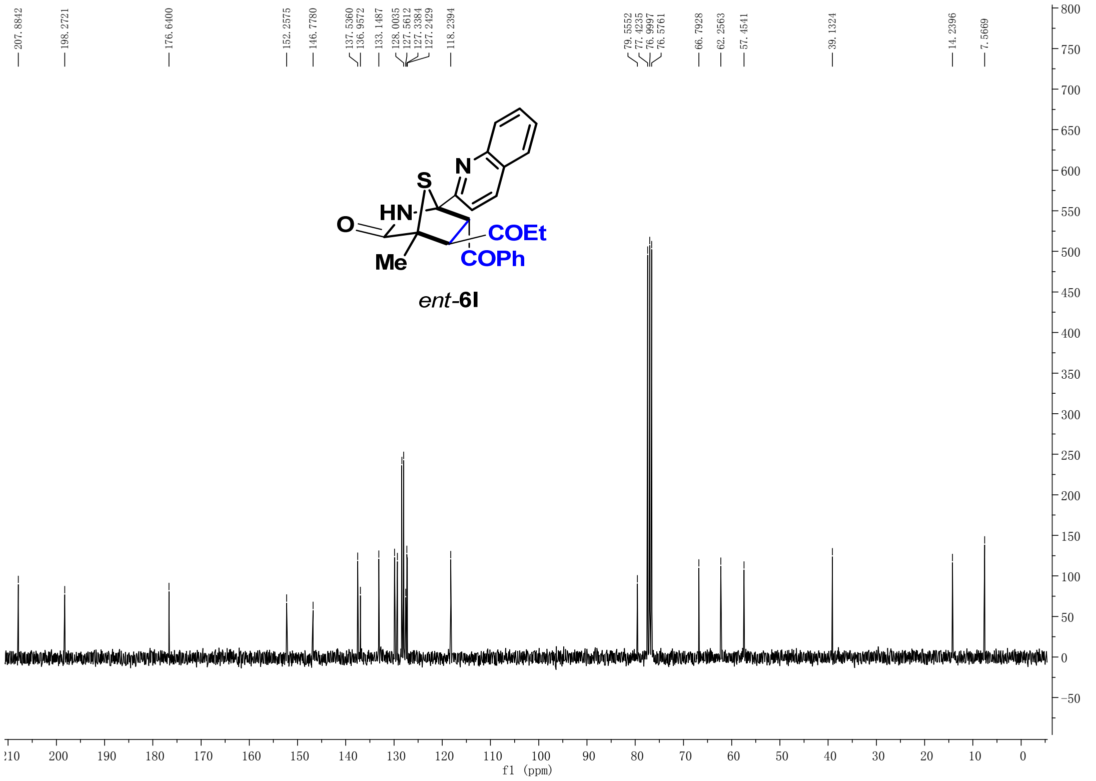
207.8842
198.2721
176.6400
152.2575
146.7780
137.5360
136.9572
133.1487
128.0035
127.5612
127.3384
127.2429
118.2394
79.5552
77.4235
76.9997
76.5761
66.7928
62.2563
57.4541
39.1324
14.2396
7.5669
S
N
HN
O
Me
COEt
COPh
ent-6l
f1 (ppm)

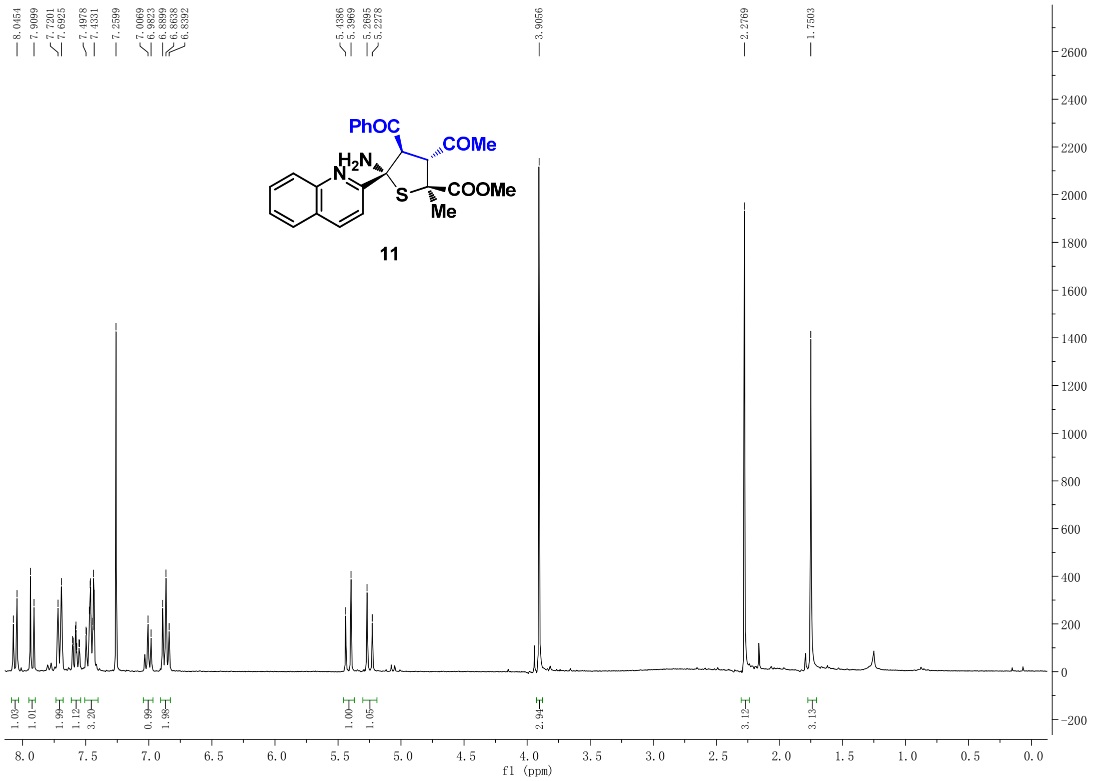
8.0454
7.9099
7.7201
7.6925
7.4978
7.4331
7.2599
7.0069
6.9823
6.8899
6.8638
6.8392
5.4386
5.3969
5.2695
5.2278
3.9056
2.2769
1.7503
PhOC
COMe
H_2N
N
S
COOMe
Me
11
1.03
1.01
1.99
1.12
3.20
0.99
1.98
1.00
1.05
2.94
3.12
3.13
f1 (ppm)

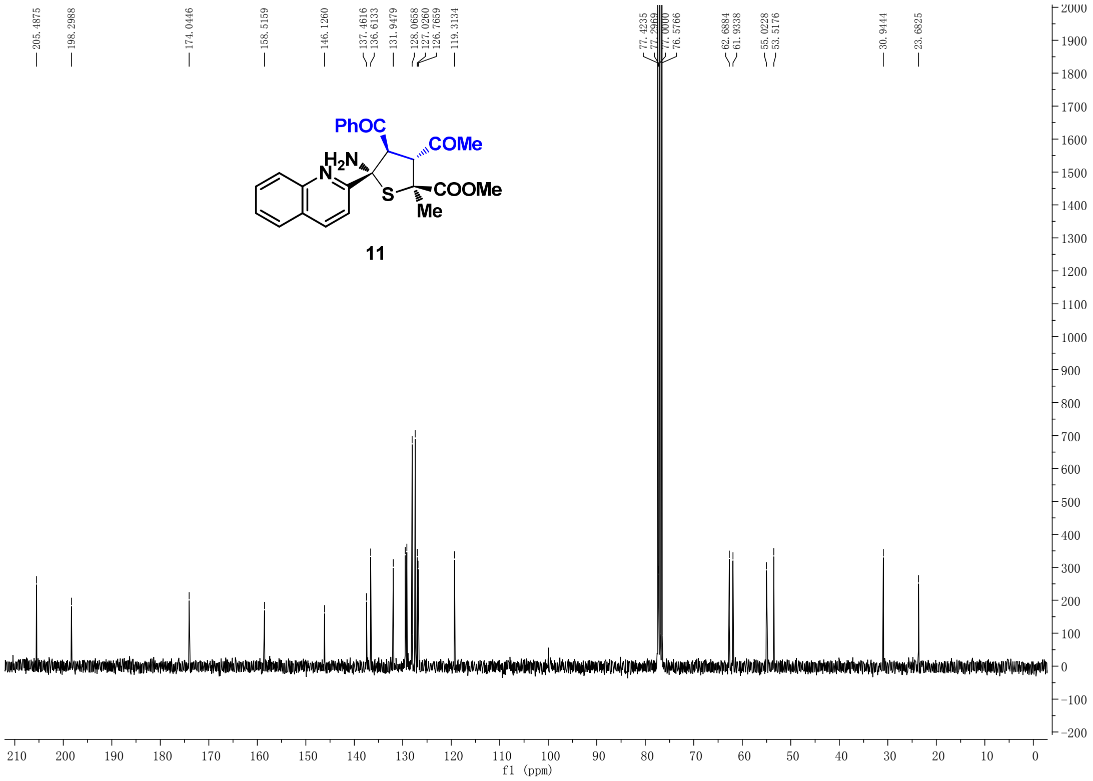
205.4875
198.2988
174.0446
158.5159
146.1260
137.4616
136.6133
131.9479
128.0658
127.0260
126.7659
119.3134
77.4235
76.5766
62.6884
61.9338
55.0228
53.5176
30.9444
23.6825
PhOC
COMe
H_2N
N
S
COOMe
Me
11
f1 (ppm)

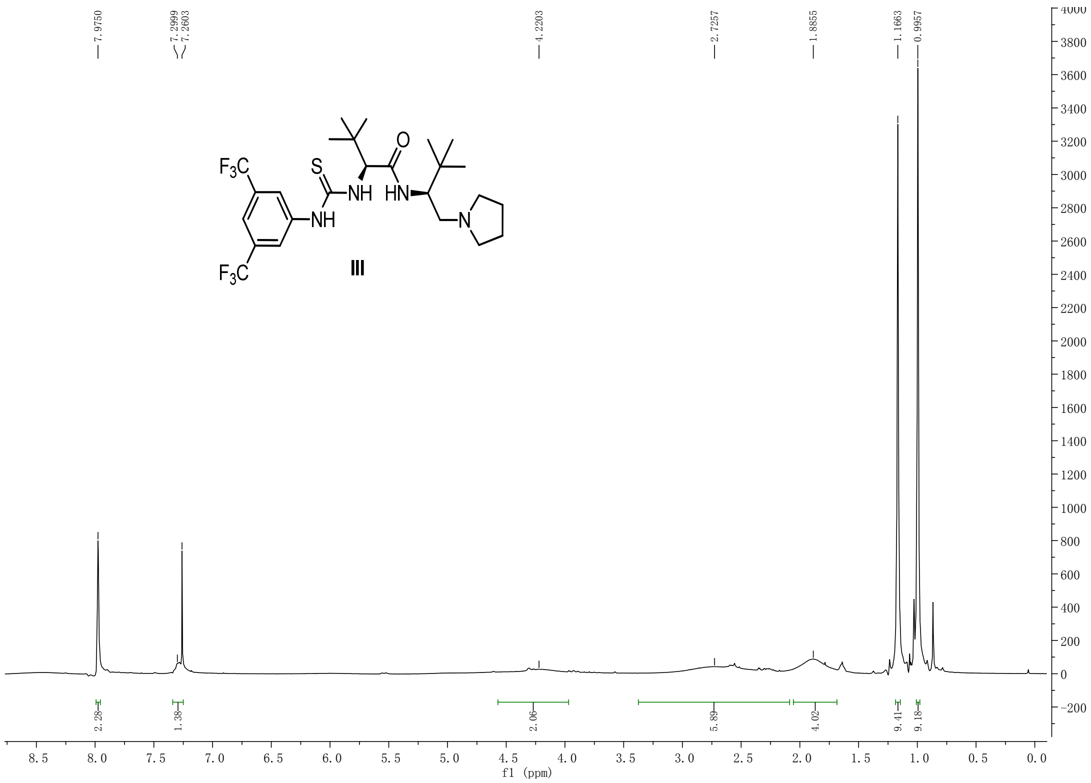

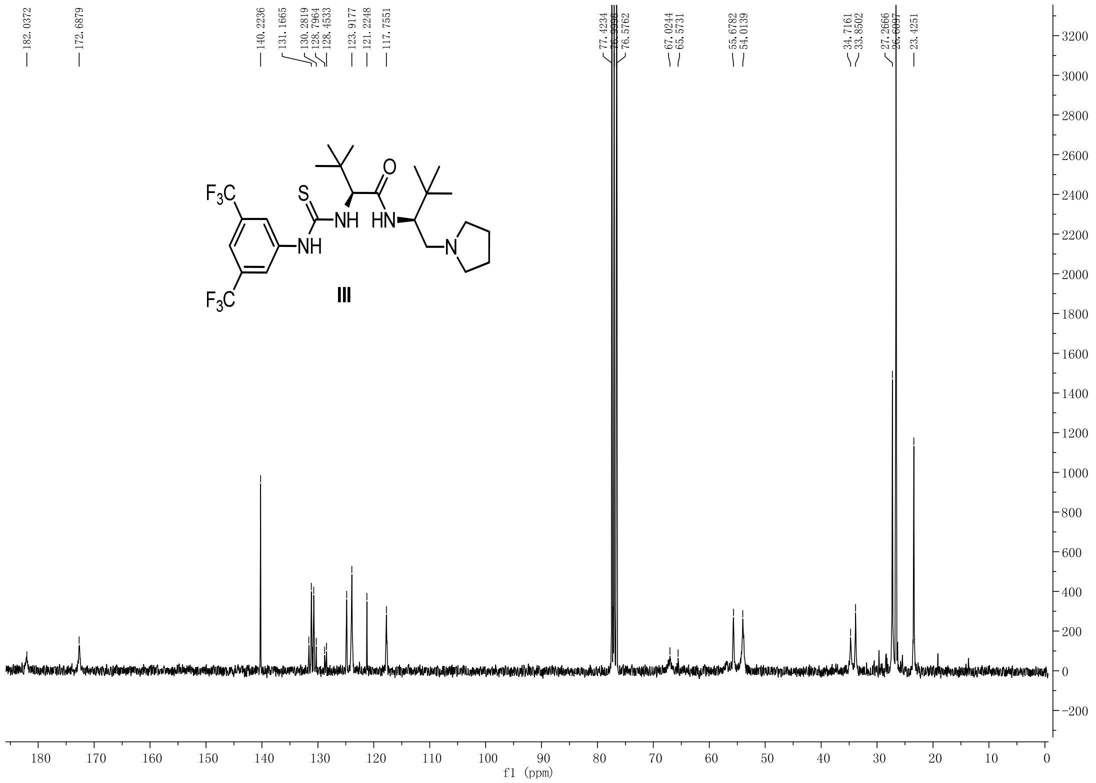

Supplement: Supplementary file 1 [file SC-007-C6SC02039A-s001.pdf]
